# Underreporting of non-study cigarette use by study participants confounds the interpretation of results from ambulatory clinical trial of reduced nicotine cigarettes

**DOI:** 10.1186/s12954-024-00953-8

**Published:** 2024-02-08

**Authors:** Mingda Zhang, Jingzhu Wang, Jeffery Edmiston

**Affiliations:** Altria Client Services LLC, 601 E. Jackson Street, Richmond, VA 23219 USA

**Keywords:** Reduced nicotine cigarette, Tobacco harm reduction, Clinical trial, Non-compliance

## Abstract

**Background:**

As part of its comprehensive plan to significantly reduce the harm from tobacco products, the US Food and Drug Administration is establishing a product standard to lower nicotine in conventional cigarettes to make them “minimally addictive or non-addictive". Many clinical studies have investigated the potential impact of such a standard on smoking behavior and exposure to cigarette constituents. These ambulatory studies required participants who smoke to switch to reduced nicotine study cigarettes. In contrast to clinical trials on pharmaceuticals or medical devices, participants had ready access to non-study conventional nicotine cigarettes and high rates of non-study cigarette use were consistently reported. The magnitude of non-compliance, which could impact the interpretation of the study results, was not adequately assessed in these trials.

**Methods:**

We conducted a secondary analysis of data from a large, randomized trial of reduced nicotine cigarettes with 840 participants to estimate the magnitude of non-compliance, i.e., the average number of non-study cigarettes smoked per day by study participants assigned to reduced nicotine cigarettes. Individual participants’ non-study cigarette use was estimated based on his/her urinary total nicotine equivalent level, the nicotine content of the study cigarette assigned and the self-reported number of cigarettes smoked, using a previously published method.

**Results:**

Our analysis showed that (1) there is a large variation in the number of non-study cigarettes smoked by participants within each group (coefficient of variation 90–232%); (2) participants in reduced nicotine cigarette groups underreported their mean number of non-study cigarettes smoked per day by 85–91%; and (3) the biochemical-based estimates indicate no reduction in the mean number of total cigarettes smoked per day for any group assigned to reduced nicotine cigarettes after accounting for non-study cigarettes.

**Conclusions:**

High levels of non-compliance, in both the rate and magnitude of non-study cigarette use, are common in ambulatory reduced nicotine cigarette trials where participants have access to conventional nicotine non-study cigarettes. The potential impact of high non-compliance on study outcomes should be considered when interpreting the results from such ambulatory studies.

**Supplementary Information:**

The online version contains supplementary material available at 10.1186/s12954-024-00953-8.

## Background

The US Food and Drug Administration (FDA) plans to establish a tobacco product standard to substantially reduce the nicotine content in conventional combustible cigarettes as a strategy to further reduce the public health impact of smoking. FDA published an advance notice of proposed rulemaking to set a maximum nicotine level in cigarettes so “they are minimally addictive or non-addictive, using the best available science” in 2018 [1]. In 2022, FDA announced its plan to issue a proposed product standard that would establish a maximum nicotine level to reduce the addictiveness of cigarettes “with the goal of reducing youth use, addiction and death” [2]. Results from clinical studies that examine the impact of switching to reduced nicotine cigarettes (RNC) on smoking cessation, smoking behavior and exposure to cigarette constituents will likely serve as the key scientific foundation for FDA’s nicotine standard.

High rates of non-compliance with non-study cigarettes have been consistently reported in ambulatory studies where participants were required to exclusively smoke reduced nicotine study cigarettes, but have ready access to conventional nicotine cigarettes (CNC), and a large percentage of them smoked their usual brand cigarettes during the study [3–6]. Except for a few small residential studies in which participants were confined in a hotel setting where access to all tobacco products was strictly controlled [7, 8], almost all recent clinical studies on extended use of RNC were ambulatory with participants living in their naturalistic settings with ready access to non-study CNC [3, 4, 9–16]. In studies that assessed both self-reported and biochemically verified compliance, non-compliance rates based on urinary nicotine biomarkers were consistently higher than those based on self-report, indicating that many study participants did not report their non-study cigarette use. For example, in the largest clinical study examining participants switching to RNC at various nicotine levels for 6 weeks, participants assigned to cigarettes with the lowest nicotine level (0.4 mg/g tobacco) had non-compliance rates of 76–78% at week 6 based on biomarkers but only 39% based on participants’ self-report [6]. This suggests about half of the participants who smoked non-study cigarettes during week 6 of the study did not report doing so. In addition, the number of non-study cigarettes per day (CPD) reported was low, with the 75th percentile of non-study CPD reported at 2 ([3]; Table S9). The reduction in exposure to nicotine observed among the RNC groups was substantially less than would have been expected from the level of nicotine in the assigned RNC and the number of self-reported non-study CPD [17]. There was no significant reduction in exposure to nicotine-derived nitrosamine ketone (NNK), despite substantially lower levels of NNK in the RNC relative to CNC [3]. The expired carbon monoxide level was not reduced proportionally to the number of self-reported CPD either [3]. These results strongly suggest that, in addition to a large percentage of participants failing to report their non-study cigarette use (i.e., the rate of non-compliance), they also underreported the numbers of non-study cigarettes smoked (i.e., the magnitude of non-compliance). In another study among vulnerable populations who smoke, 82–83% of the participants were estimated to be non-compliant based on urinary cotinine [18% and 17% of participants fully adherent at weeks 6 and 12, respectively] [5], compared to 55–60% based on self-report ([5]; Table S1). Both studies set the compliance biomarker cutoff conservatively at fourfold higher than expected nicotine intake from the reduced nicotine cigarettes to accommodate for potential compensation (i.e., more intense puffing and inhalation) or other sources of variability. As acknowledged by the authors, available research on switching from CNC to RNC shows minimal evidence of extended compensatory smoking [18], indicating that 400% compensatory smoking was unlikely. Consequently, the actual non-compliance rates are likely even higher than those estimated biochemically using this threshold.

In addition to the rate, the magnitude of non-compliance in non-study cigarette use (i.e., the number of cigarettes) can also impact the study outcomes of the trials. While the rate of non-compliance has been assessed biochemically in the studies discussed above, the magnitude of non-compliance was not addressed. In an earlier publication [19], we developed a method, using urinary biomarkers of exposure to nicotine and NNK, to biochemically estimate the magnitude of non-study cigarette use in ambulatory RNC studies. Similar to the rate results, study participants assigned RNC consistently underreported the magnitude of their non-study cigarette use by 73–89% across study groups using different RNC [19]. A major limitation of that analysis is the use of aggregate group average CPD and biomarker values for each study group from the respective publications to estimate the magnitude of non-compliance, because raw data on individual participants were not available [19].

Raw study data from one of the largest RNC switching studies [3] have since become publicly available through NIDA’s Data Share Website [20], which enables this secondary analysis to biochemically assess the magnitude of non-compliance using raw data from individual study participants. The objective of this secondary analysis is to estimate the average numbers of non-study CPD and total CPD for each group assigned to RNC, with the group assigned CNC as the reference, based on individual values for self-reported study and non-study CPD numbers, and urinary total nicotine equivalents (TNE) from [3], using the previously published method [19].

## Methods

### Data source

This is a secondary analysis of data from a large, randomized trial of RNC among a general population who regularly smoke combustible cigarettes [3], from NIDA’s Data Share Website [20]. In this study, 840 healthy participants who currently smoked and were not intending to quit in the next 30 days were randomly assigned to smoke for 6 weeks their usual brand cigarettes or one of six investigational SPECTRUM® cigarettes with nominal nicotine content of 15.8, 5.2, 2.4, 1.3 and 0.4 mg per gram tobacco filler (mg/g) including a 0.4 mg/g variant at high tar yield. The primary outcome measure of the study was the average number of participants’ self-reported study, non-study and total CPD during week 6. First void urine (or a spot urine for participants who forgot to obtain the first voiding sample) collected at randomization, week 2 and week 6 was used to measure biomarkers of exposure to tobacco smoke including total nicotine equivalents (TNE, as a measure of nicotine exposure) and total 4-(methylnitrosamino)-1-(3-pyridyl)-1-butanol (NNAL, a biomarker of NNK exposure).

### Non-study CPD estimation

The estimated number of non-study cigarettes for each study participant was calculated based on the nicotine content of the RNC assigned, the number of self-reported study CPD and the levels of biomarkers of exposure (BOE) to nicotine (TNE) from Donny et al. [3] using a previously published method [19]. This method has been shown to accurately estimate (within 1–3%) non-study CPD numbers with data from a confined study [7] during which the use of tobacco products were fully controlled and accounted for in the study data. The method generated consistent non-study CPD estimates using different tobacco constituents (nicotine, NNK and anatabine) and their corresponding urinary BOEs (nicotine, cotinine and TNE for nicotine; NNAL for NNK; and anatabine for anatabine) [19]. Nicotine content in both tobacco filler and cigarette smoke of the study cigarette assigned (Table 1) and the individual’s TNE values for weeks 2 and 6 from Donny et al. [3] were used for non-study CPD estimation in this analysis.

**Table 1:** Average nicotine yields of study cigarettes (mg/cigarette)*

Cigarette (NIDA Product Codes)	15.8 (NRC600/1)	5.2 (NRC400/1)	2.4 (NRC300/1)	1.3 (NRC200/1)	0.4 (NRC102/3)	0.4 high tar (NRC104/5)
Tobacco Filler	11.16	3.18	1.27	0.63	0.18	0.21
Smoke (ISO)	0.7	0.235	0.105	0.06	0.02	0.04
Smoke (Canadian Intense)	1.48	0.5	0.215	0.115	0.04	0.07

^*^Average for menthol and non-menthol cigarettes at the same nicotine level (mg/cigarette). Nicotine yield in tobacco filler was derived by multiplying mg/g nicotine by filler weight data from [21]; smoke nicotine yield data from [22]

Briefly, if a participant exclusively used the assigned study cigarettes, the level of his/her urinary TNE was expected to be proportional to the amount of nicotine per cigarette ("NIC" for simplicity) and CPD:

1 $${\text{TNE}}={\text{NIC}} \times {\text{CPD}} \times k$$

where k represents a composite factor comprising how a cigarette was smoked by the participant and the absorption, distribution and clearance of nicotine. The *k* value for the control CNC group can be derived using:

2 $${K}_{\text{cnc}}=\frac{{{\text{TNE}}}_{{\text{cnc}}}}{{{\text{NIC}}}_{{\text{cnc}}}\times {{\text{CPD}}}_{{\text{cnc}}}}$$

where TNE_CNC_ is the group mean TNE, CPD_CNC_ is the group mean CPD and NIC_CNC_ is the nicotine content of the SPECTRUM® CNC cigarette.

When a study participant smoked non-study cigarettes in addition to the assigned study cigarettes, the TNE level would reflect the combined contributions from both sources:

3 $${\text{TNE}}=\;{{\text{NIC}}}_{{\text{study}}}\times {{\text{CPD}}}_{\mathrm{study }}\times { k}_{{\text{study}}}+{{\text{NIC}}}_{{\text{non}}-{\text{study}}}\times {{\text{CPD}}}_{{\text{non}}-{\text{study}}} \times {k}_{{\text{non}}-{\text{study}}}$$

Using k derived from the CNC group for both study and non-study cigarettes and using NIC_CNC_ as a surrogate for NIC_non-study_ [see [19] for a detailed discussion]:

4 $${\text{TNE}}=\;{{\text{NIC}}}_{{\text{study}}}\times {{\text{CPD}}}_{\mathrm{study }}\times { k}_{{\text{cnc}}}+ {{\text{NIC}}}_{{\text{cnc}}}\times {{\text{CPD}}}_{{\text{non}}-{\text{study}}}\times {k}_{{\text{cnc}}}$$

Using the individual TNE, NIC and CPD data at weeks 2 and 6 for both the CNC (reference) and RNC groups in [3], the corresponding non-study CPD number for each participant in an RNC group was calculated as:

5 $${{\text{CPD}}}_{\text{non-study}}=\frac{{\mathrm{TNE }-{\mathrm{ NIC}}_{{\text{study}}}\times {{\text{CPD}}}_{\mathrm{study }}\times { k}_{{\text{cnc}}}}}{{{\text{NIC}}}_{{\text{cnc}}}\times {k}_{{\text{cnc}}}}$$

A participant’s underreported non-study CPD is the difference between the estimated CPD_non-study_ and self-reported non-study CPD in [3].

A participant’s estimated total CPD is the sum of self-reported study CPD and estimated non-study CPD.

6 $${{\text{CPD}}}_{{\text{total}}}= {{\text{CPD}}}_{{\text{study}}}+ {{\text{CPD}}}_{{\text{non}}-{\text{study}}}$$

### Statistical analysis

Individual participant’s estimated non-study CPD at weeks 2 and 6 was first calculated based on the urinary TNE levels, nicotine content of the assigned cigarette and their self-reported study CPD, with negative estimated non-study CPD set to zero. Individual participants’ estimated total CPD was derived as described in the previous section.

Estimated non-study CPD and estimated total CPD, together with the original total CPD reported in Donny et al. (2015), were summarized by study group and time point. The estimated non-study and total CPD at week 6 are the primary endpoints of this analysis.

Wilcoxon matched-pairs signed-rank test was conducted to test the difference between the participants’ self-reported non-study CPD and estimated non-study CPD within each of the RNC groups by visit due to data not normally distributed (Additional file 1).

The RNC groups' estimated total CPD at weeks 2 and 6 were compared with the control CNC group’s self-reported total CPD using a linear mixed model. The following model terms were included in the model: group, visit, group-by-visit interaction and individual participant’s CPD at baseline (Additional file 2). A Dunnett–Hsu method was used for the multiple comparisons.

## Results

### Non-study CPD estimates

**Fig. 1:**
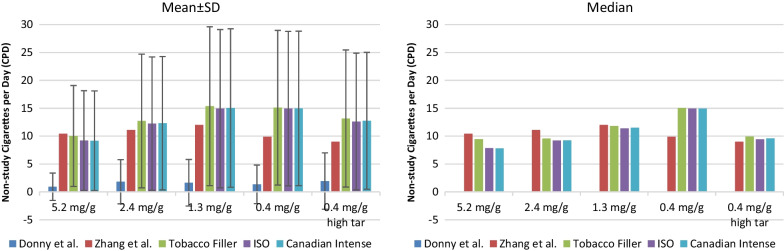
Mean and median non-study CPD at week 6. Mean and median non-study CPD at week 6 for groups assigned RNC including participants’ self-report from Donny et al. [3], estimates based on group averages from Zhang et al. [19], estimates from this analysis based on nicotine content of the assigned cigarettes in tobacco filler, smoke nicotine yield under ISO and Canadian Intense smoking conditions, respectively. All estimated mean non-study CPD numbers from this analysis are significantly higher than those self-reported in Donny et al. [3] (*p* < 0.0001, Wilcoxon matched-pairs signed-rank test). Median self-reported non-study CPD numbers in Donny et al. [3] were zero for all groups

Estimates of non-study CPD using nicotine content based on tobacco filler, ISO and Canadian Intense machine smoking conditions (Table 1), respectively, produced comparable mean and median results during week 6 for each of the five RNC groups with the CNC group as the reference (Fig. 1). All estimated mean non-study CPD numbers from this analysis are significantly higher (Wilcoxon matched-pairs signed-rank test, *p* < 0.0001) than those self-reported by study participants for all the RNC groups [3]. Overall, both the mean and median non-study CPD estimates based on the raw data from this analysis are comparable with or higher than the prior point estimates based on group average values [19], with the mean estimates using the raw data trending higher for the three groups assigned to RNC at nicotine level of 1.3 mg/g and below but similar for the 5.2 and 2.4 mg/g groups. The median non-study cigarette CPD estimates based on the raw data are higher than previous estimates in “Zhang et al.” [19] for the 0.4 mg/g group only and are generally comparable for the other RNC groups. For groups assigned to RNC at the regular tar level (i.e., not high tar), both the mean and median non-study CPD numbers trend higher as the nicotine content in the assigned cigarettes decreases. Results from the group assigned 0.4 mg/g high tar cigarette does not follow this trend. This group was “identified a priori as exploratory” in the original study design [3], because results from this group are confounded by the difference in tar level, which can influence participants’ smoking behavior.

Since estimated non-study CPD based on the three nicotine content measures (i.e., tobacco filler, ISO and Canadian Intense machine smoking conditions) were comparable (Fig. 1), only results based on nicotine content in the tobacco filler are discussed in the sections below.

### Underreporting of non-study CPD

Table 2 presents the magnitude of underreporting of non-study CPD by study participants comparing the mean self-reported non-study CPD numbers from Donny et al. [3] with the mean biochemically estimated non-study CPD from this analysis at weeks 2 and 6. Study participants in all the RNC groups substantially underreported their non-study CPD at both week 2 (by 82–88%) and week 6 (by 85–91%). These underreporting estimates based on the raw data are similar to (or higher than) the 73–89% based on group averages reported in Zhang et al. [19]. Similar to the large variation in self-reported CPD, there is a large variation in the estimated number of non-study cigarettes smoked by participants within each group, with the variation being higher at week 2 (coefficient of variation, CV% 103–232%) than week 6 (CV% 90–94%).

**Table 2:** Underreporting of non-study cigarette use by study group at weeks 2 and 6

	Investigational Cigarettes (mg/g nominal nicotine content)					
Group	15.8	5.2	2.3	1.3	0.4	0.4 high tar
*Week 2*						
Self-reported mean non-study CPD (SD)	0.51 (1.586)	1.26 (2.508)	1.94 (3.876)	2.22 (4.601)	2.01 (3.895)	2.05 (4.340)
Estimated mean non-study CPD (SD)	NA	10.85 (25.211)	10.71 (11.073)	13.51 (14.255)	16.51 (21.986)	11.98 (13.471)
Underreported non-study CPD	–	9.59	8.77	11.29	14.50	9.93
Underreporting (%)	–	88	81.9	83.6	87.8	82.9
*Week 6*						
Self-reported mean non-study CPD (SD)	0.41 (1.198)	0.92 (2.466)	1.81 (3.967)	1.63 (4.164)	1.35 (3.454)	1.94 (5.046)
Estimated mean non-study CPD (SD)	NA	10.04 (9.053)	12.72 (12.005)	15.36 (14.247)	15.10 (13.874)	13.15 (12.294)
Underreported non-study CPD		9.12	10.91	13.73	13.75	11.21
Underreporting (%)	–	90.8	85.8	89.4	91.1	85.2

NA, not applicable, the 15.8 mg/g CNC group was used as reference

### Total CPD estimates

**Fig. 2:**
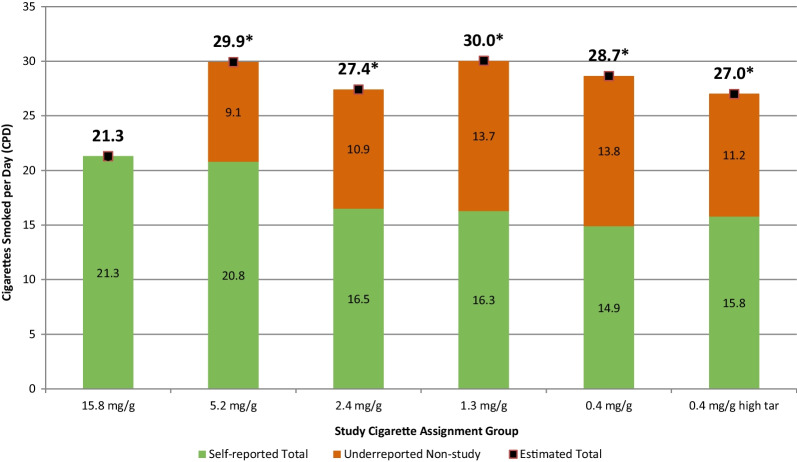
Mean self-reported and estimated total CPD during week 6. Self-reported Total CPD from Donny et al. [3]; underreported non-study CPD = biochemically estimated minus self-reported non-study CPD from Table 2; Mean estimated total CPD = mean total self-reported CPD plus mean underreported non-study CPD. *significantly higher total CPD than the control 15.8 mg/g group (*p* < 0.05)

Figure 2 presents a composite picture of the mean CPD results at week 6. Based on self-reported data alone, participants in the groups assigned RNC with 2.4, 1.3 or 0.4 mg/g nicotine had significantly lower total CPD during week 6 of the study, as reported in Donny et al. [3]. However, when the biochemically estimated underreported non-study CPD numbers from Table 2 are taken into consideration, there is no reduction in the total CPD for any of the RNC groups compared to the CNC group. On the contrary, all RNC groups had significantly higher total CPD than the control CNC group (*p*-values 0.0001, 0.0037, < 0.0001, < 0.0001, 0.0464) for RNC Groups 5.2, 2.3, 1.3, 0.4 and 0.4 mg/g high tar, respectively, compared to the 15.8 mg/g control group (Fig. 2).

**Fig. 3:**
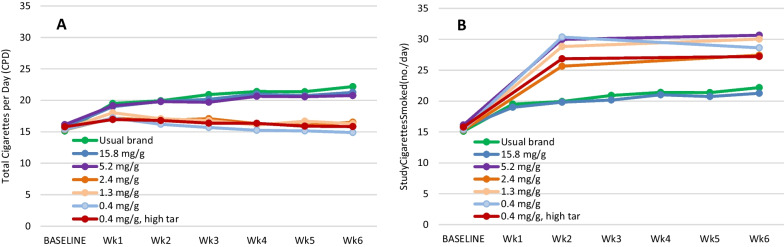
Mean self-reported and estimated total CPD. Mean self-reported total CPD are from Donny et. al. [3] (**A**). Mean estimated total CPD for weeks 2 and 6 (**B**), based on estimated total CPD for each individual participants, i.e., the sums of the self-reported total CPD from Donny et. al. [3] and the underreported non-study CPD estimated in this study. CPD numbers for the usual brand and the 15.8 mg/g control group in 3B are from Donny et. al. [3]. Total CPD numbers for all RNC groups are significantly higher than the control 15.8 mg/g group at both weeks 2 and 6 (*p* < 0.05)

Figure 3 compares the total CPD results based on self-report only during weeks 1 to 6 as reported in [6] (3A) with total CPD estimates (3B) for weeks 2 and 6 from this analysis. Consistent with Fig. 2, when underreported non-study CPD is taken into consideration, there is no reduction in total CPD for any of the groups assigned to RNC during either week 2 or 6 (3B). All RNC groups had significantly higher mean total CPD than the control CNC group during both weeks 2 and 6 (p-values for RNC Group 5.2, 2.3, 1.3, 0.4 and 0.4 mg/g high tar are 0.0009, 0.0333, 0.0005, < 0.0001, 0.0212 at week 2, and 0.0001, 0.0037, < 0.0001, < 0.0001, 0.0464 at week 6, respectively).

## Discussion

Clinical studies switching adults who smoke to RNC are critical for a science-based product standard establishing a maximum nicotine level in combustible cigarettes. Except for a few small studies, most clinical trials on RNC were of ambulatory design during which study participants had ready access to non-study CNC. Understanding the degree of non-compliance in such trials is important for the proper interpretation of research outcomes to support science-based regulatory decision-making.

High percentages of study participants self-reported using non-study cigarettes across all ambulatory RNC studies [3–6]. In addition, the percentage of participants who actually used non-study CNC during the studies are likely even higher than self-report because a large proportion of study participants did not report their non-study cigarette use, as indicated by the substantially higher biochemically estimated non-compliance rates compared to those self-reported by the study participants [5, 6, 23]. High rates of non-study cigarette use have been observed even when nicotine-containing e-cigarettes were provided concurrently during the study [23].

The high magnitude (i.e., in CPD numbers) of non-compliance estimated from this secondary analysis using raw data concurs with an earlier analysis using the group average data from one of the largest RNC clinical trials [19]. Study participants underreported their mean non-study cigarette CPD by 85–91% in week 6 (81–88% in week 2). During both weeks 2 and 6, after accounting for the underreported non-study CPD, switching to RNC not only did not reduce but rather increased the total number of cigarettes smoked per day by the participants for every RNC group, which is different from the conclusion based on self-report alone [3].

Such high levels of conventional nicotine non-study cigarette use, in both the rate and magnitude, not only will impact the interpretation of the self-reported CPD data but also will likely complicate the assessment of the impact of switching to RNC on other study measures including nicotine dependence and smoking abstinence. Factors other than nicotine content can also affect responses to RNCs, further complicating the generalization of results from a limited set of research cigarettes in the context of a nicotine standard. In addition to lower nicotine levels, sensory dissatisfaction with the SPECTRUM® research cigarettes commonly used in the recent RNC clinical trials has been identified as likely to be a driver for the high non-compliance [6]. Results from this analysis also show that, among participants assigned RNC at 0.4 mg/g nicotine level, those assigned the high tar variety smoked less non-study cigarettes than those assigned standard tar cigarettes, indicating an RNC’s tar level can affect the response of adults who smoke. Since this is the only clinical trial that examined the impact of RNC’s tar level on switching behavior, additional studies are warranted. An RNC clinical trial including arms in which participants were provided NRT and alternative non-combustible nicotine products including e-cigarettes in different flavors (vs tobacco flavor only) reported lower non-study cigarette use among the RNC group when e-liquids at a higher nicotine level with different flavors were provided [23]. This suggests the availability of alternative tobacco products with or without flavors can also affect the response of adults who smoke cigarettes under a potential nicotine standard.

Study participants’ strong willingness to spend their own money to buy non-study cigarettes as reflected in the high non-compliance rates and high non-study CPD numbers, when the reduced nicotine study cigarettes (plus e-cigarettes and/or NRT in some studies) were provided to them free of charge during the study, suggests that people who regularly smoke will likely seek alternative sources of nicotine including NRT, smoke-free tobacco products and illicit cigarettes with conventional nicotine levels when a low nicotine product standard is implemented. This drive to seek alternative nicotine sources might be stronger among certain vulnerable populations. In studies with both the general and vulnerable populations who smoke, greater cigarette dependence severity, lower satisfaction with study cigarette and younger age have been reported to be associated with non-compliance [5, 6]. Vulnerable participants who used opioids also exhibited reduced adherence [5]. Mitigating measures that can ease the transition of vulnerable populations away from conventional cigarettes should be considered as an integral part of any plan to implement a low nicotine product standard for combusted cigarettes to reduce unintended consequences.

This analysis has several strengths. Compared to the prior analysis using summary group average values [19], raw data for individual participants were used which permitted the application of standard statistical tools rather than point estimates only. The results from this analysis are consistent with previous results based on group summaries. The higher mean total CPD numbers from this analysis for the group assigned 0.4 mg/g nicotine cigarette at weeks 2 and 6 (30.4 and 28.6, respectively), after accounting for underreported non-study CPD, are also in line with the 28.3 CPD (20.1 at baseline) from a confined clinical trial where the same 0.4 mg/g nicotine RNC was used under confined conditions and underreporting of CPD was not possible [7]. In two confined RNC studies [7, 8], TNE levels decreased proportionally in the RNC group, compared to the CNC group, as expected from the difference in the nicotine contents between the cigarettes. In contrast, the reductions in TNE in all ambulatory studies are substantially less than expected from the differences in the nicotine content of the study cigarettes [3, 4, 9–13], which provides strong support for the high estimated non-study CPD numbers in this secondary analysis.

One limitation of this analysis is applying the k values derived from CNC to RNC groups, with the assumption that there was no substantial compensatory smoking with RNC. Studies that specifically examined compensatory smoking during both ambulatory and confined trials with RNC did not report evidence of substantial compensation [3, 8, 24–27]. A related limitation is using group average k values to estimate non-study CPD. The k value for each constituent/biomarker combination represents a composite factor that is not only influenced by the exposure to the constituent but also by individual differences in the metabolism, distribution and clearance of the constituent involved which can be affected by factors such as age, sex and genetics [28–30]. While the impact of inter-individual pharmacokinetic variations on the k value is partially mitigated in this analysis as comparisons were made between large groups of randomized participants, caution is warranted when such comparisons are made between small or non-randomized groups. In addition, while the use of urinary TNE mitigated individual differences in nicotine and cotinine metabolism [31], the potential impact of inter-individual differences in nicotine metabolism should be considered when urinary nicotine or cotinine alone is used. Using group average k values to estimated individual non-study CPD resulted in negative estimated non-study CPD values for 4.7% (25 out of 535) participants at week 6, which were set to zero during the analysis. As discussed earlier, the validity of the method using group average k values derived from the CNC group was confirmed with data from a confined study using the same RNC during which all cigarettes smoked by participants were accounted for [19]. Another limitation of the study is using participants’ assigned SPECTRUM® CNC as the reference group with the assumption of no underreporting of non-study cigarettes use in this group. Using study participants’ usual brand cigarettes as a reference, Zhang et al. [19] estimated that the CNC group in Donny et al. [3] underreported non-study cigarette use by 1.8 CPD. The underreported non-study CPD estimates using participants’ usual brand group as reference were about 10% higher across all RNC groups than those using the CNC group as the reference [19]. Therefore, results from this analysis using the CNC group as the reference likely underestimated participants’ actual non-study cigarette CPD by about 10%.

## Conclusions

This secondary analysis shows that (1) self-reported non-study CPD numbers may not be reliable in ambulatory RNC studies where participants continue to have ready access to their usual brand cigarettes; (2) in addition to substantially underreporting the rate of their non-study cigarette use which has been consistently reported in the literature, participants in all RNC groups underreported the number of their non-study CPD during the study by a factor of almost ten (85–91%); and (3) after accounting for the underreported non-study CPD, there appears to be no reduction in total CPD in any of the RNC groups, which is contrary to the conclusion based on self-reported CPD numbers alone [3].

Due to consistently high rates and magnitudes of non-study cigarette use across studies, results from ambulatory RNC trials should be interpreted considering this limitation. Future ambulatory RNC switching studies may benefit by incorporating assessments, such as biomarkers, that would enable the quantification of both the prevalence and the magnitude of non-study cigarette use.

### Supplementary Information

**Additional file 1:** Wilcoxon matched-pairs signed-rank test output.

**Additional file 2:** Statistical analysis SAS codes.

## Data Availability

The datasets analyzed during the current study are available in NIDA’s Data Share Website: https://datashare.nida.nih.gov/study/nidacenicp1s1.

## Abbreviations

BOE: Biomarker of exposure
CI: Canadian Intense
CNC: Conventional nicotine cigarette
CPD: Cigarettes per day
FDA: Food and Drug Administration
ISO: International Organization for Standardization
NIC: Nicotine
NNAL: 4-(Methylnitrosamino)-1-(3-pyridyl)-1-butanol
NNK: 4-(Methylnitrosamino)-1-(3-pyridyl)-1-butanone
NRT: Nicotine replacement therapy
RNC: Reduced nicotine cigarette
TNE: Total nicotine equivalent
