# Supplementary material for: Underreporting of non-study cigarette use by study participants confounds the interpretation of results from ambulatory clinical trial of reduced nicotine cigarettes
Source: Harm Reduct J. 2024 Feb 8;21:35. doi: 10.1186/s12954-024-00953-8 (PMC10854148; doi:10.1186/s12954-024-00953-8)
Supplement: Supplementary file 1 — Additional file 1: Wilcoxon matched-pairs signed-rank test output. [file 12954_2024_953_MOESM1_ESM.pdf]

Supplemental File I – Wilcoxon Matched-Pairs Signed-rank Test SAS output

Paired t-test on raw self-reported and estimated non-study CPD

The TTEST Procedure

Difference: ADJ_CAL_NONSTUD_CPD_TF - NON_STUDY_CPD
AVISIT=week 2 TRTA=A ARM=2.4 mg/g

N	Mean	Std Dev	Std Err	Minimum	Maximum
110	8.8462	9.4409	0.9002	-2.8333	49.9986

Mean	95% CL Mean		Std Dev	95% CL Std Dev	
8.8462	7.0621	10.6303	9.4409	8.3368	10.8848

DF	t Value	Pr > |t|
109	9.83	<.0001

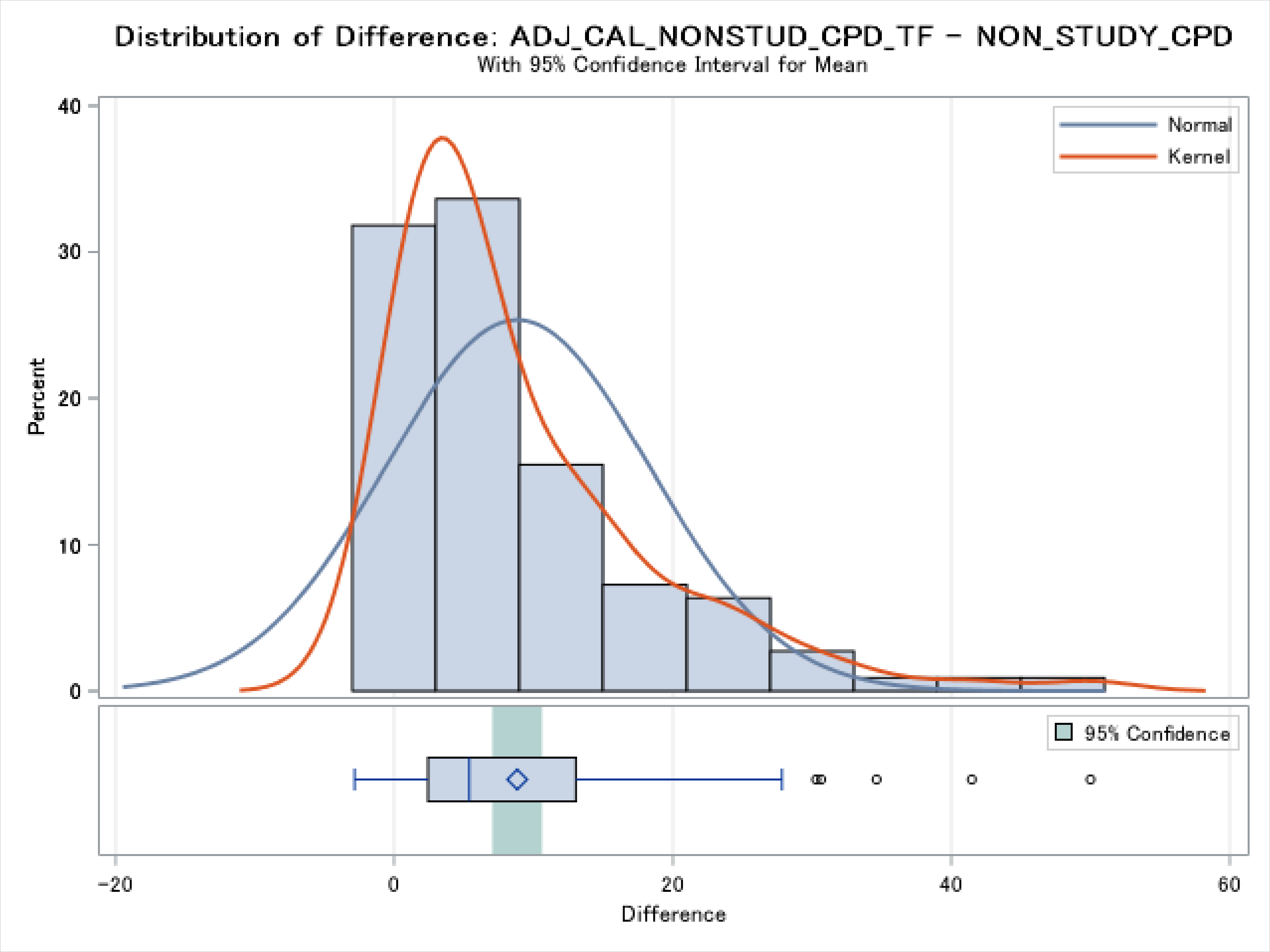

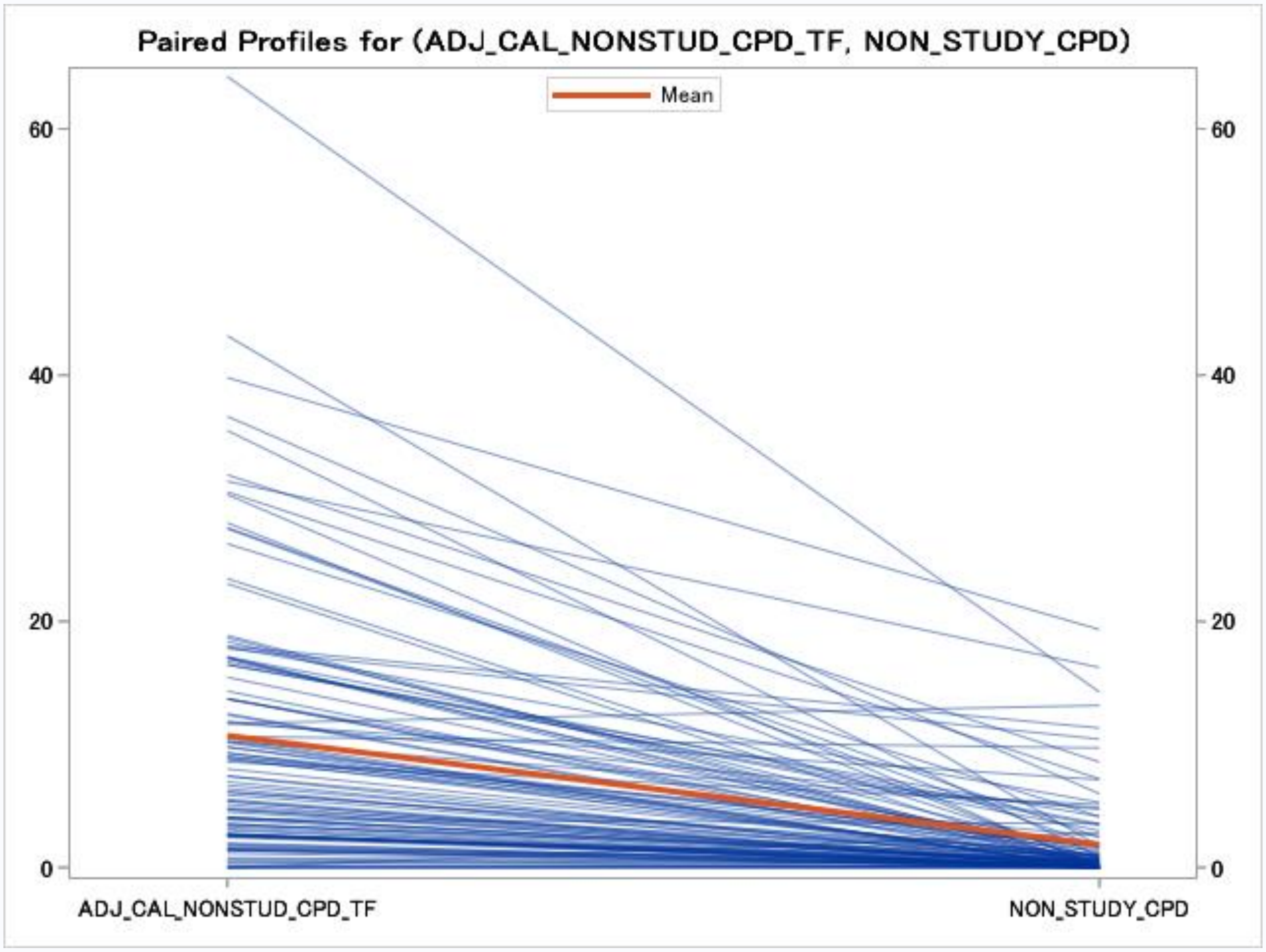
Paired Profiles for (ADJ_CAL_NONSTUD_CPD_TF, NON_STUDY_CPD)
Mean
60
40
20
0
ADJ_CAL_NONSTUD_CPD_TF
NON_STUDY_CPD

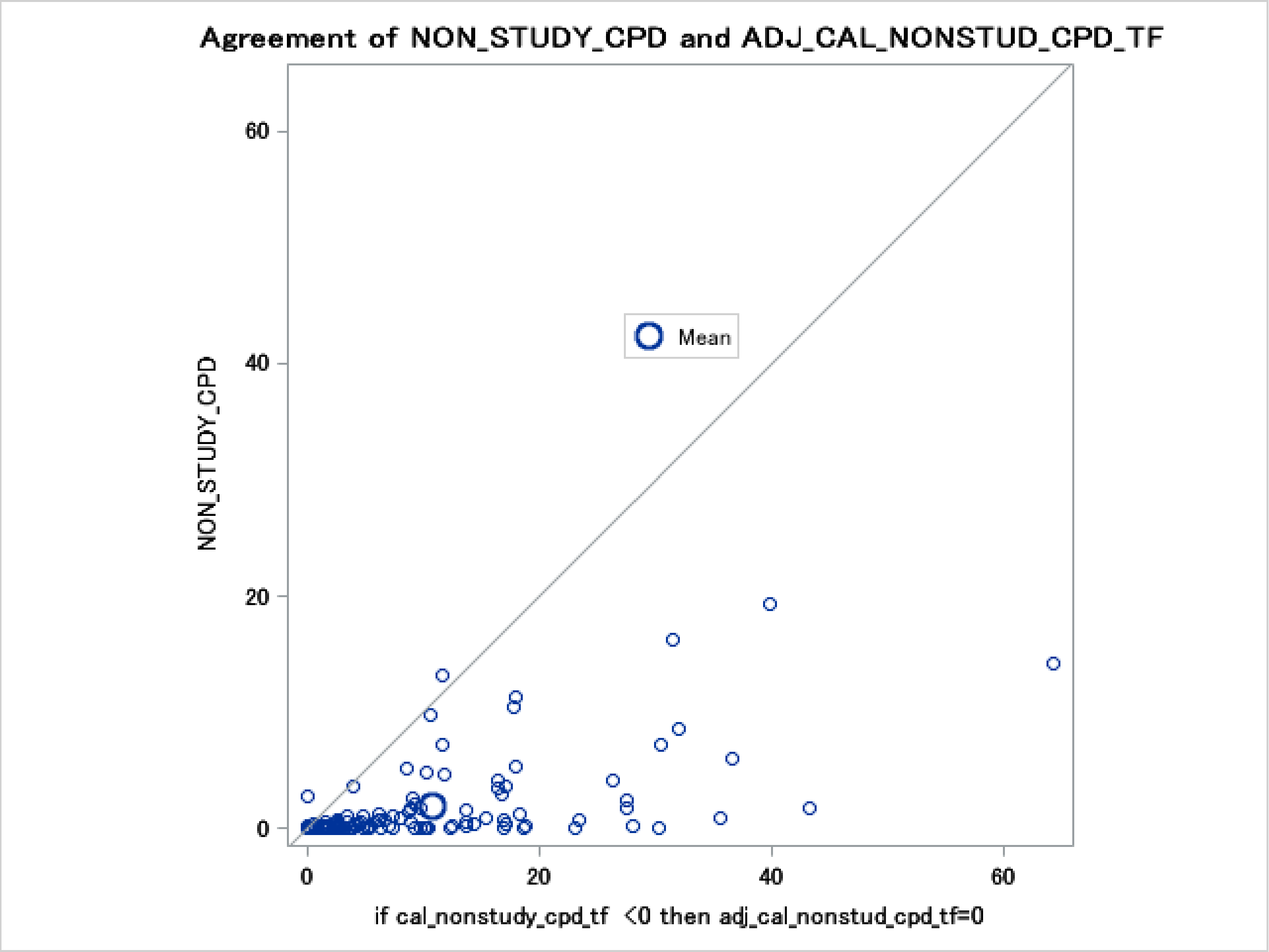
Agreement of NON_STUDY_CPD and ADJ_CAL_NONSTUD_CPD_TF
Mean
NON_STUDY_CPD
60
40
20
0
0
20
40
60
if cal_nonstudy_cpd_tf <0 then adj_cal_nonstud_cpd_tf=0

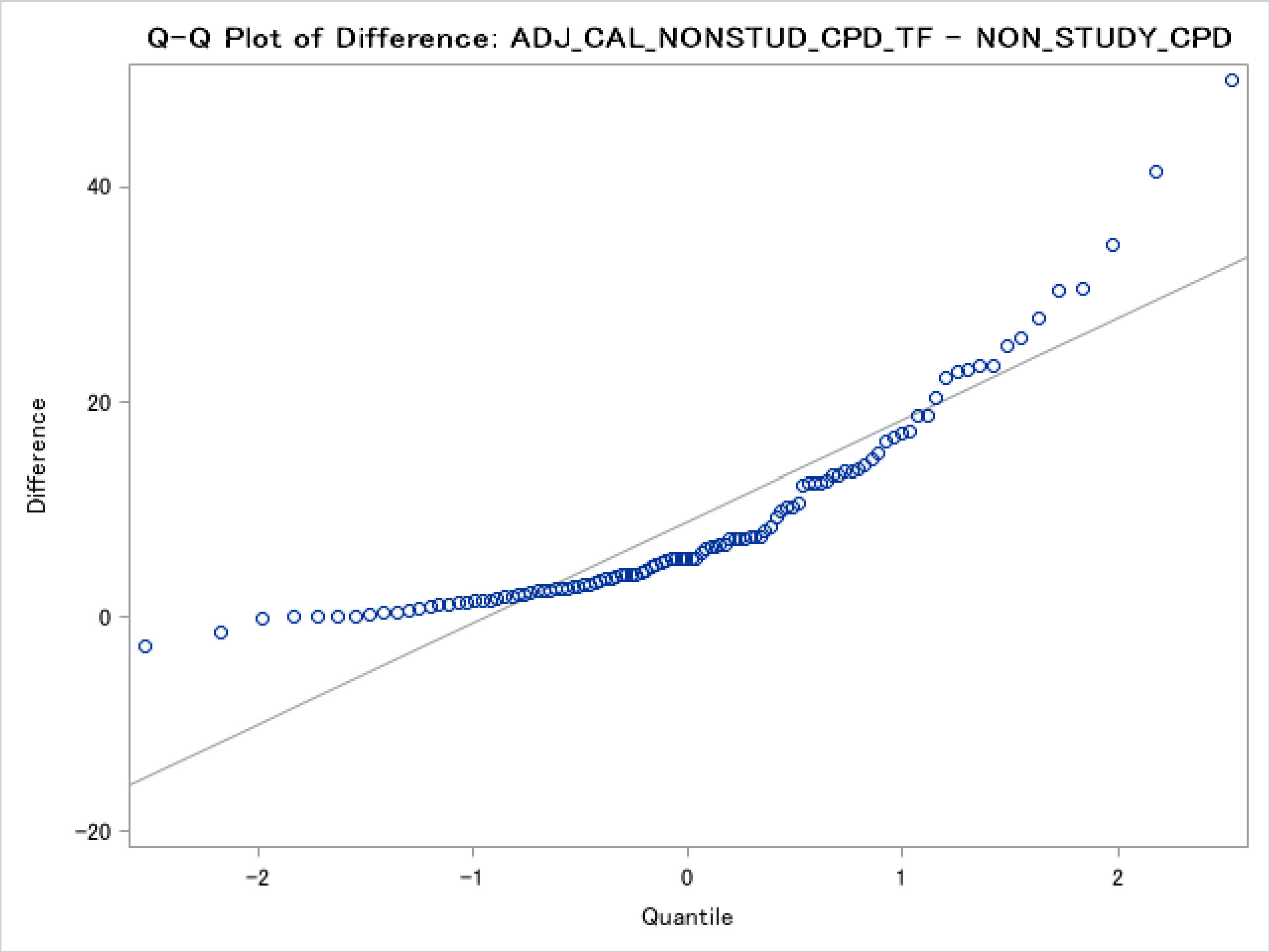
Q-Q Plot of Difference: ADJ_CAL_NONSTUD_CPD_TF - NON_STUDY_CPD
Difference
40
20
0
−20
−2
−1
0
1
2
Quantile

Paired t-test on raw self-reported and estimated non-study CPD

The TTEST Procedure

Difference: ADJ_CAL_NONSTUD_CPD_TF - NON_STUDY_CPD
AVISIT=week 2 TRTA=B ARM=0.4 mg/g (HT)

N	Mean	Std Dev	Std Err	Minimum	Maximum
115	9.9769	12.7414	1.1881	-5.5536	82.3765

Mean	95% CL Mean		Std Dev	95% CL Std Dev	
9.9769	7.6232	12.3306	12.7414	11.2804	14.6405

DF	t Value	Pr > |t|
114	8.40	<.0001

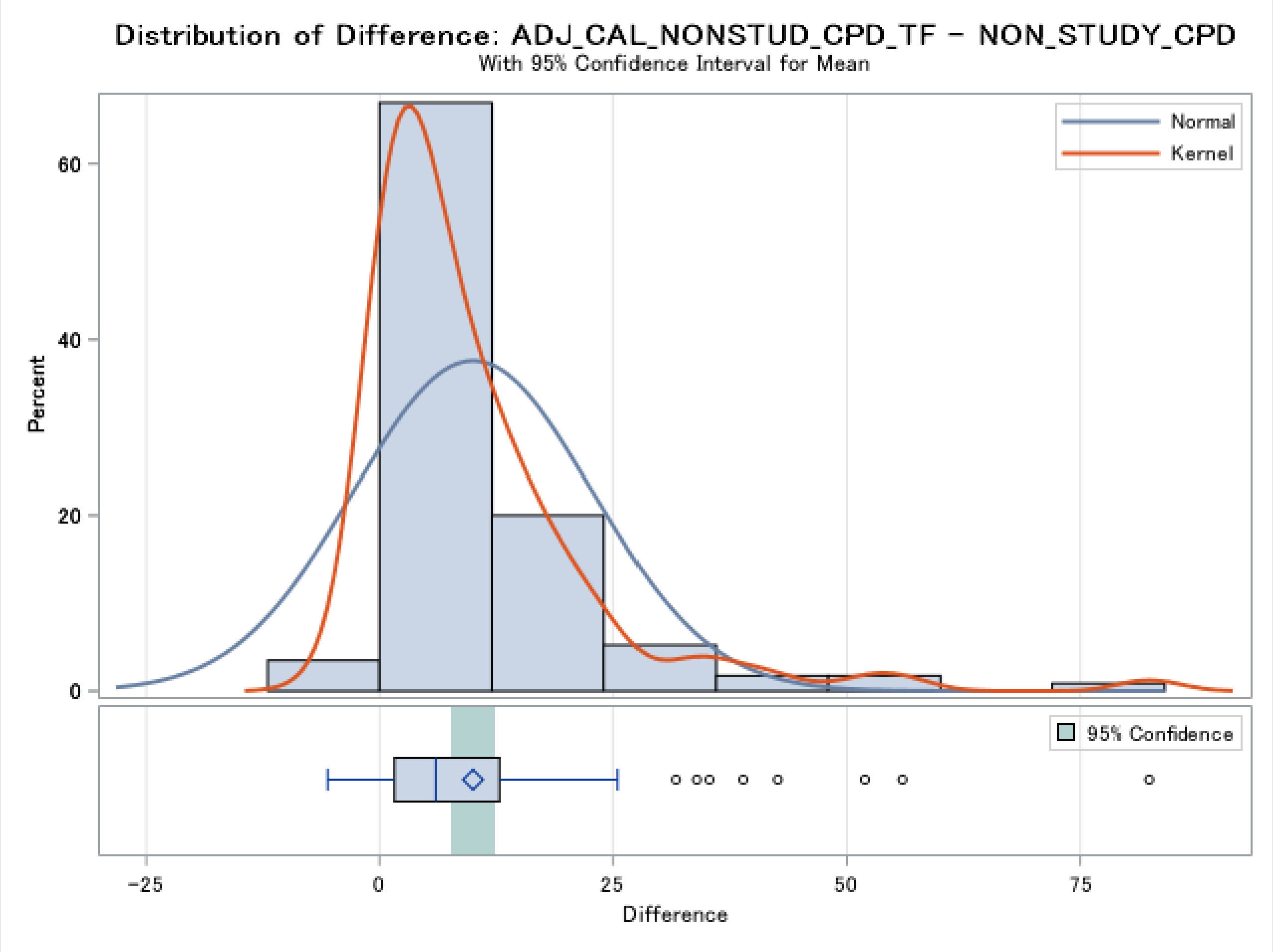

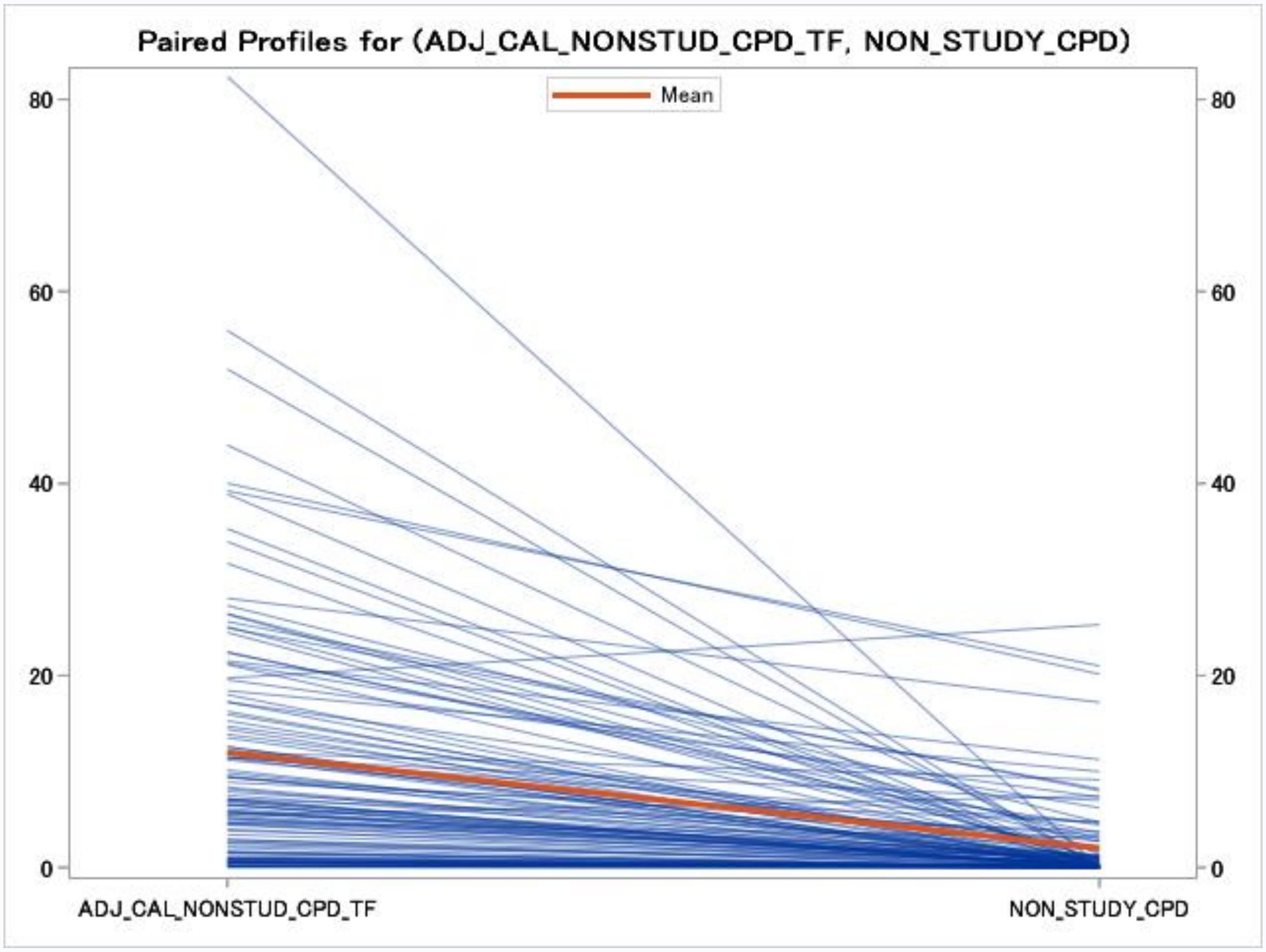
Paired Profiles for (ADJ_CAL_NONSTUD_CPD_TF, NON_STUDY_CPD)
Mean
80
60
40
20
0
ADJ_CAL_NONSTUD_CPD_TF
NON_STUDY_CPD

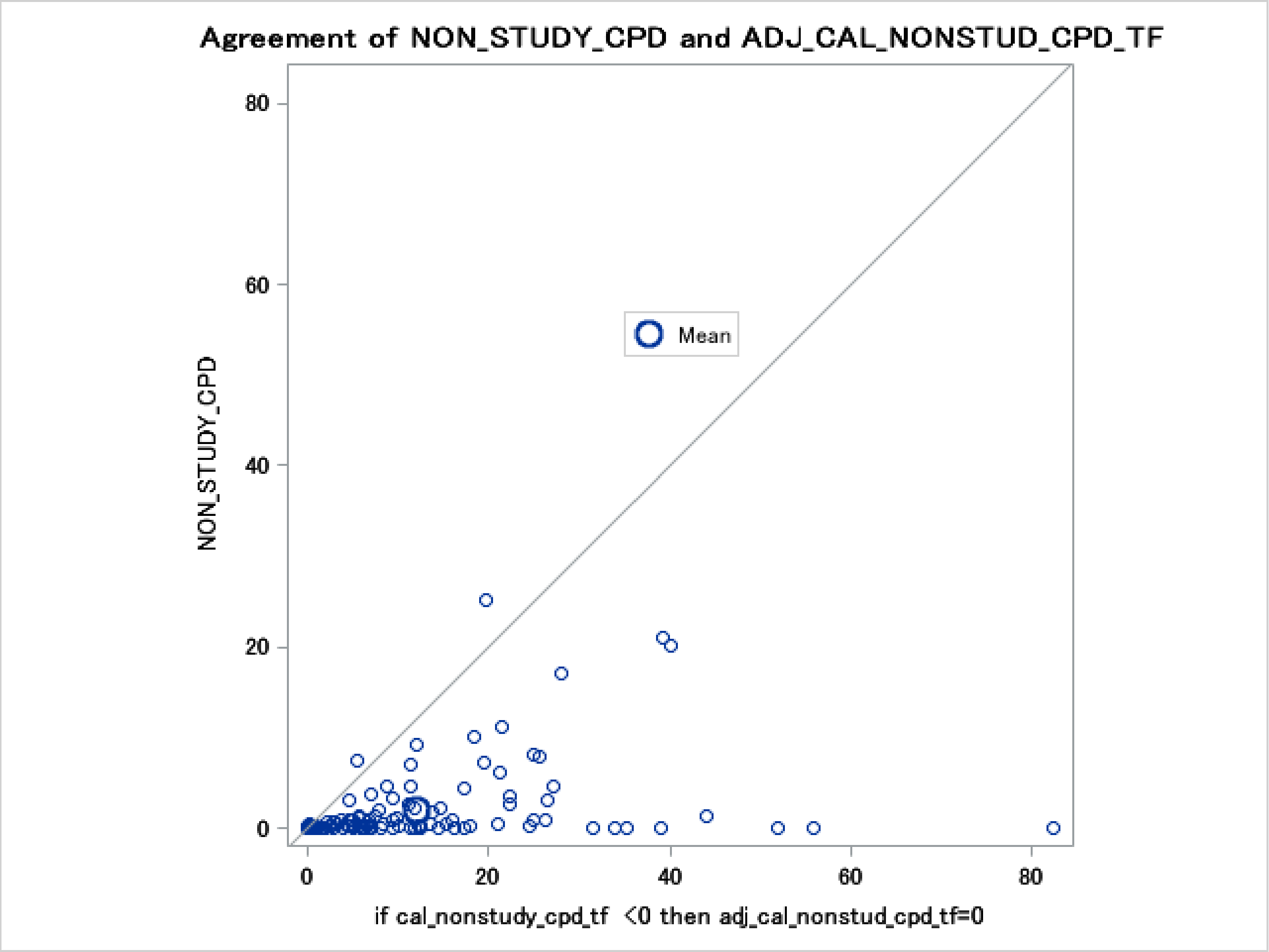
Agreement of NON_STUDY_CPD and ADJ_CAL_NONSTUD_CPD_TF
NON_STUDY_CPD
80
60
40
20
0
Mean
0
20
40
60
80
if cal_nonstudy_cpd_tf <0 then adj_cal_nonstud_cpd_tf=0

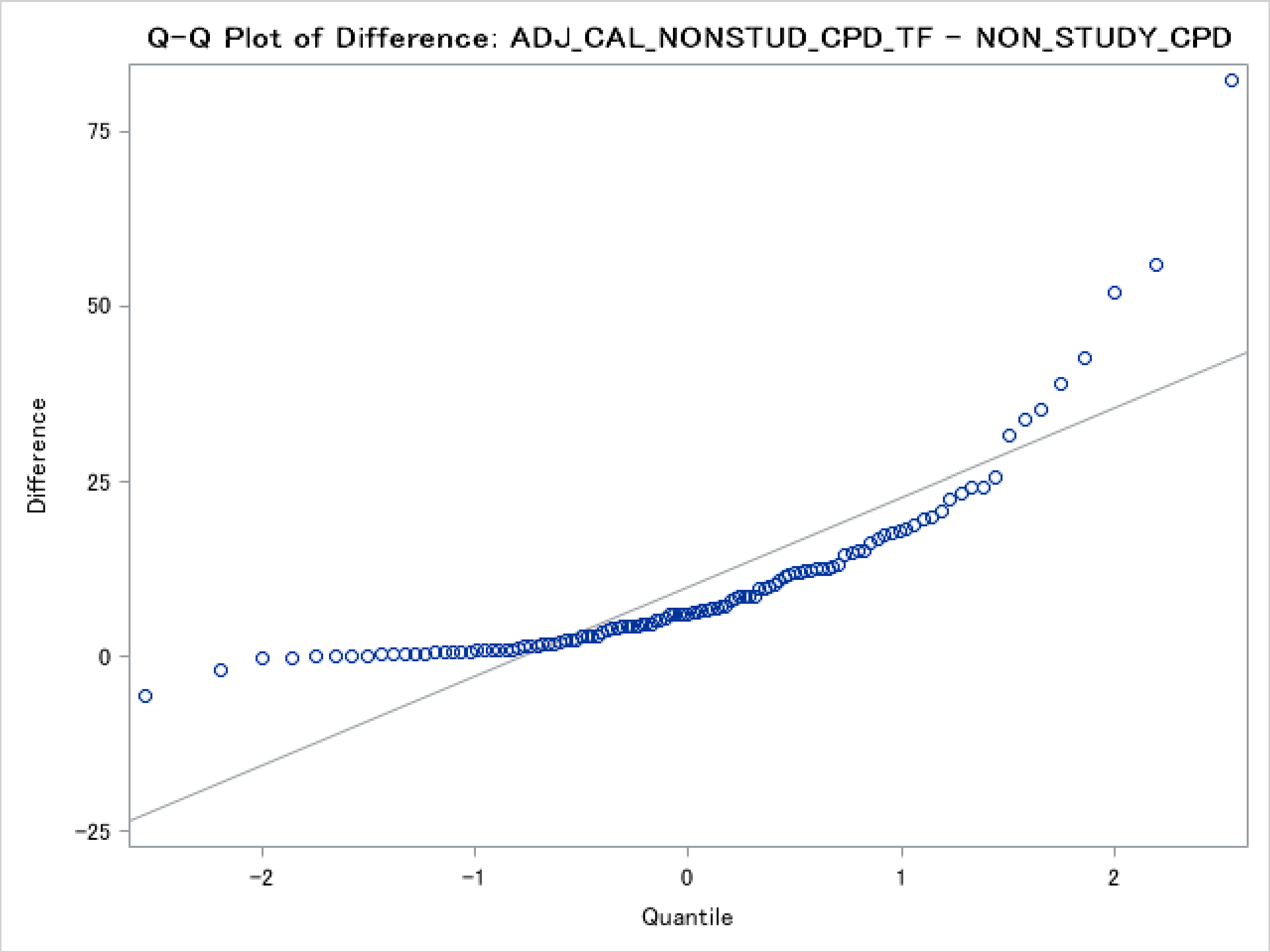

Q-Q Plot of Difference: ADJ_CAL_NONSTUD_CPD_TF - NON_STUDY_CPD
Difference
75
50
25
0
-25
-2
-1
0
1
2
Quantile

Paired t-test on raw self-reported and estimated non-study CPD

The TTEST Procedure

Difference: ADJ_CAL_NONSTUD_CPD_TF - NON_STUDY_CPD
AVISIT=week 2 TRTA=D ARM=1.3 mg/g

N	Mean	Std Dev	Std Err	Minimum	Maximum
110	11.5813	13.9832	1.3332	-8.3542	74.3285

Mean	95% CL Mean		Std Dev	95% CL Std Dev	
11.5813	8.9389	14.2238	13.9832	12.3478	16.1218

DF	t Value	Pr > |t|
109	8.69	<.0001

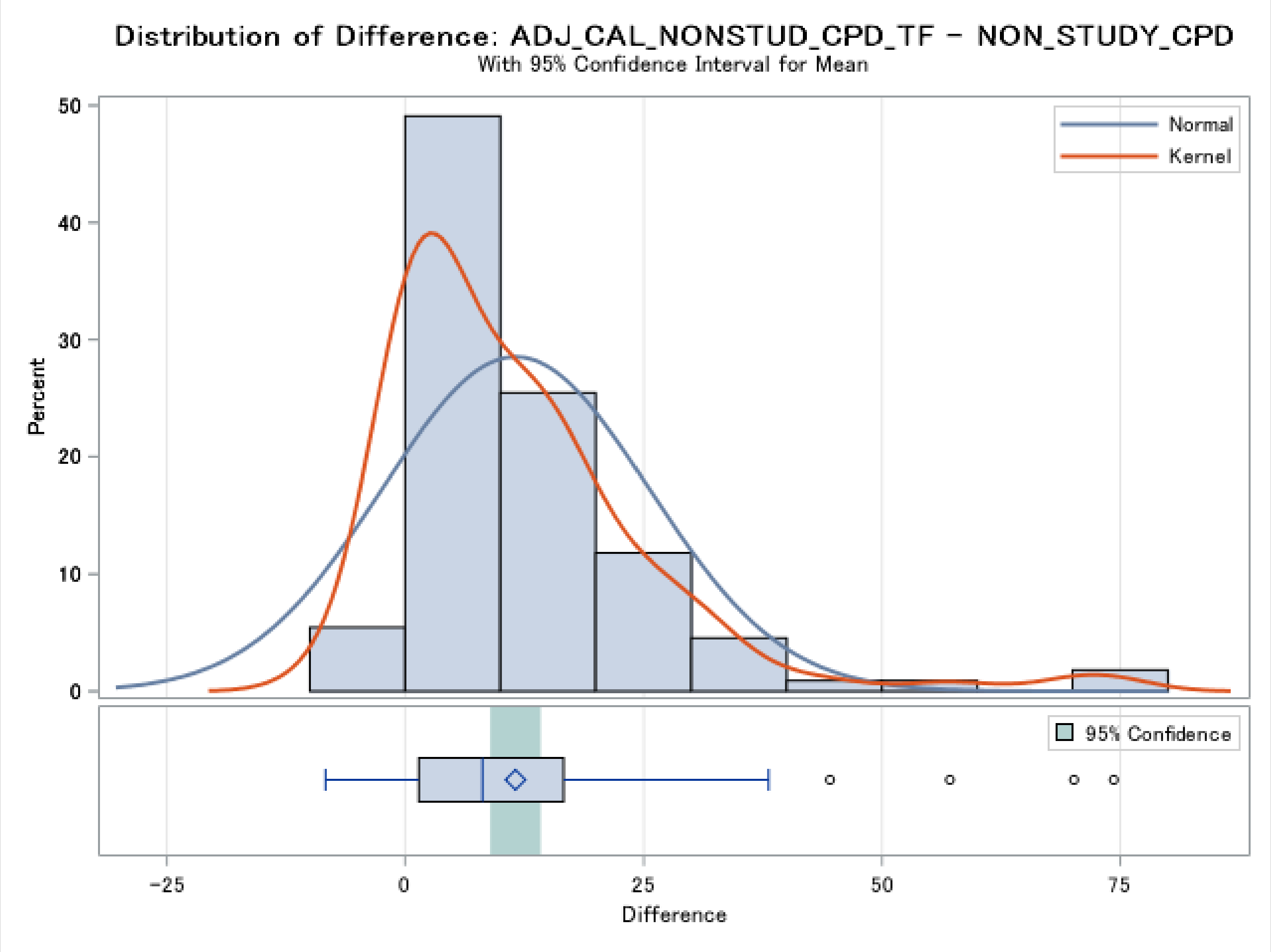

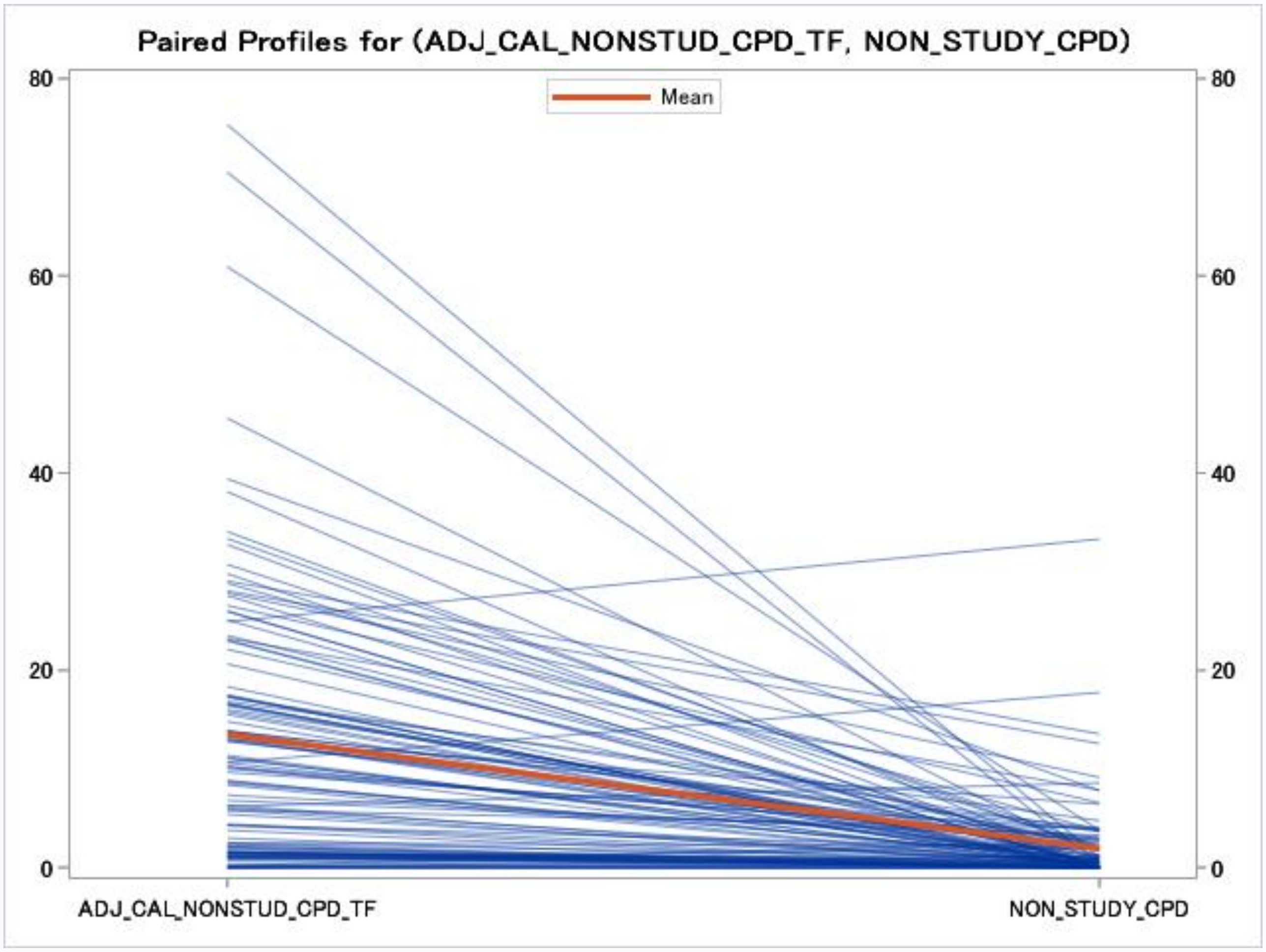

Paired Profiles for (ADJ_CAL_NONSTUD_CPD_TF, NON_STUDY_CPD)
Mean
80
60
40
20
0
ADJ_CAL_NONSTUD_CPD_TF
NON_STUDY_CPD

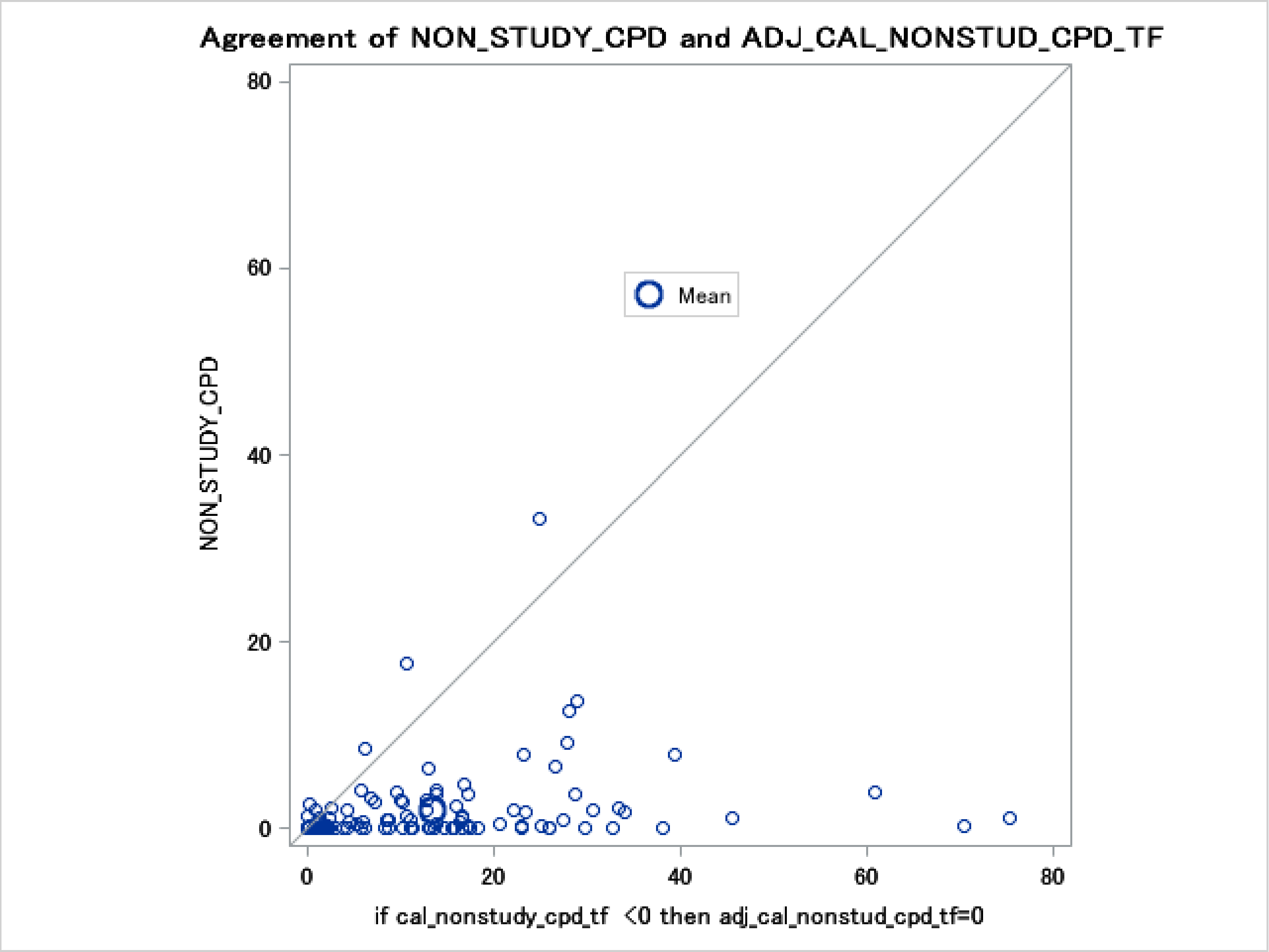
Agreement of NON_STUDY_CPD and ADJ_CAL_NONSTUD_CPD_TF
NON_STUDY_CPD
80
60
40
20
0
Mean
0
20
40
60
80
if cal_nonstudy_cpd_tf <0 then adj_cal_nonstud_cpd_tf=0

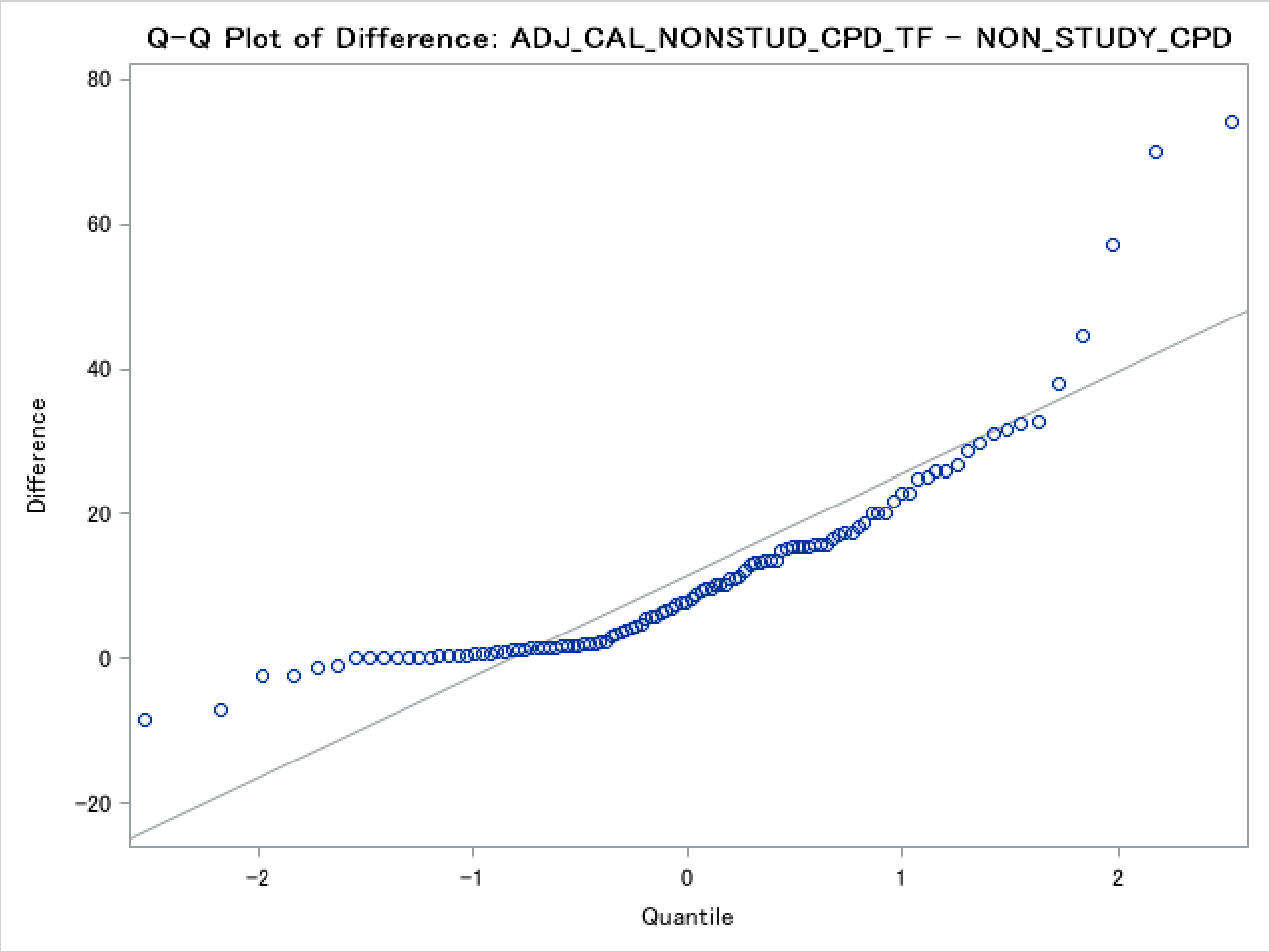

Q-Q Plot of Difference: ADJ_CAL_NONSTUD_CPD_TF - NON_STUDY_CPD
80
60
40
20
0
-20
Difference
-2
-1
0
1
2
Quantile

Paired t-test on raw self-reported and estimated non-study CPD

The TTEST Procedure

Difference: ADJ_CAL_NONSTUD_CPD_TF - NON_STUDY_CPD
AVISIT=week 2 TRTA=F ARM=5.2 mg/g

N	Mean	Std Dev	Std Err	Minimum	Maximum
110	9.5648	24.9733	2.3811	-3.6667	252.4

Mean	95% CL Mean		Std Dev	95% CL Std Dev	
9.5648	4.8455	14.2841	24.9733	22.0526	28.7927

DF	t Value	Pr > |t|
109	4.02	0.0001

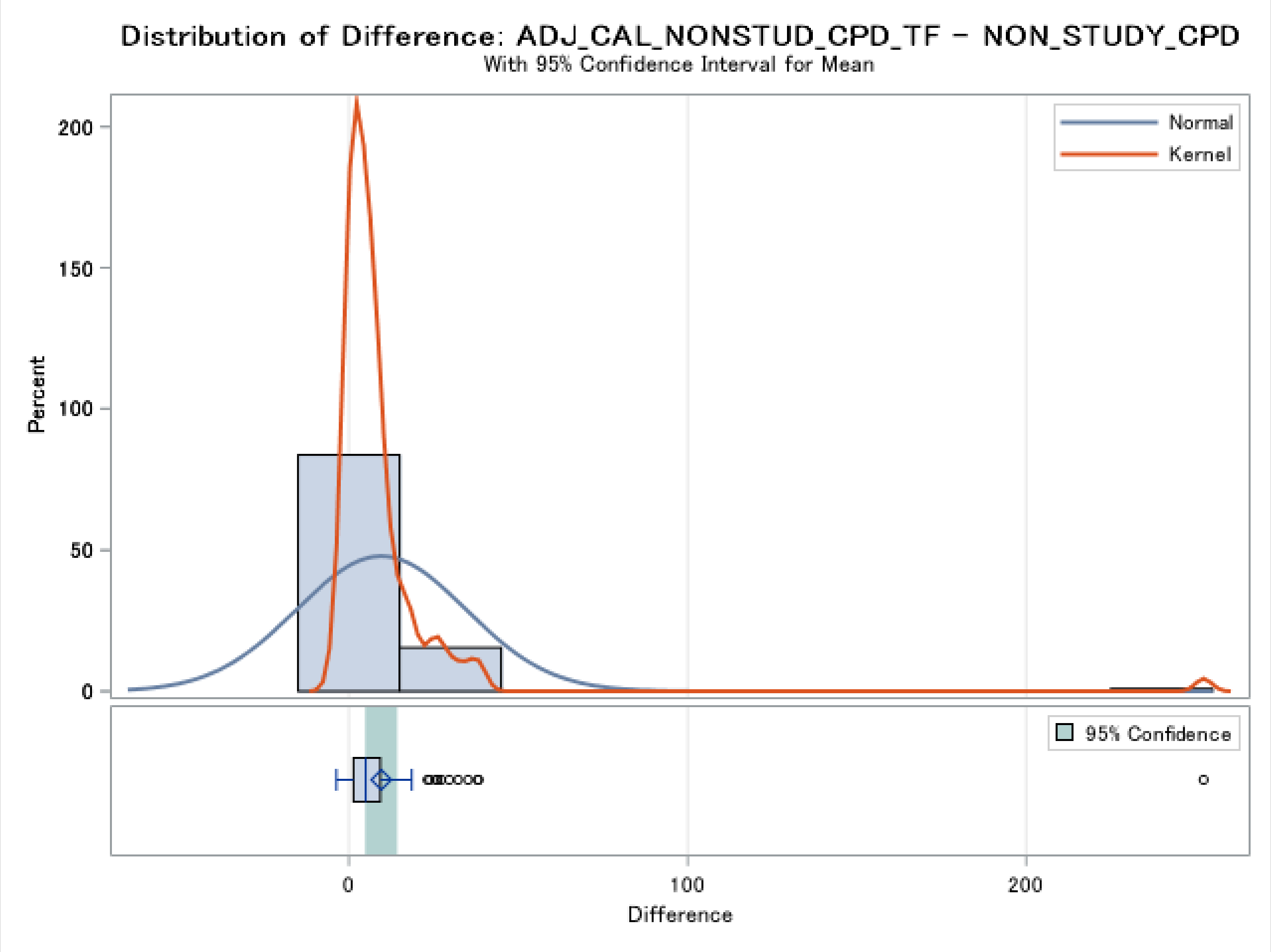

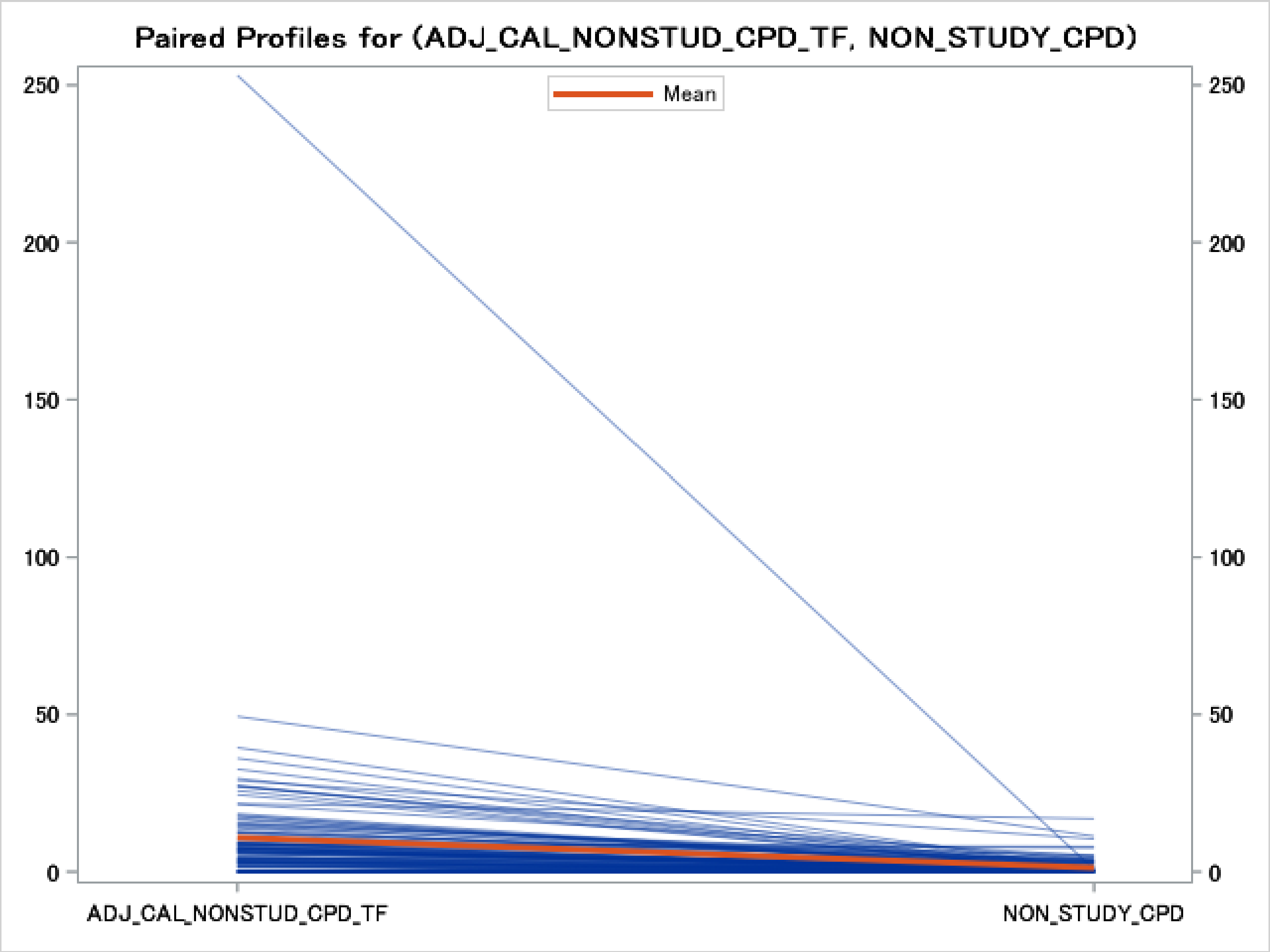
Paired Profiles for (ADJ_CAL_NONSTUD_CPD_TF, NON_STUDY_CPD)
Mean
250
200
150
100
50
0
ADJ_CAL_NONSTUD_CPD_TF
NON_STUDY_CPD

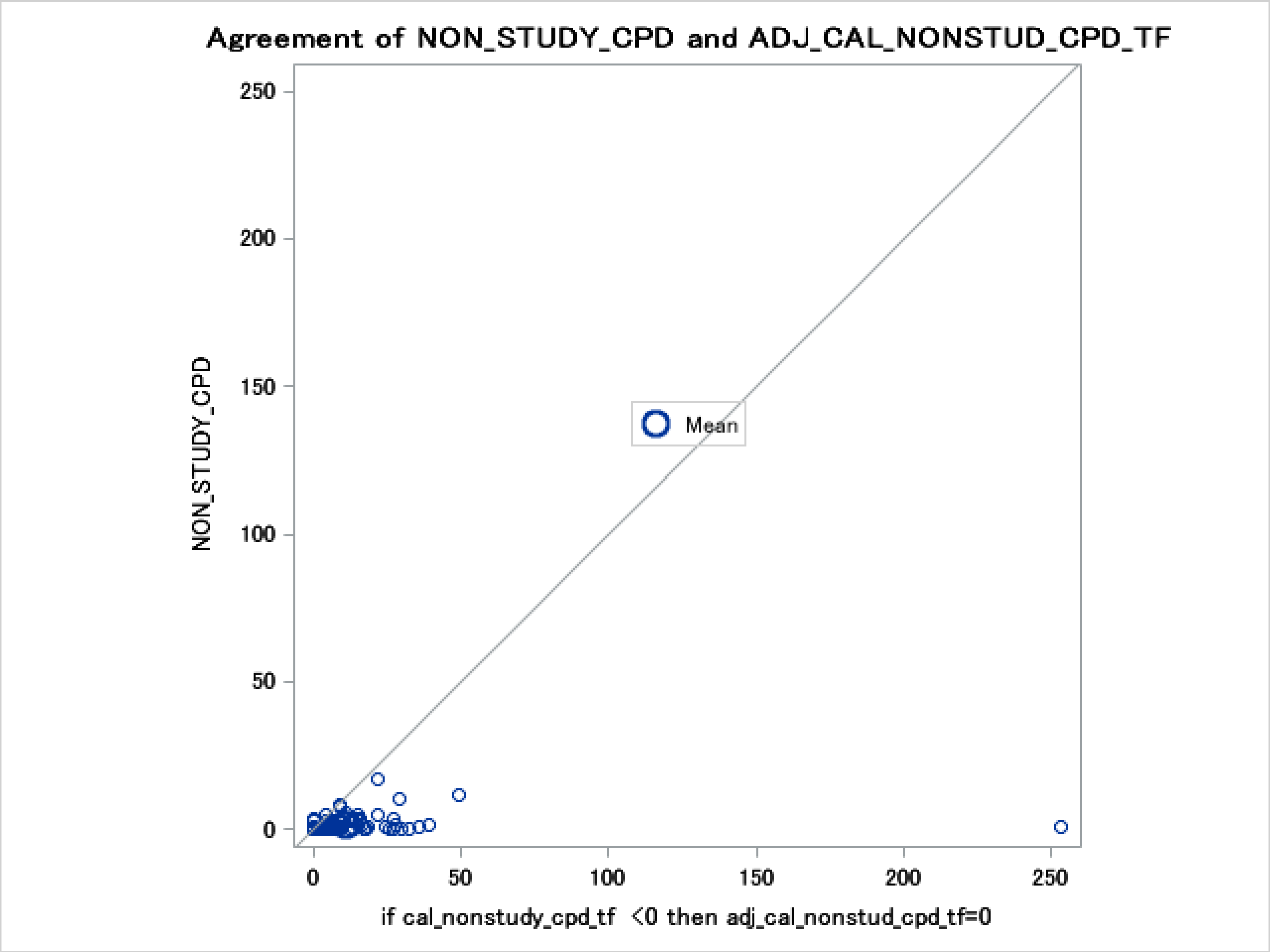

Agreement of NON_STUDY_CPD and ADJ_CAL_NONSTUD_CPD_TF
NON_STUDY_CPD
0
50
100
150
200
250
Mean
0
50
100
150
200
250
if cal_nonstudy_cpd_tf <0 then adj_cal_nonstud_cpd_tf=0

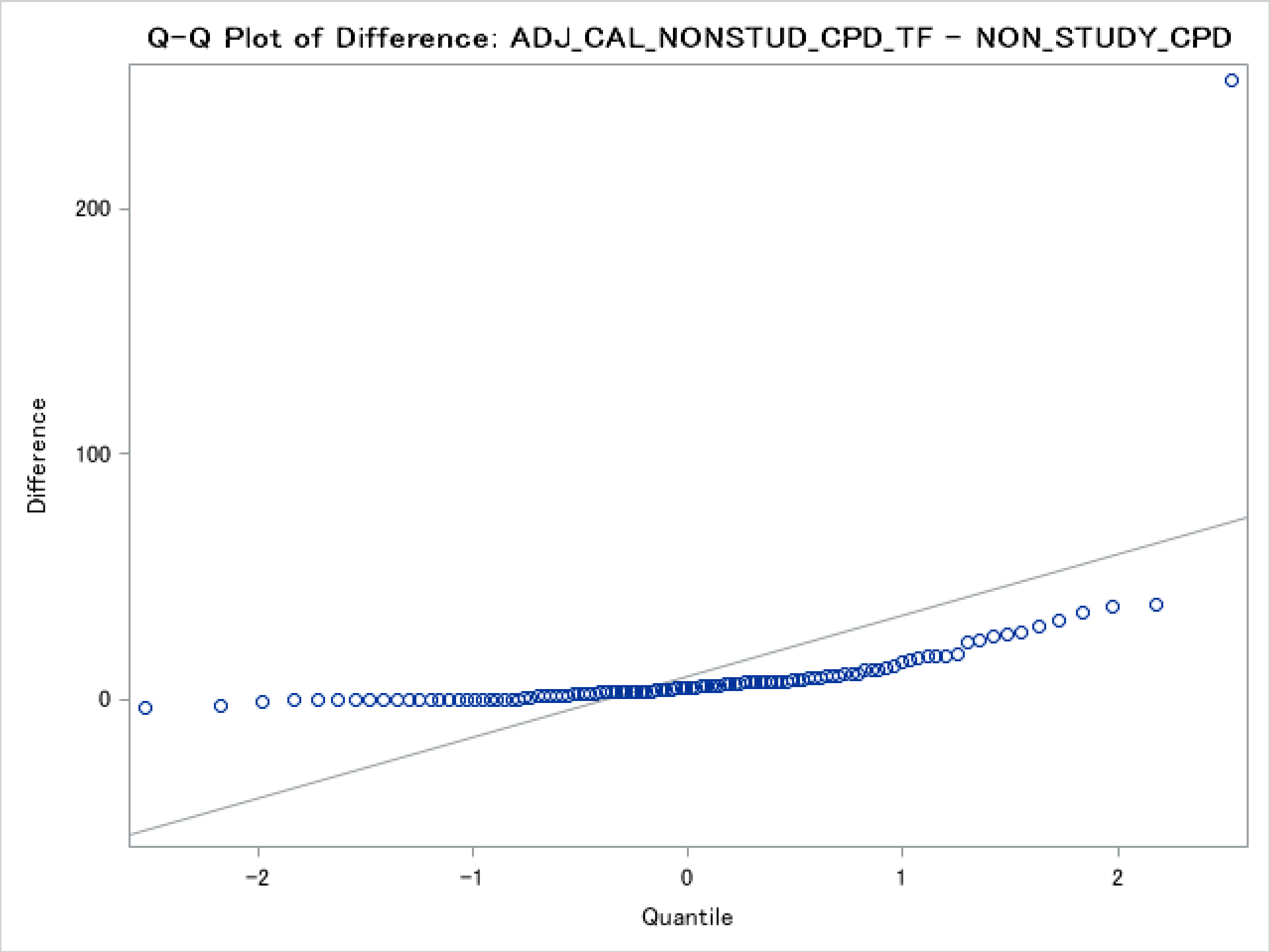
Q-Q Plot of Difference: ADJ_CAL_NONSTUD_CPD_TF - NON_STUDY_CPD
Difference
200
100
0
−2
−1
0
1
2
Quantile

Paired t-test on raw self-reported and estimated non-study CPD

The TTEST Procedure

Difference: ADJ_CAL_NONSTUD_CPD_TF - NON_STUDY_CPD
AVISIT=week 2 TRTA=G ARM=0.4 mg/g

N	Mean	Std Dev	Std Err	Minimum	Maximum
111	14.6297	21.4020	2.0314	-3.1607	142.1

Mean	95% CL Mean		Std Dev	95% CL Std Dev	
14.6297	10.6040	18.6554	21.4020	18.9090	24.6581

DF	t Value	Pr > |t|
110	7.20	<.0001

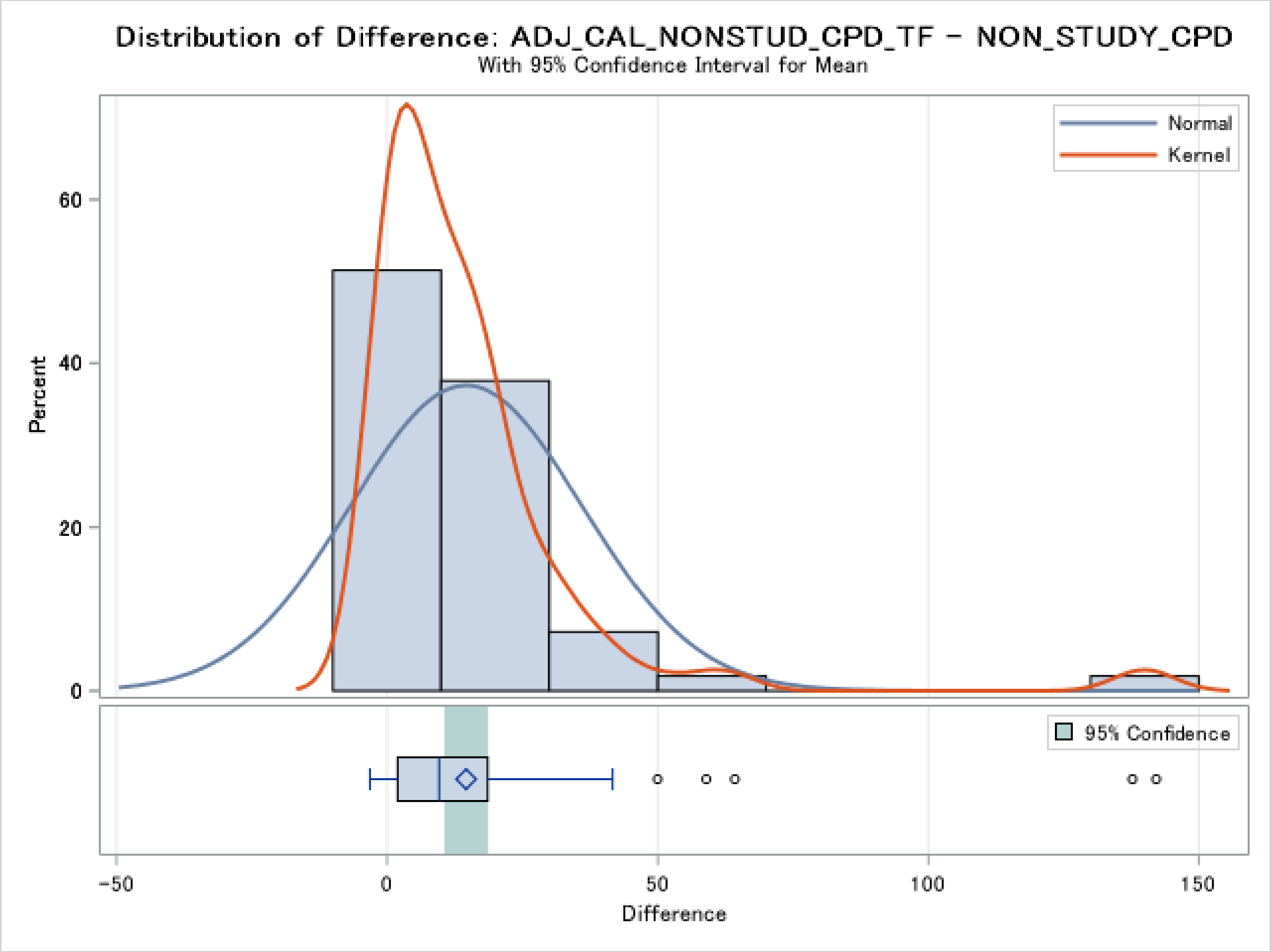

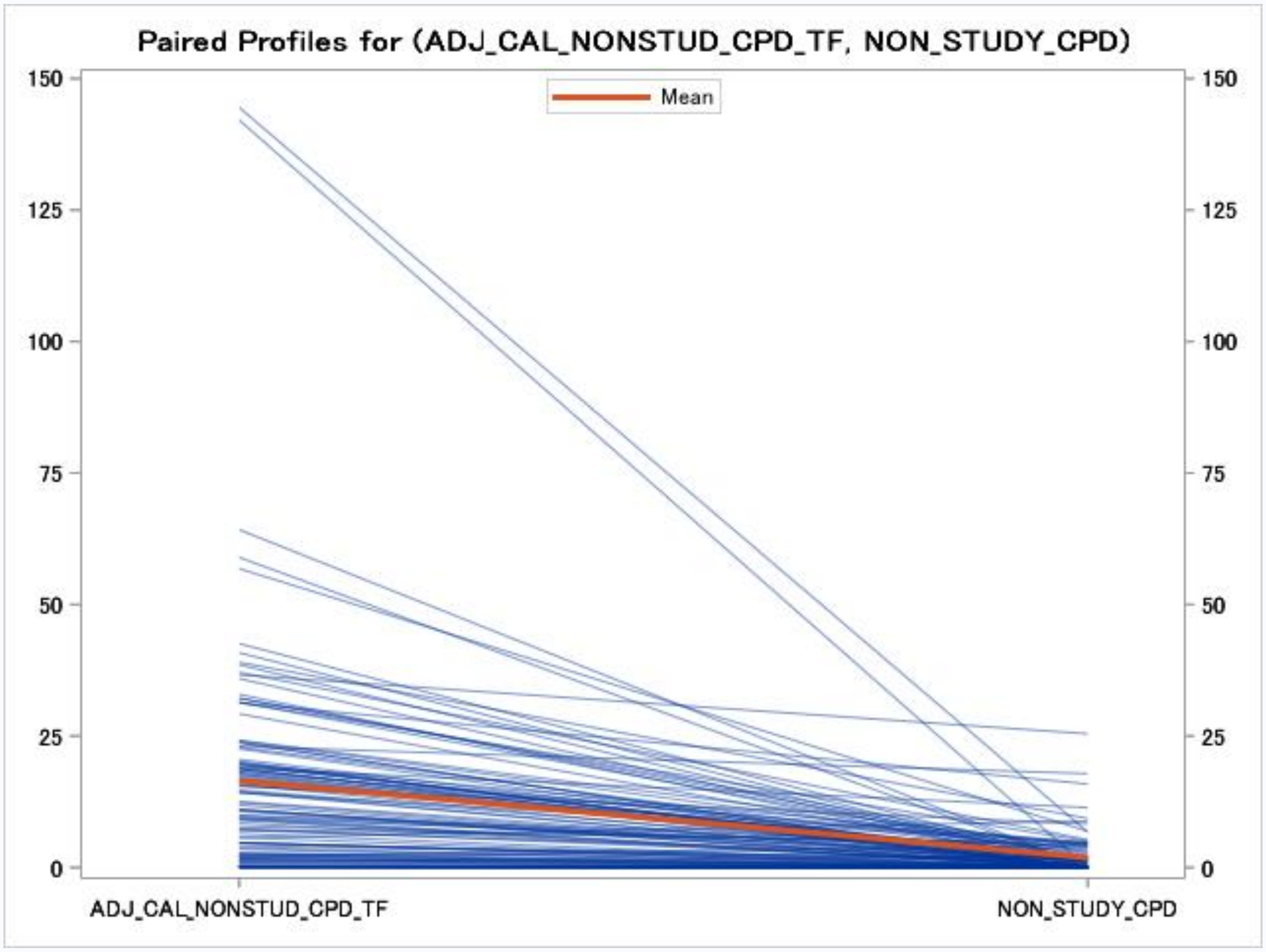

Paired Profiles for (ADJ_CAL_NONSTUD_CPD_TF, NON_STUDY_CPD)
Mean
150
125
100
75
50
25
0
ADJ_CAL_NONSTUD_CPD_TF
NON_STUDY_CPD

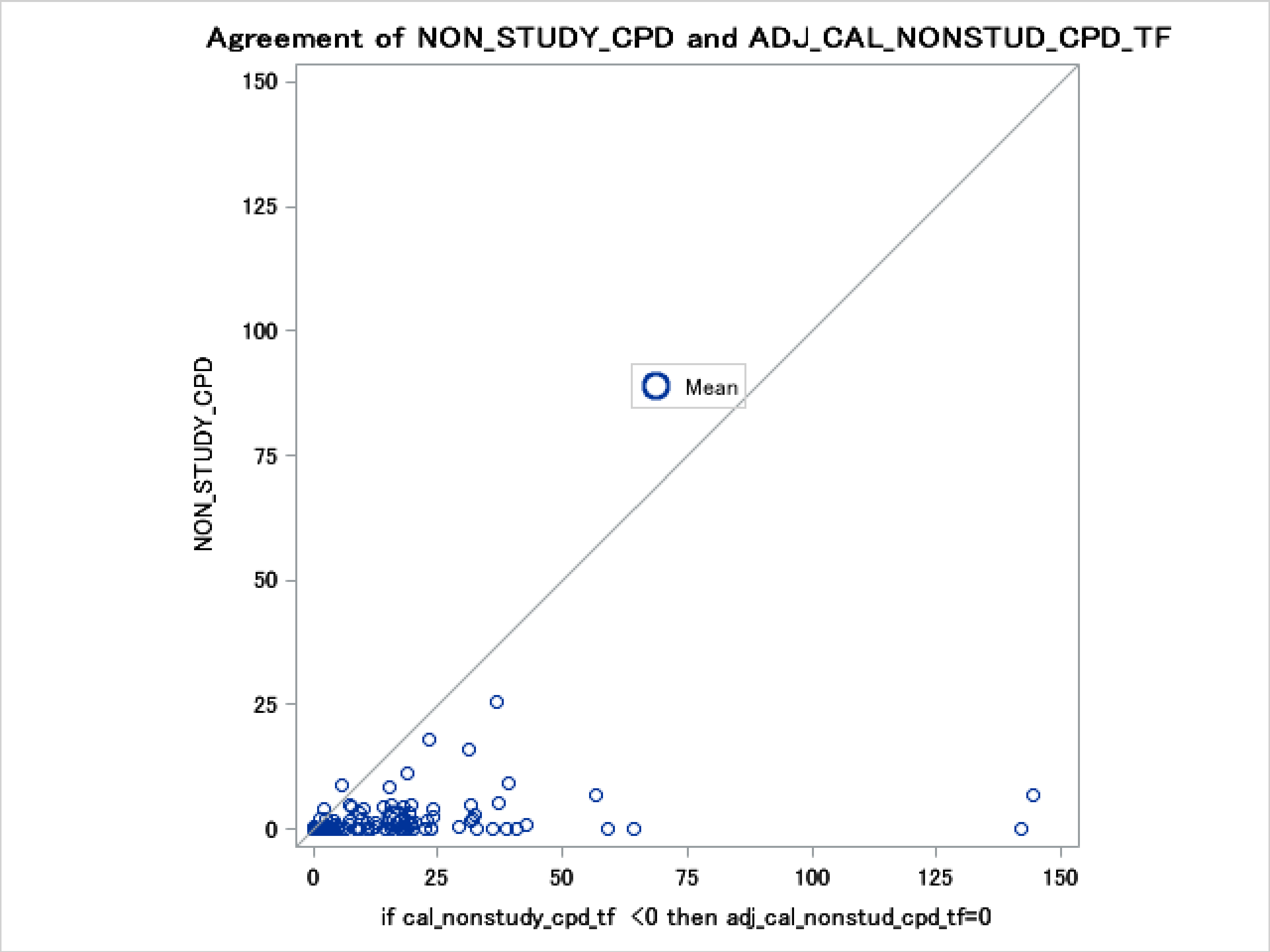
Agreement of NON_STUDY_CPD and ADJ_CAL_NONSTUD_CPD_TF
150
125
100
75
50
25
0
NON_STUDY_CPD
Mean
0
25
50
75
100
125
150
if cal_nonstudy_cpd_tf <0 then adj_cal_nonstud_cpd_tf=0

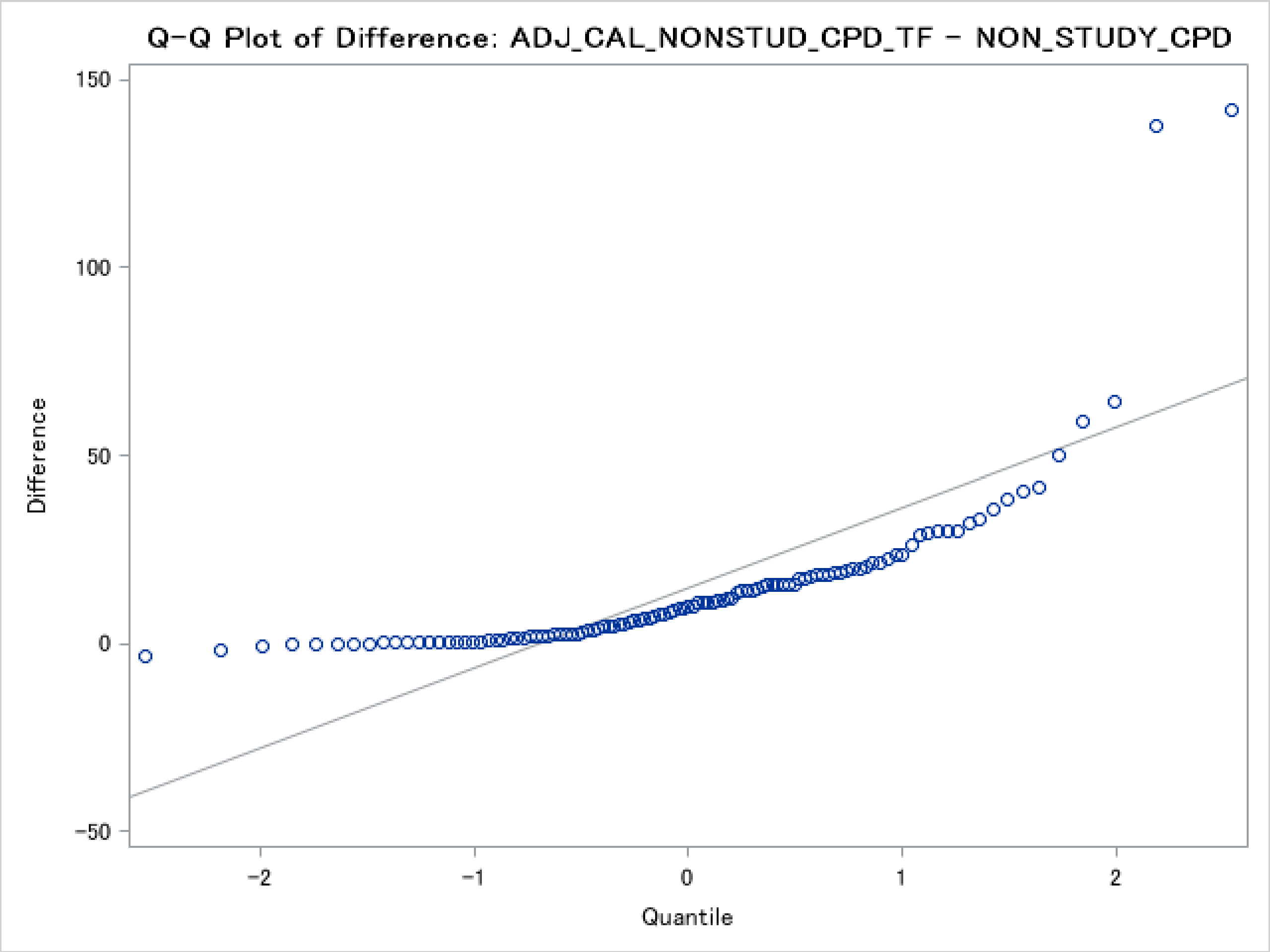
Q-Q Plot of Difference: ADJ_CAL_NONSTUD_CPD_TF - NON_STUDY_CPD
150
100
50
0
-50
Difference
-2
-1
0
1
2
Quantile

Paired t-test on raw self-reported and estimated non-study CPD

The TTEST Procedure

Difference: ADJ_CAL_NONSTUD_CPD_TF - NON_STUDY_CPD
AVISIT=week 6 TRTA=A ARM=2.4 mg/g

N	Mean	Std Dev	Std Err	Minimum	Maximum
106	10.8919	10.8017	1.0492	-6.5301	48.3089

Mean	95% CL Mean		Std Dev	95% CL Std Dev	
10.8919	8.8117	12.9722	10.8017	9.5175	12.4897

DF	t Value	Pr > |t|
105	10.38	<.0001

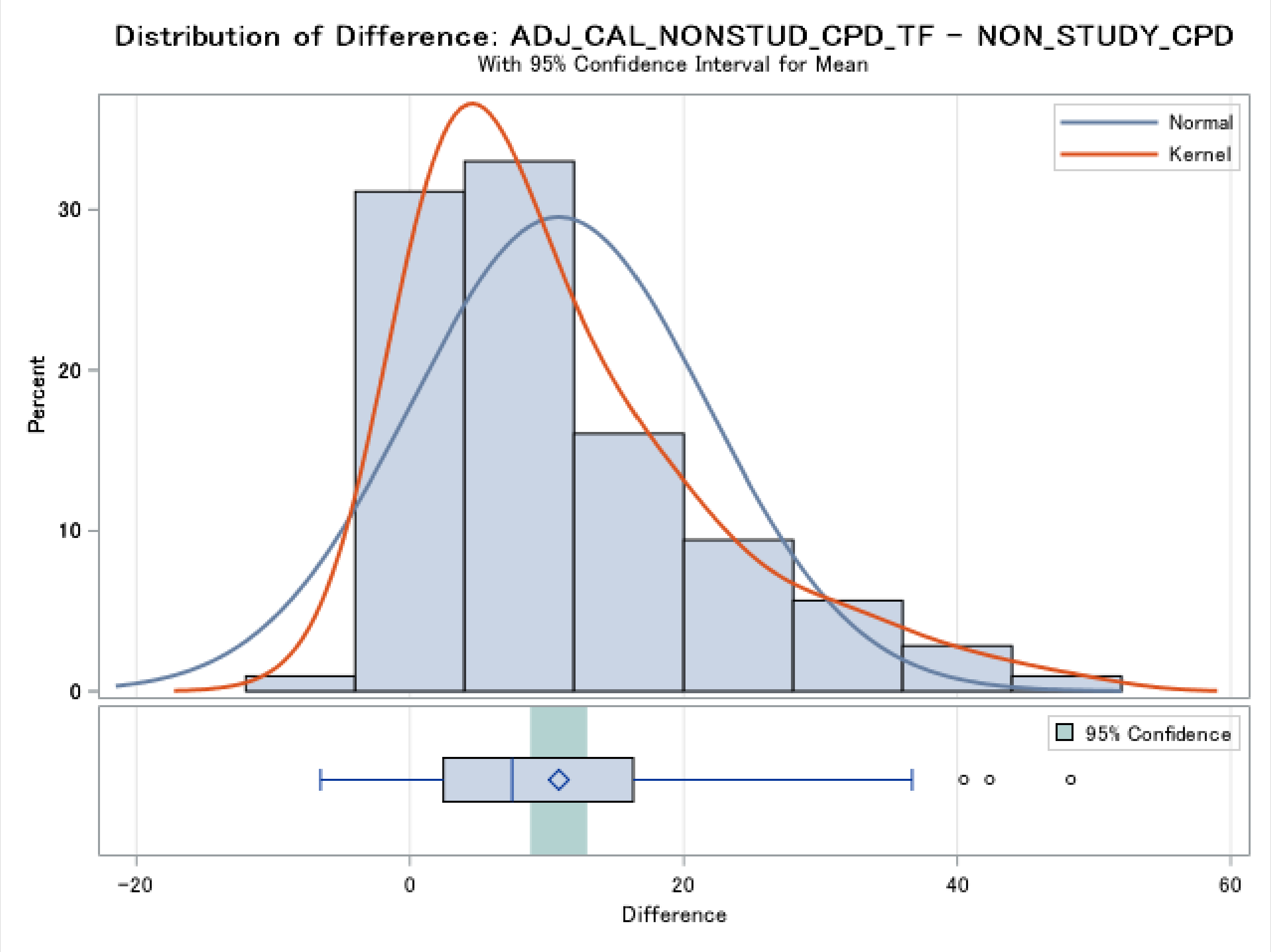

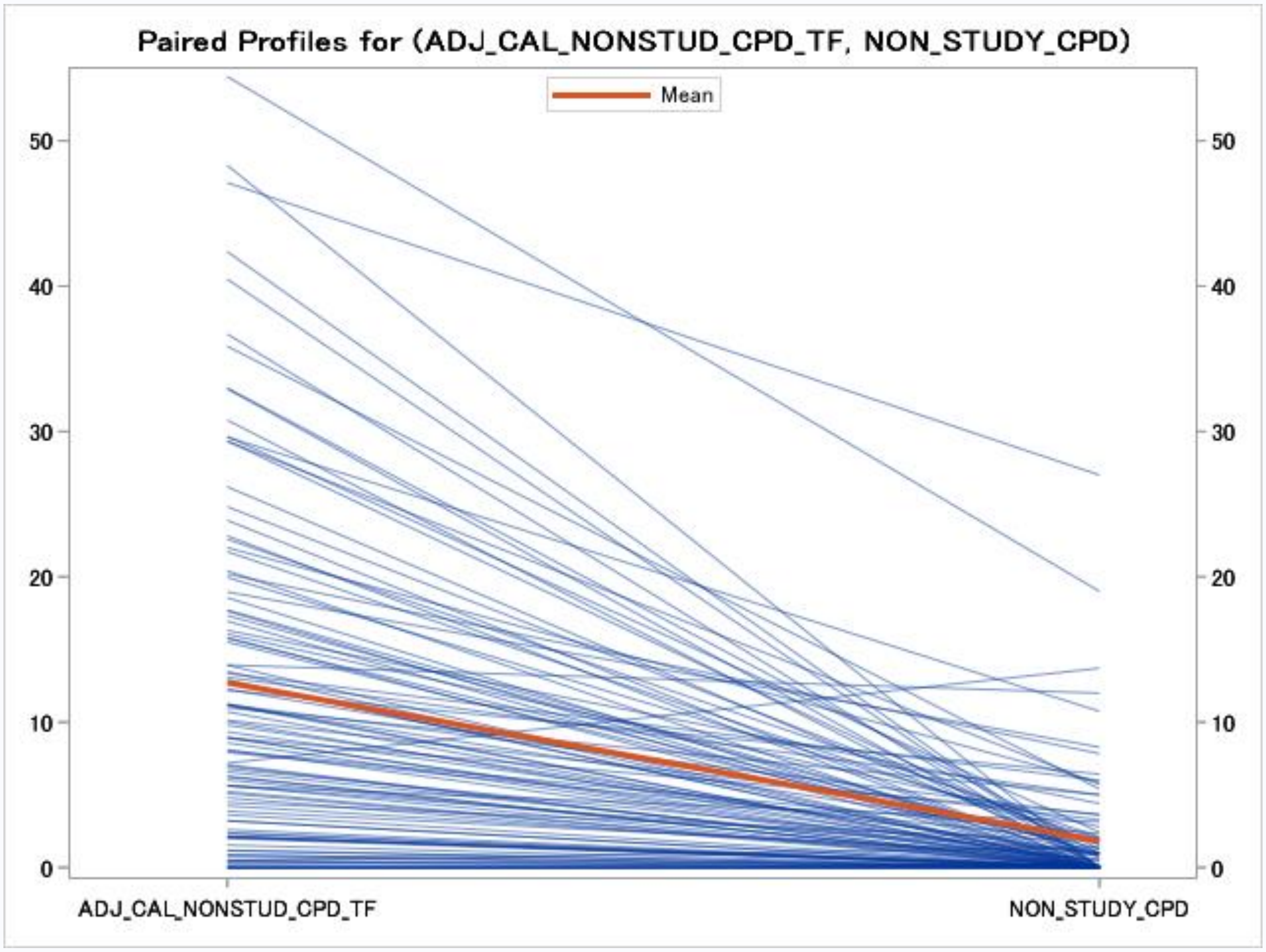

Paired Profiles for (ADJ_CAL_NONSTUD_CPD_TF, NON_STUDY_CPD)
Mean
0
10
20
30
40
50
ADJ_CAL_NONSTUD_CPD_TF
NON_STUDY_CPD

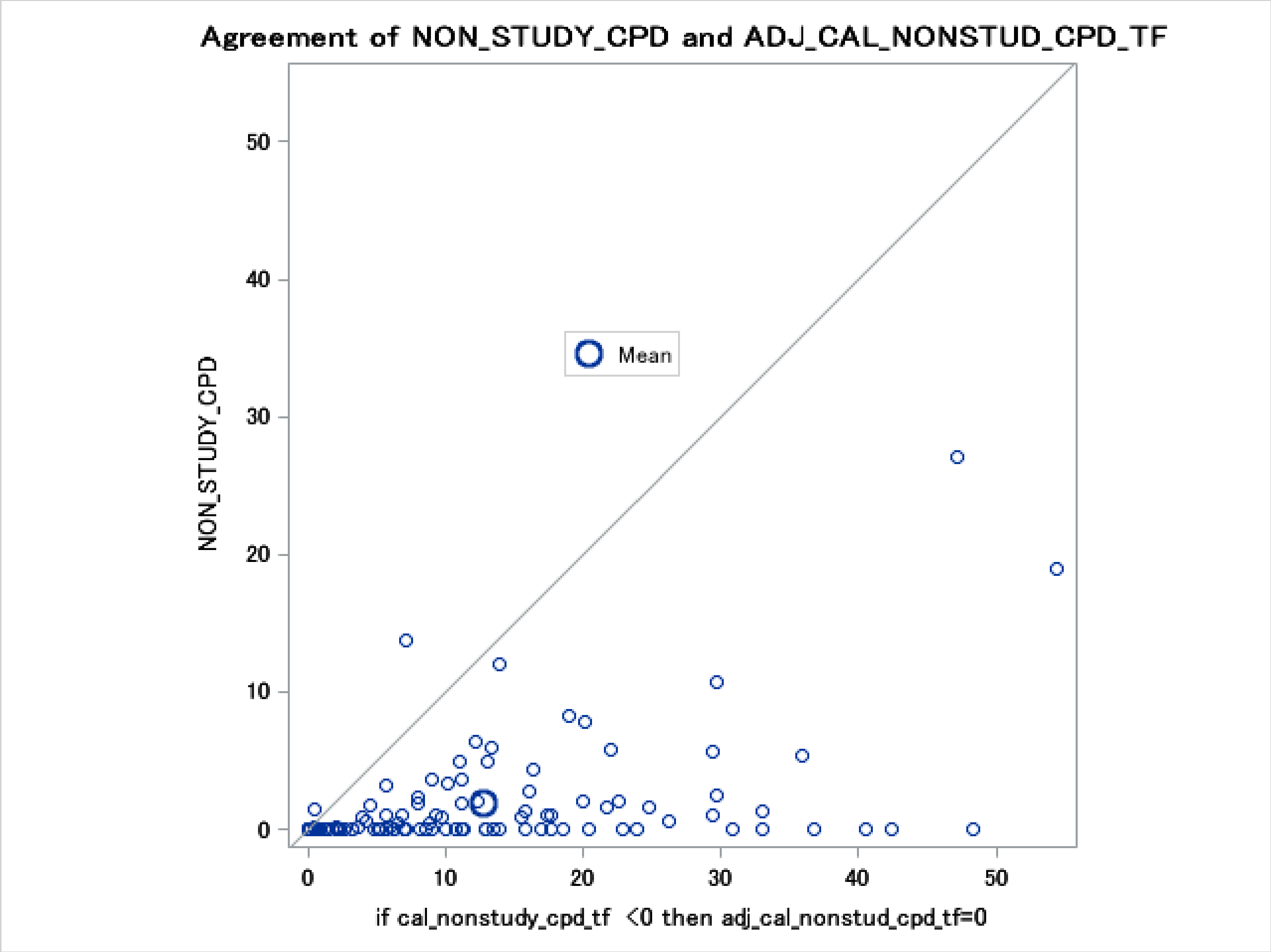

Agreement of NON_STUDY_CPD and ADJ_CAL_NONSTUD_CPD_TF
NON_STUDY_CPD
Mean
0
10
20
30
40
50
if cal_nonstudy_cpd_tf <0 then adj_cal_nonstud_cpd_tf=0

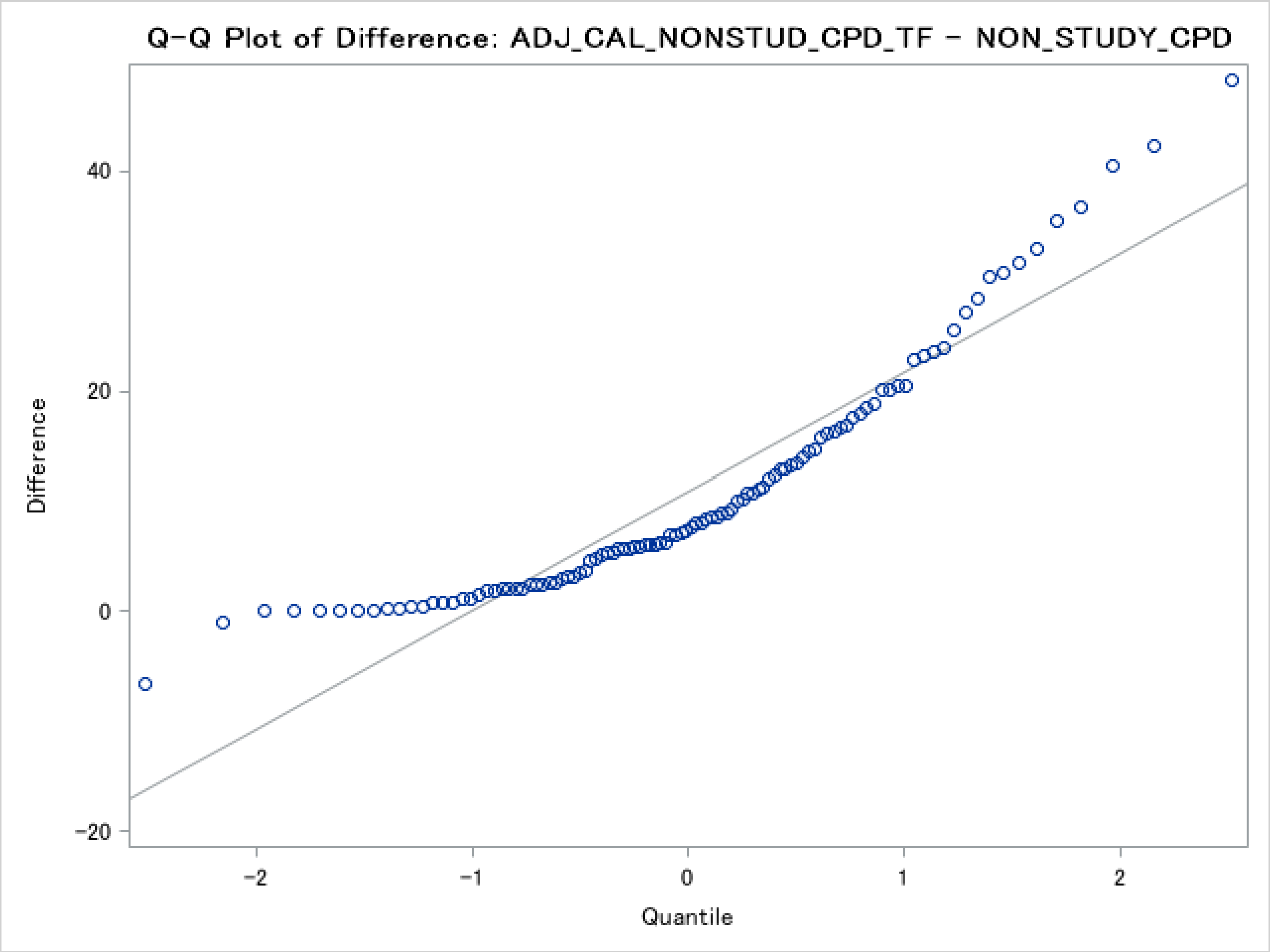

Q-Q Plot of Difference: ADJ_CAL_NONSTUD_CPD_TF - NON_STUDY_CPD
Difference
40
20
0
-20
-2
-1
0
1
2
Quantile

Paired t-test on raw self-reported and estimated non-study CPD

The TTEST Procedure

Difference: ADJ_CAL_NONSTUD_CPD_TF - NON_STUDY_CPD
AVISIT=week 6 TRTA=B ARM=0.4 mg/g (HT)

N	Mean	Std Dev	Std Err	Minimum	Maximum
113	11.1654	11.4981	1.0816	-3.7350	43.9397

Mean	95% CL Mean		Std Dev	95% CL Std Dev	
11.1654	9.0223	13.3086	11.4981	10.1693	13.2294

DF	t Value	Pr > |t|
112	10.32	<.0001

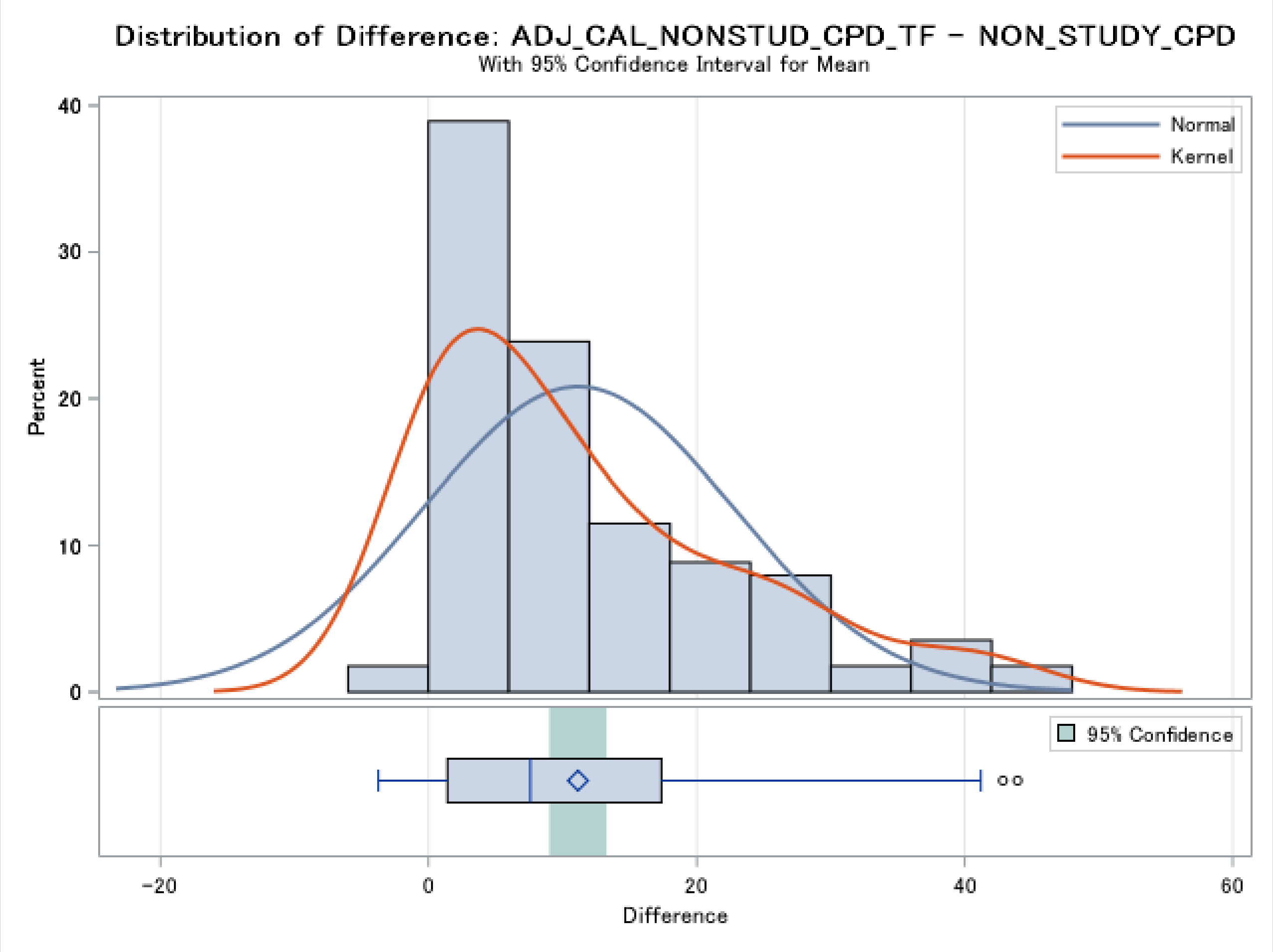

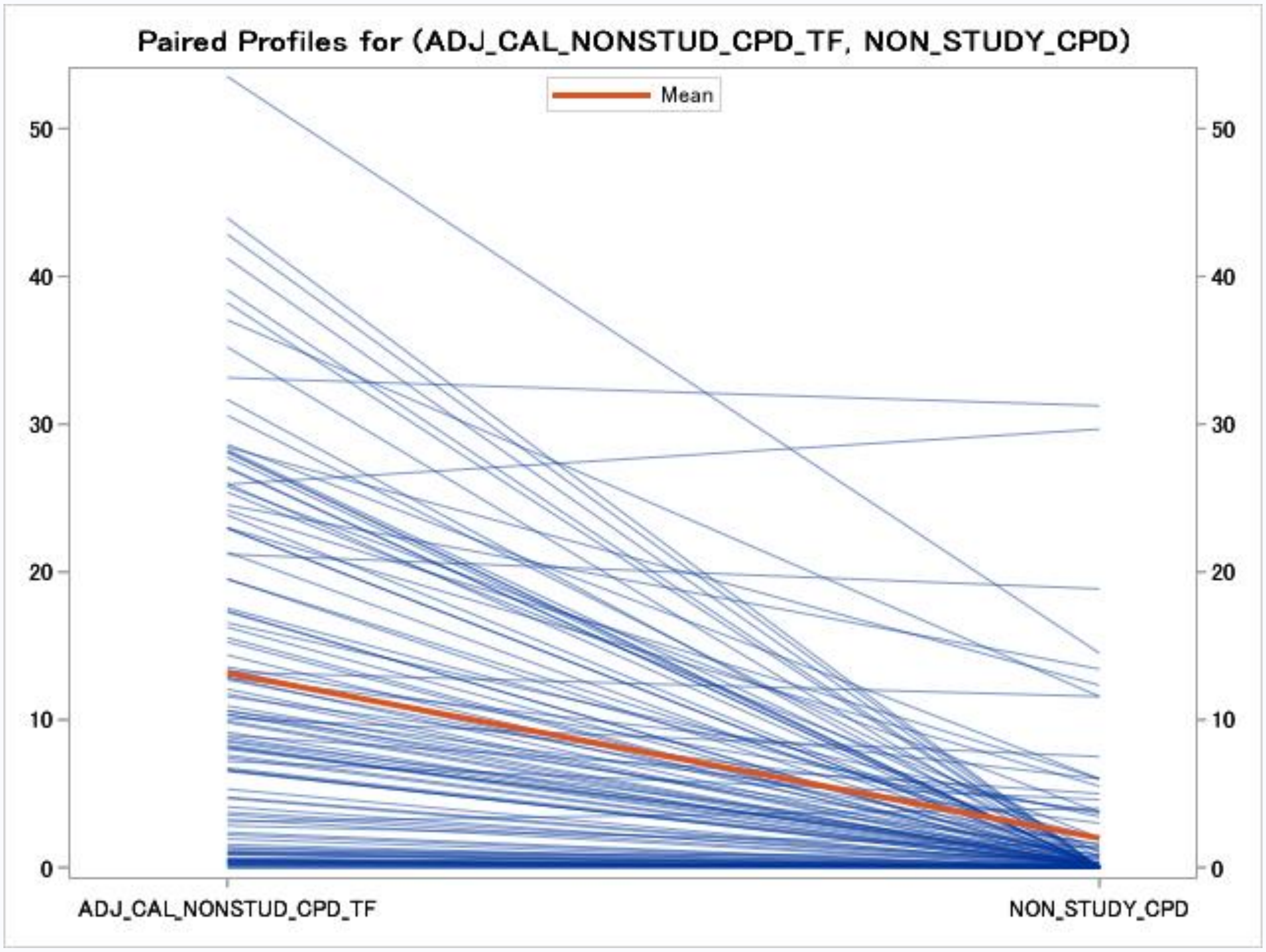

Paired Profiles for (ADJ_CAL_NONSTUD_CPD_TF, NON_STUDY_CPD)
Mean
50
40
30
20
10
0
ADJ_CAL_NONSTUD_CPD_TF
NON_STUDY_CPD

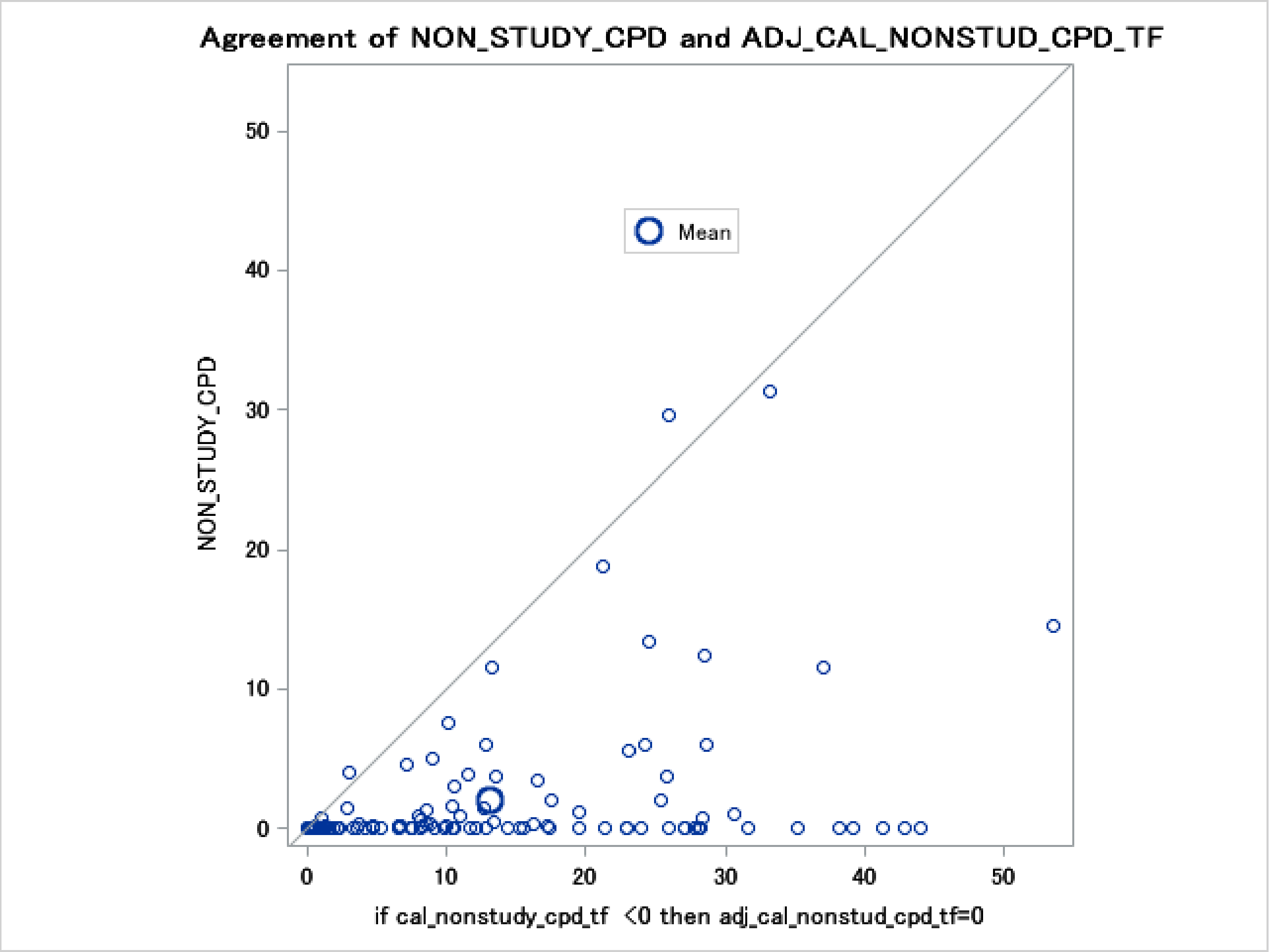
Agreement of NON_STUDY_CPD and ADJ_CAL_NONSTUD_CPD_TF
Mean
NON_STUDY_CPD
0
10
20
30
40
50
if cal_nonstudy_cpd_tf <0 then adj_cal_nonstud_cpd_tf=0

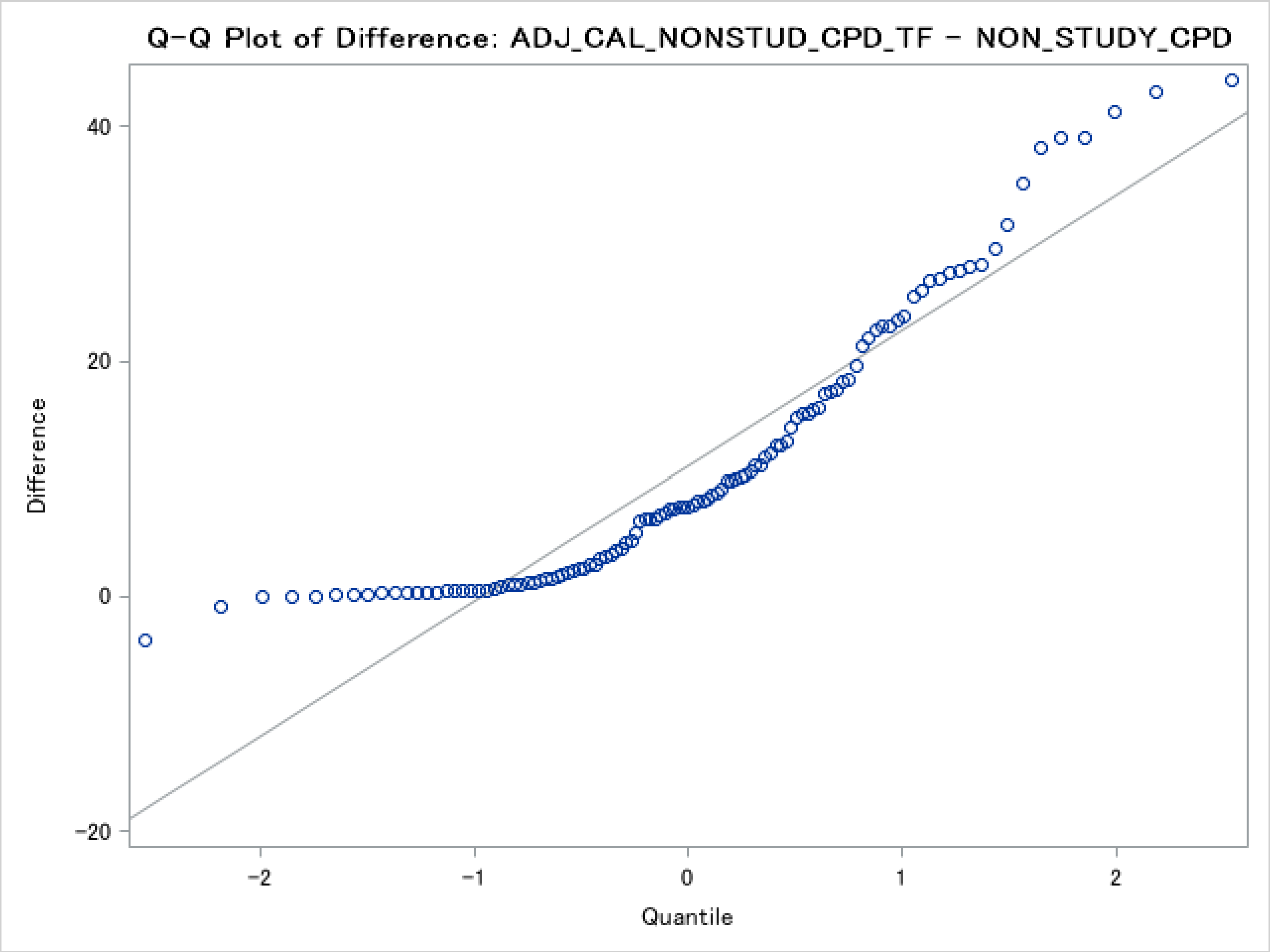

Q-Q Plot of Difference: ADJ_CAL_NONSTUD_CPD_TF - NON_STUDY_CPD
Difference
40
20
0
-20
-2
-1
0
1
2
Quantile

Paired t-test on raw self-reported and estimated non-study CPD

The TTEST Procedure

Difference: ADJ_CAL_NONSTUD_CPD_TF - NON_STUDY_CPD
AVISIT=week 6 TRTA=D ARM=1.3 mg/g

N	Mean	Std Dev	Std Err	Minimum	Maximum
106	13.6707	12.9025	1.2532	-0.5365	82.3516

Mean	95% CL Mean		Std Dev	95% CL Std Dev	
13.6707	11.1858	16.1556	12.9025	11.3685	14.9188

DF	t Value	Pr > |t|
105	10.91	<.0001

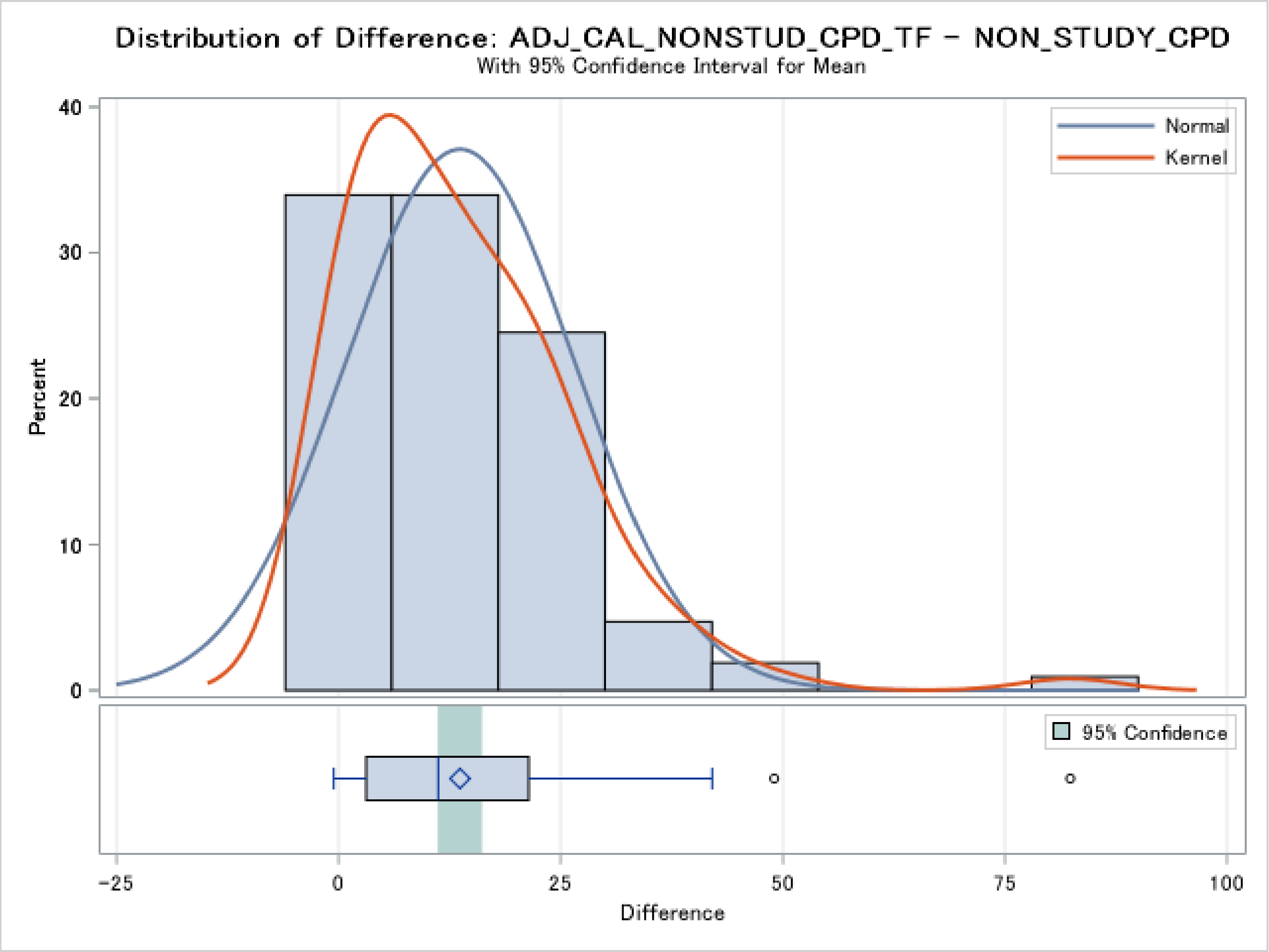

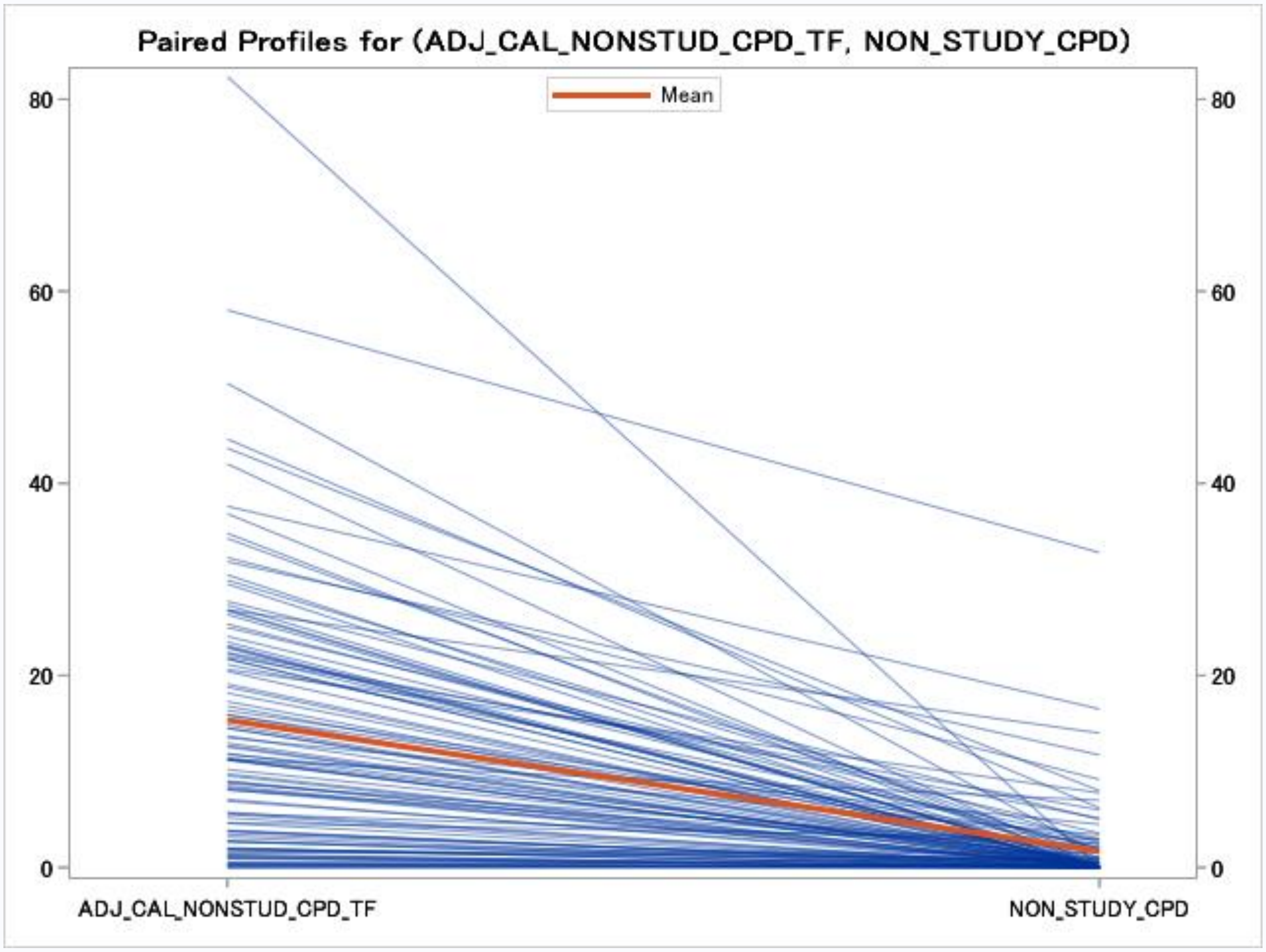

Paired Profiles for (ADJ_CAL_NONSTUD_CPD_TF, NON_STUDY_CPD)
Mean
80
60
40
20
0
ADJ_CAL_NONSTUD_CPD_TF
NON_STUDY_CPD

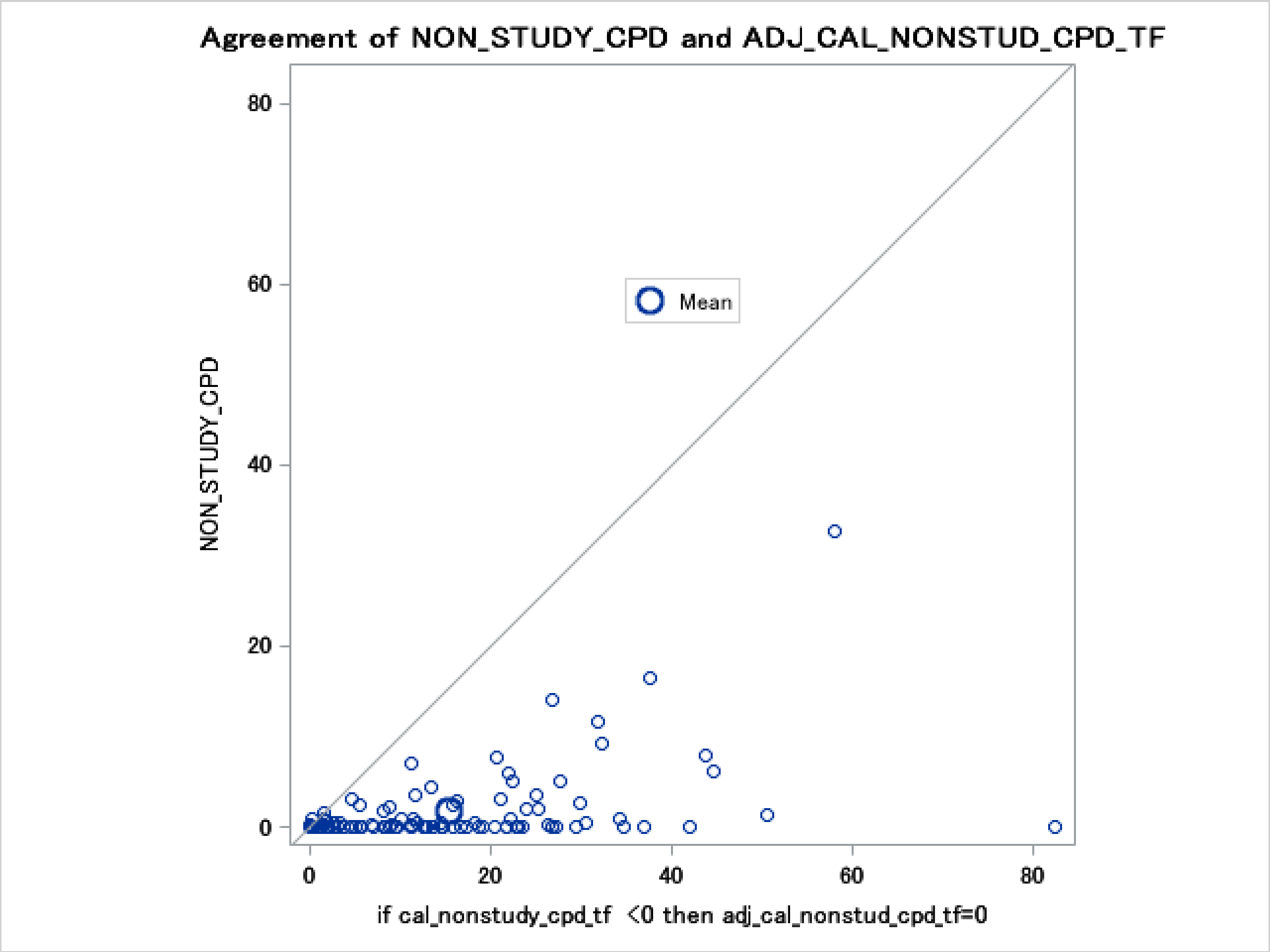

Agreement of NON_STUDY_CPD and ADJ_CAL_NONSTUD_CPD_TF
Mean
NON_STUDY_CPD
0
20
40
60
80
if cal_nonstudy_cpd_tf <0 then adj_cal_nonstud_cpd_tf=0

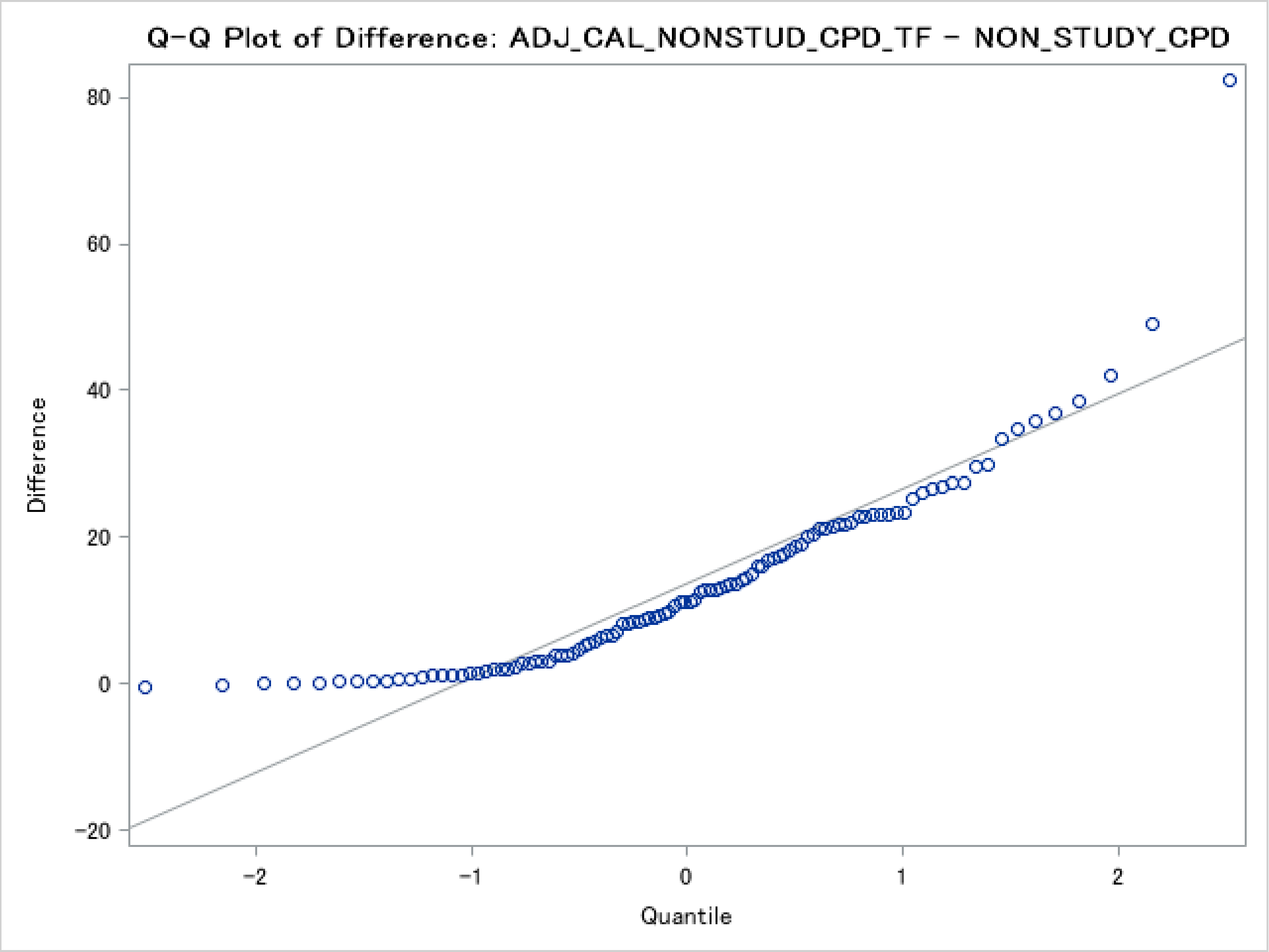
Q-Q Plot of Difference: ADJ_CAL_NONSTUD_CPD_TF - NON_STUDY_CPD
Difference
80
60
40
20
0
-20
-2
-1
0
1
2
Quantile

Paired t-test on raw self-reported and estimated non-study CPD

The TTEST Procedure

Difference: ADJ_CAL_NONSTUD_CPD_TF - NON_STUDY_CPD
AVISIT=week 6 TRTA=F ARM=5.2 mg/g

N	Mean	Std Dev	Std Err	Minimum	Maximum
106	9.2118	8.6354	0.8387	-1.0000	51.6750

Mean	95% CL Mean		Std Dev	95% CL Std Dev	
9.2118	7.5487	10.8748	8.6354	7.6087	9.9848

DF	t Value	Pr > |t|
105	10.98	<.0001

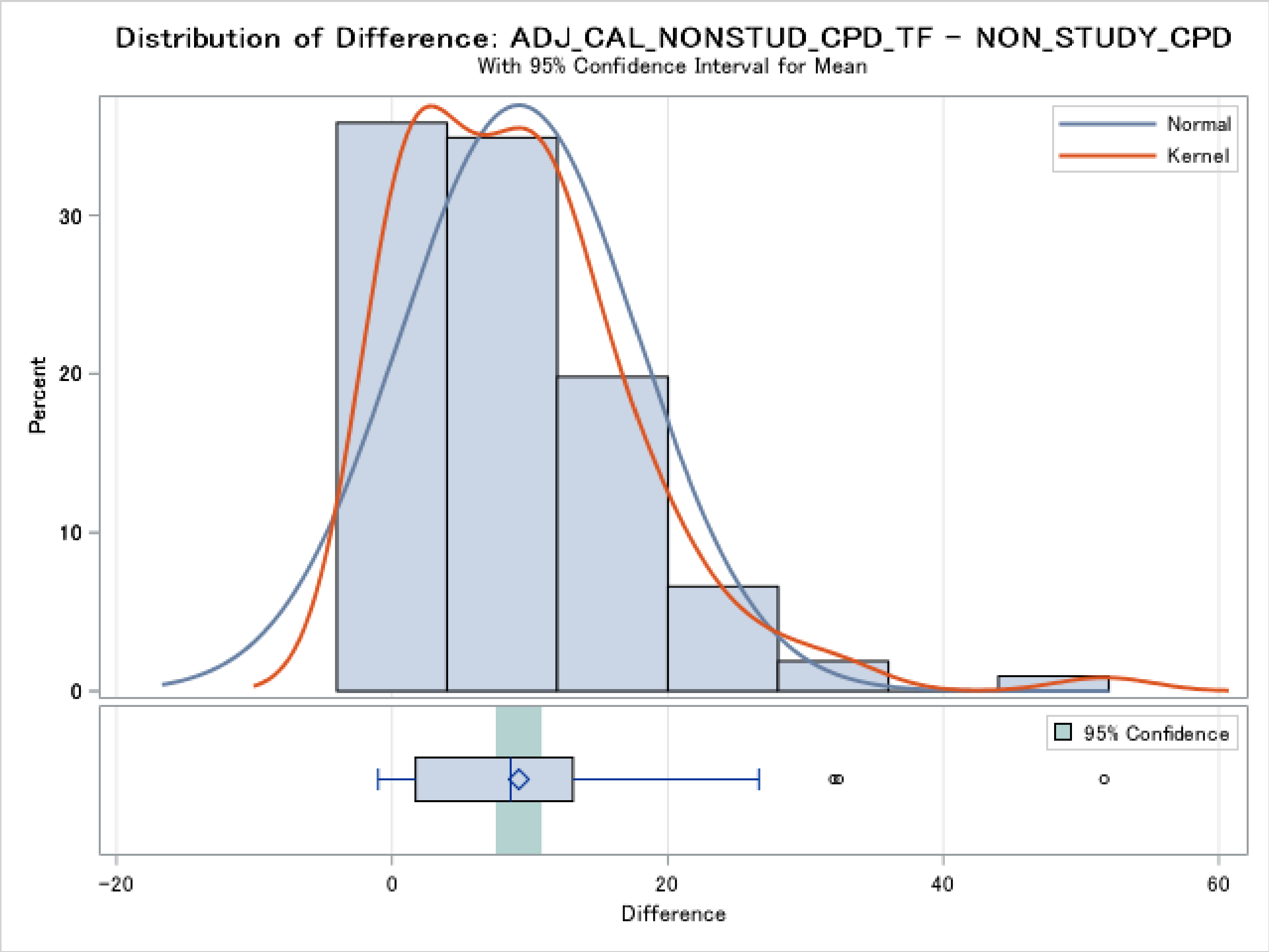

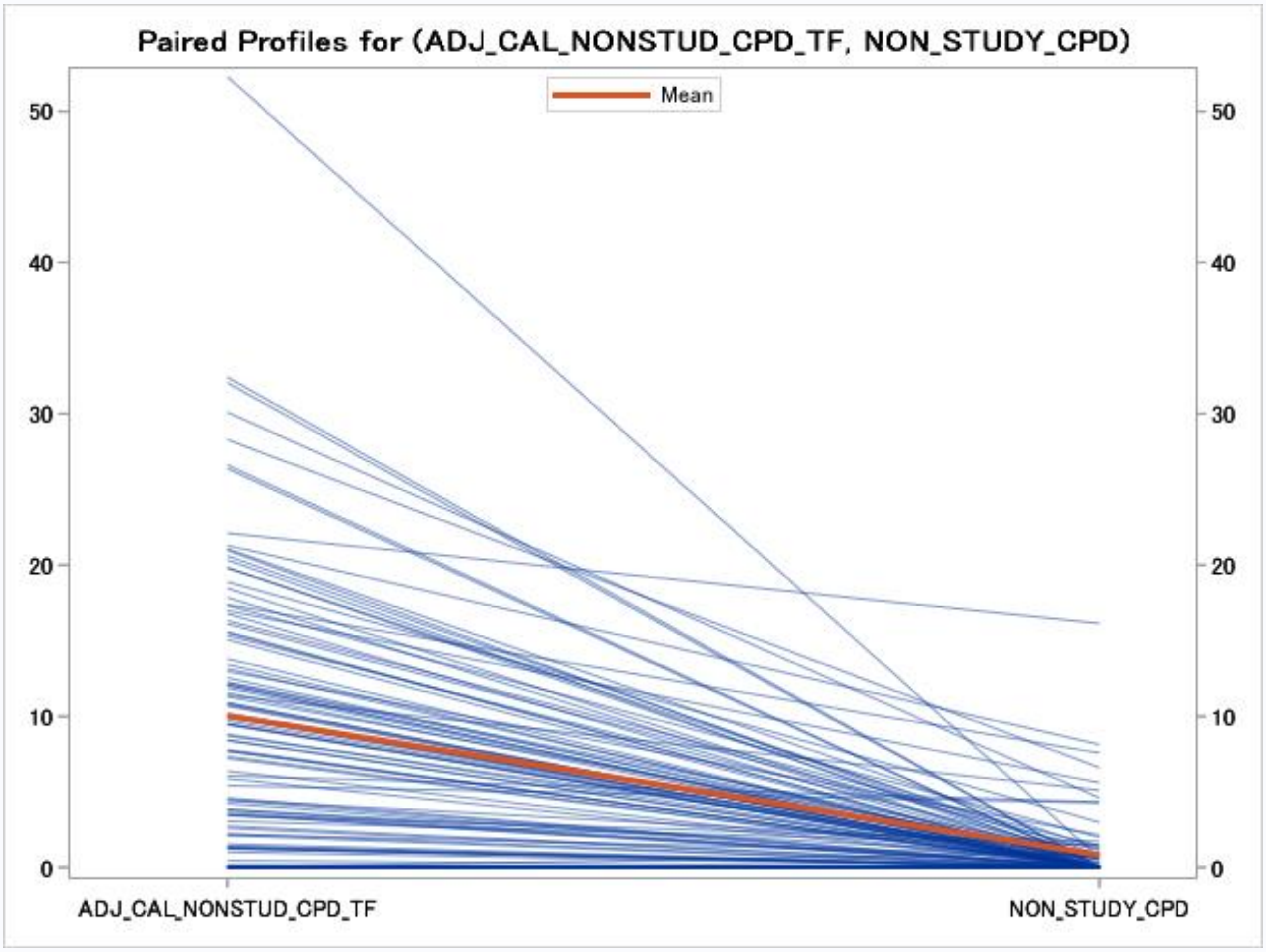

Paired Profiles for (ADJ_CAL_NONSTUD_CPD_TF, NON_STUDY_CPD)
Mean
50
40
30
20
10
0
ADJ_CAL_NONSTUD_CPD_TF
NON_STUDY_CPD

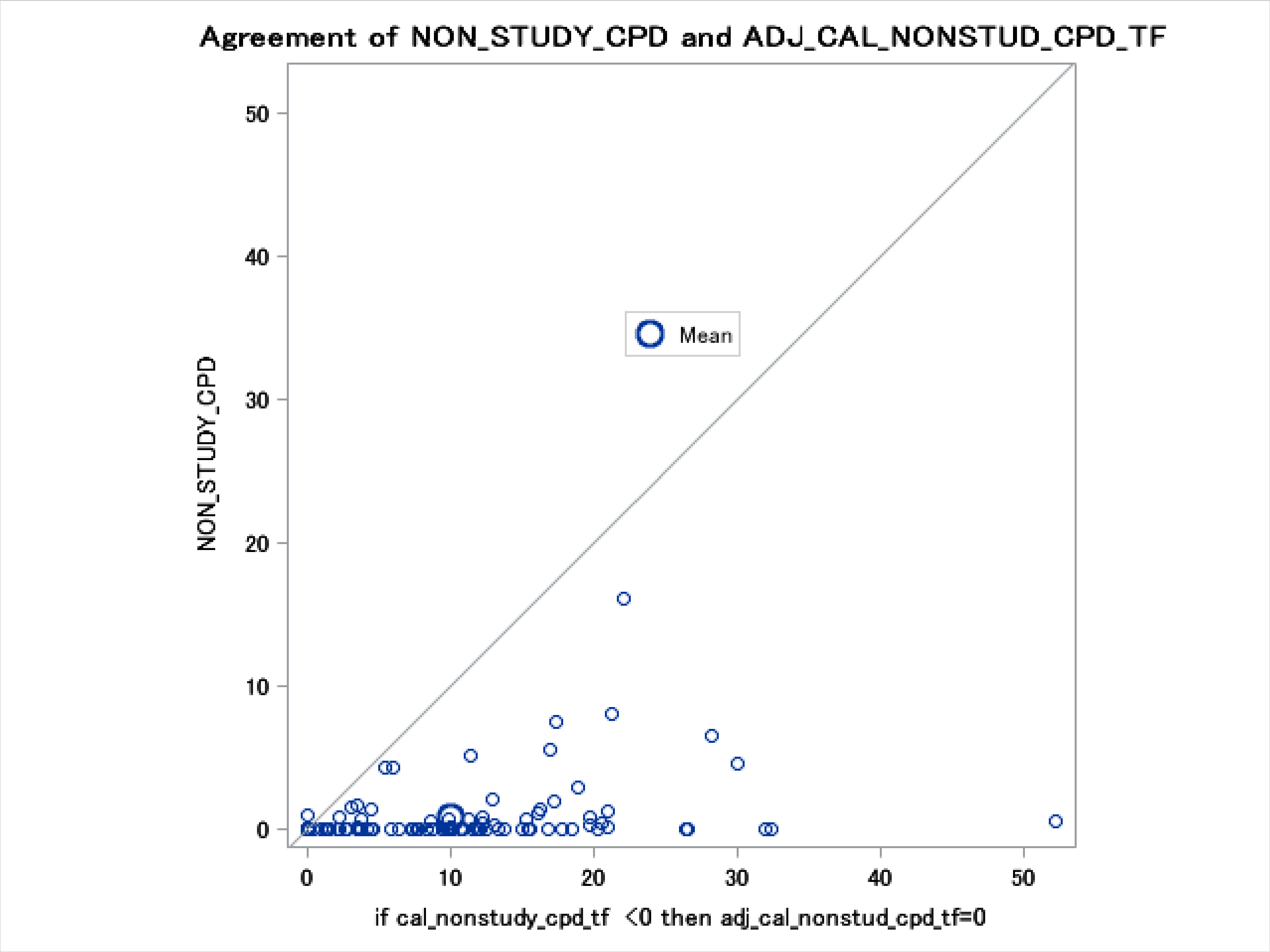

Agreement of NON_STUDY_CPD and ADJ_CAL_NONSTUD_CPD_TF
NON_STUDY_CPD
0
10
20
30
40
50
Mean
0
10
20
30
40
50
if cal_nonstudy_cpd_tf <0 then adj_cal_nonstud_cpd_tf=0

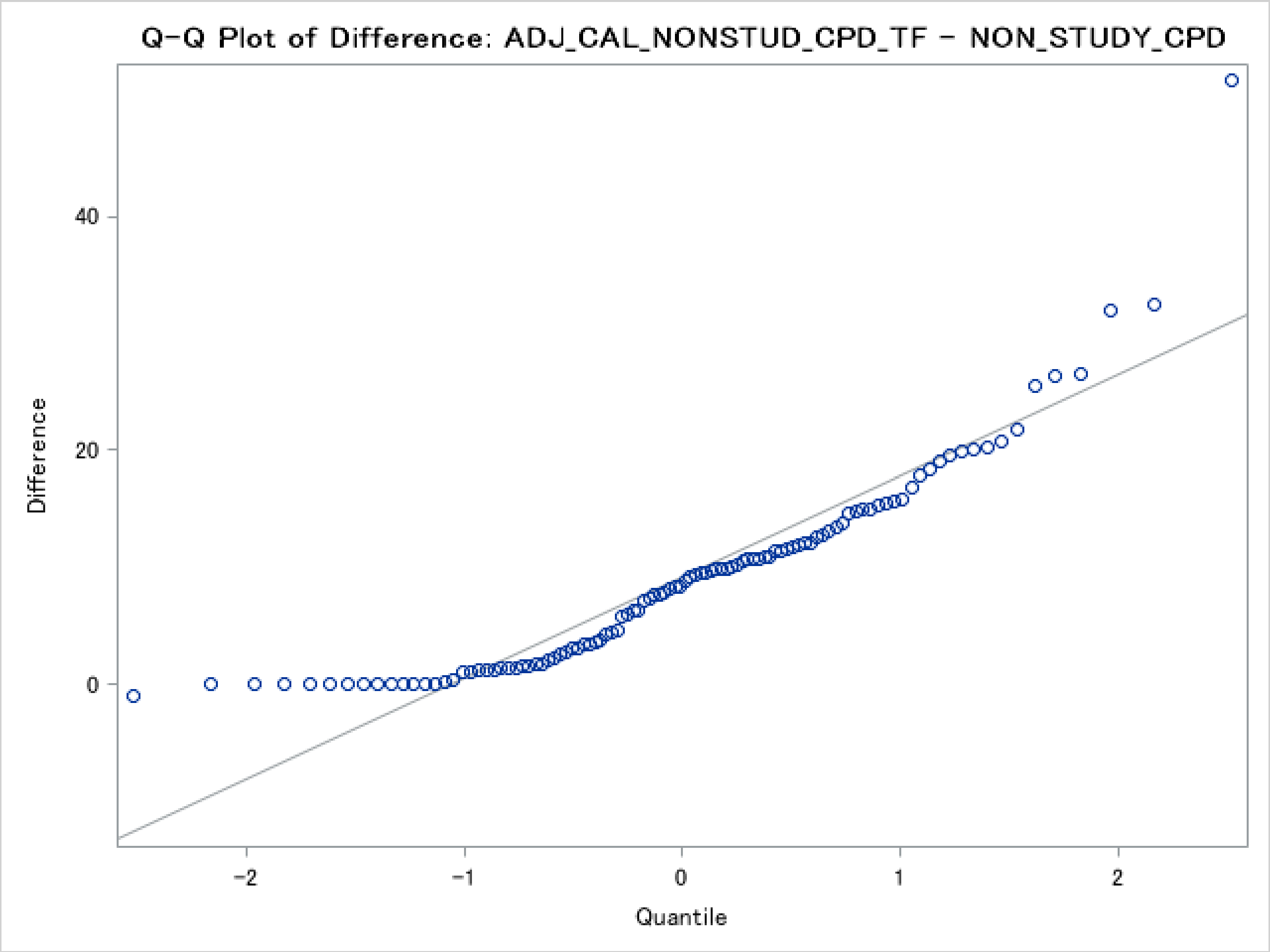
Q-Q Plot of Difference: ADJ_CAL_NONSTUD_CPD_TF - NON_STUDY_CPD
Difference
40
20
0
−2
−1
0
1
2
Quantile

Paired t-test on raw self-reported and estimated non-study CPD

The TTEST Procedure

Difference: ADJ_CAL_NONSTUD_CPD_TF - NON_STUDY_CPD
AVISIT=week 6 TRTA=G ARM=0.4 mg/g

N	Mean	Std Dev	Std Err	Minimum	Maximum
104	13.9148	13.4955	1.3233	-2.8098	90.1867

Mean	95% CL Mean		Std Dev	95% CL Std Dev	
13.9148	11.2903	16.5394	13.4955	11.8774	15.6279

DF	t Value	Pr > |t|
103	10.51	<.0001

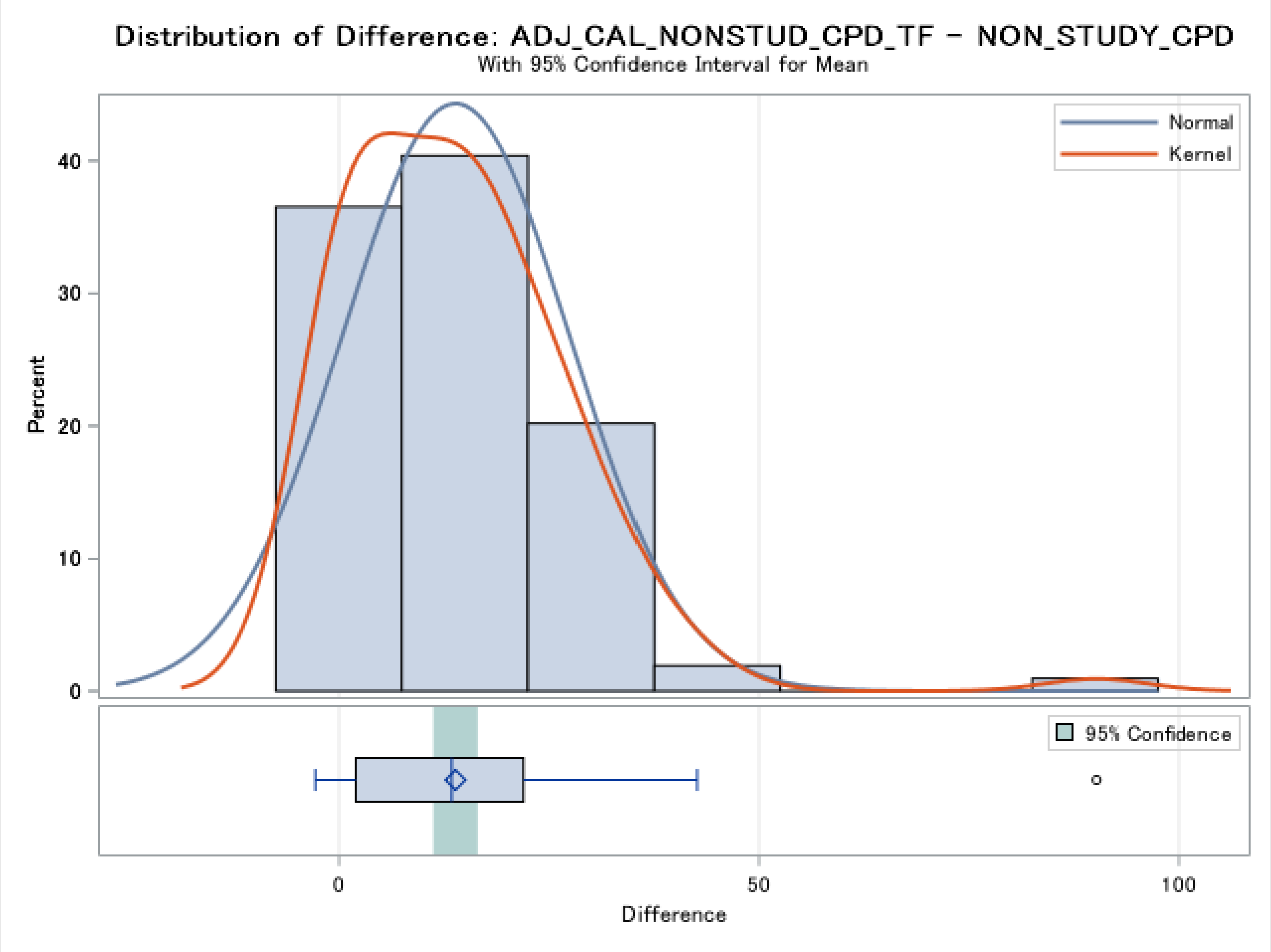

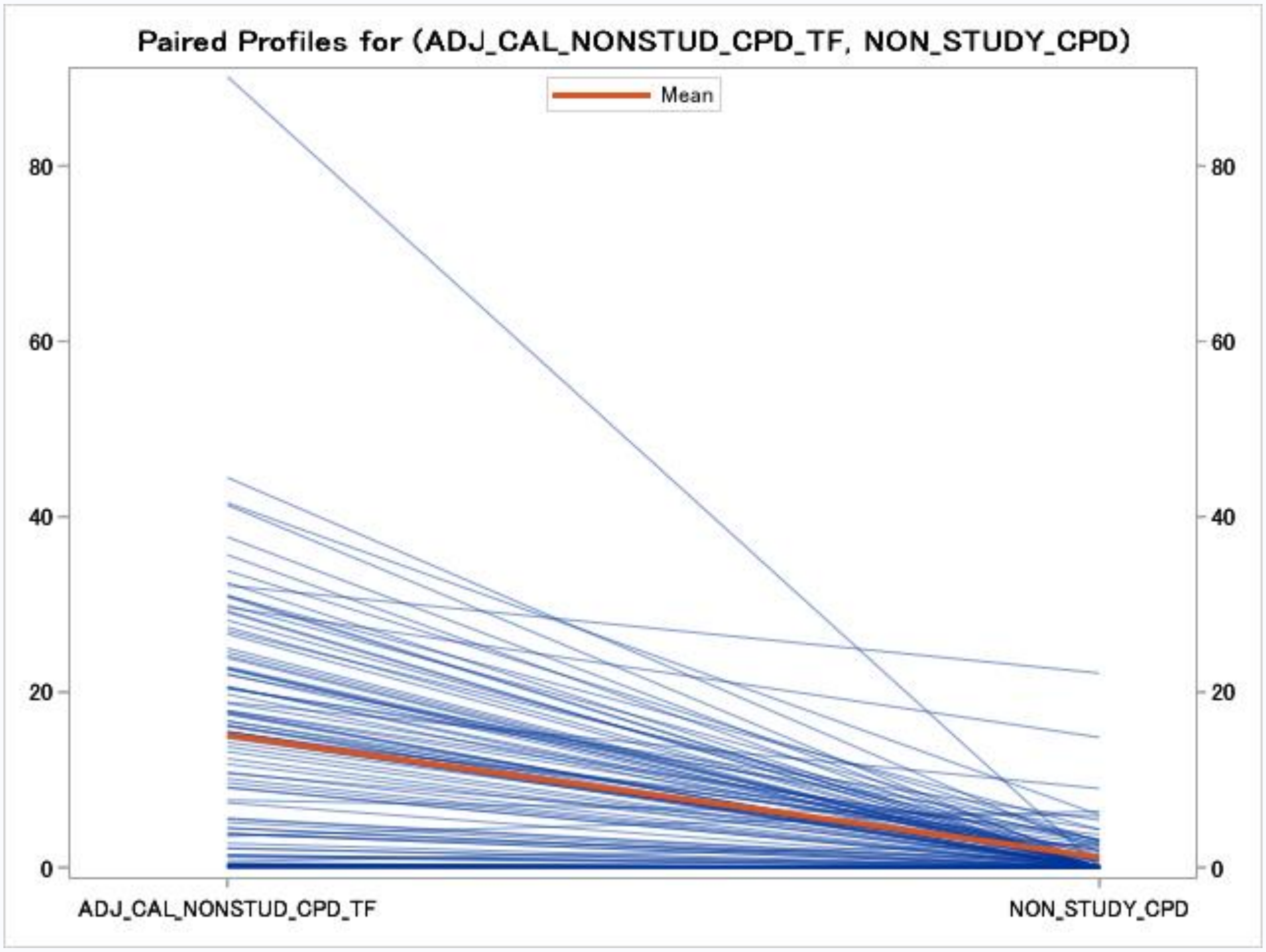
Paired Profiles for (ADJ_CAL_NONSTUD_CPD_TF, NON_STUDY_CPD)
Mean
80
60
40
20
0
ADJ_CAL_NONSTUD_CPD_TF
NON_STUDY_CPD

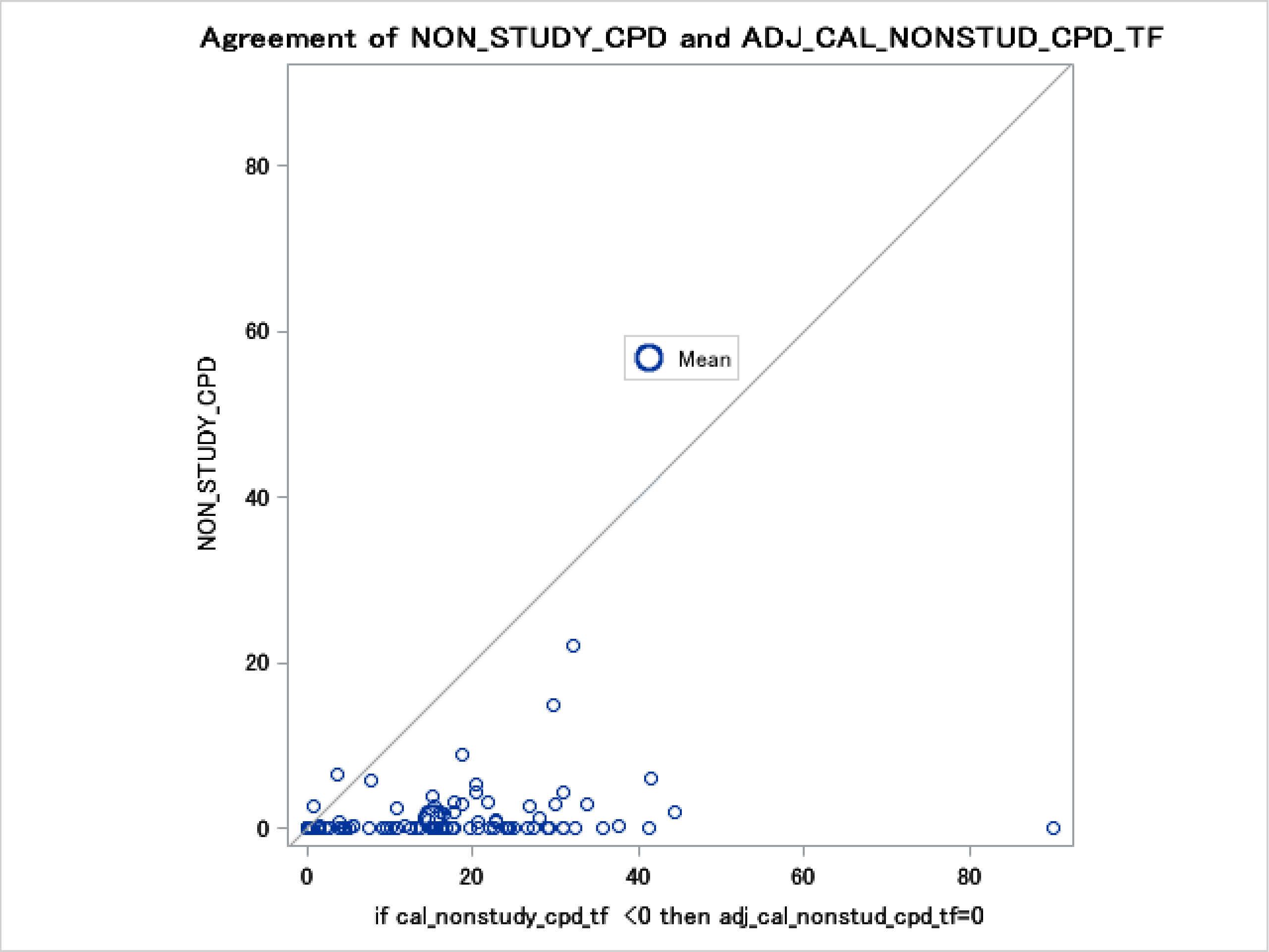
Agreement of NON_STUDY_CPD and ADJ_CAL_NONSTUD_CPD_TF
NON_STUDY_CPD
80
60
40
20
0
Mean
0
20
40
60
80
if cal_nonstudy_cpd_tf <0 then adj_cal_nonstud_cpd_tf=0

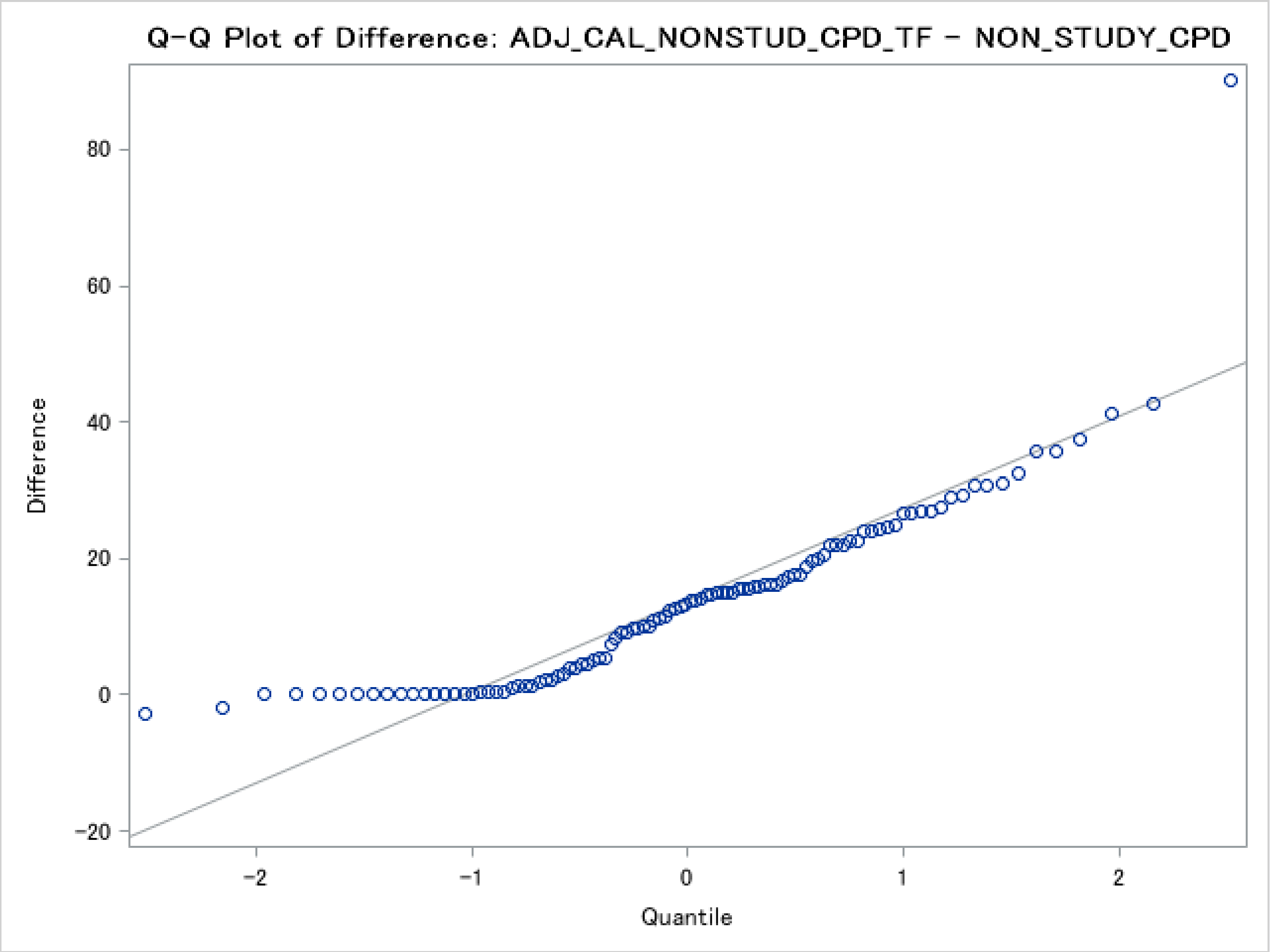

Q-Q Plot of Difference: ADJ_CAL_NONSTUD_CPD_TF - NON_STUDY_CPD
Difference
80
60
40
20
0
-20
-2
-1
0
1
2
Quantile

t-test on variable DIFF

The TTEST Procedure

Variable: diff
AVISIT=week 2 TRTA=A ARM=2.4 mg/g

N	Mean	Std Dev	Std Err	Minimum	Maximum
110	8.8462	9.4409	0.9002	-2.8333	49.9986

Mean	95% CL Mean		Std Dev	95% CL Std Dev	
8.8462	7.0621	10.6303	9.4409	8.3368	10.8848

DF	t Value	Pr > |t|
109	9.83	<.0001

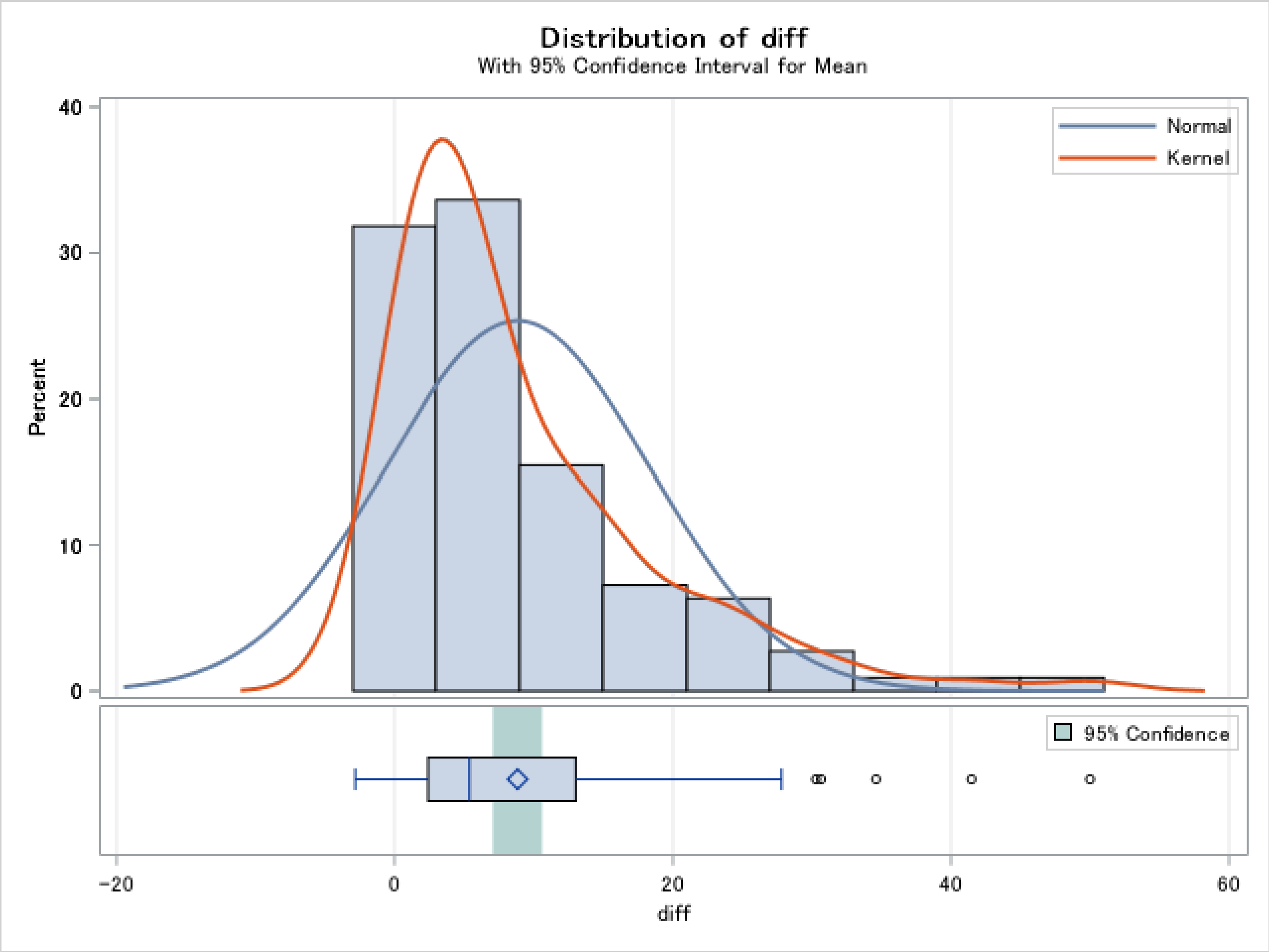

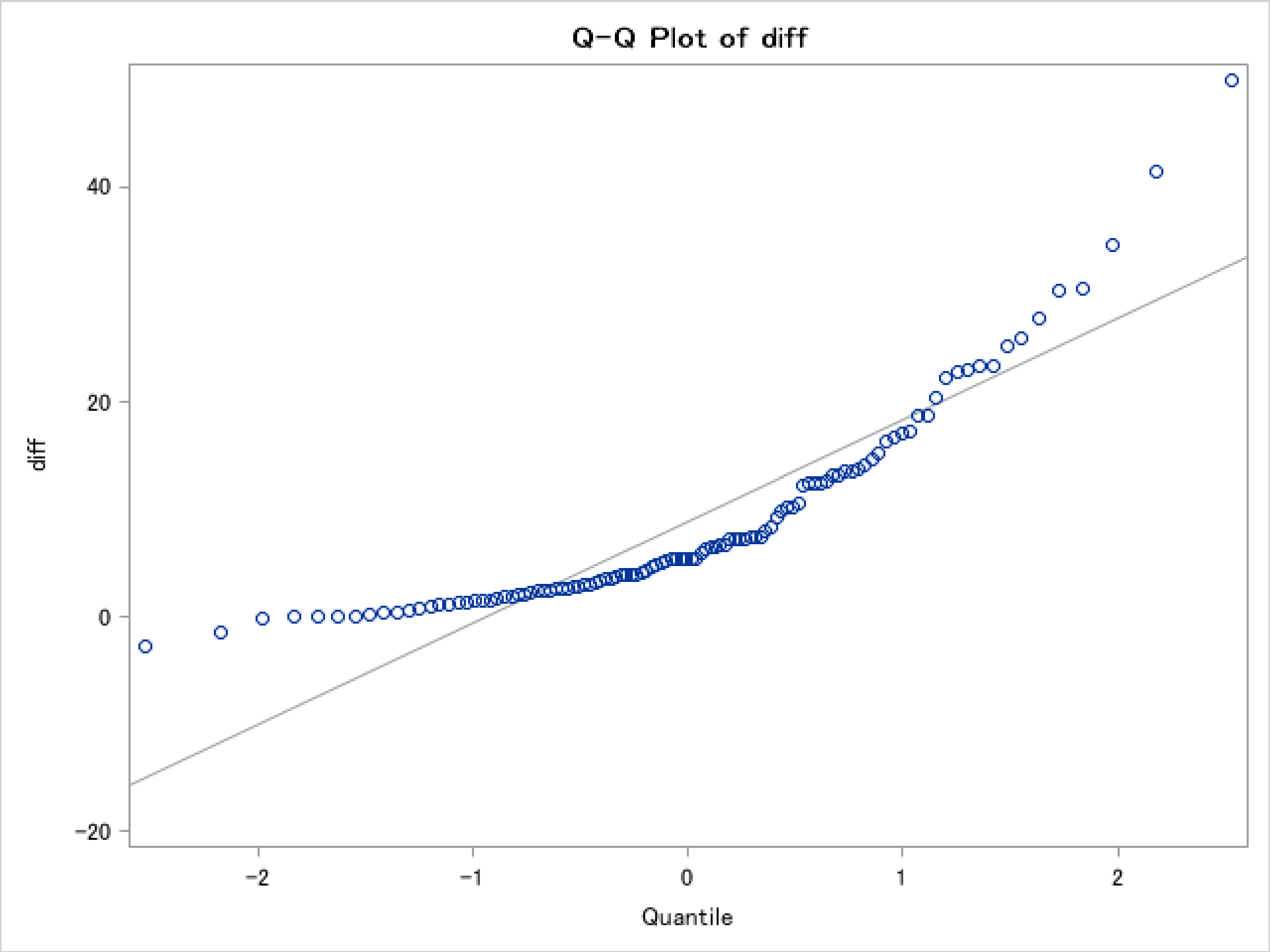
Q-Q Plot of diff
diff
40
20
0
-20
-2
-1
0
1
2
Quantile

t-test on variable DIFF

The TTEST Procedure

Variable: diff

AVISIT=week 2 TRTA=B ARM=0.4 mg/g (HT)

N	Mean	Std Dev	Std Err	Minimum	Maximum
115	9.9769	12.7414	1.1881	-5.5536	82.3765

Mean	95% CL Mean		Std Dev	95% CL Std Dev	
9.9769	7.6232	12.3306	12.7414	11.2804	14.6405

DF	t Value	Pr > |t|
114	8.40	<.0001

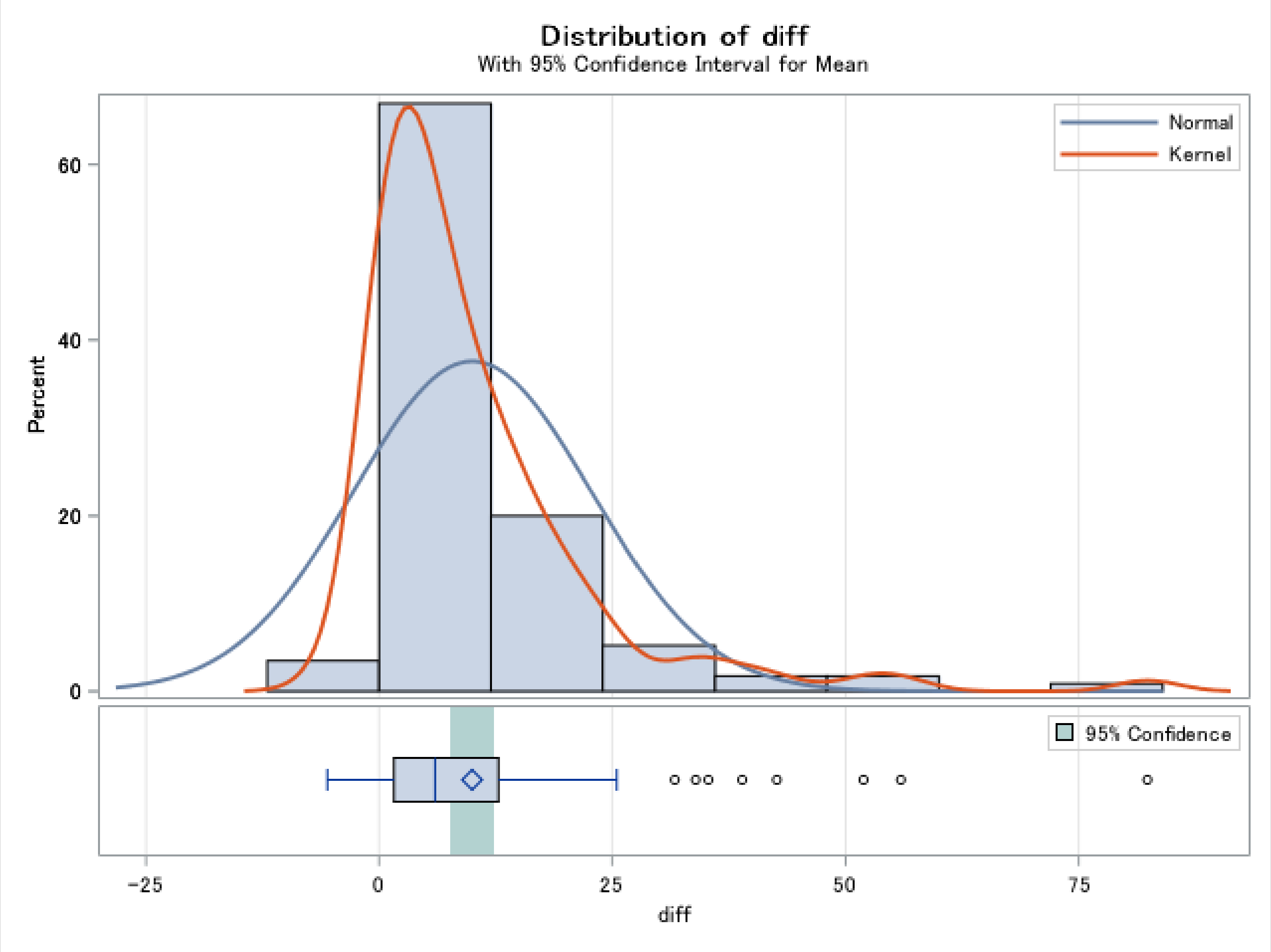

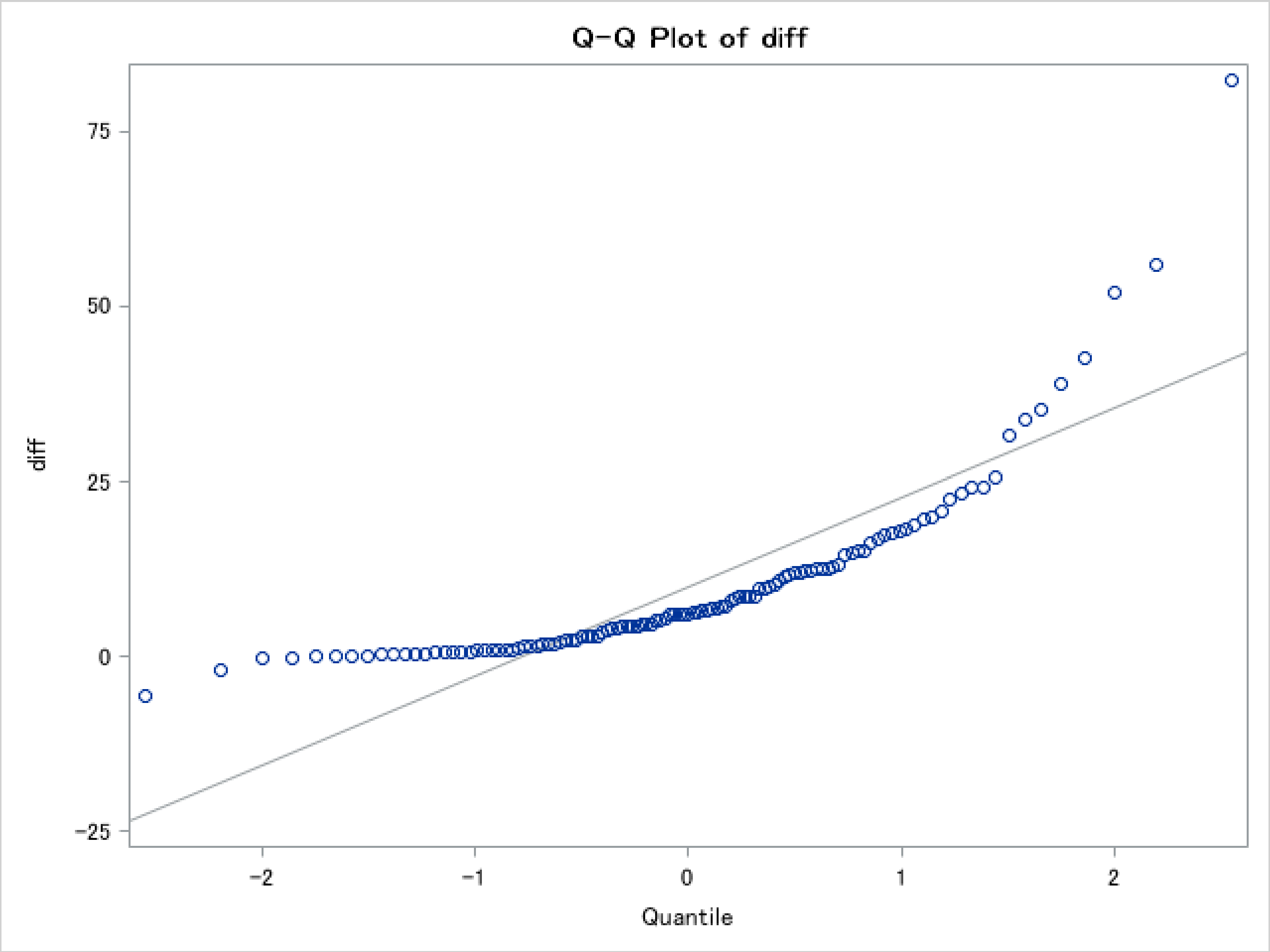

Q-Q Plot of diff
diff
75
50
25
0
-25
-2
-1
0
1
2
Quantile

t-test on variable DIFF

The TTEST Procedure

Variable: diff

AVISIT=week 2 TRTA=D ARM=1.3 mg/g

N	Mean	Std Dev	Std Err	Minimum	Maximum
110	11.5813	13.9832	1.3332	-8.3542	74.3285

Mean	95% CL Mean		Std Dev	95% CL Std Dev	
11.5813	8.9389	14.2238	13.9832	12.3478	16.1218

DF	t Value	Pr > |t|
109	8.69	<.0001

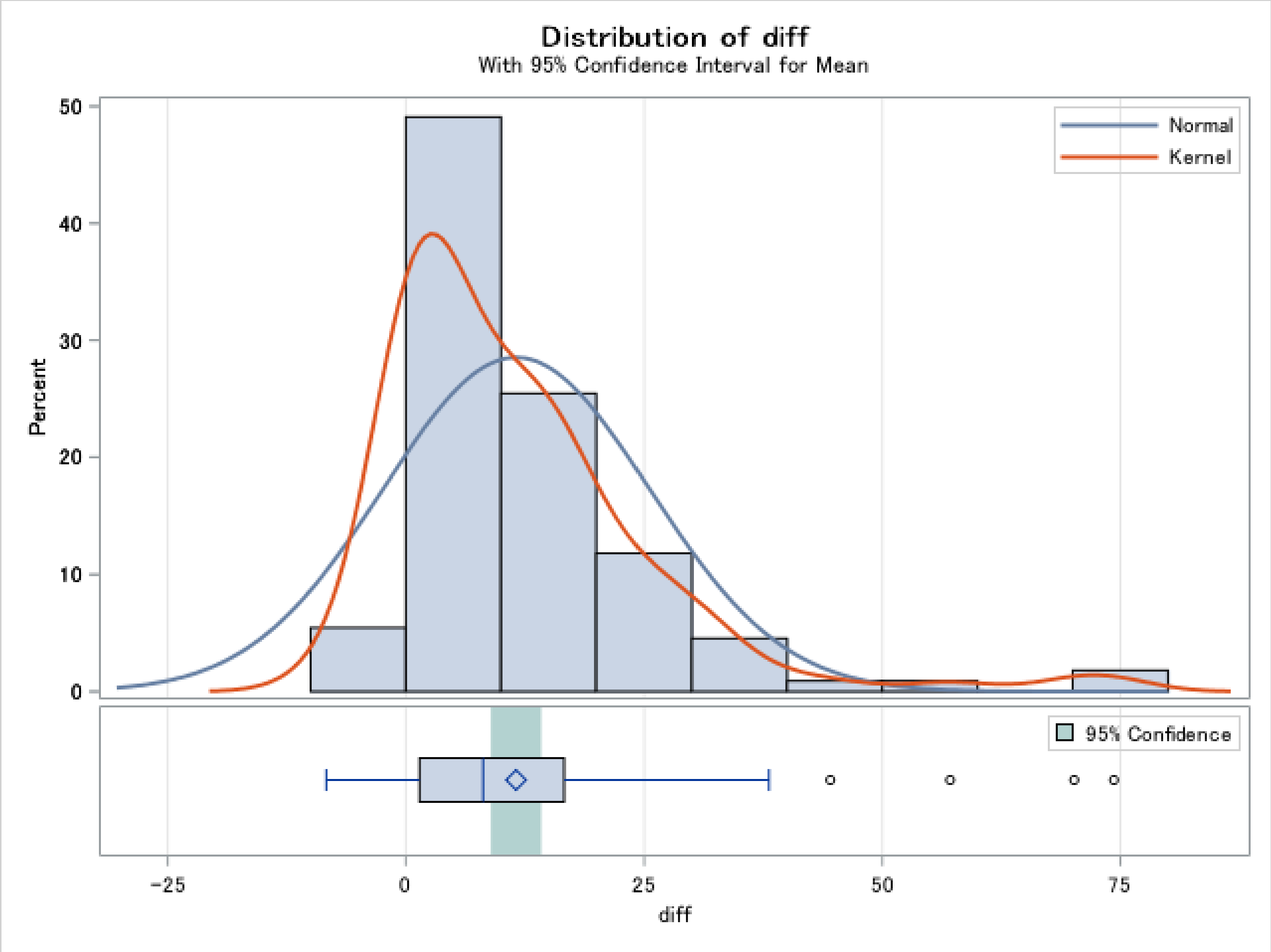

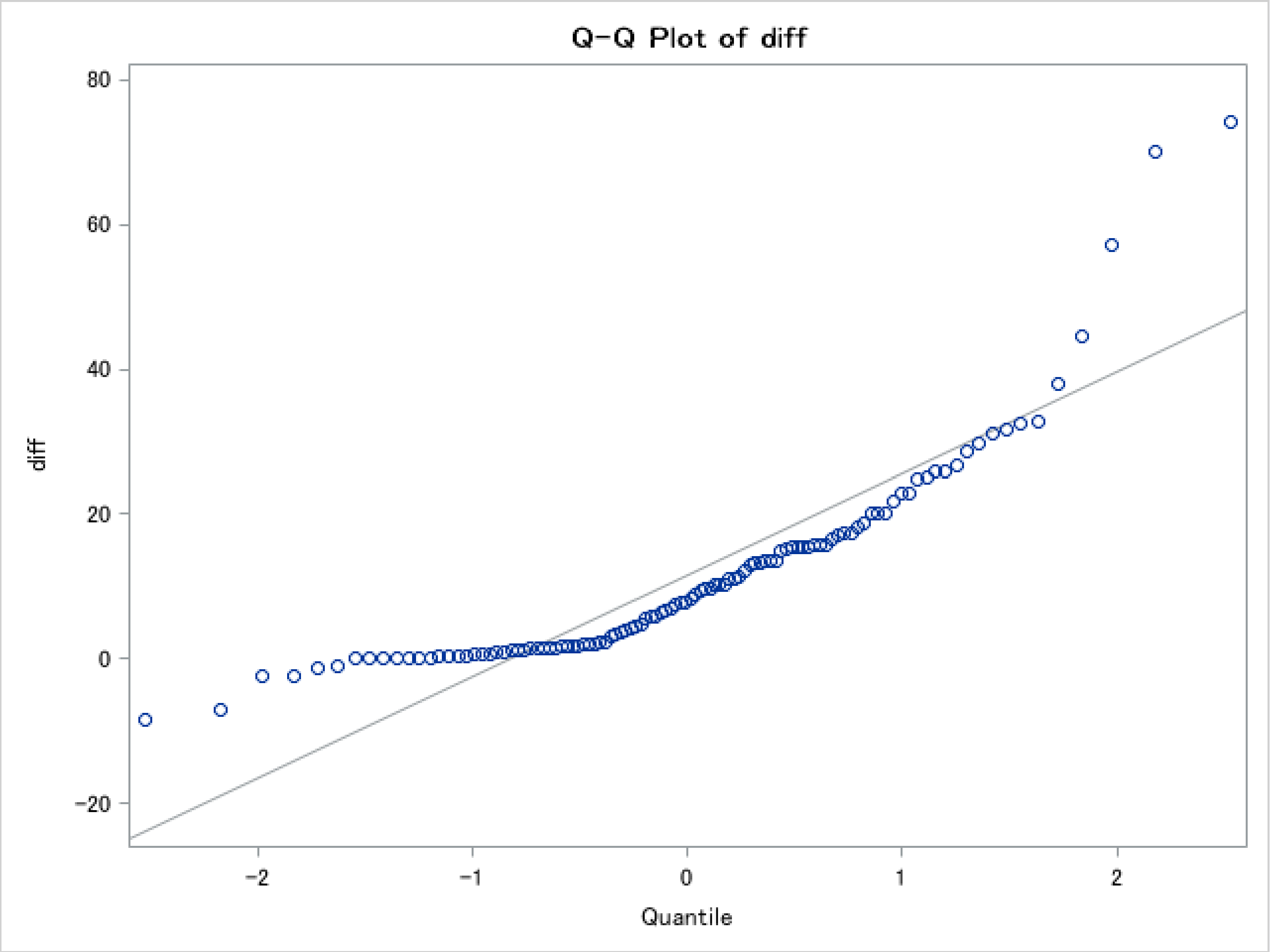

Q-Q Plot of diff
80
60
40
20
0
-20
diff
-2
-1
0
1
2
Quantile

t-test on variable DIFF

The TTEST Procedure

Variable: diff

AVISIT=week 2 TRTA=F ARM=5.2 mg/g

N	Mean	Std Dev	Std Err	Minimum	Maximum
110	9.5648	24.9733	2.3811	-3.6667	252.4

Mean	95% CL Mean		Std Dev	95% CL Std Dev	
9.5648	4.8455	14.2841	24.9733	22.0526	28.7927

DF	t Value	Pr > |t|
109	4.02	0.0001

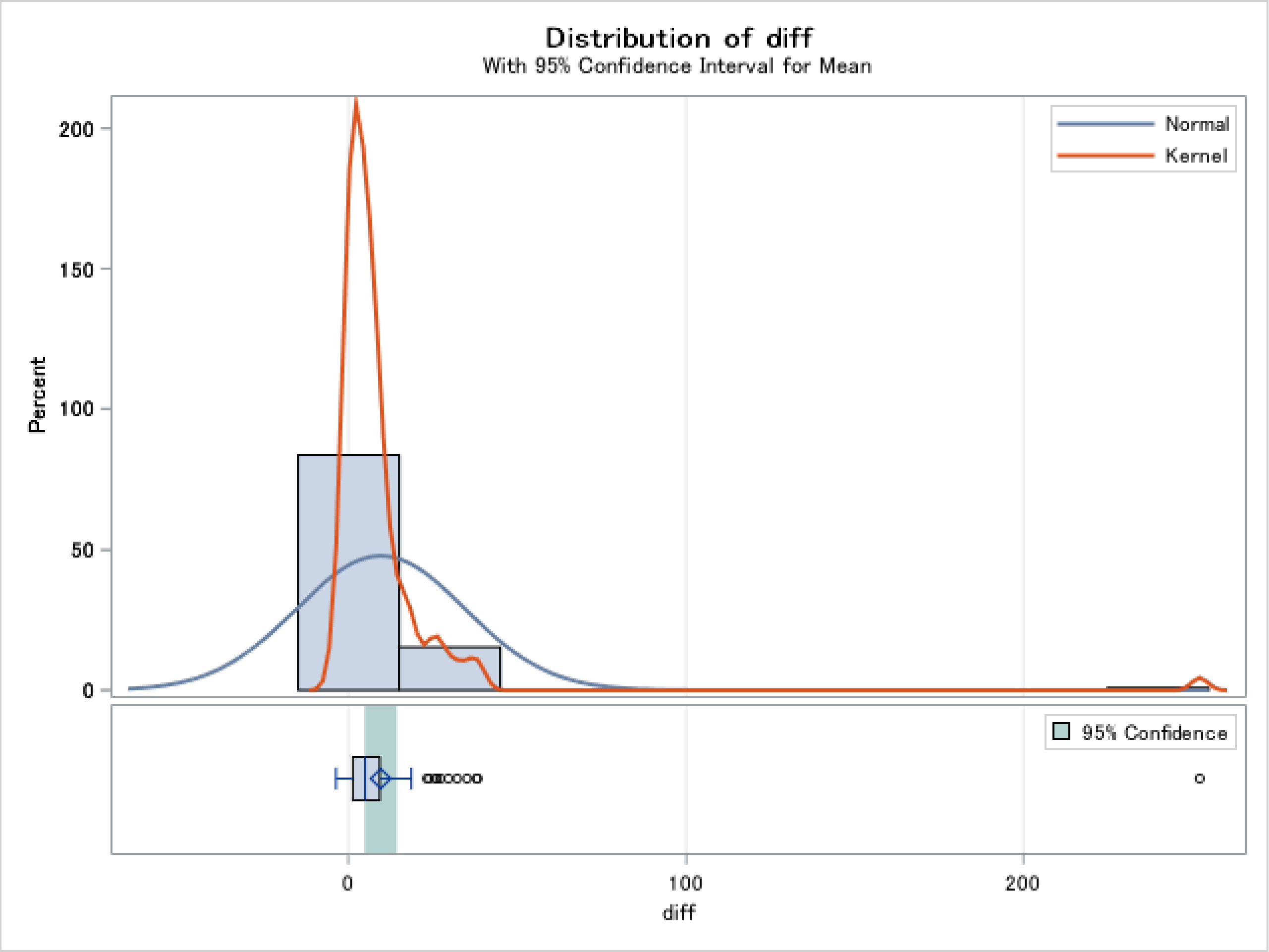

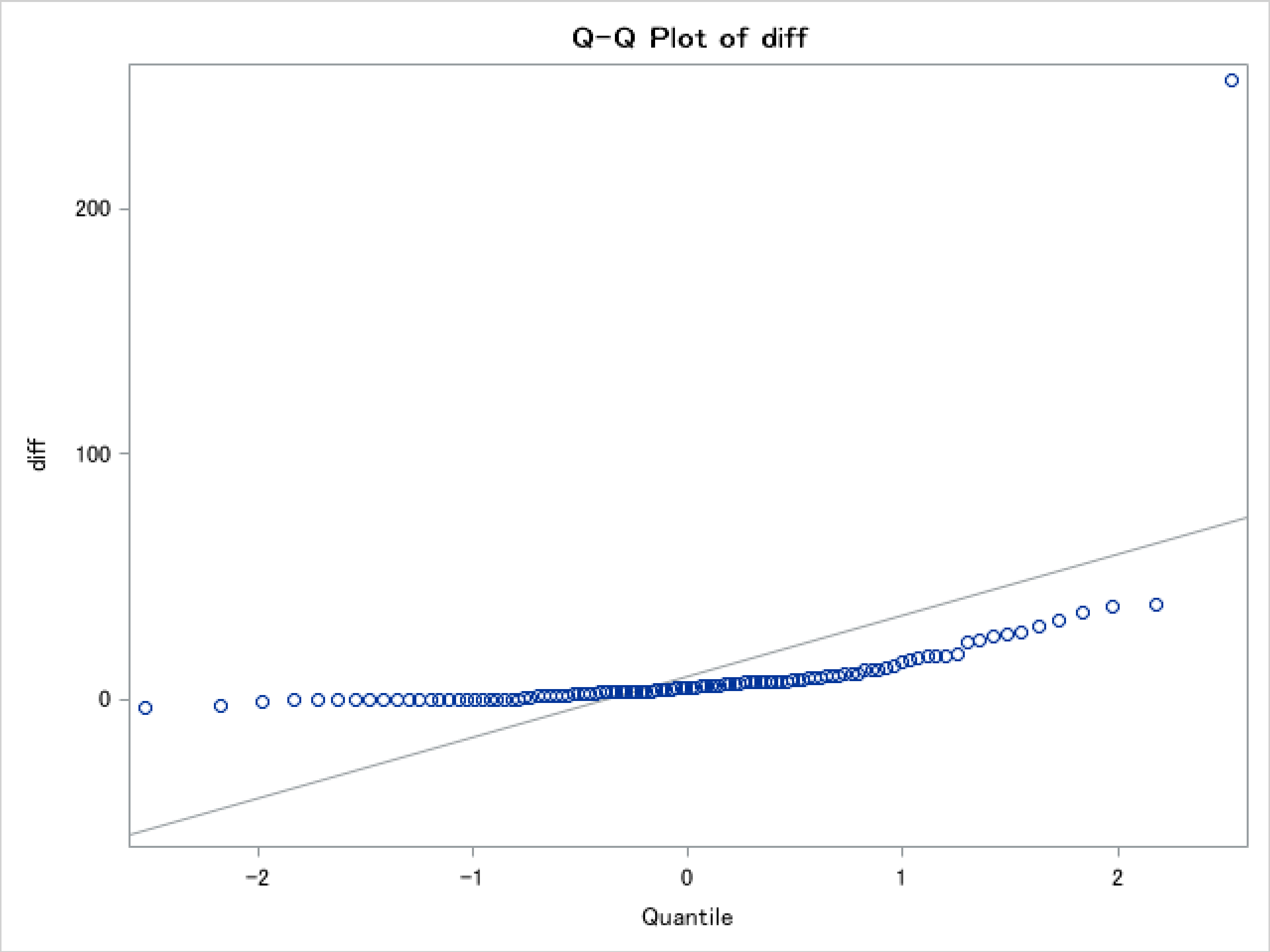

Q-Q Plot of diff
diff
200
100
0
−2
−1
0
1
2
Quantile

t-test on variable DIFF

The TTEST Procedure

Variable: diff
AVISIT=week 2 TRTA=G ARM=0.4 mg/g

N	Mean	Std Dev	Std Err	Minimum	Maximum
111	14.6297	21.4020	2.0314	-3.1607	142.1

Mean	95% CL Mean		Std Dev	95% CL Std Dev	
14.6297	10.6040	18.6554	21.4020	18.9090	24.6581

DF	t Value	Pr > |t|
110	7.20	<.0001

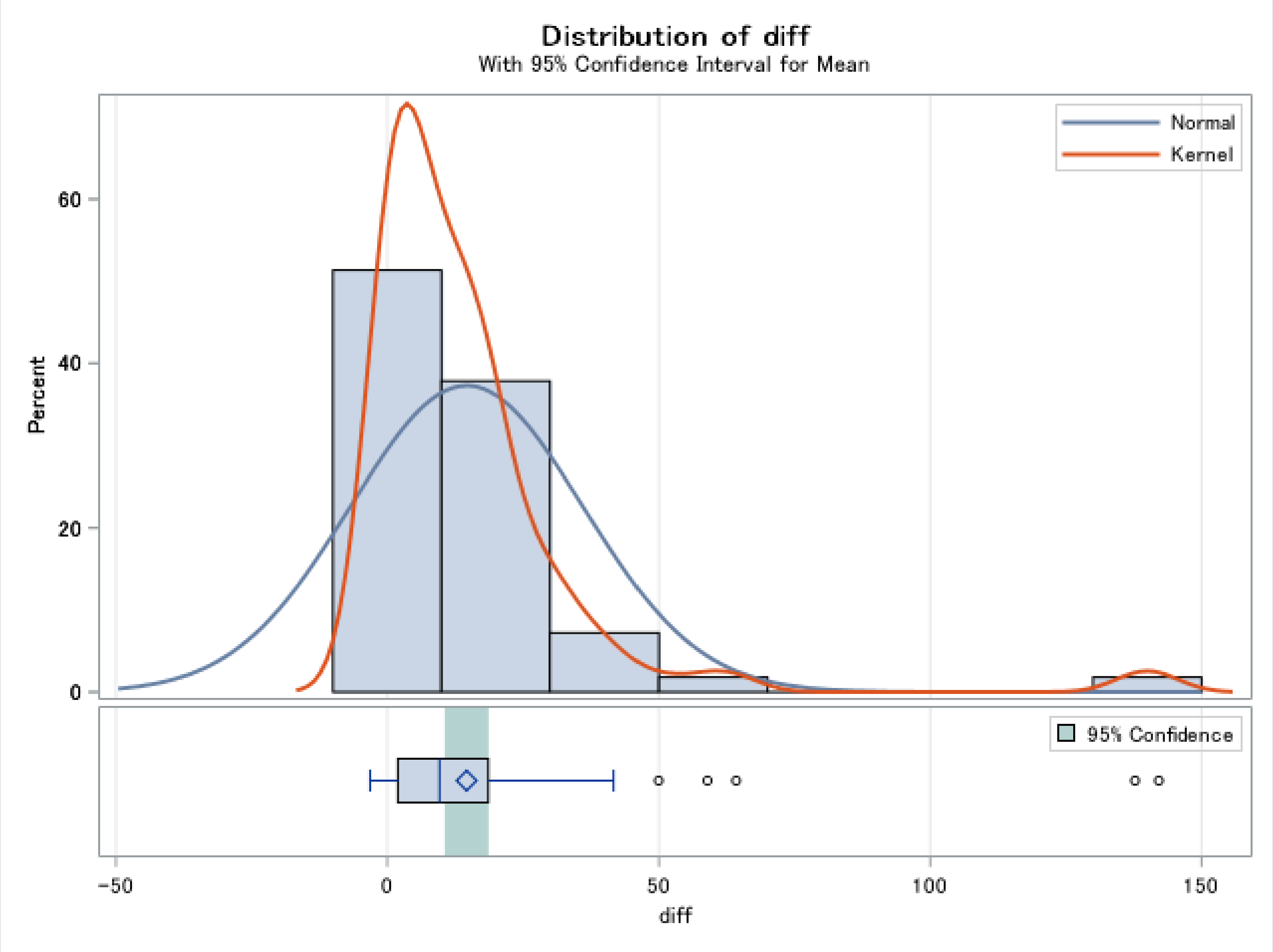

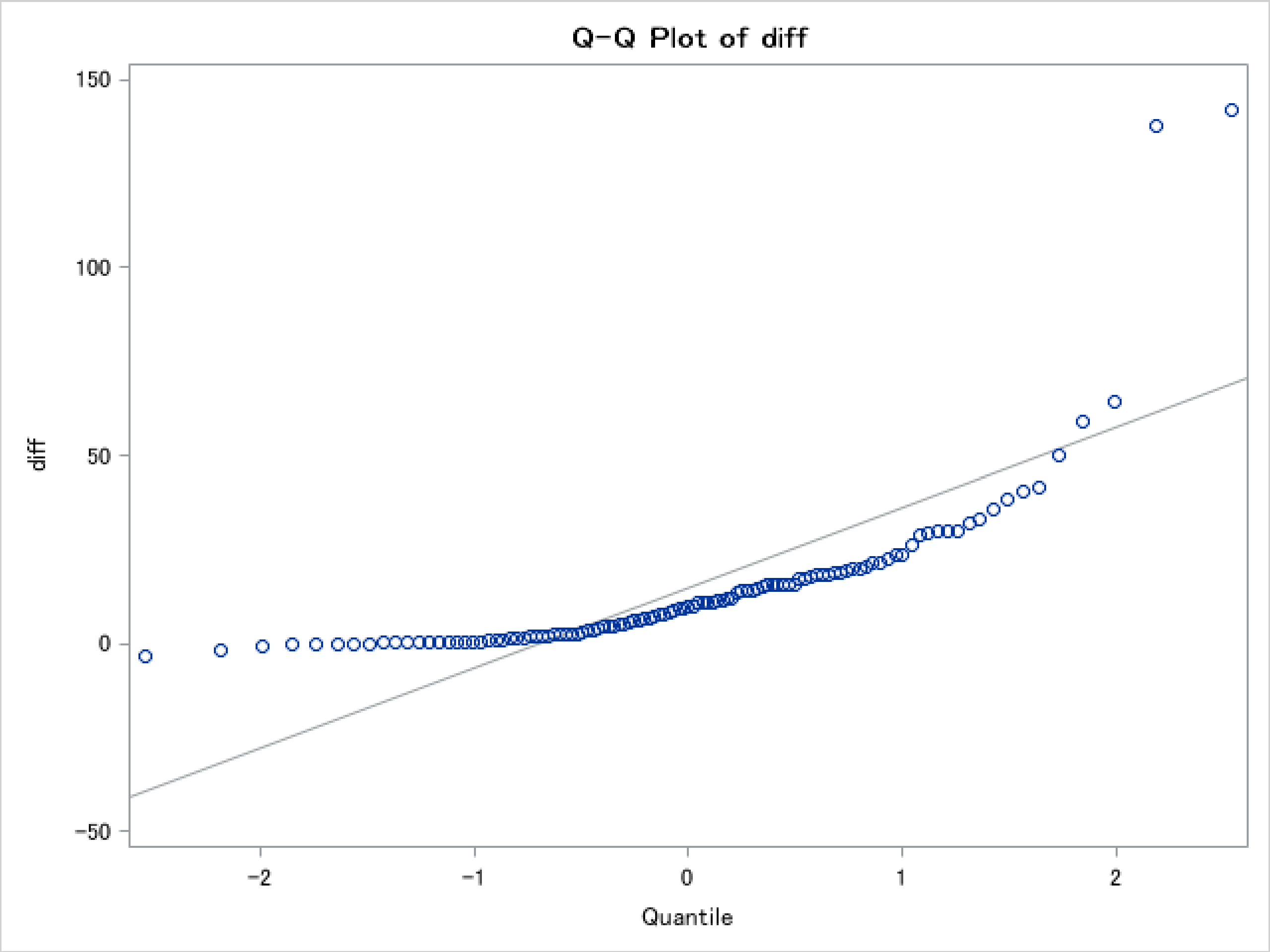
Q-Q Plot of diff
150
100
50
0
-50
diff
-2
-1
0
1
2
Quantile

t-test on variable DIFF

The TTEST Procedure

Variable: diff

AVISIT=week 6 TRTA=A ARM=2.4 mg/g

N	Mean	Std Dev	Std Err	Minimum	Maximum
106	10.8919	10.8017	1.0492	-6.5301	48.3089

Mean	95% CL Mean		Std Dev	95% CL Std Dev	
10.8919	8.8117	12.9722	10.8017	9.5175	12.4897

DF	t Value	Pr > |t|
105	10.38	<.0001

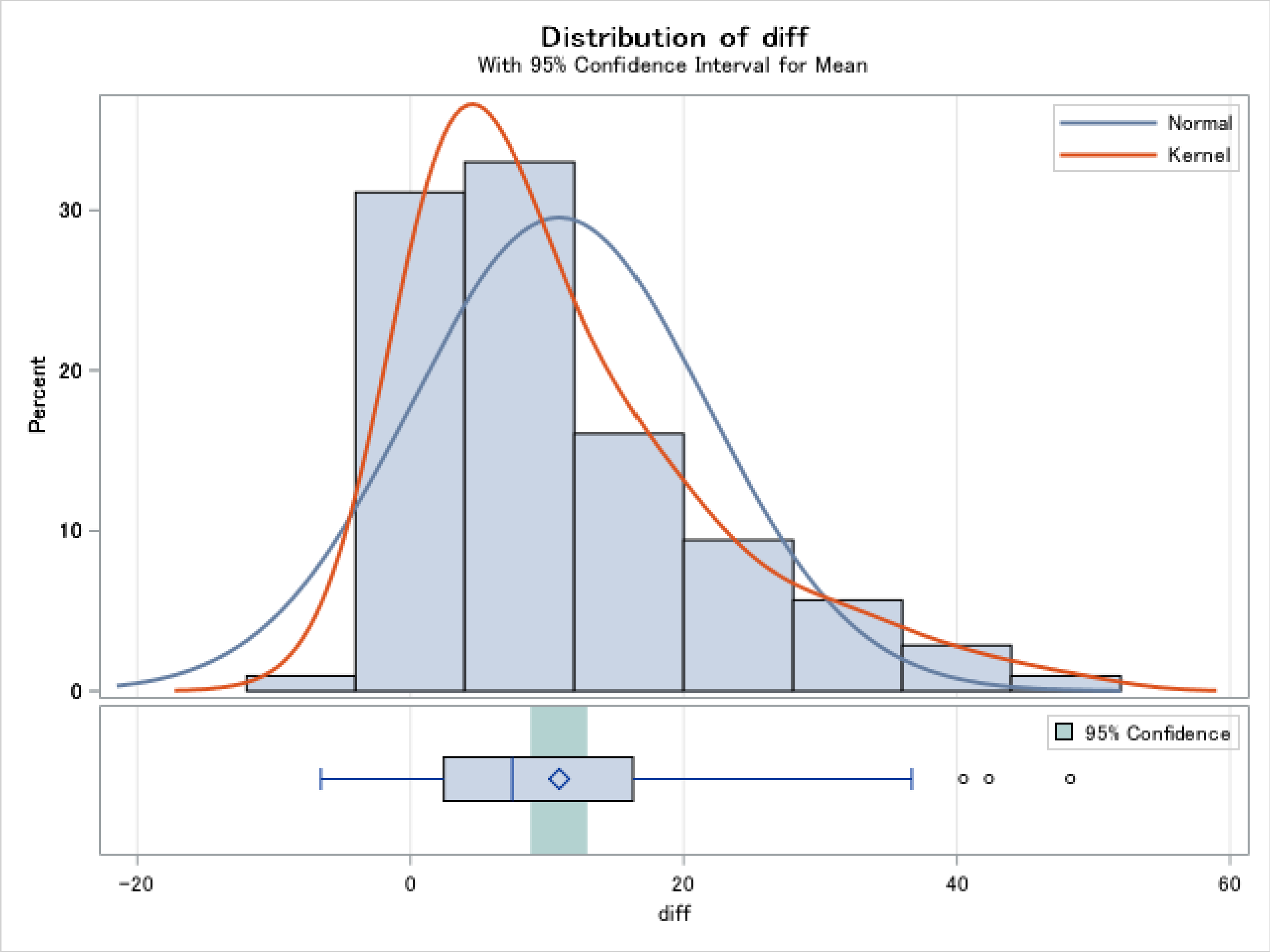

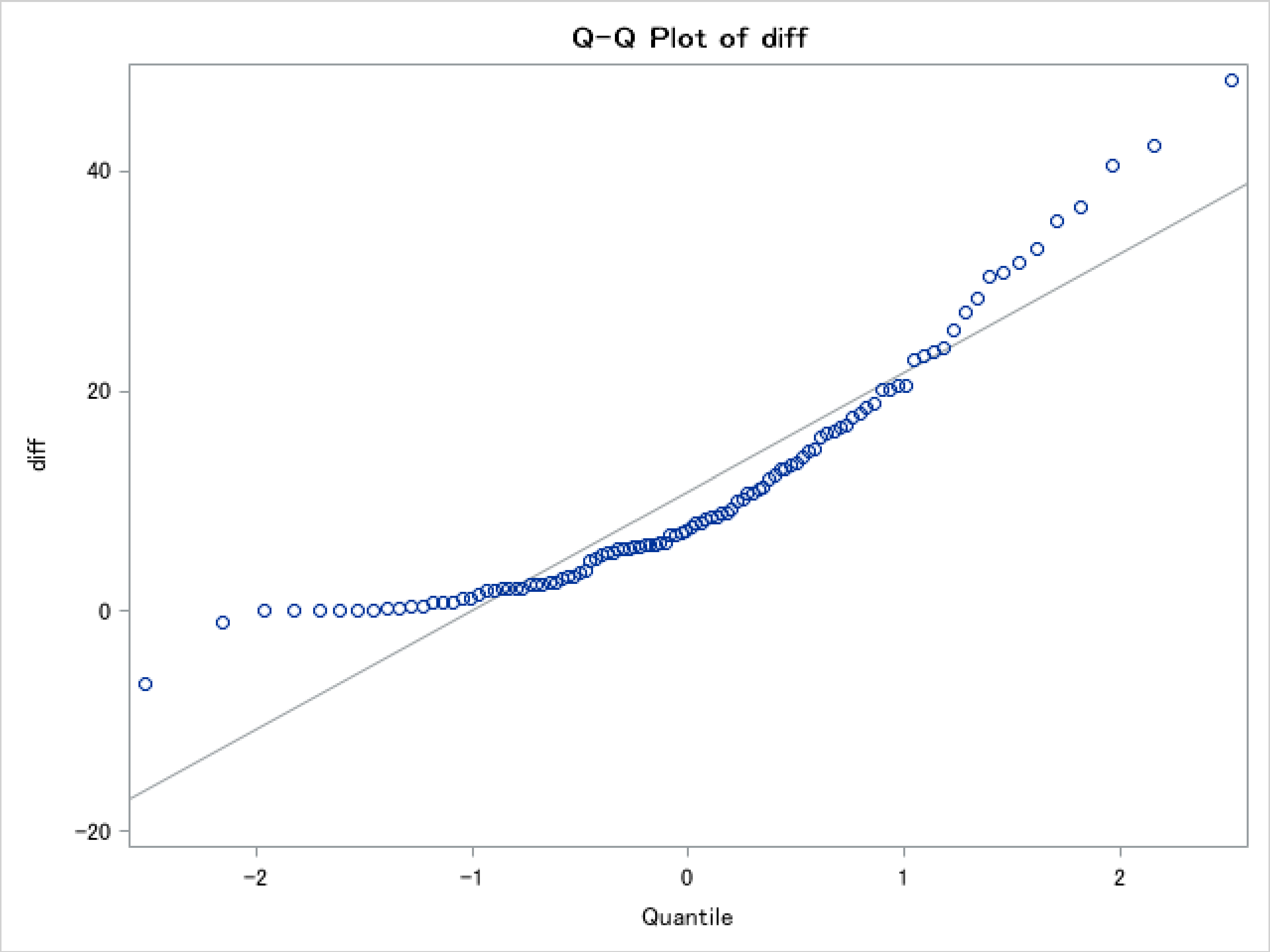
Q-Q Plot of diff
diff
40
20
0
-20
-2
-1
0
1
2
Quantile

t-test on variable DIFF

The TTEST Procedure

Variable: diff
AVISIT=week 6 TRTA=B ARM=0.4 mg/g (HT)

N	Mean	Std Dev	Std Err	Minimum	Maximum
113	11.1654	11.4981	1.0816	-3.7350	43.9397

Mean	95% CL Mean		Std Dev	95% CL Std Dev	
11.1654	9.0223	13.3086	11.4981	10.1693	13.2294

DF	t Value	Pr > |t|
112	10.32	<.0001

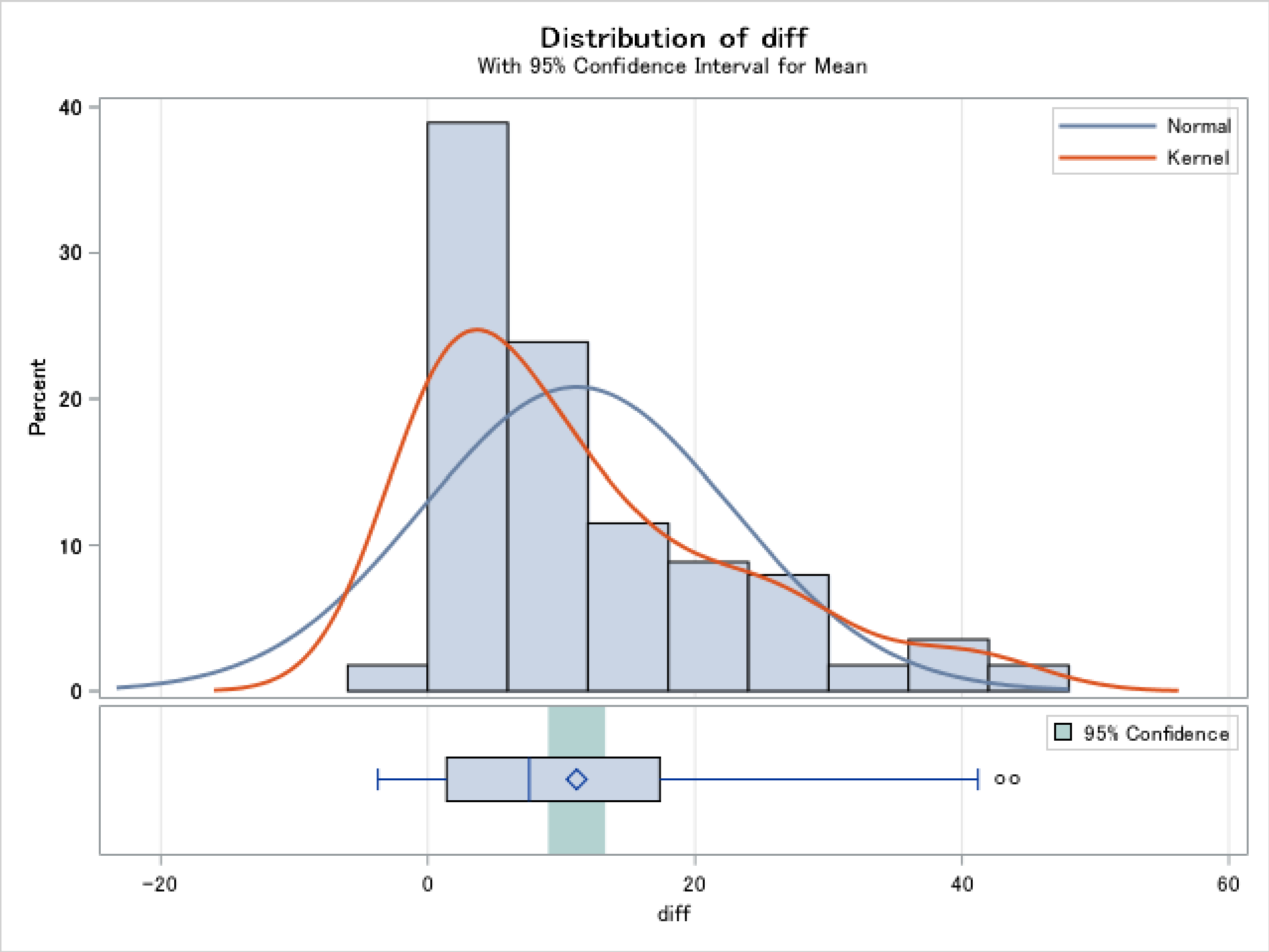

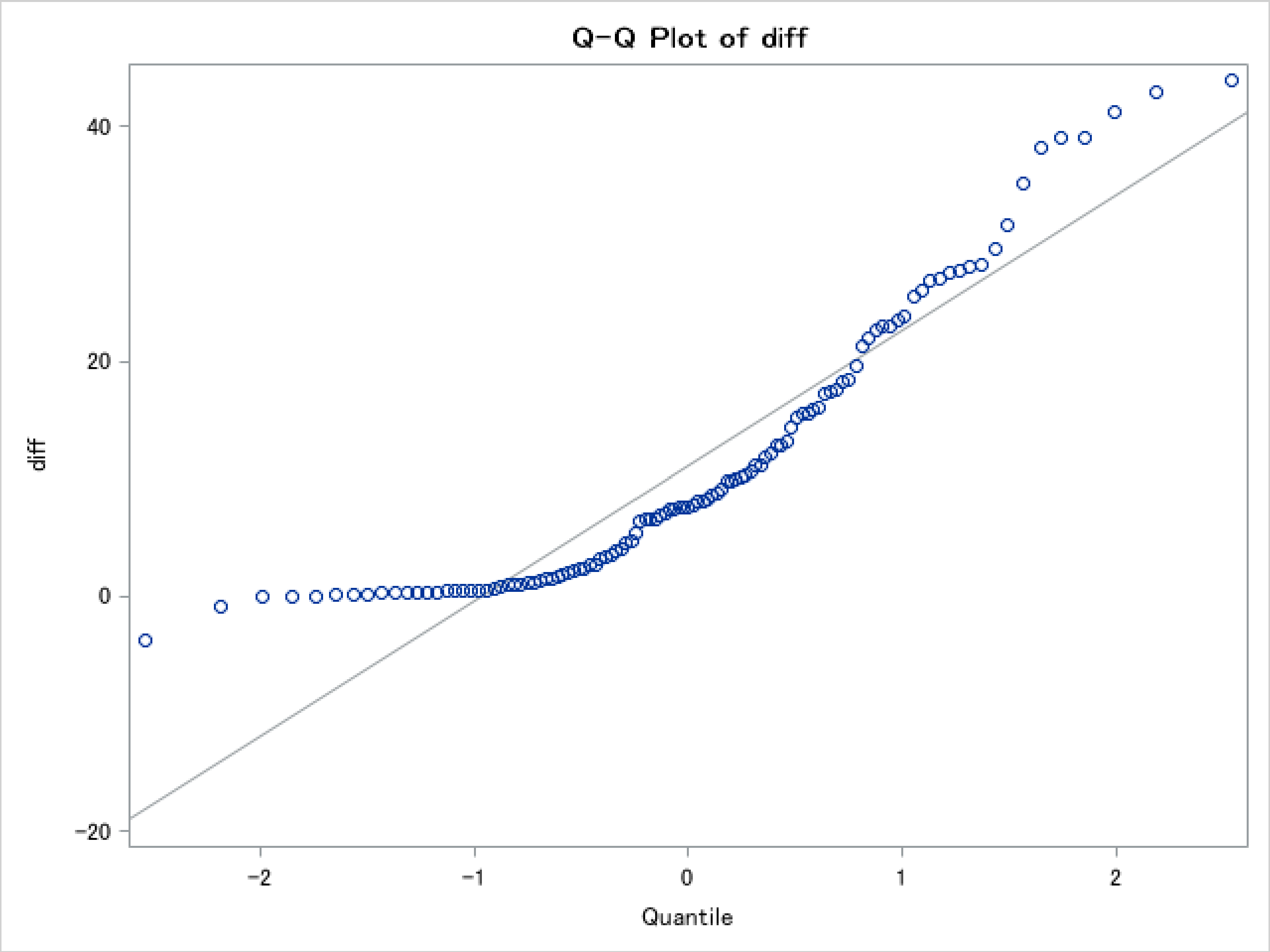

Q-Q Plot of diff
40
20
0
−20
diff
−2
−1
0
1
2
Quantile

t-test on variable DIFF

The TTEST Procedure

Variable: diff

AVISIT=week 6 TRTA=D ARM=1.3 mg/g

N	Mean	Std Dev	Std Err	Minimum	Maximum
106	13.6707	12.9025	1.2532	-0.5365	82.3516

Mean	95% CL Mean		Std Dev	95% CL Std Dev	
13.6707	11.1858	16.1556	12.9025	11.3685	14.9188

DF	t Value	Pr > |t|
105	10.91	<.0001

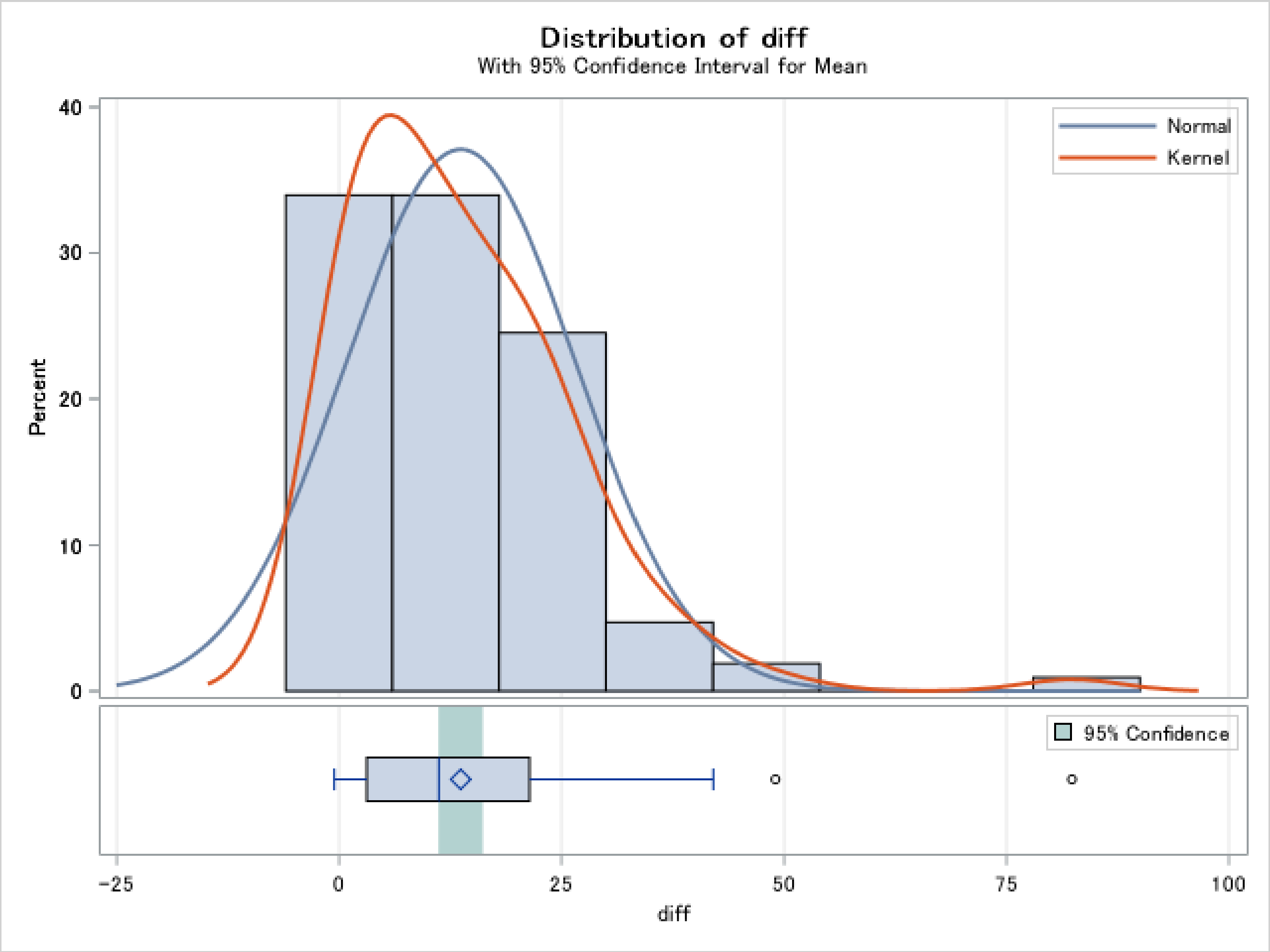

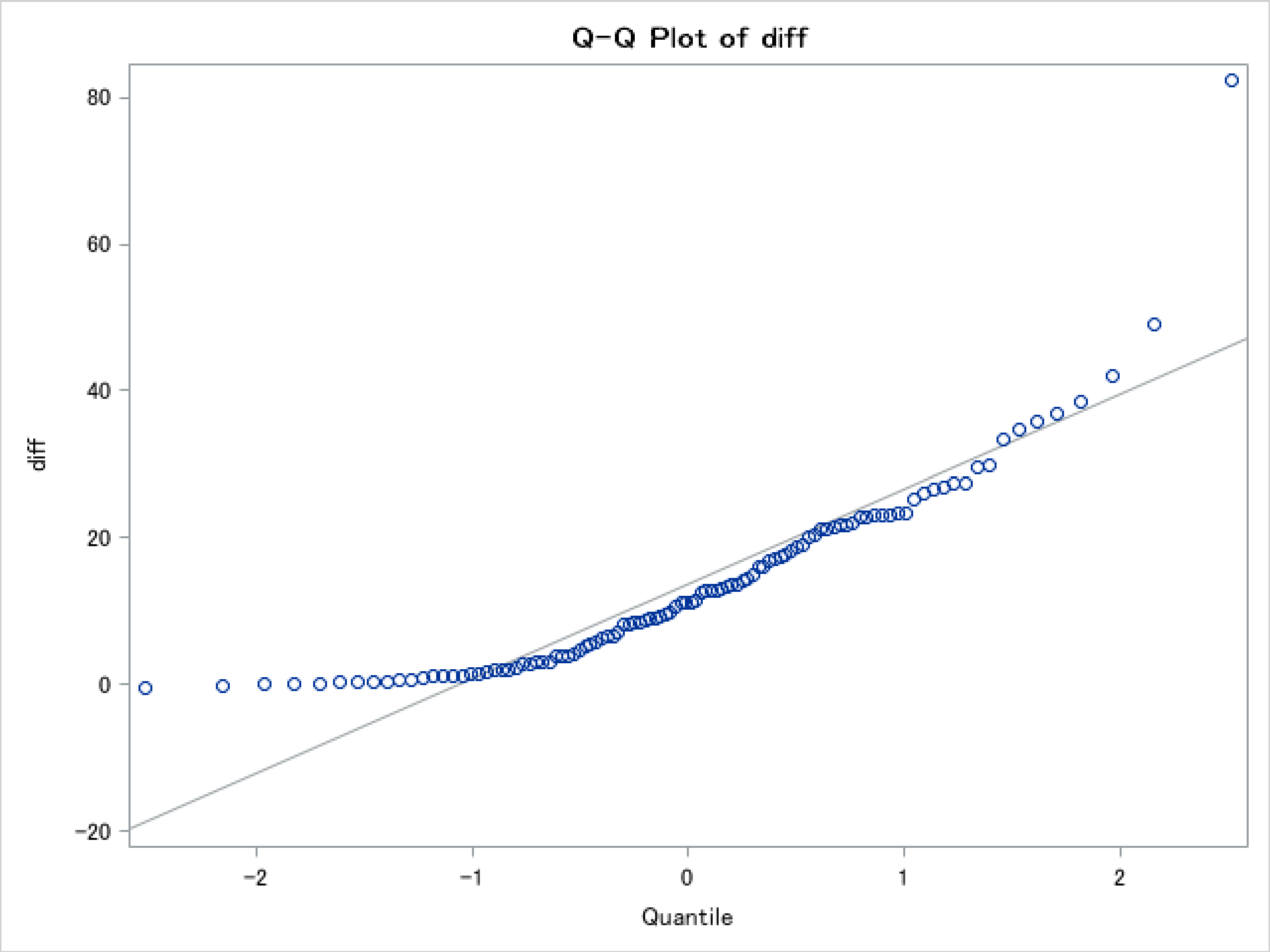
Q-Q Plot of diff
diff
Quantile
80
60
40
20
0
-20
-2
-1
0
1
2

t-test on variable DIFF

The TTEST Procedure

Variable: diff

AVISIT=week 6 TRTA=F ARM=5.2 mg/g

N	Mean	Std Dev	Std Err	Minimum	Maximum
106	9.2118	8.6354	0.8387	-1.0000	51.6750

Mean	95% CL Mean		Std Dev	95% CL Std Dev	
9.2118	7.5487	10.8748	8.6354	7.6087	9.9848

DF	t Value	Pr > |t|
105	10.98	<.0001

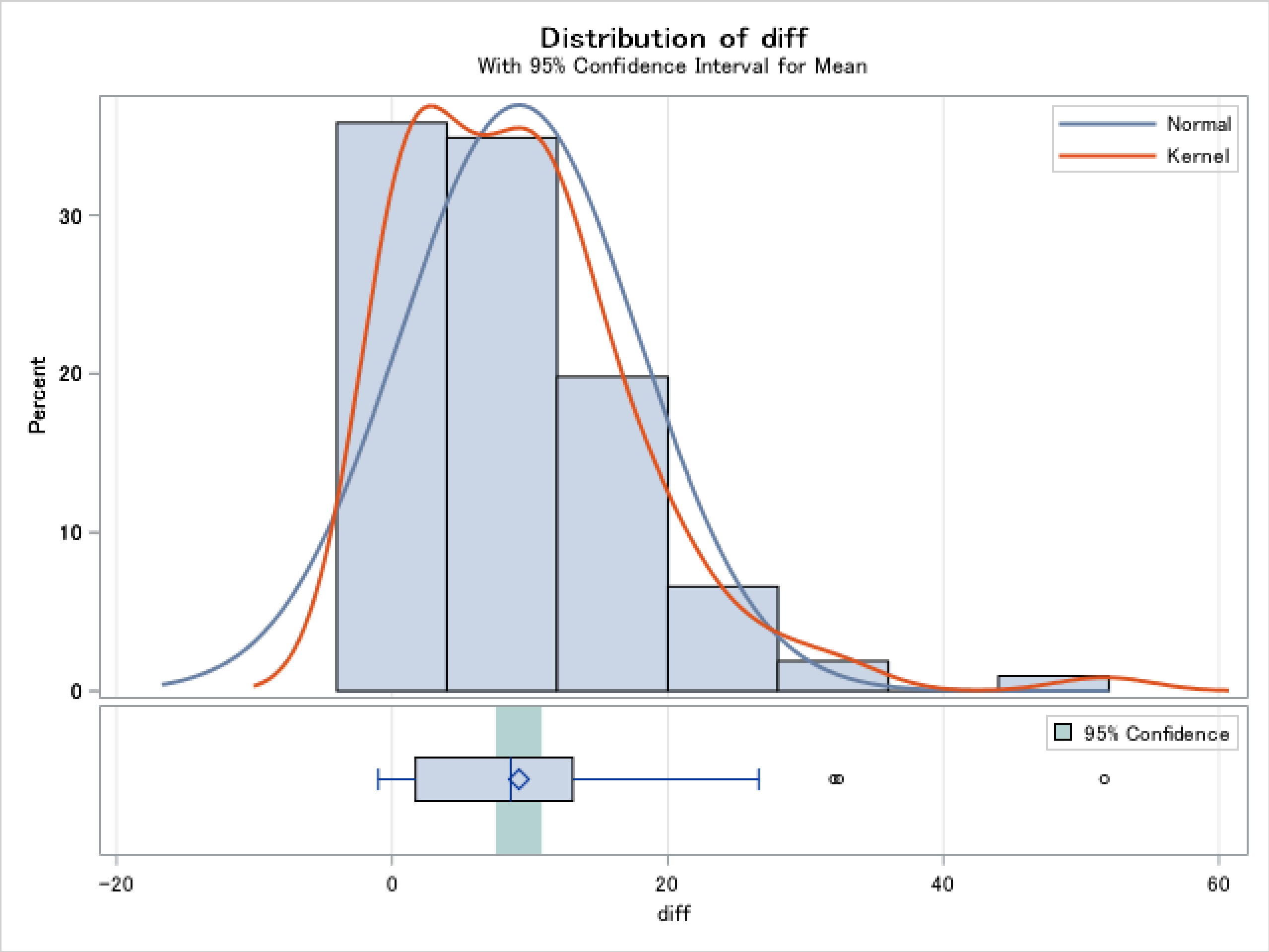

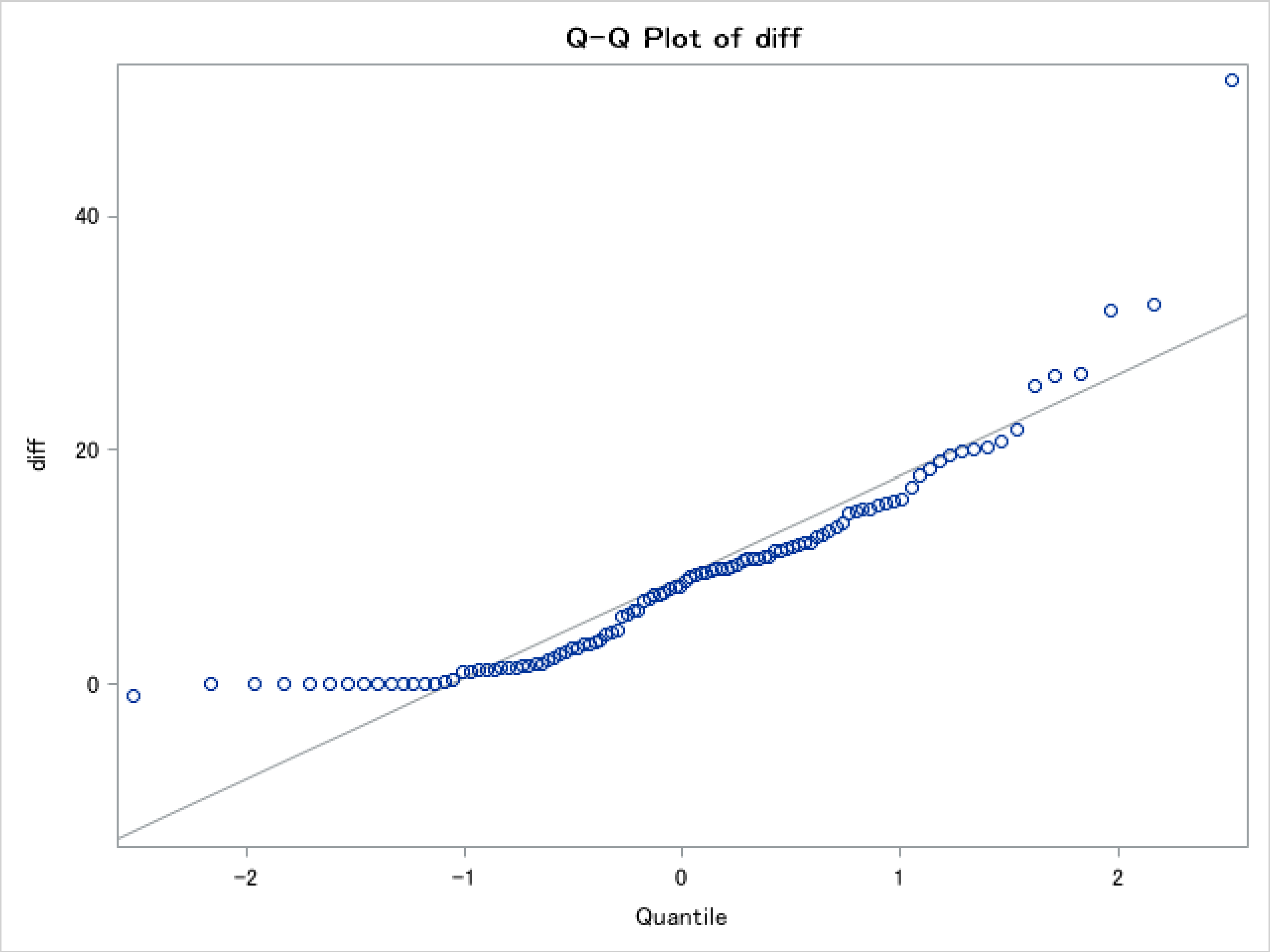
Q-Q Plot of diff
diff
40
20
0
−2
−1
0
1
2
Quantile

t-test on variable DIFF

The TTEST Procedure

Variable: diff

AVISIT=week 6 TRTA=G ARM=0.4 mg/g

N	Mean	Std Dev	Std Err	Minimum	Maximum
104	13.9148	13.4955	1.3233	-2.8098	90.1867

Mean	95% CL Mean		Std Dev	95% CL Std Dev	
13.9148	11.2903	16.5394	13.4955	11.8774	15.6279

DF	t Value	Pr > |t|
103	10.51	<.0001

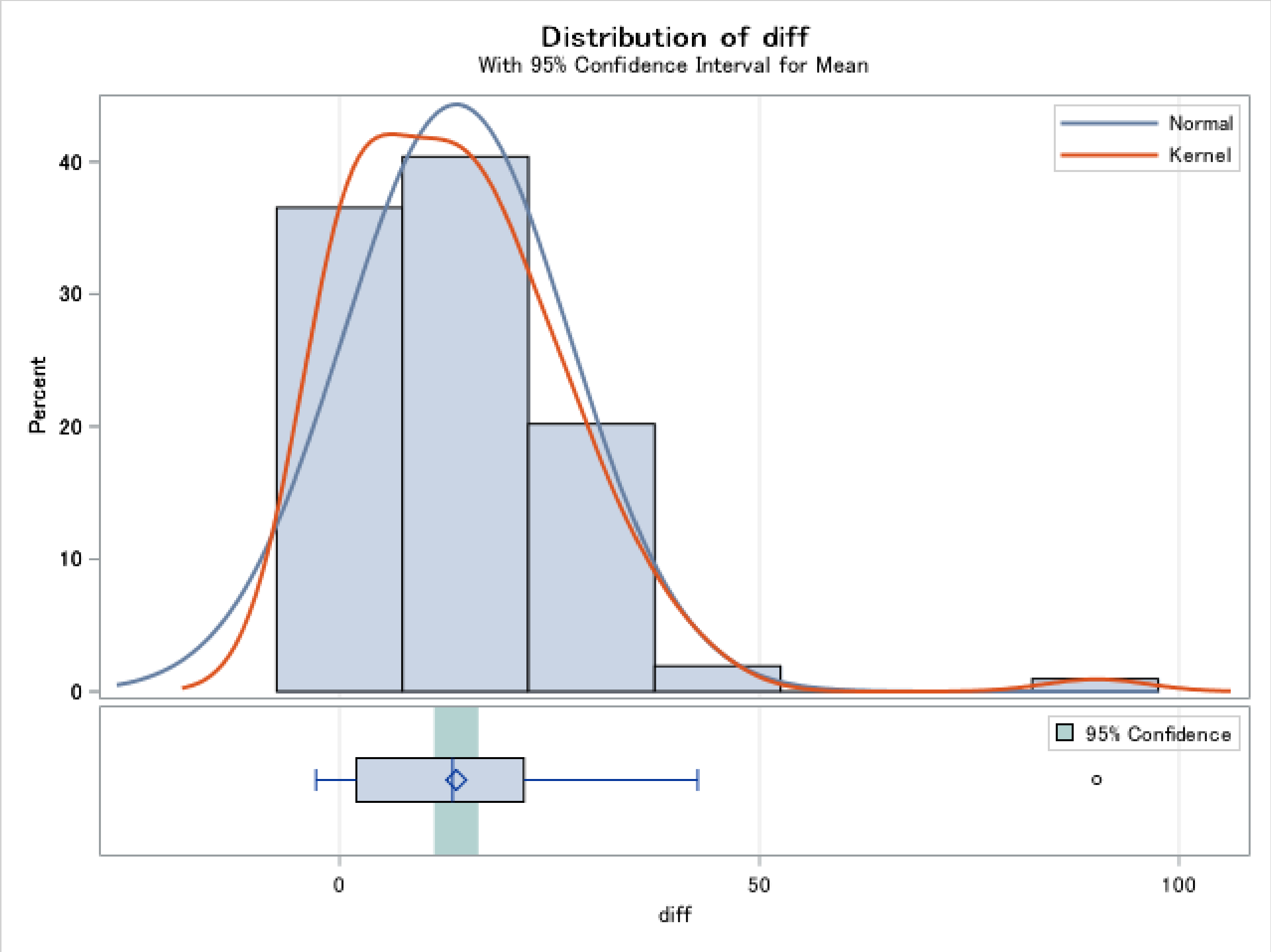

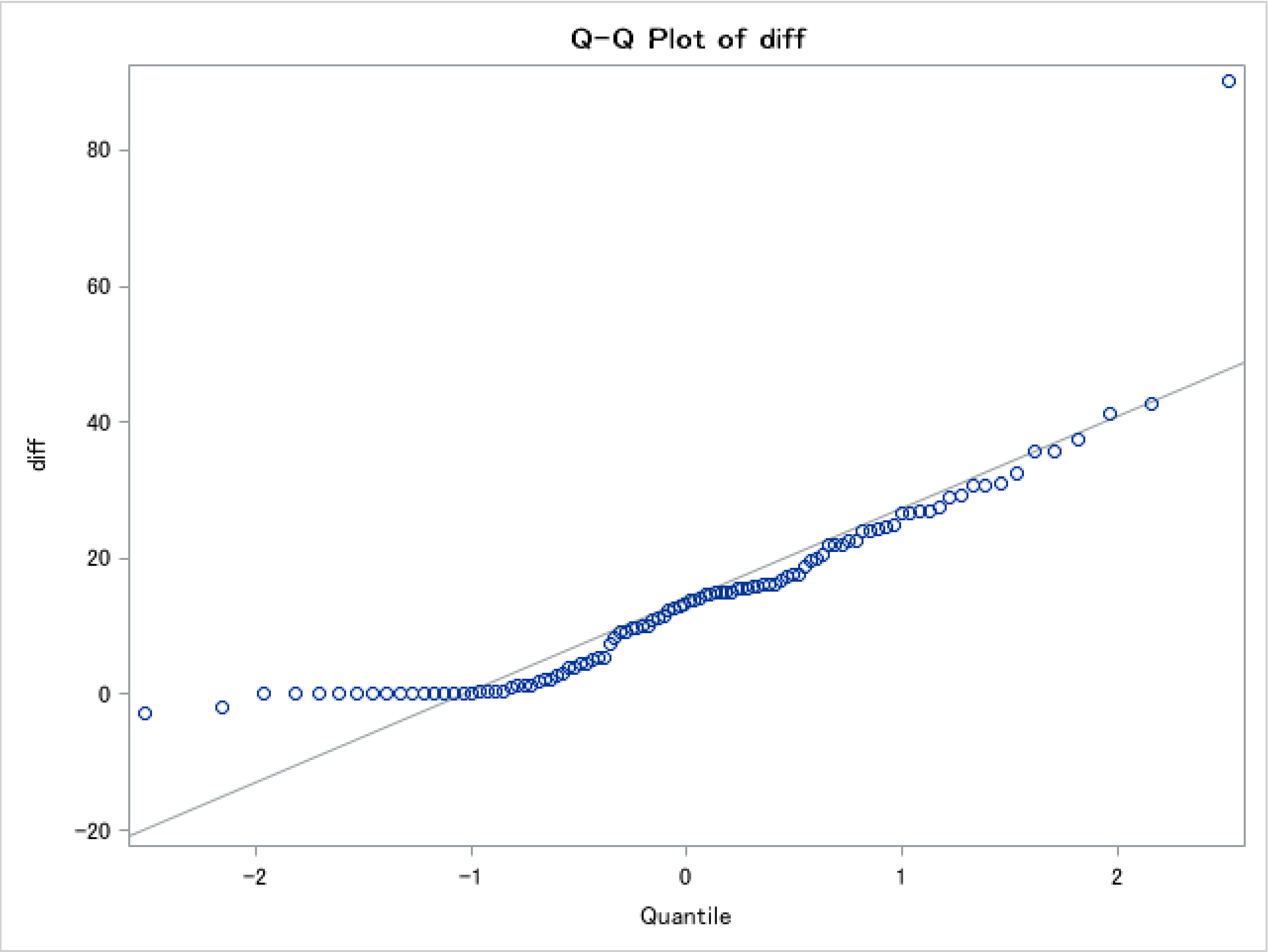
Q-Q Plot of diff
diff
80
60
40
20
0
−20
−2
−1
0
1
2
Quantile

log transformed CPD variables then conduct paired t-test

The TTEST Procedure

Difference: log_self - log_est
AVISIT=week 2 TRTA=A ARM=2.4 mg/g

N	Mean	Std Dev	Std Err	Minimum	Maximum
110	-1.4025	0.8566	0.0817	-3.4434	1.3437

Mean	95% CL Mean		Std Dev	95% CL Std Dev	
-1.4025	-1.5644	-1.2407	0.8566	0.7564	0.9876

DF	t Value	Pr > |t|
109	-17.17	<.0001

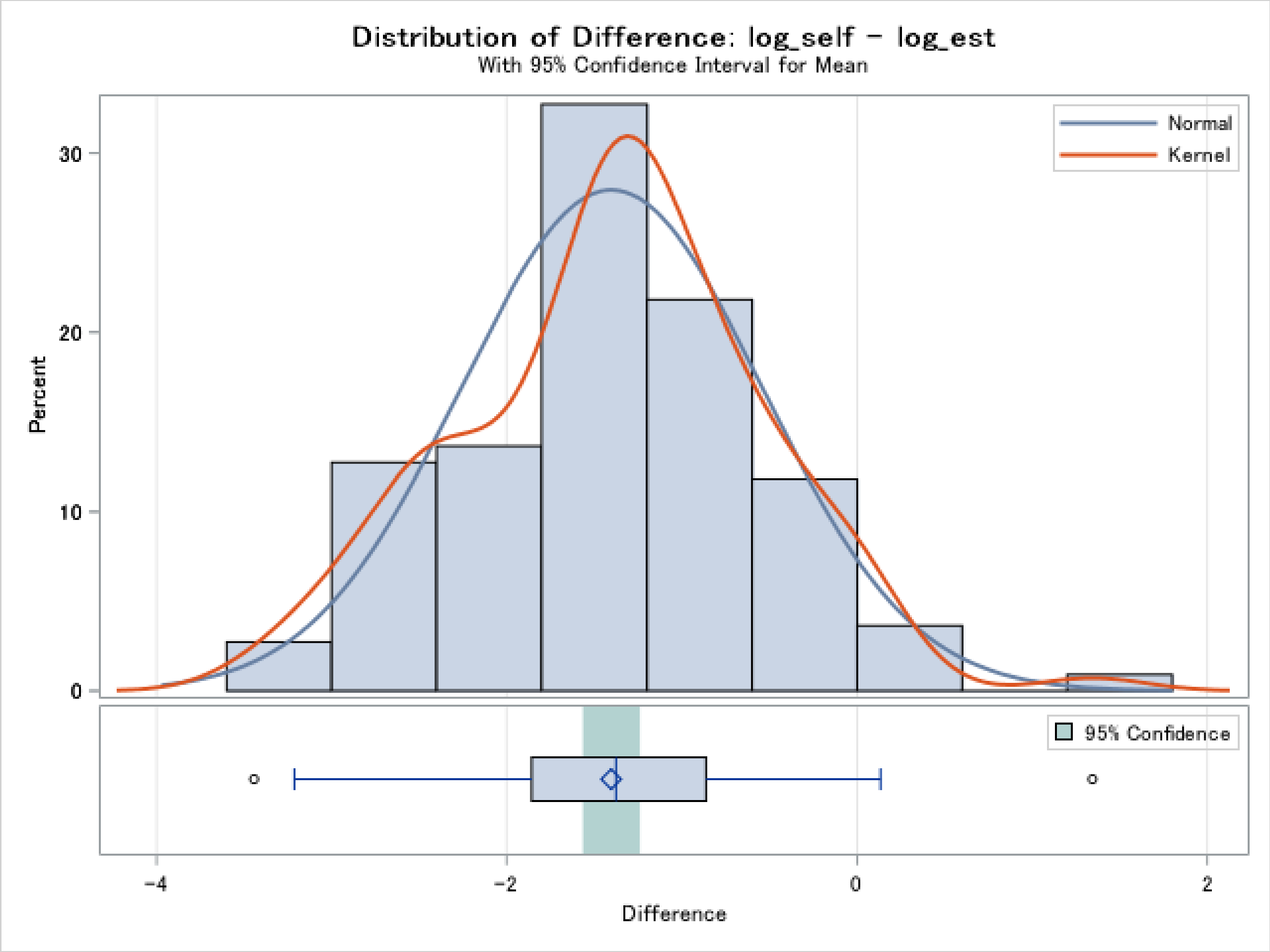

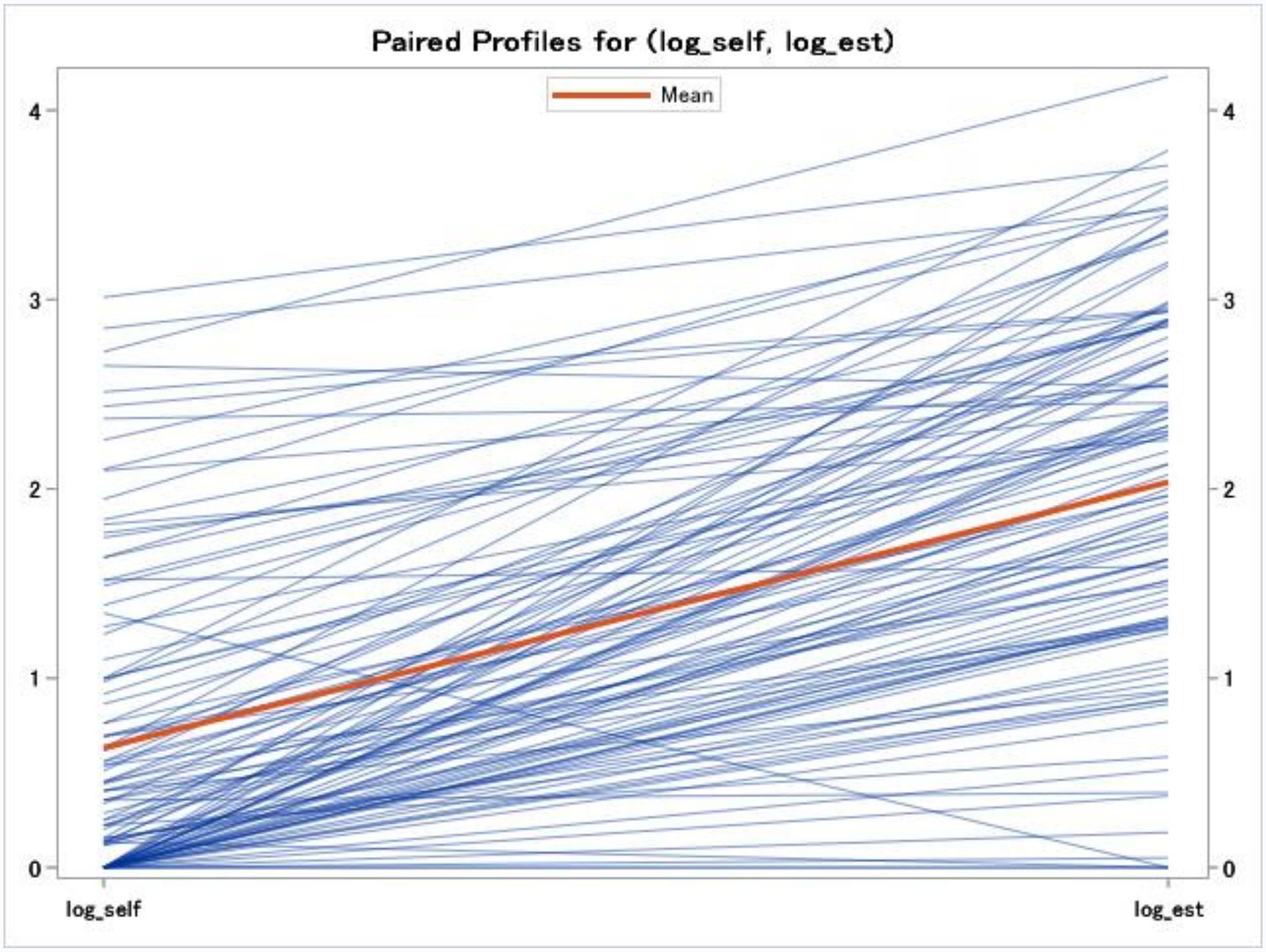

Paired Profiles for (log_self, log_est)
Mean
4
3
2
1
0
log_self
log_est

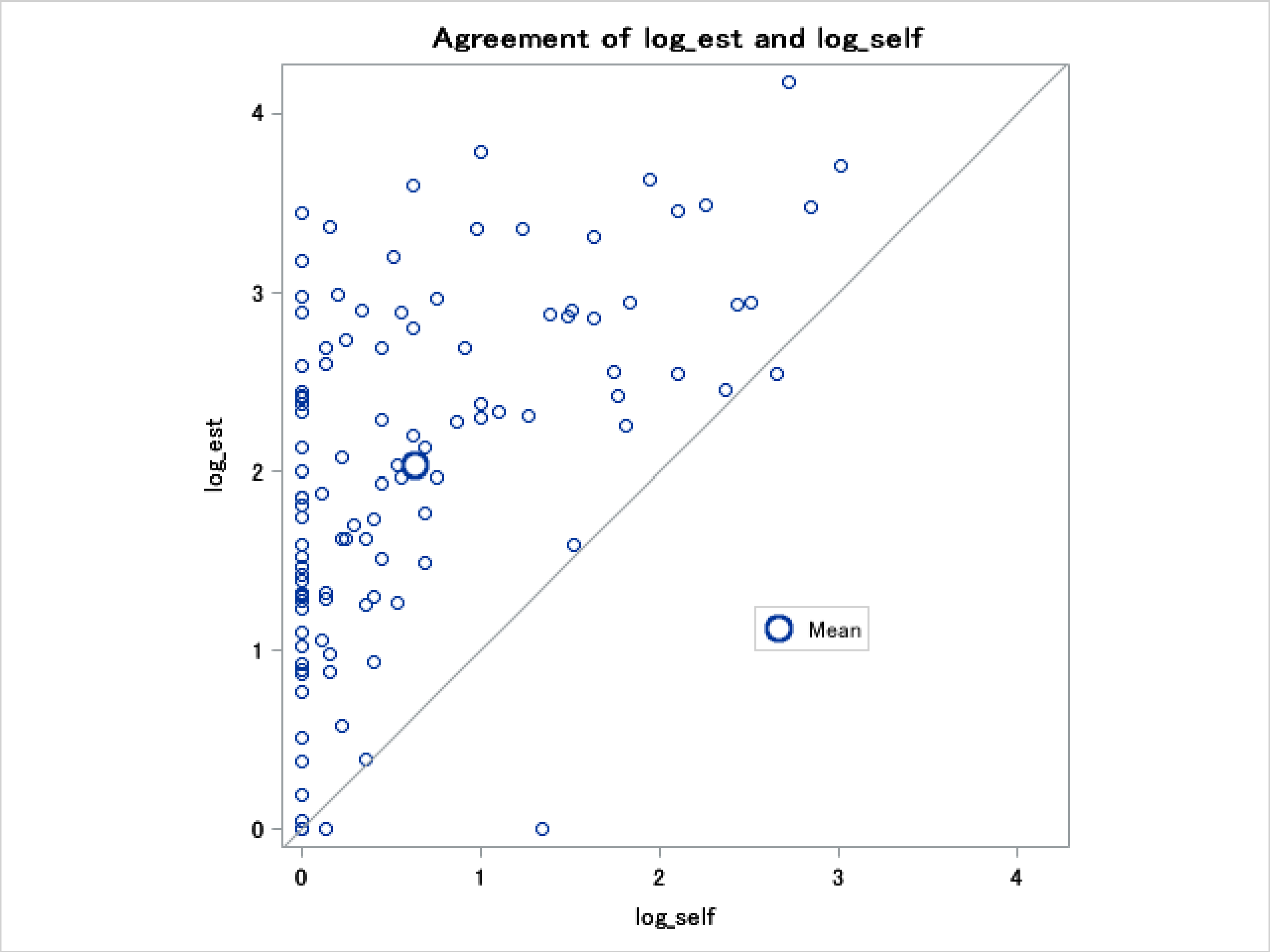

Agreement of log_est and log_self
log_est
log_self
0
1
2
3
4
0
1
2
3
4
Mean

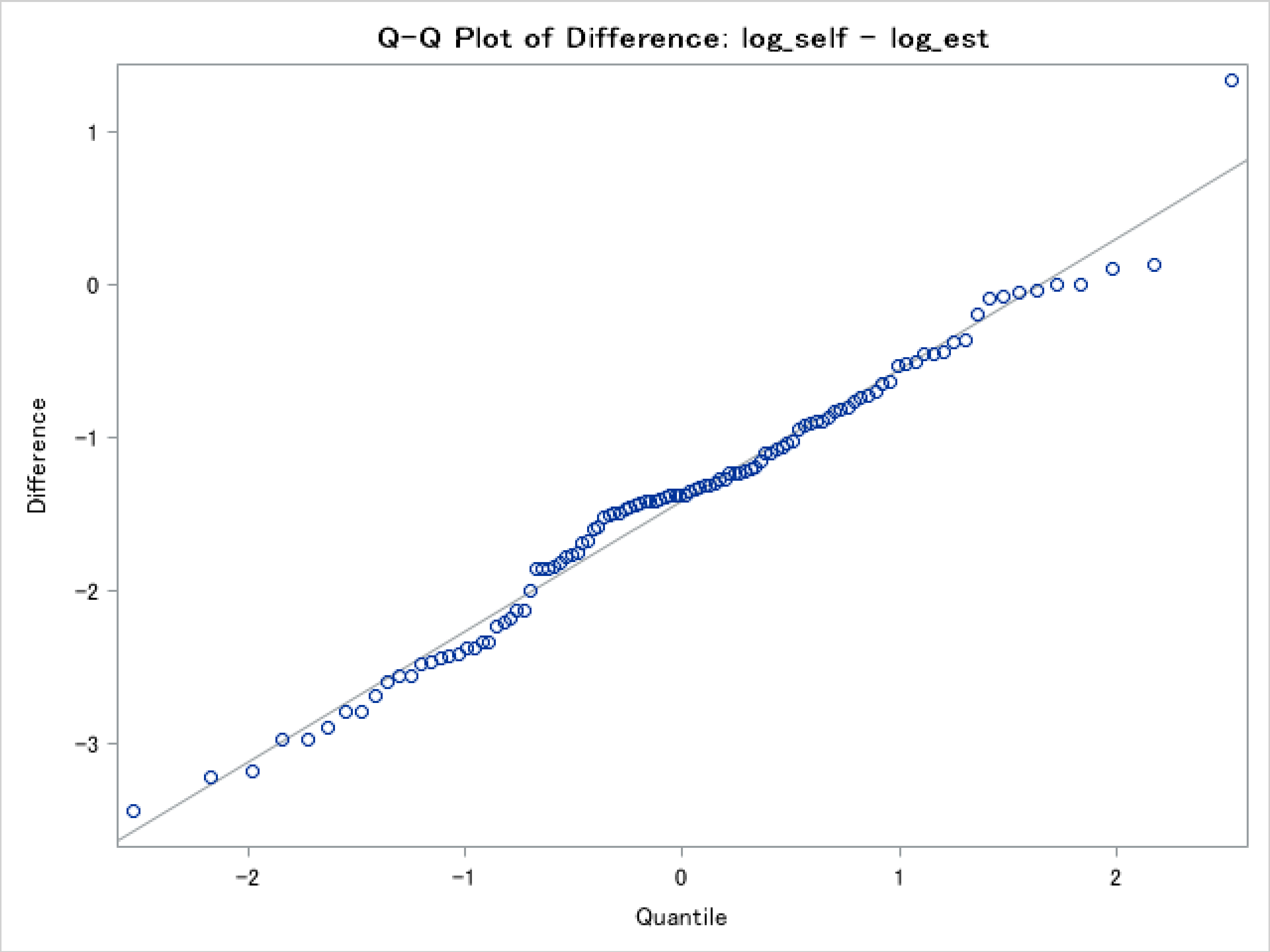

Q-Q Plot of Difference: log_self - log_est
1
0
-1
-2
-3
Difference
-2
-1
0
1
2
Quantile

log transformed CPD variables then conduct paired t-test

The TTEST Procedure

Difference: log_self - log_est

AVISIT=week 2 TRTA=B ARM=0.4 mg/g (HT)

N	Mean	Std Dev	Std Err	Minimum	Maximum
115	-1.4317	1.0272	0.0958	-4.4234	0.2634

Mean	95% CL Mean		Std Dev	95% CL Std Dev	
-1.4317	-1.6214	-1.2419	1.0272	0.9095	1.1804

DF	t Value	Pr > |t|
114	-14.95	<.0001

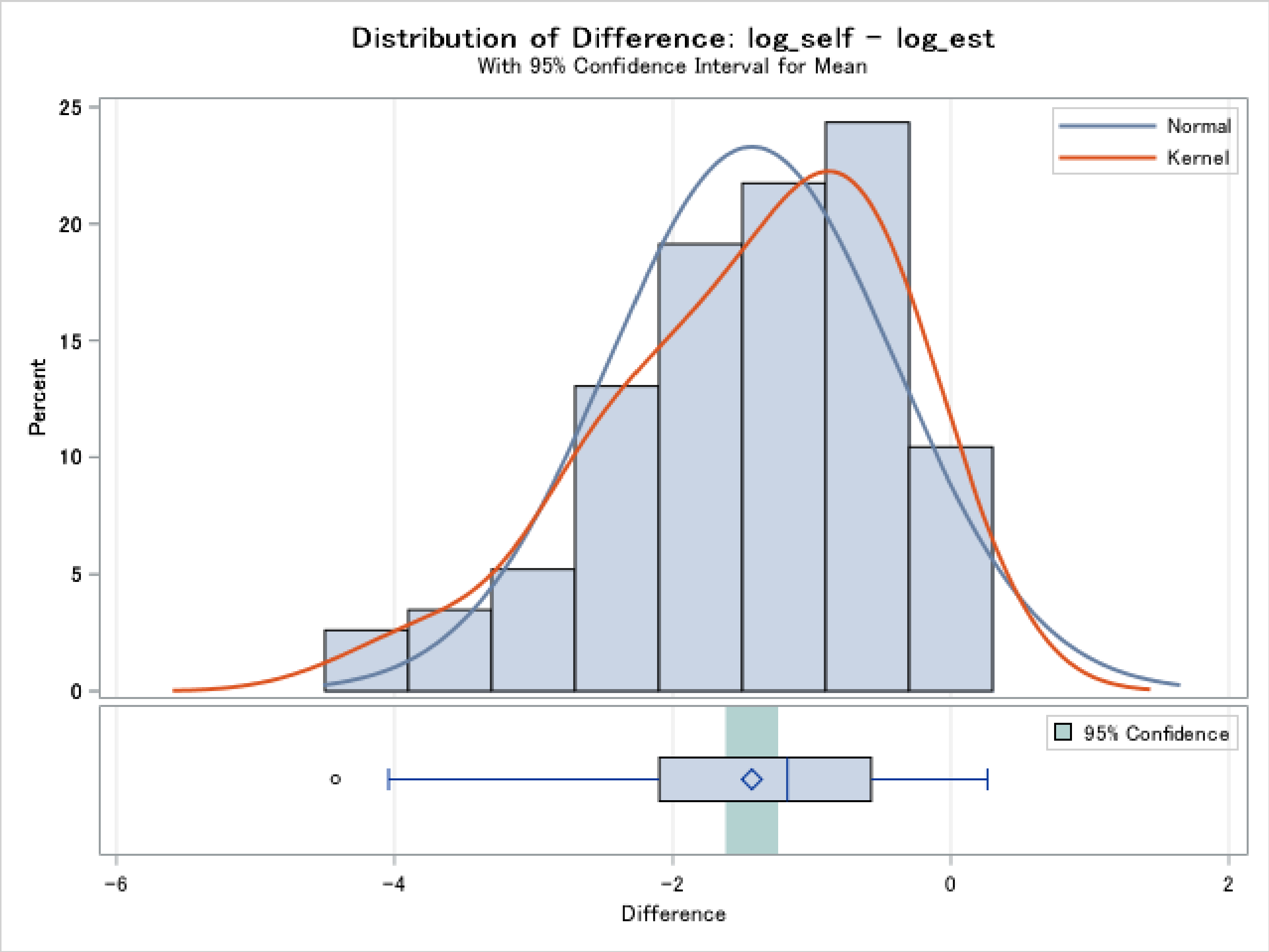

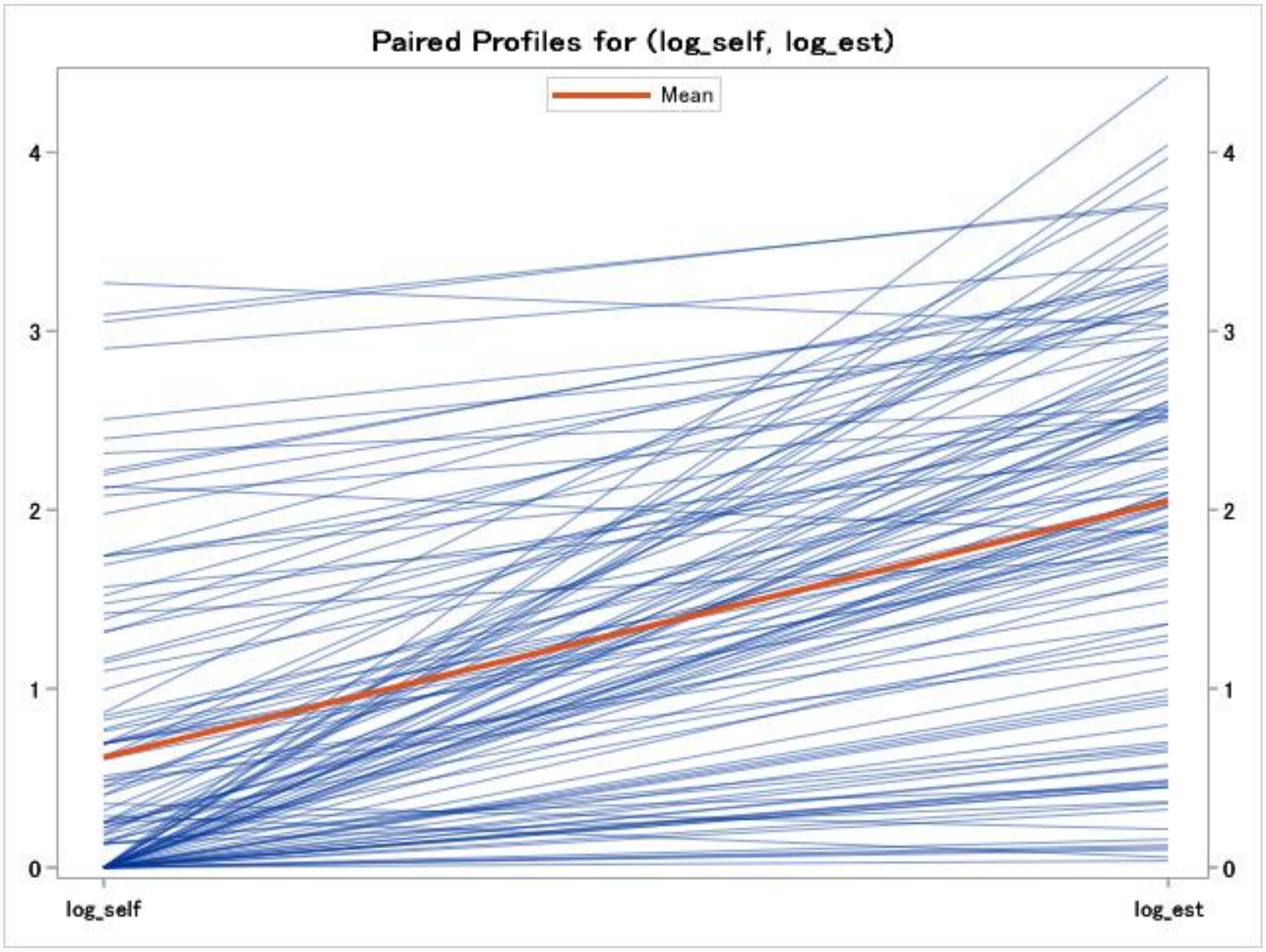
Paired Profiles for (log_self, log_est)
Mean
4
3
2
1
0
log_self
log_est

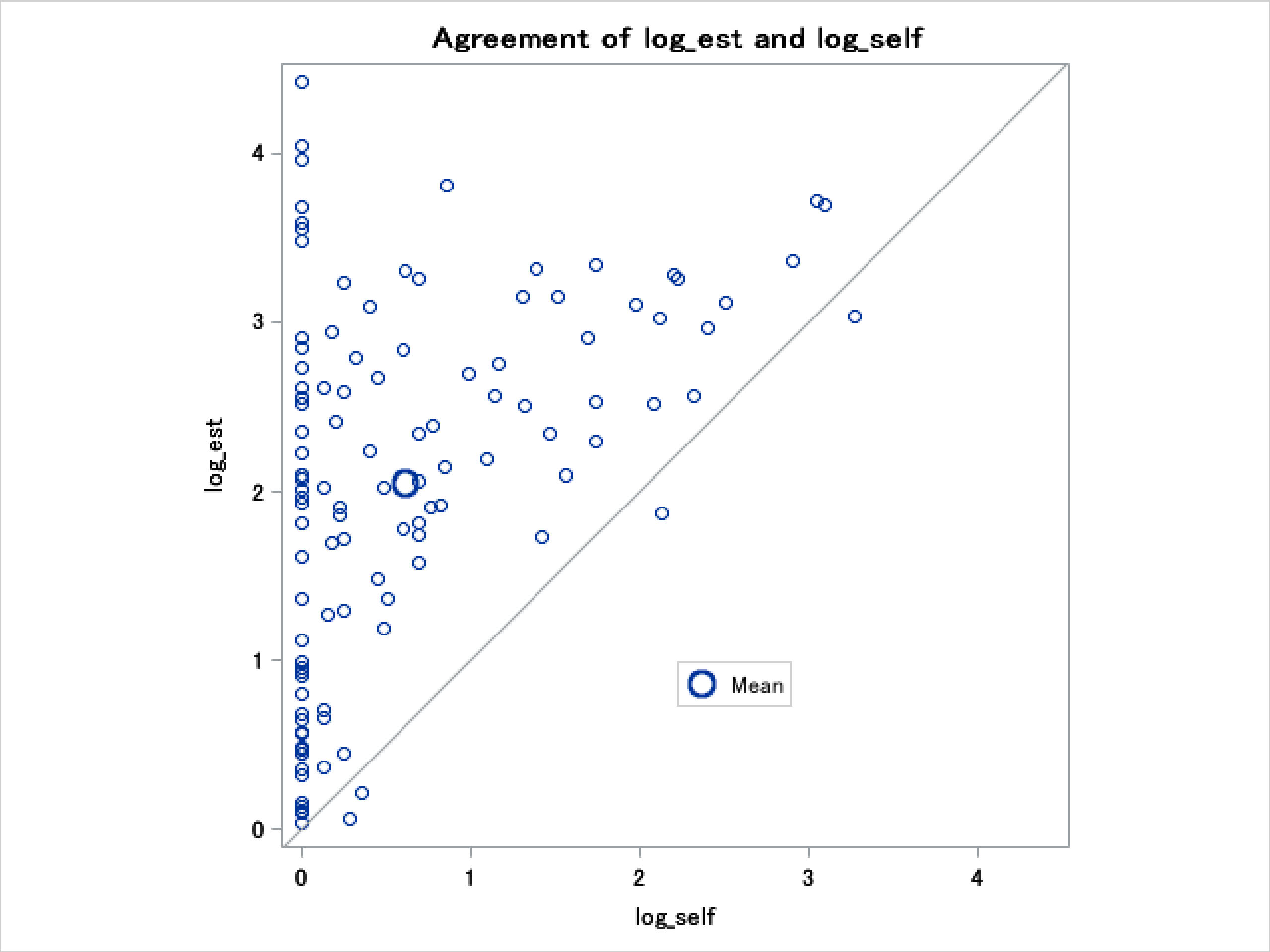
Agreement of log_est and log_self
log_est
log_self
Mean
0
1
2
3
4
0
1
2
3
4

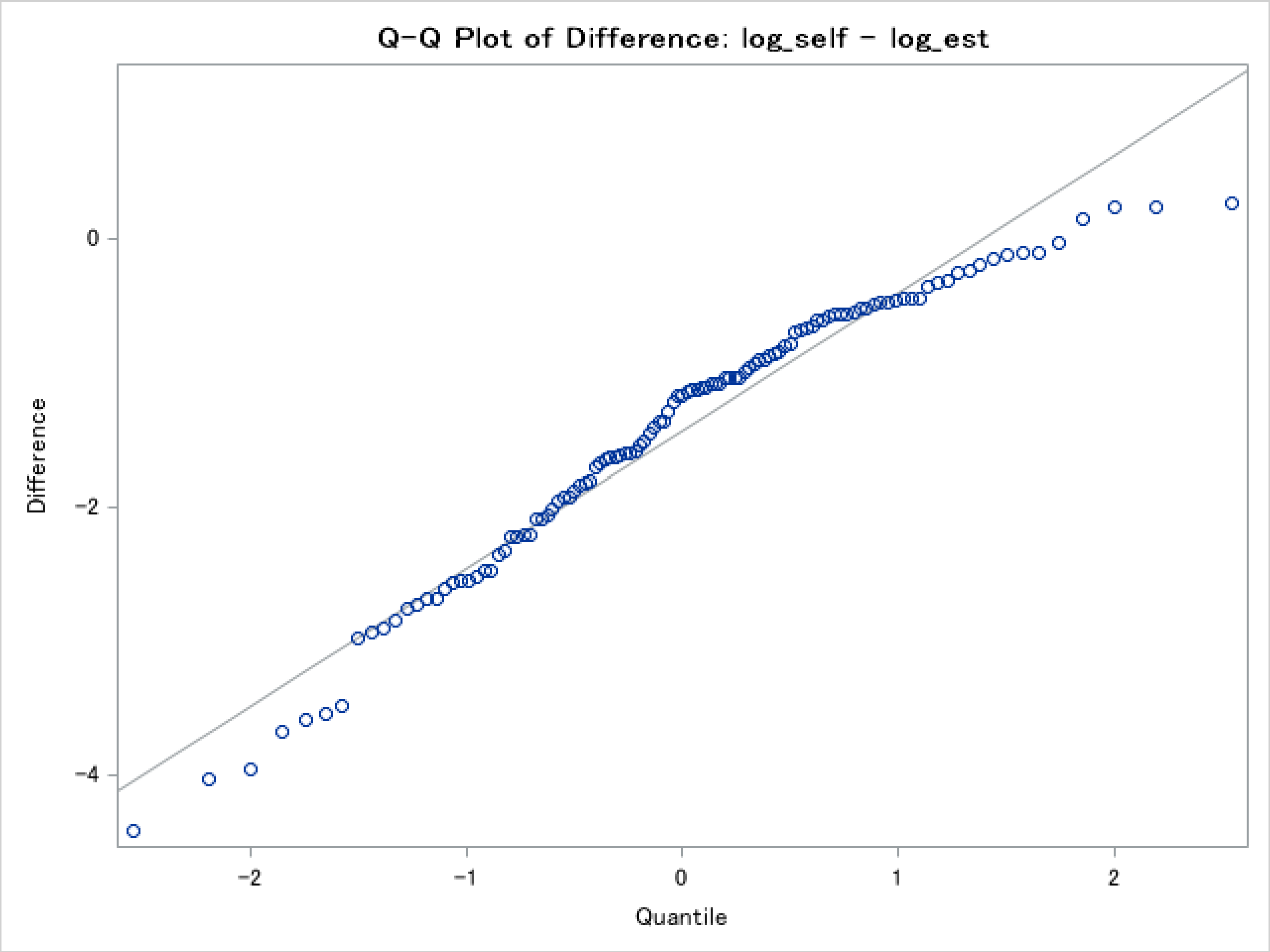

Q-Q Plot of Difference: log_self - log_est
Difference
0
-2
-4
-2
-1
0
1
2
Quantile

log transformed CPD variables then conduct paired t-test

The TTEST Procedure

Difference: log_self - log_est
AVISIT=week 2 TRTA=D ARM=1.3 mg/g

N	Mean	Std Dev	Std Err	Minimum	Maximum
110	-1.4962	1.1332	0.1080	-3.9819	1.1477

Mean	95% CL Mean		Std Dev	95% CL Std Dev	
-1.4962	-1.7104	-1.2821	1.1332	1.0007	1.3065

DF	t Value	Pr > |t|
109	-13.85	<.0001

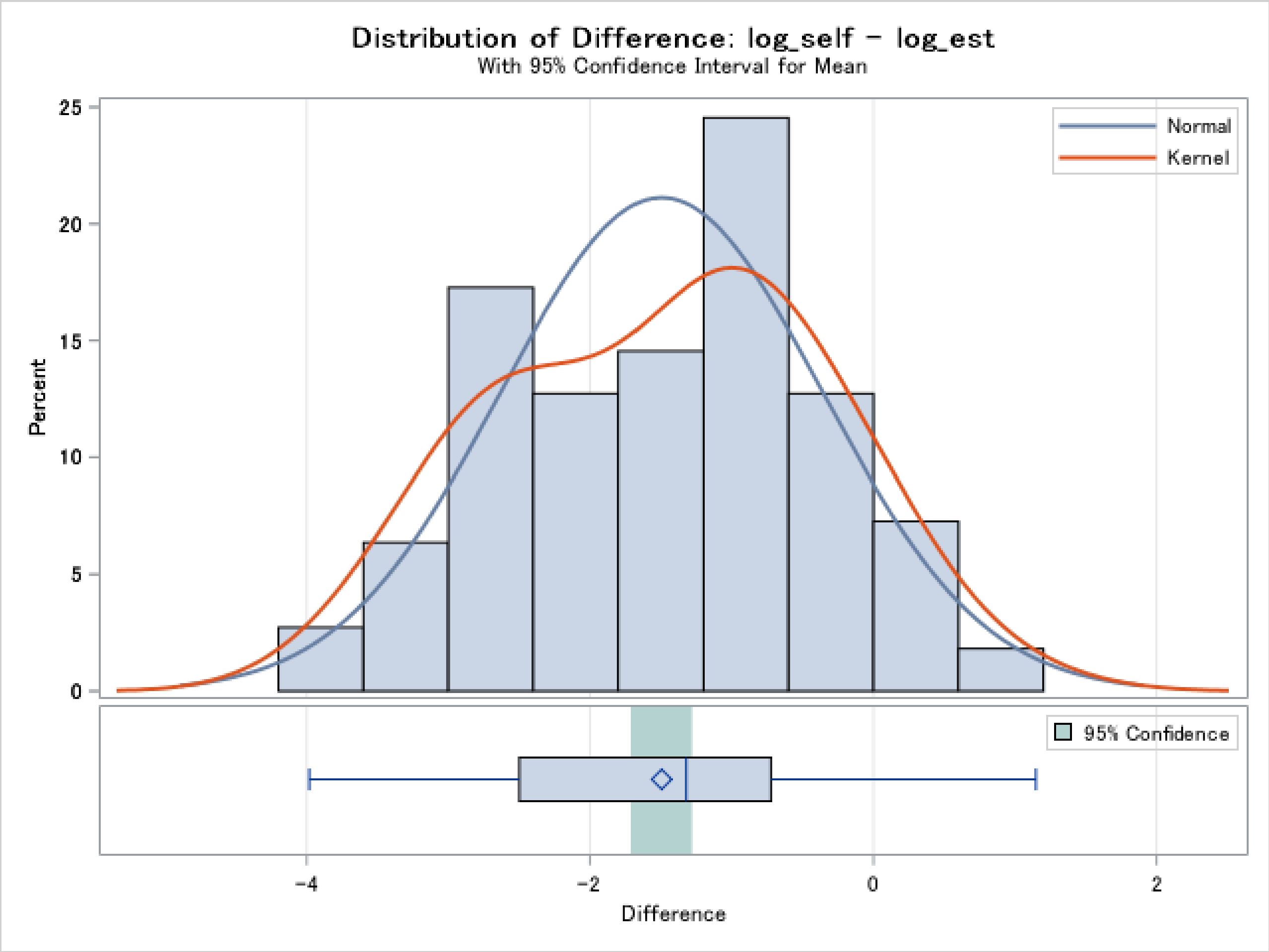

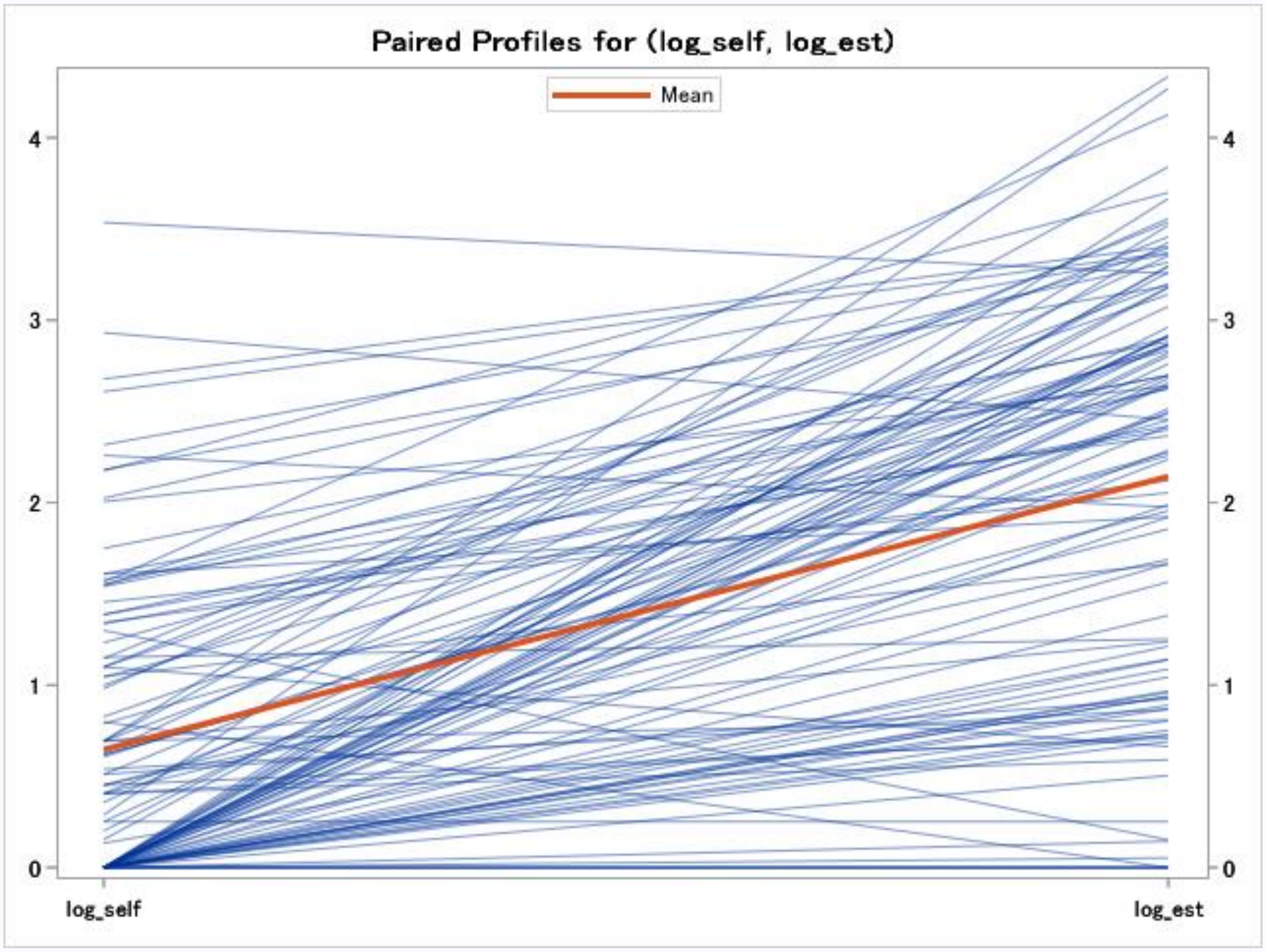
Paired Profiles for (log_self, log_est)
Mean
0
1
2
3
4
log_self
log_est

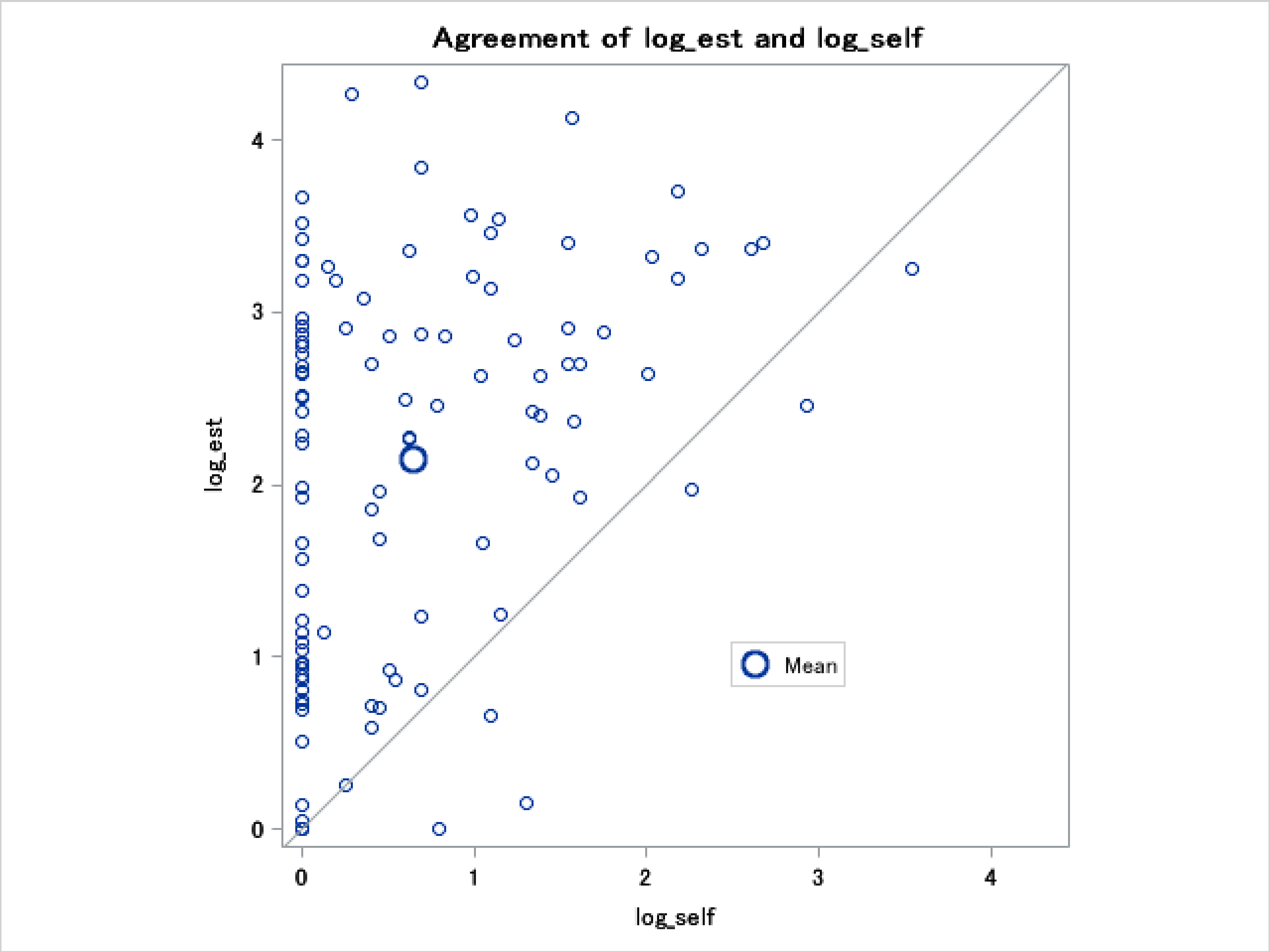

Agreement of log_est and log_self
log_est
log_self
Mean
0
1
2
3
4
0
1
2
3
4

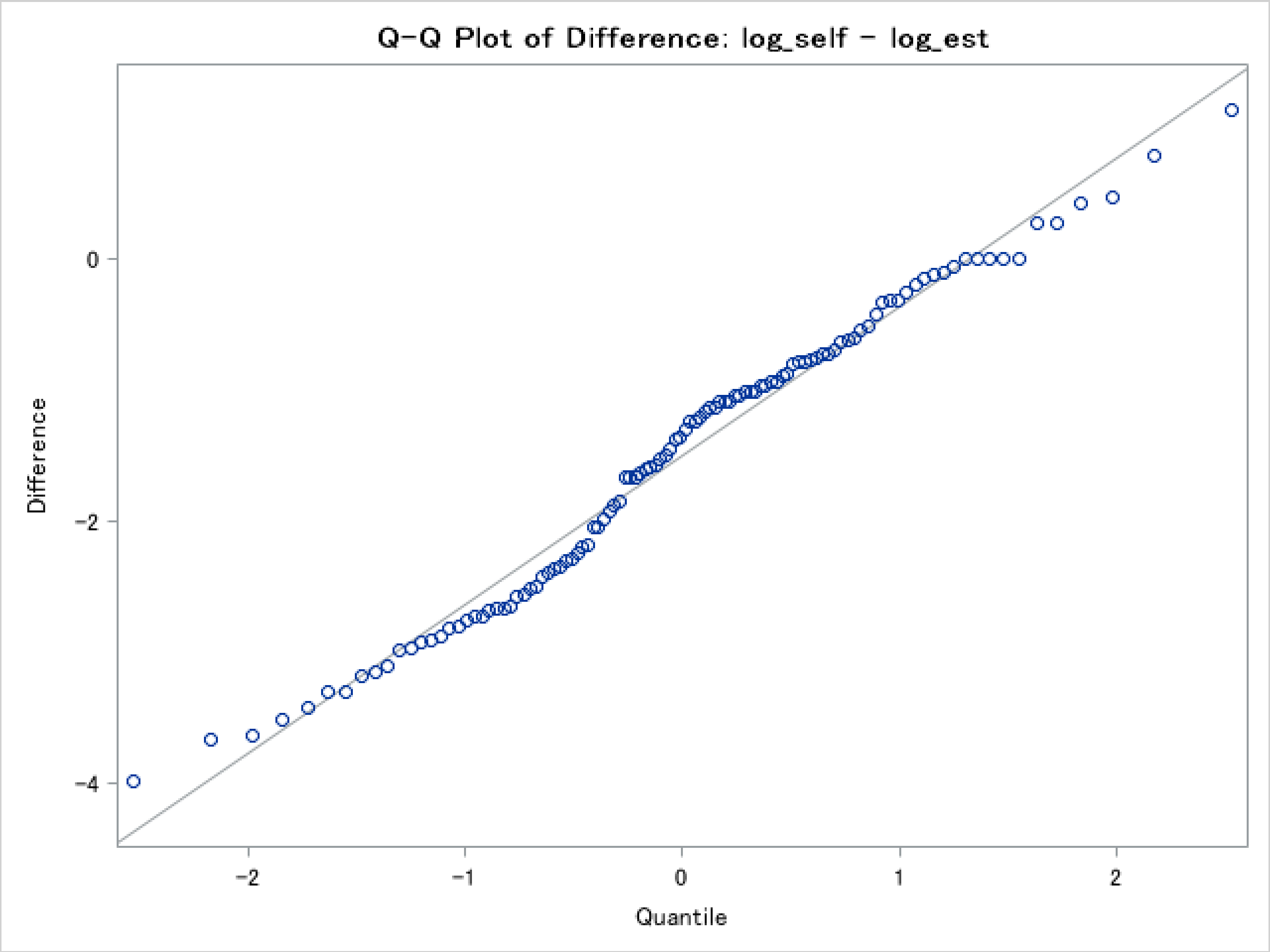

Q-Q Plot of Difference: log_self - log_est
Difference
Quantile
0
-2
-4
-2
-1
0
1
2

log transformed CPD variables then conduct paired t-test

The TTEST Procedure

Difference: log_self - log_est
AVISIT=week 2 TRTA=F ARM=5.2 mg/g

N	Mean	Std Dev	Std Err	Minimum	Maximum
110	-1.2698	1.0831	0.1033	-4.9989	1.5404

Mean	95% CL Mean		Std Dev	95% CL Std Dev	
-1.2698	-1.4745	-1.0651	1.0831	0.9564	1.2487

DF	t Value	Pr > |t|
109	-12.30	<.0001

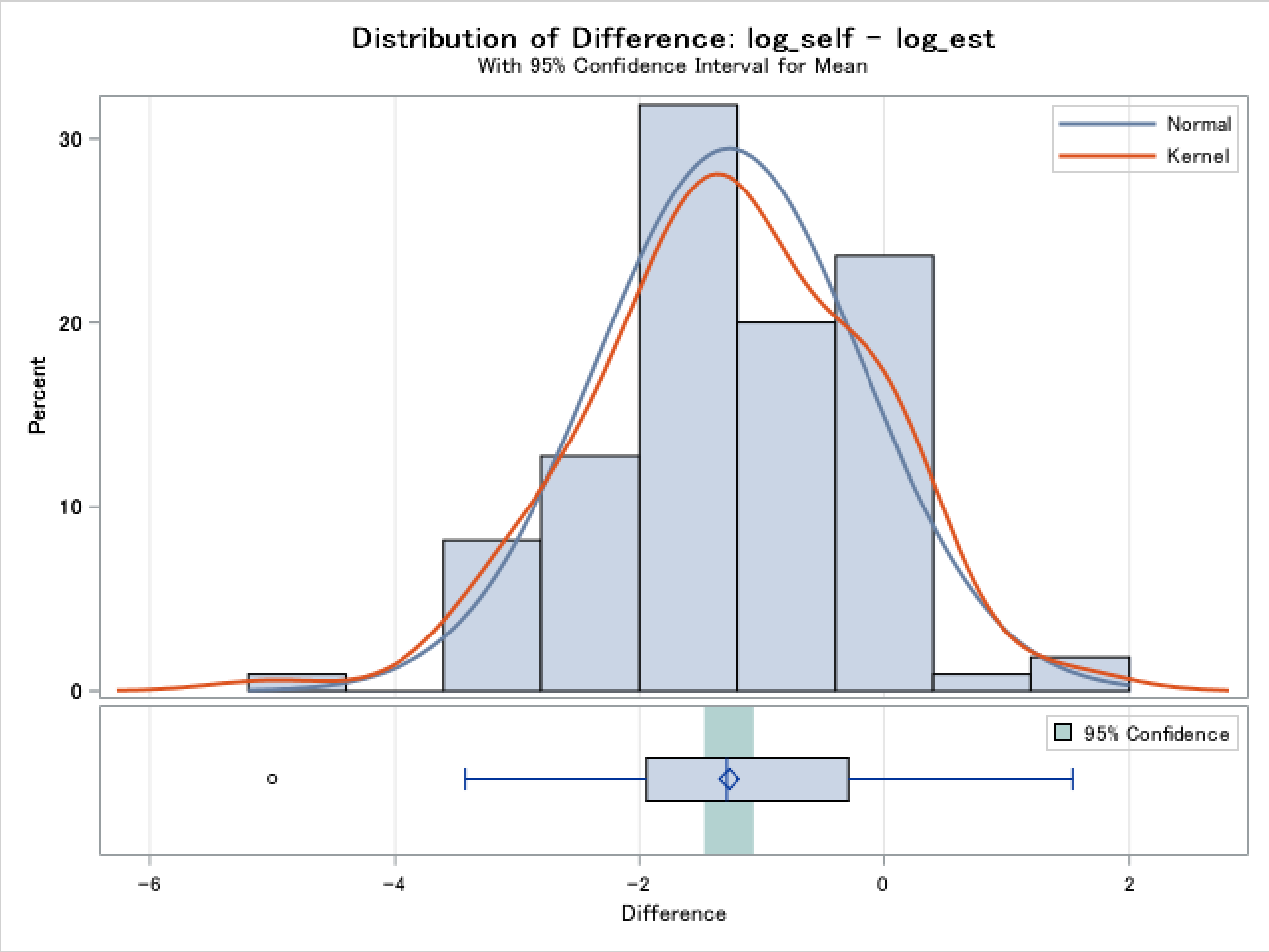

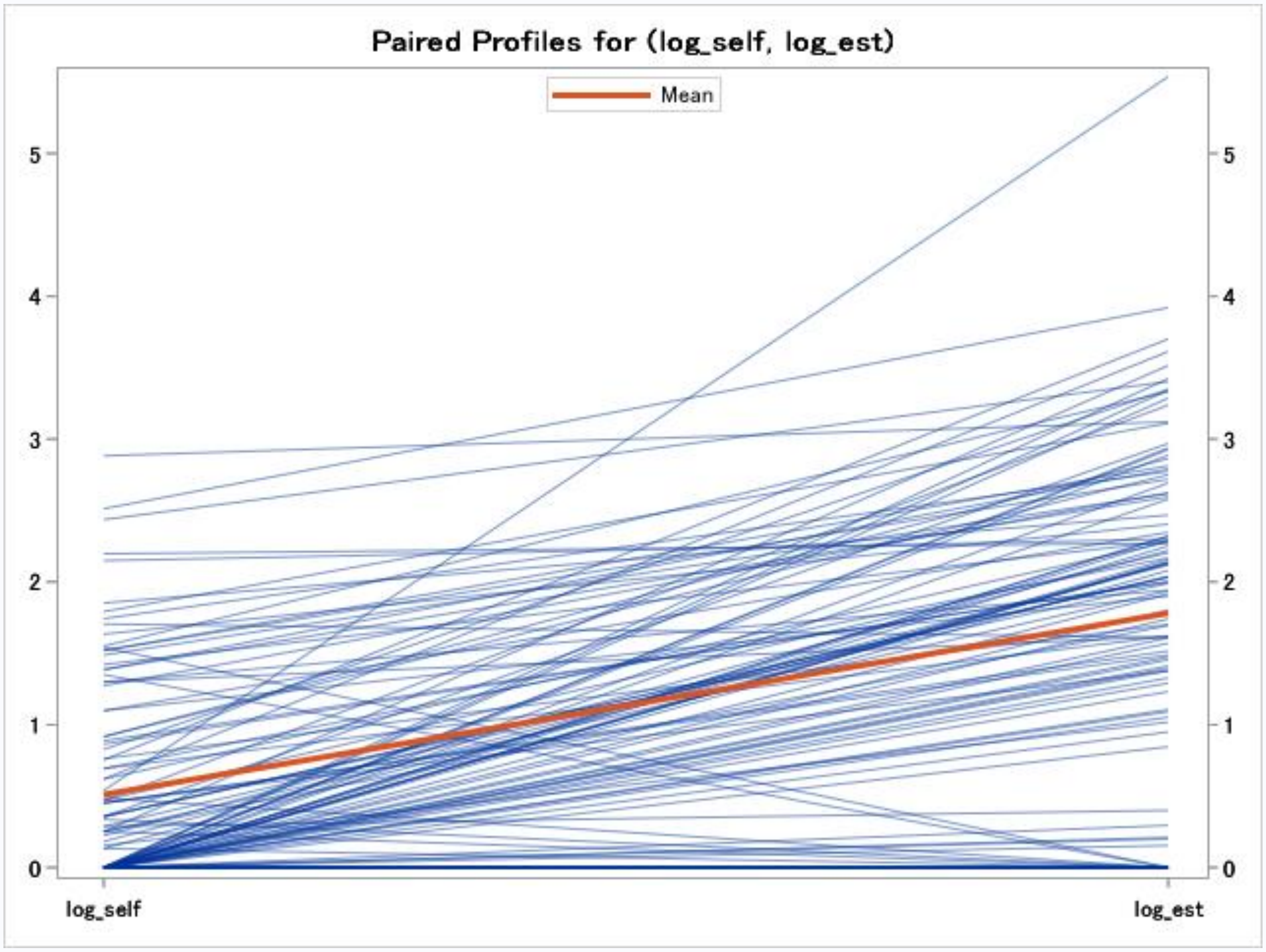

Paired Profiles for (log_self, log_est)
Mean
0
1
2
3
4
5
log_self
log_est

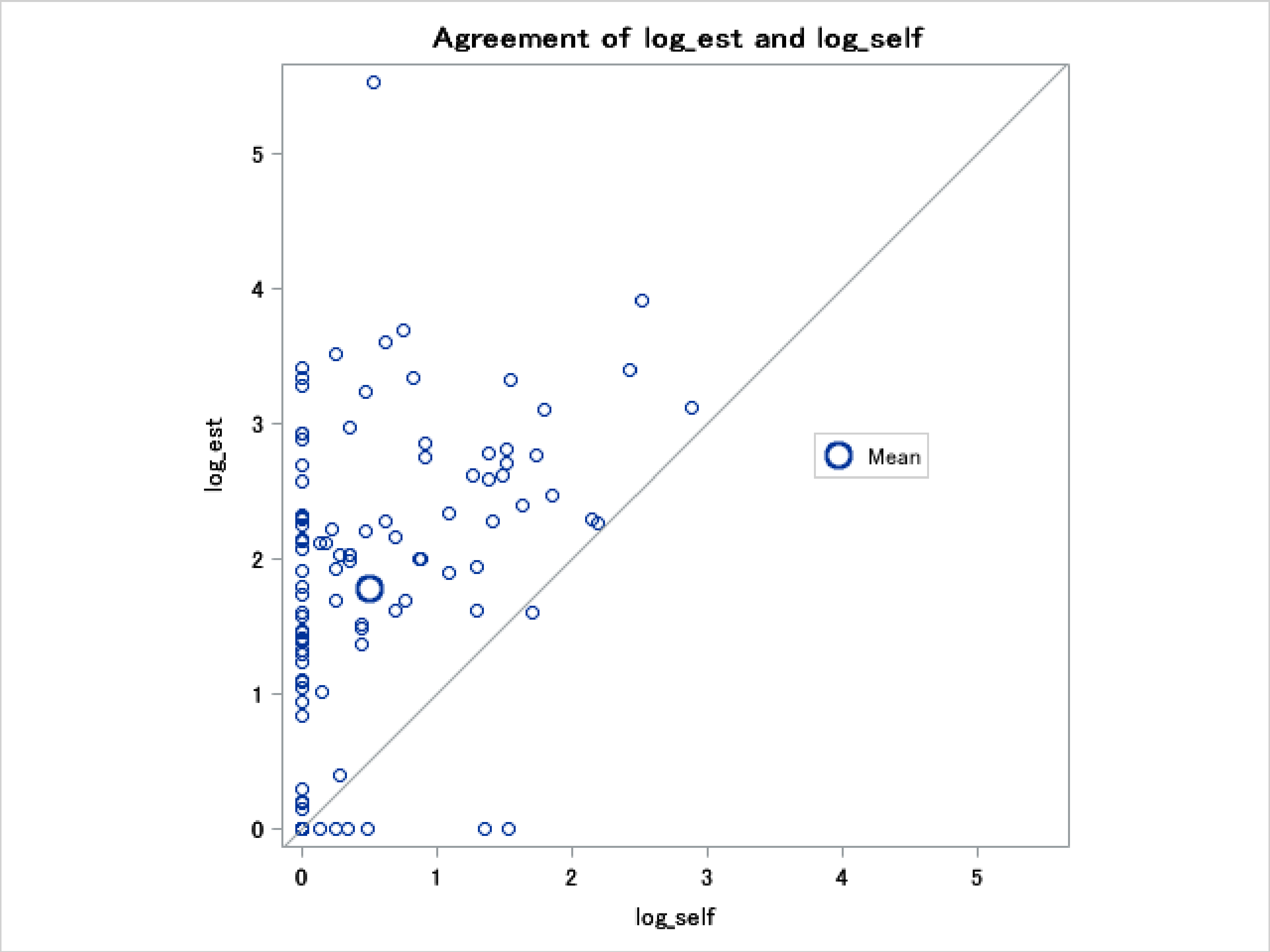

Agreement of log_est and log_self
log_est
log_self
Mean
0
1
2
3
4
5

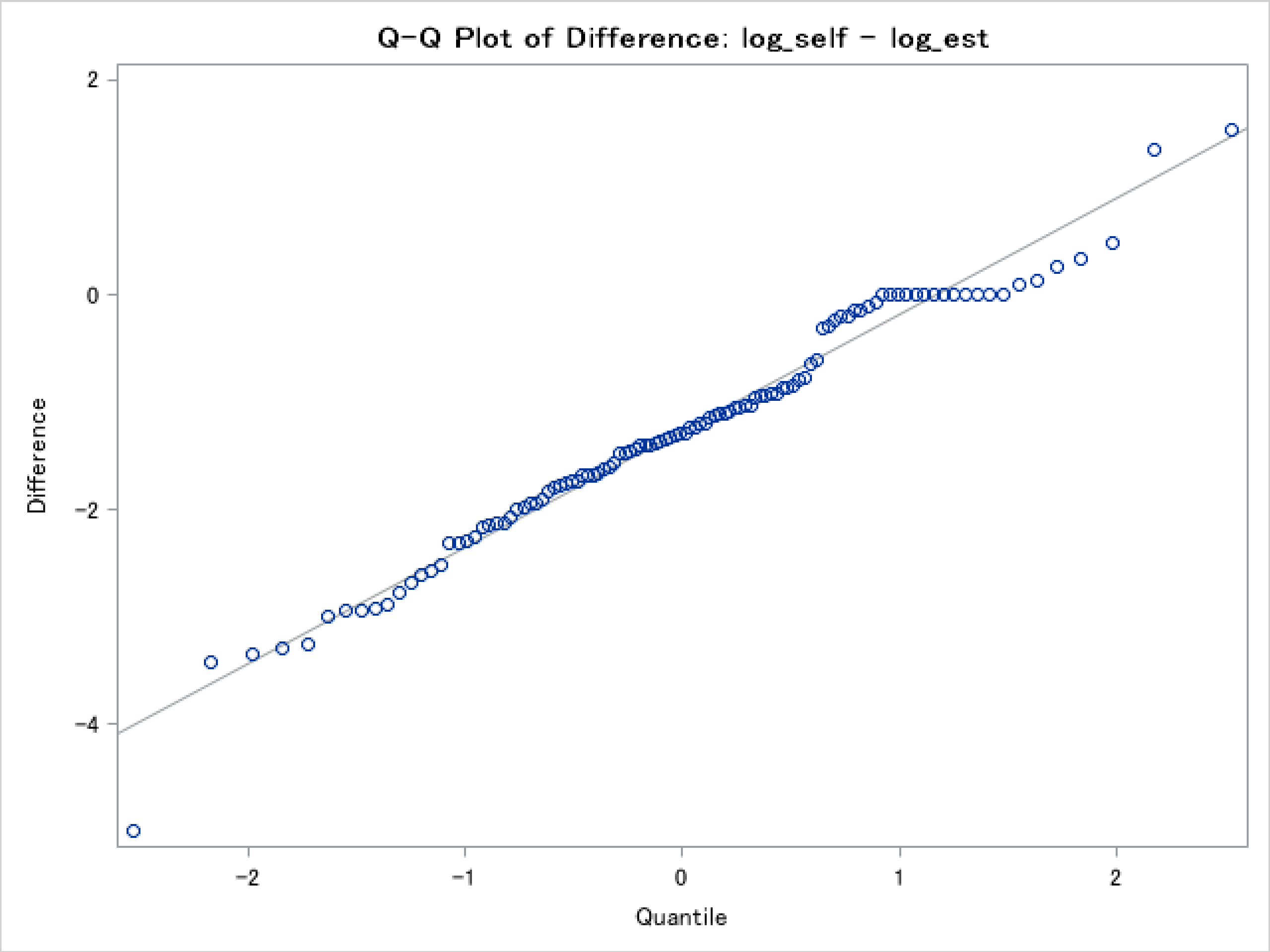
Q-Q Plot of Difference: log_self - log_est
Difference
Quantile
2
0
-2
-4
−2
−1
0
1
2

log transformed CPD variables then conduct paired t-test

The TTEST Procedure

Difference: log_self - log_est

AVISIT=week 2 TRTA=G ARM=0.4 mg/g

N	Mean	Std Dev	Std Err	Minimum	Maximum
111	-1.6101	1.1649	0.1106	-4.9637	0.4739

Mean	95% CL Mean		Std Dev	95% CL Std Dev	
-1.6101	-1.8293	-1.3910	1.1649	1.0292	1.3421

DF	t Value	Pr > |t|
110	-14.56	<.0001

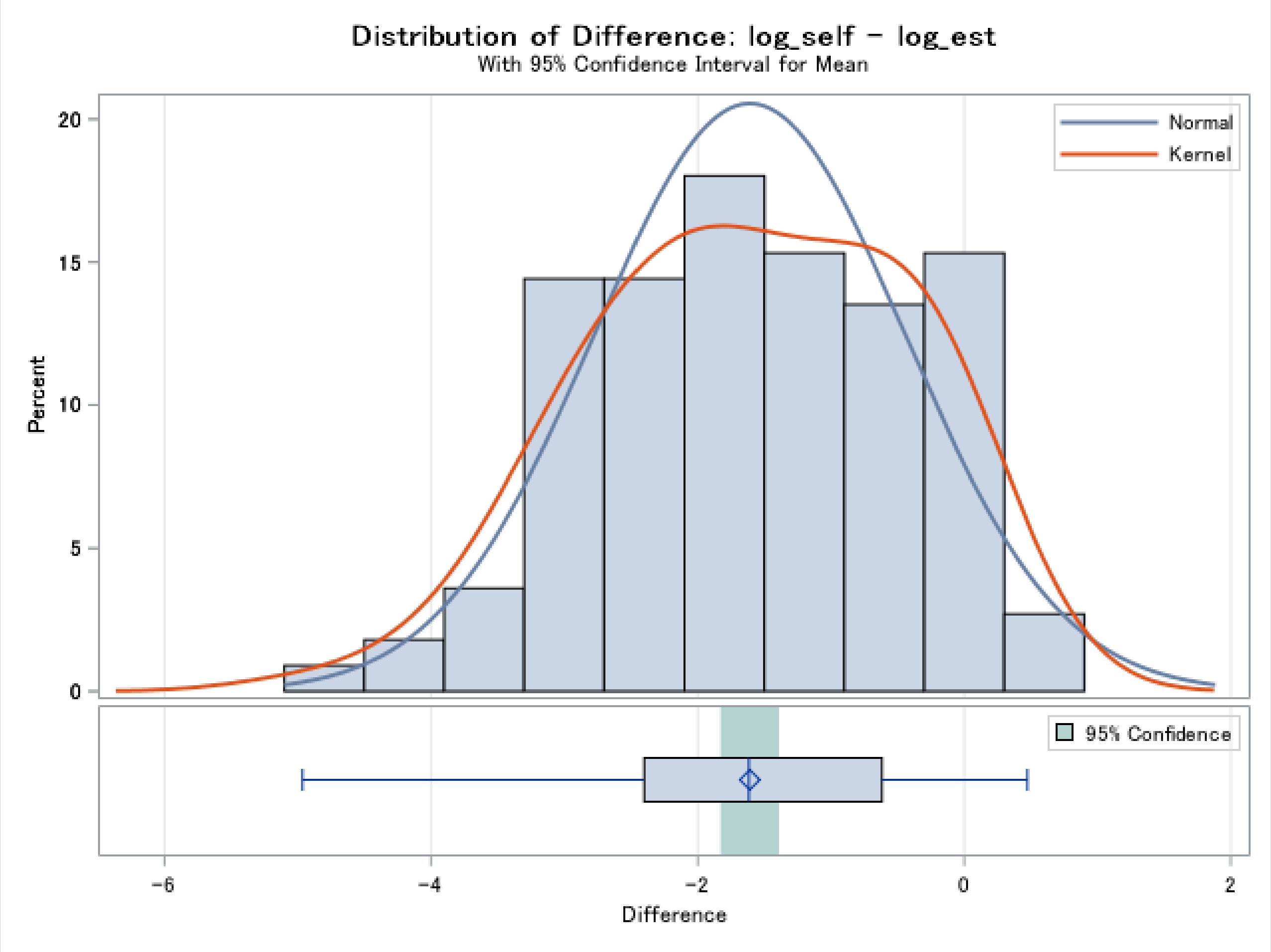

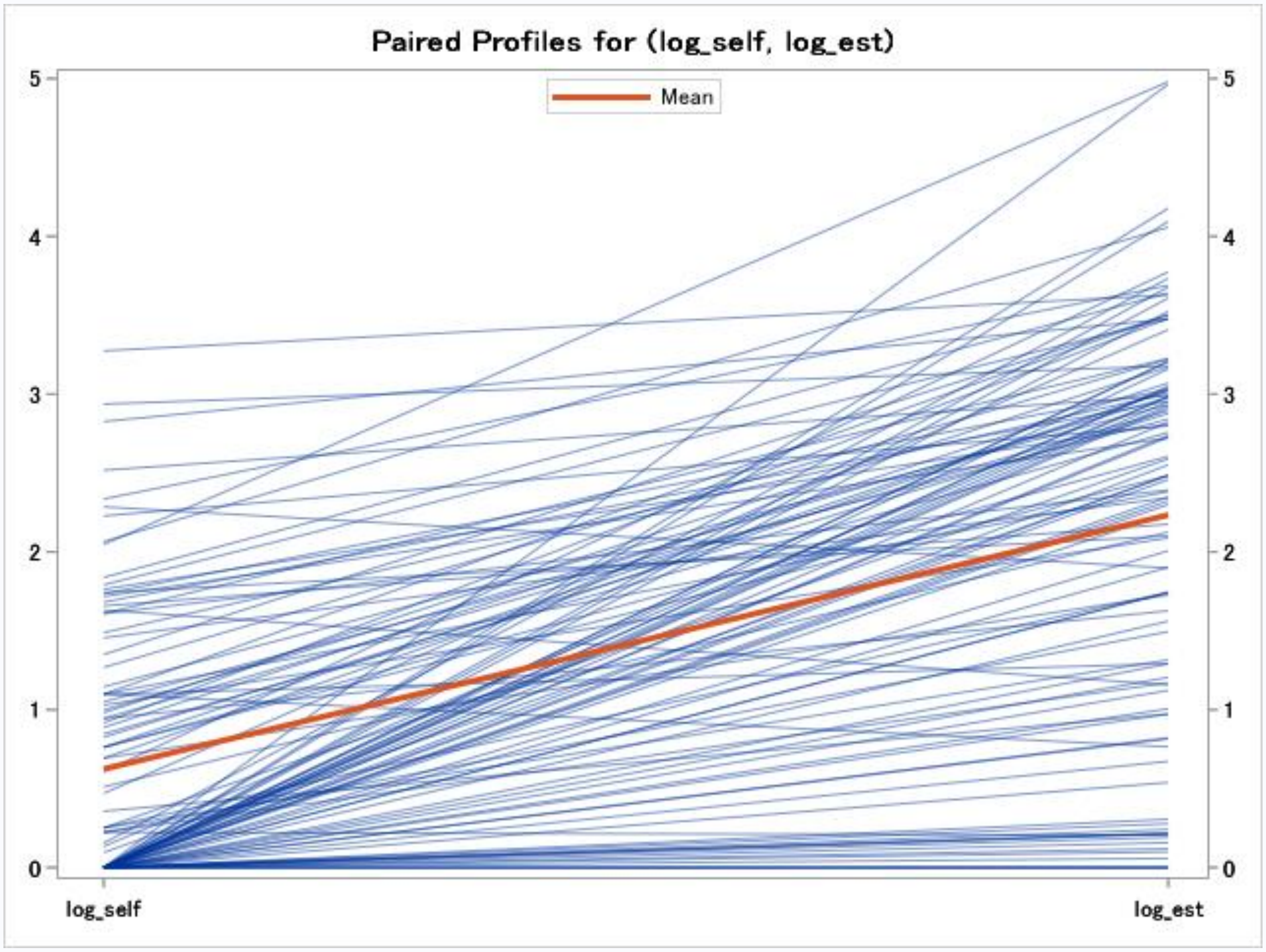
Paired Profiles for (log_self, log_est)
Mean
5
4
3
2
1
0
log_self
log_est

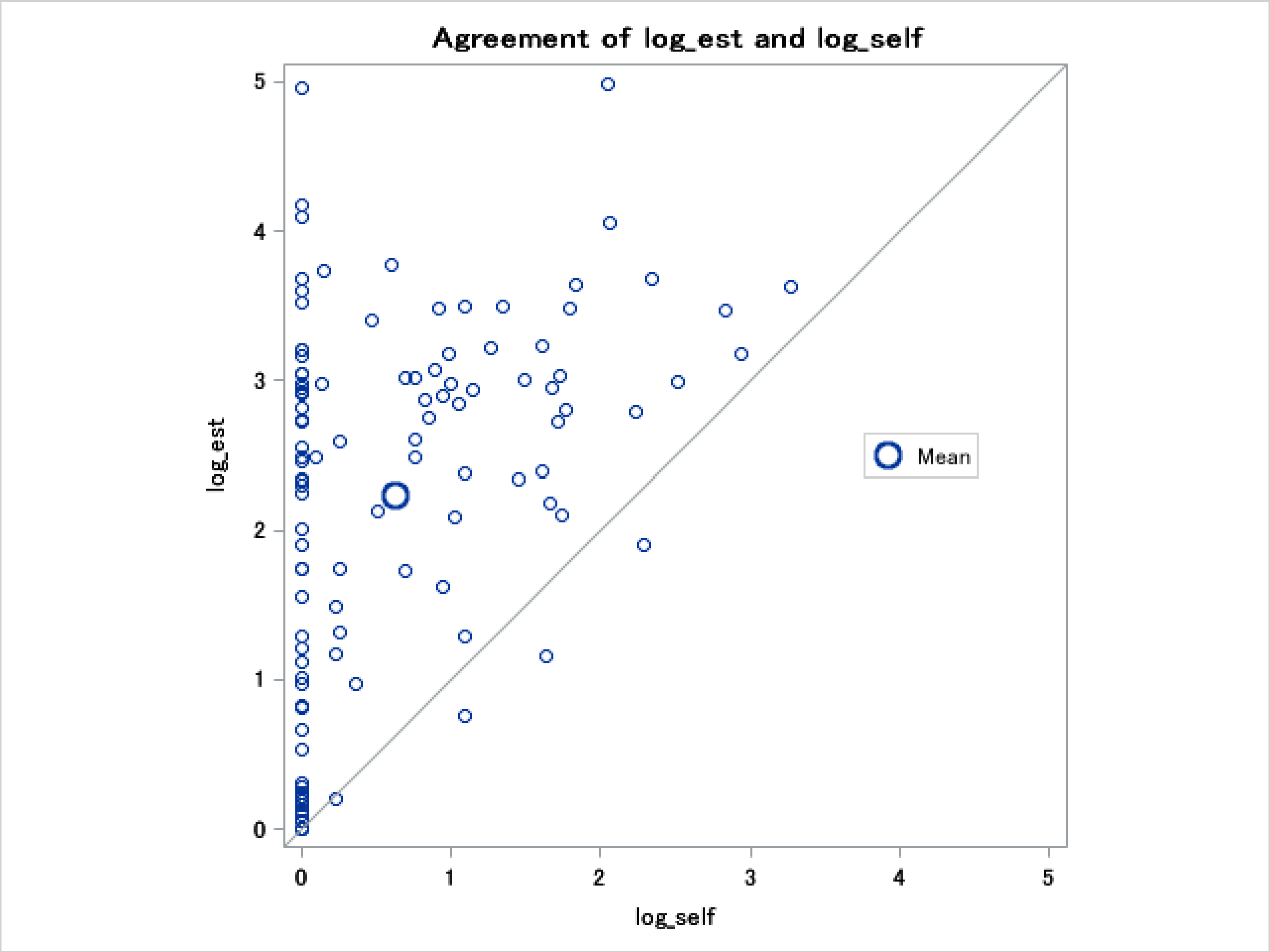
Agreement of log_est and log_self
log_est
log_self
Mean
0
1
2
3
4
5
0
1
2
3
4
5

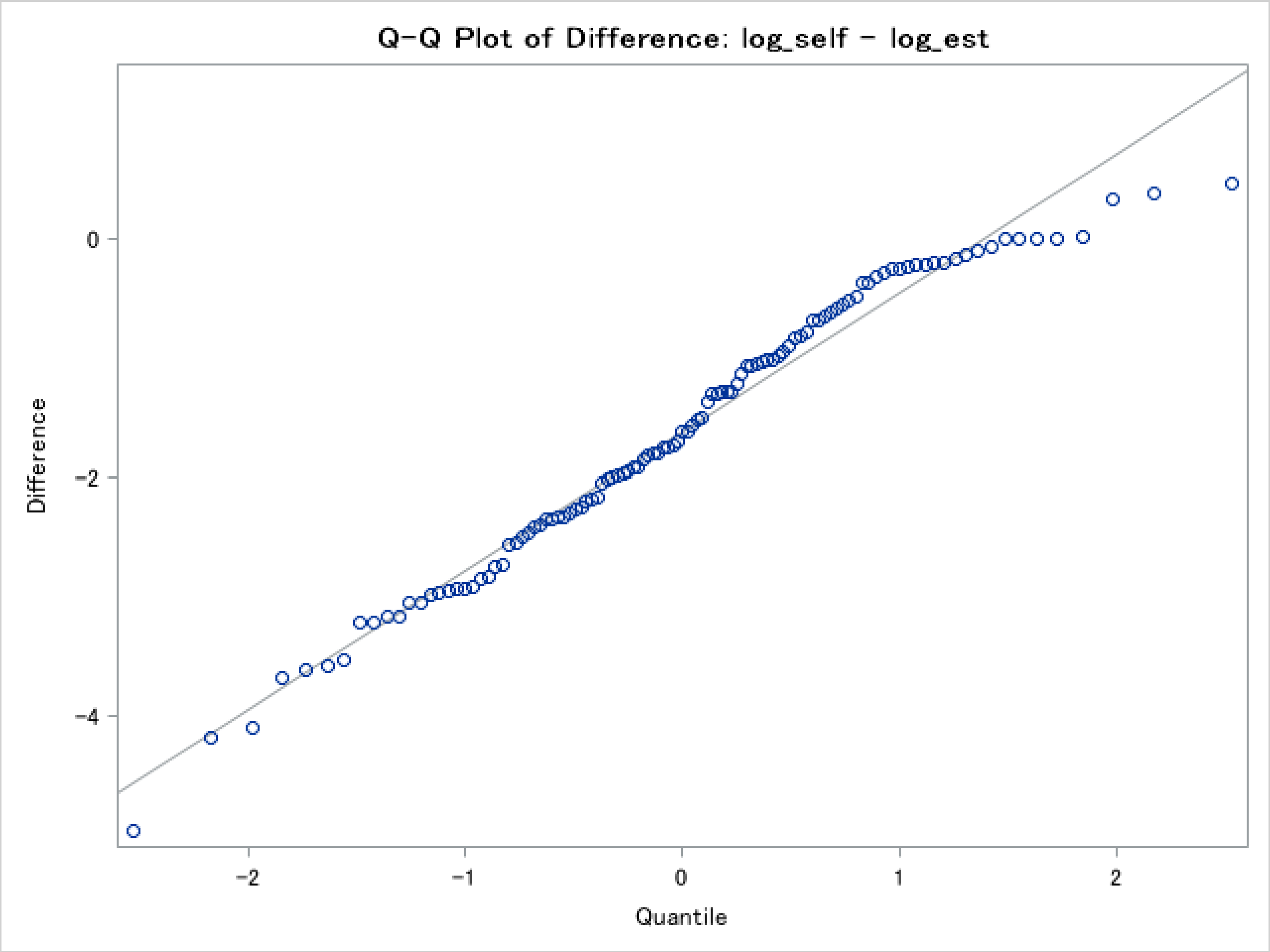

Q-Q Plot of Difference: log_self - log_est
Difference
0
-2
-4
-2
-1
0
1
2
Quantile

log transformed CPD variables then conduct paired t-test

The TTEST Procedure

Difference: log_self - log_est
AVISIT=week 6 TRTA=A ARM=2.4 mg/g

N	Mean	Std Dev	Std Err	Minimum	Maximum
106	-1.5826	1.0160	0.0987	-3.8981	0.5866

Mean	95% CL Mean		Std Dev	95% CL Std Dev	
-1.5826	-1.7783	-1.3869	1.0160	0.8952	1.1748

DF	t Value	Pr > |t|
105	-16.04	<.0001

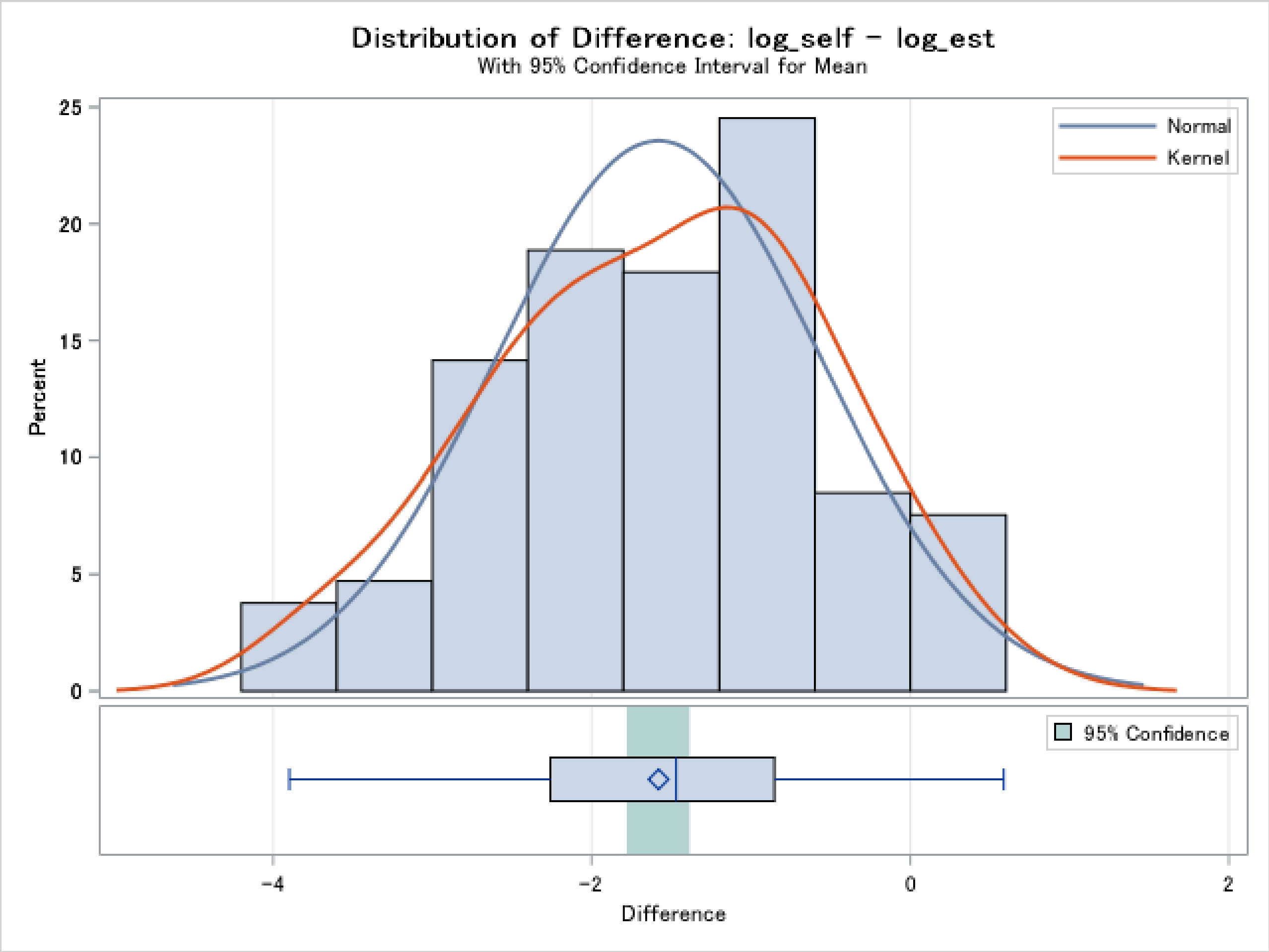

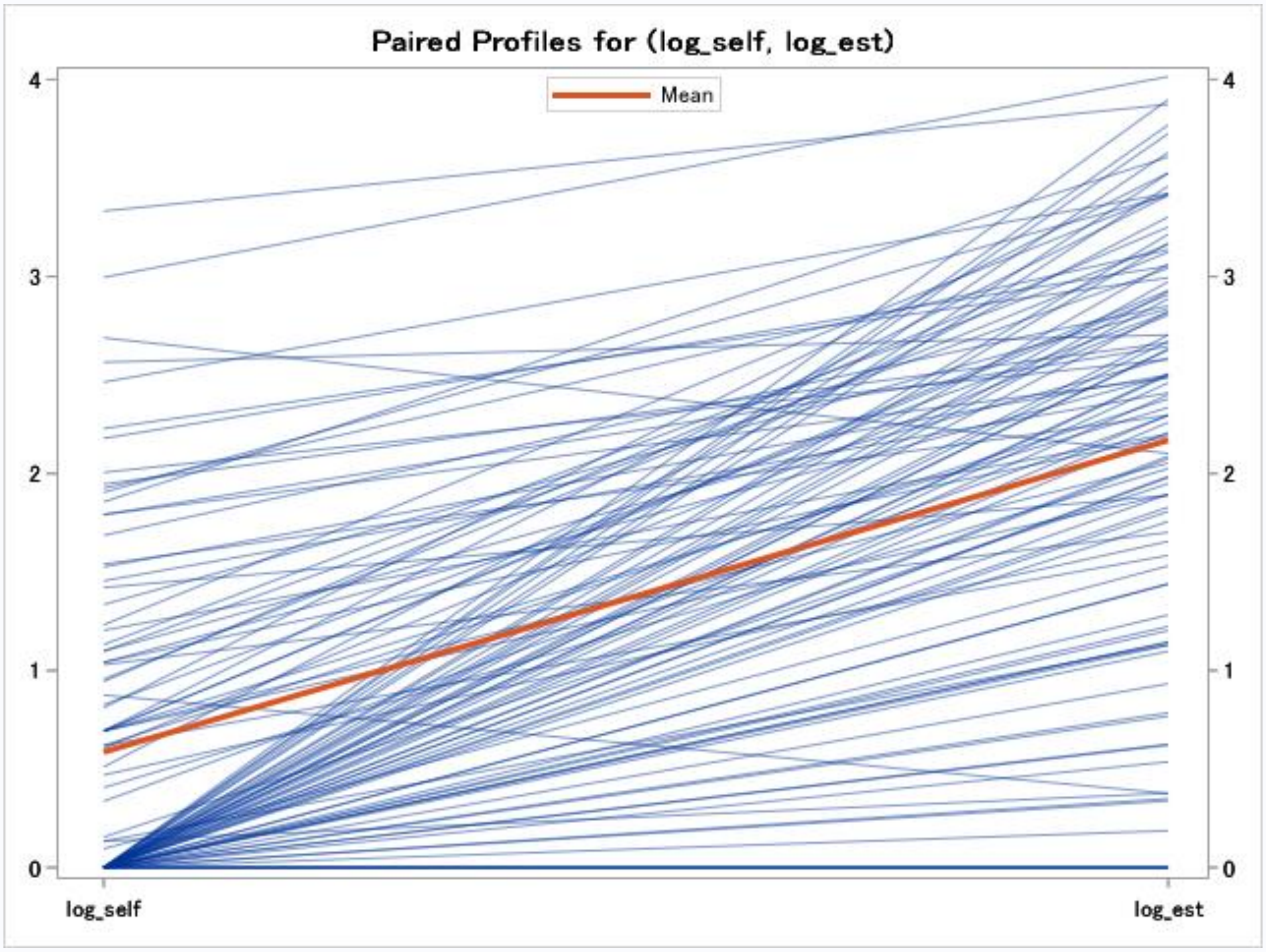
Paired Profiles for (log_self, log_est)
Mean
0
1
2
3
4
log_self
log_est

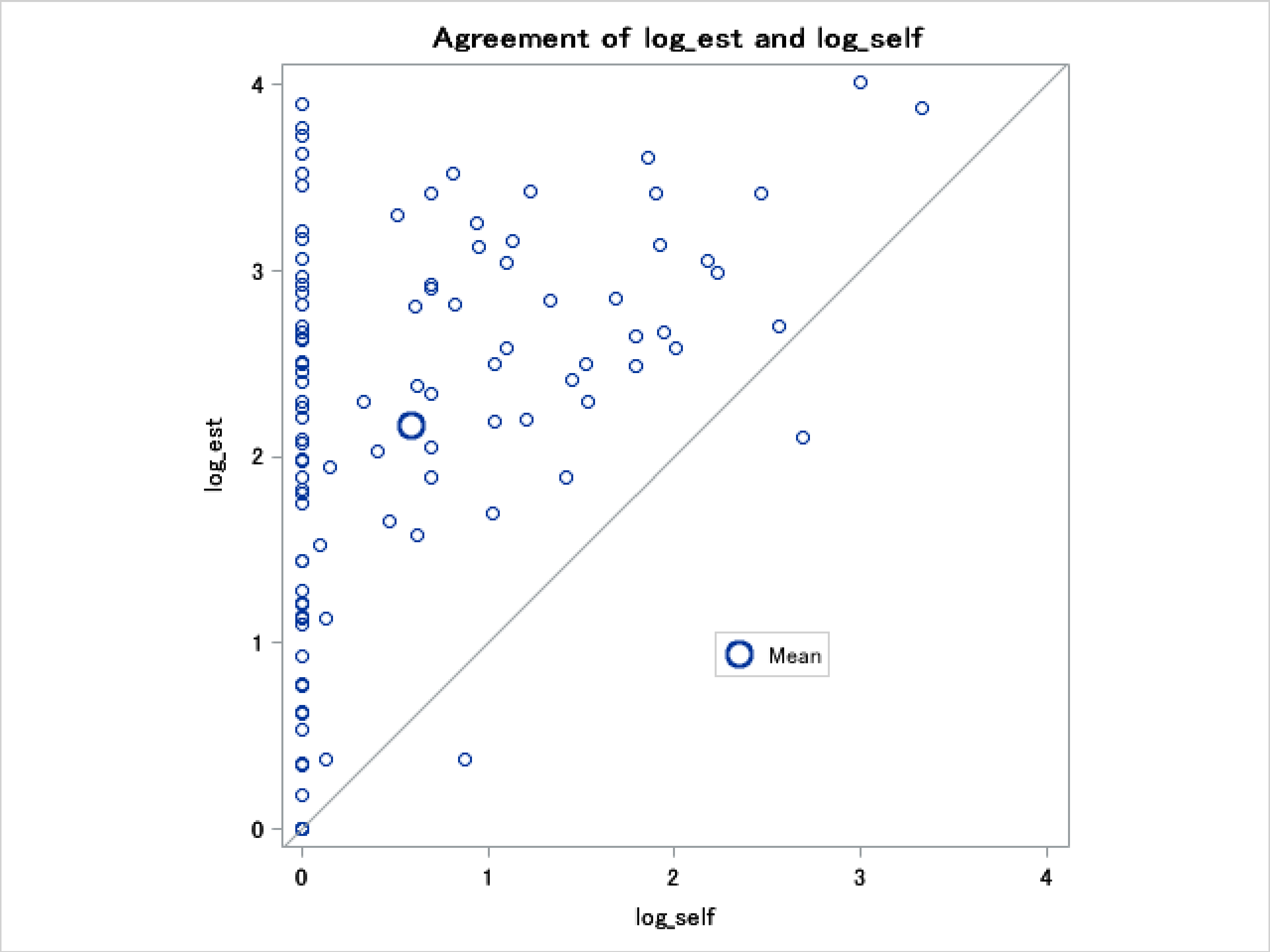
Agreement of log_est and log_self
log_est
log_self
Mean
0
1
2
3
4
0
1
2
3
4

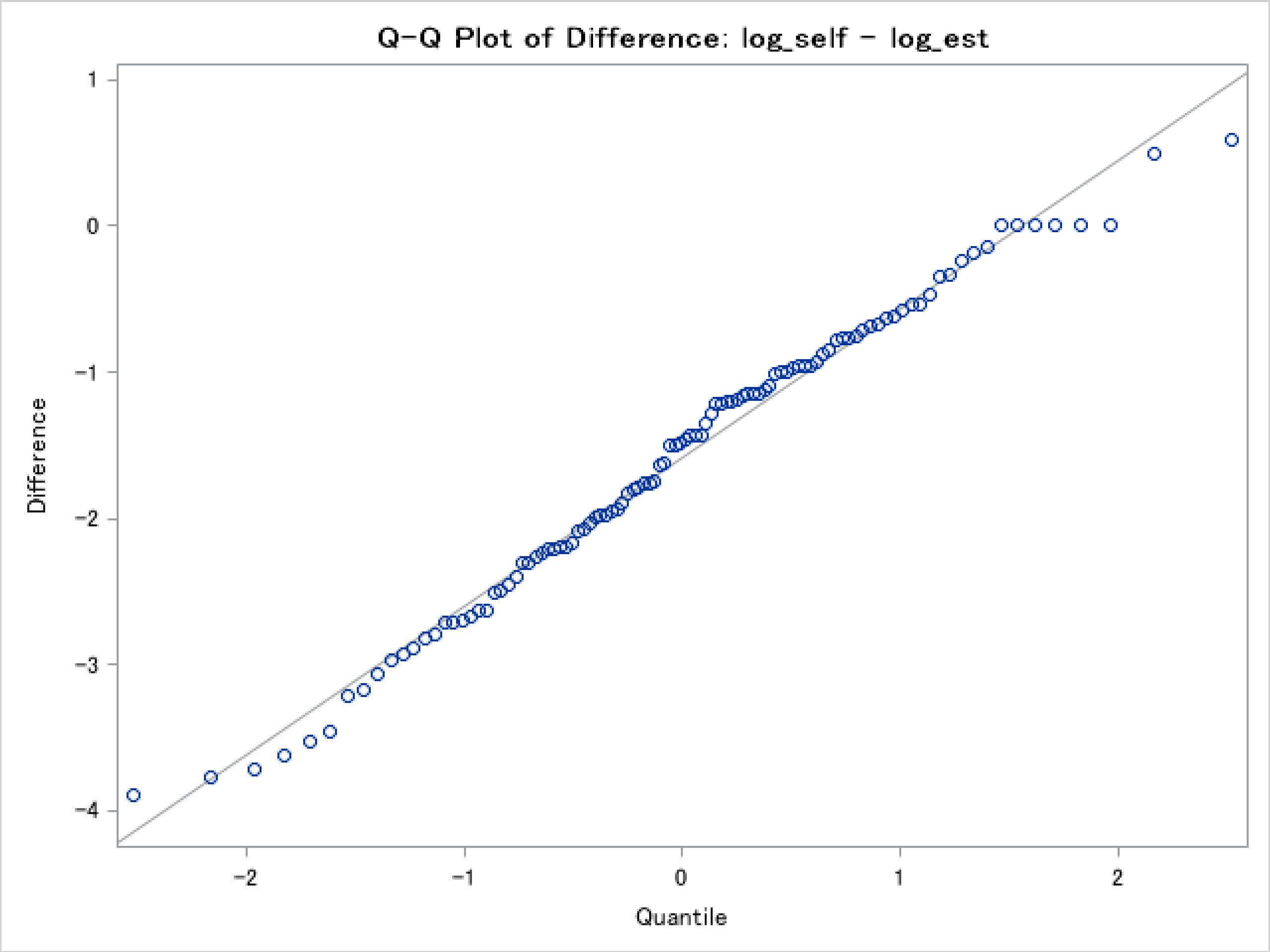
Q-Q Plot of Difference: log_self - log_est
1
0
−1
−2
−3
−4
Difference
−2
−1
0
1
2
Quantile

log transformed CPD variables then conduct paired t-test

The TTEST Procedure

Difference: log_self - log_est
AVISIT=week 6 TRTA=B ARM=0.4 mg/g (HT)

N	Mean	Std Dev	Std Err	Minimum	Maximum
113	-1.6236	1.1382	0.1071	-3.8053	0.2024

Mean	95% CL Mean		Std Dev	95% CL Std Dev	
-1.6236	-1.8358	-1.4115	1.1382	1.0066	1.3096

DF	t Value	Pr > |t|
112	-15.16	<.0001

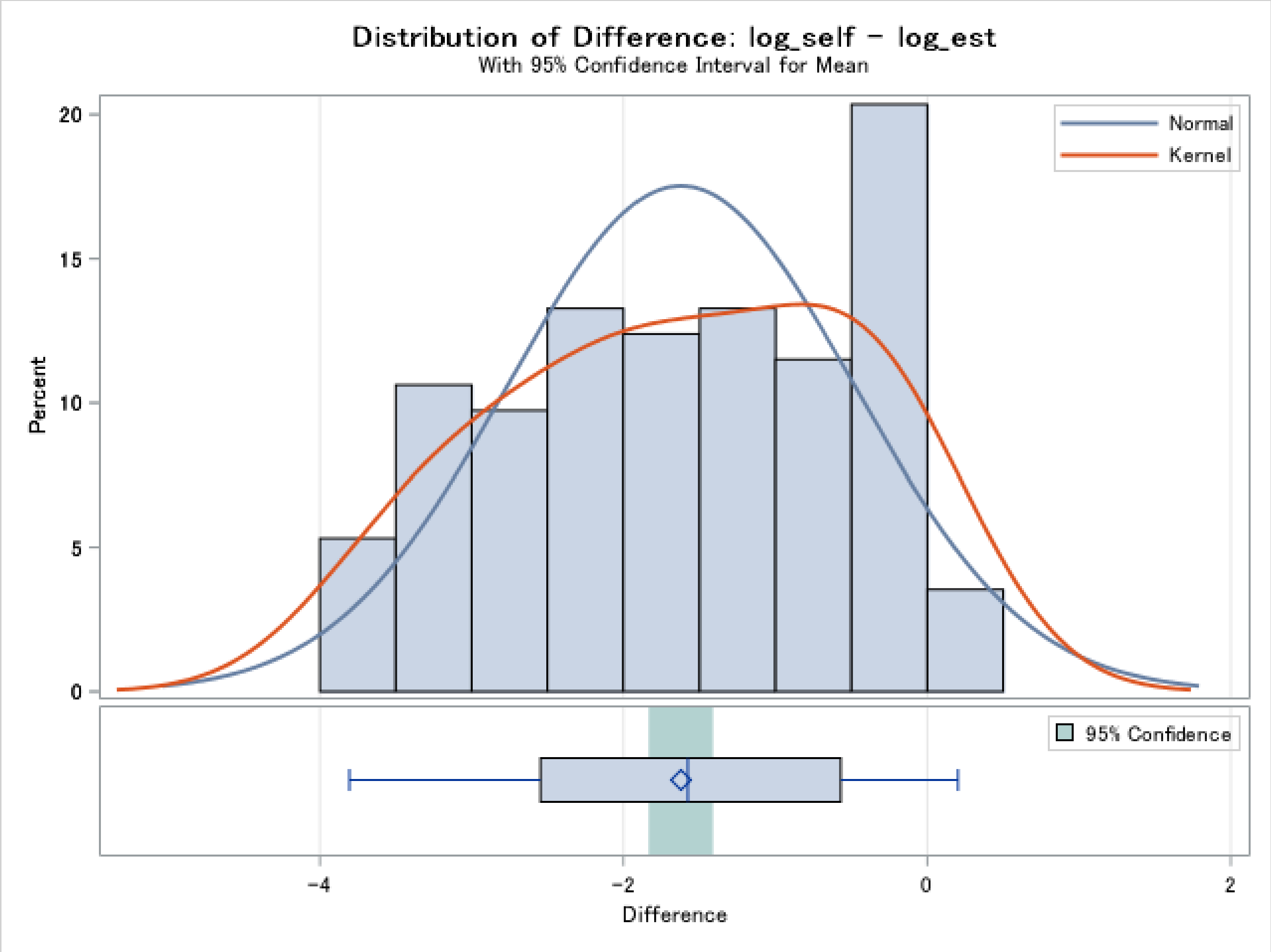

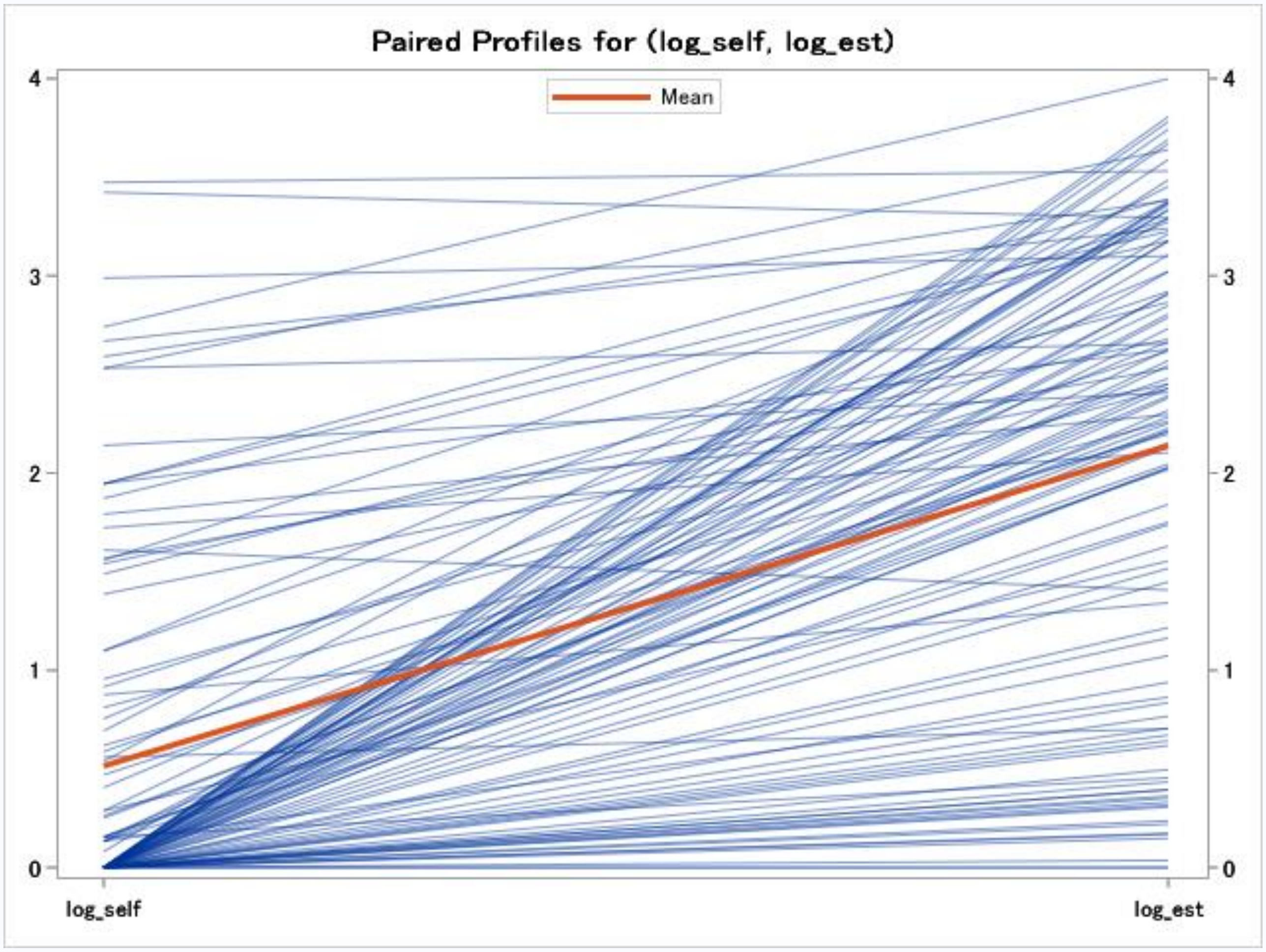

Paired Profiles for (log_self, log_est)
Mean
4
3
2
1
0
log_self
log_est

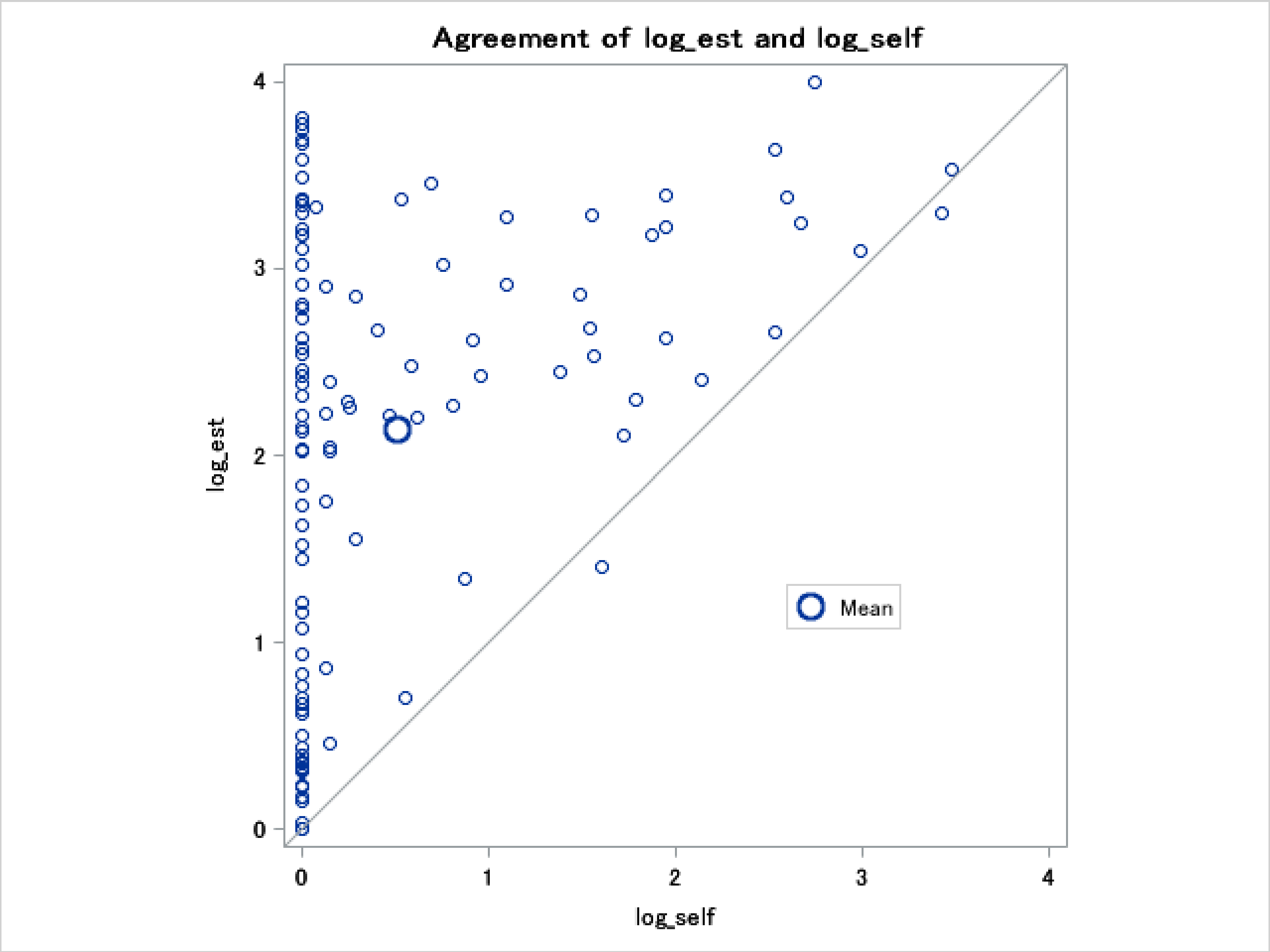

Agreement of log_est and log_self
log_est
log_self
Mean
0
1
2
3
4

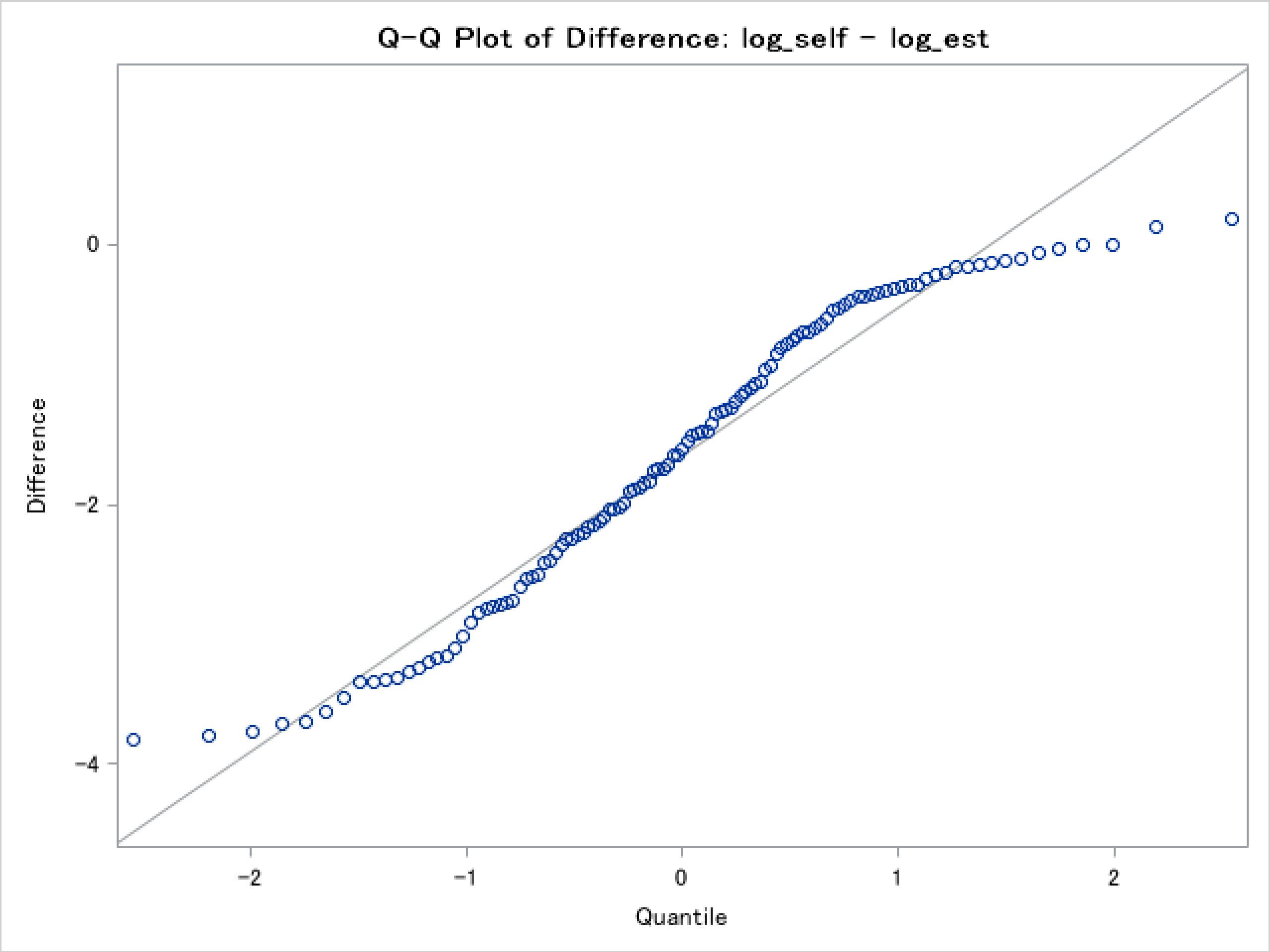

Q-Q Plot of Difference: log_self - log_est
0
-2
-4
Difference
-2
-1
0
1
2
Quantile

log transformed CPD variables then conduct paired t-test

The TTEST Procedure

Difference: log_self - log_est
AVISIT=week 6 TRTA=D ARM=1.3 mg/g

N	Mean	Std Dev	Std Err	Minimum	Maximum
106	-1.8195	1.0637	0.1033	-4.4231	0.3371

Mean	95% CL Mean		Std Dev	95% CL Std Dev	
-1.8195	-2.0243	-1.6146	1.0637	0.9373	1.2300

DF	t Value	Pr > |t|
105	-17.61	<.0001

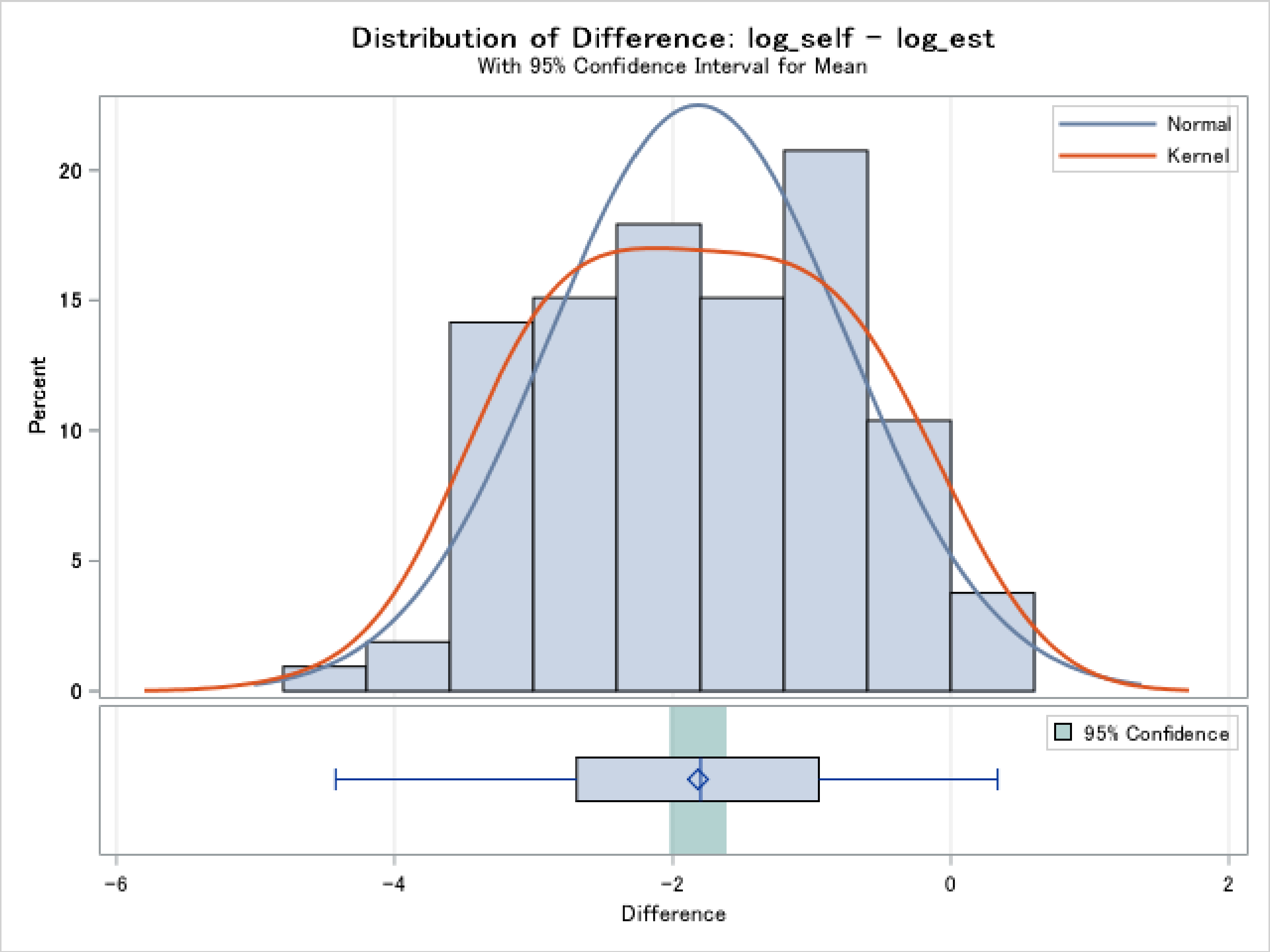

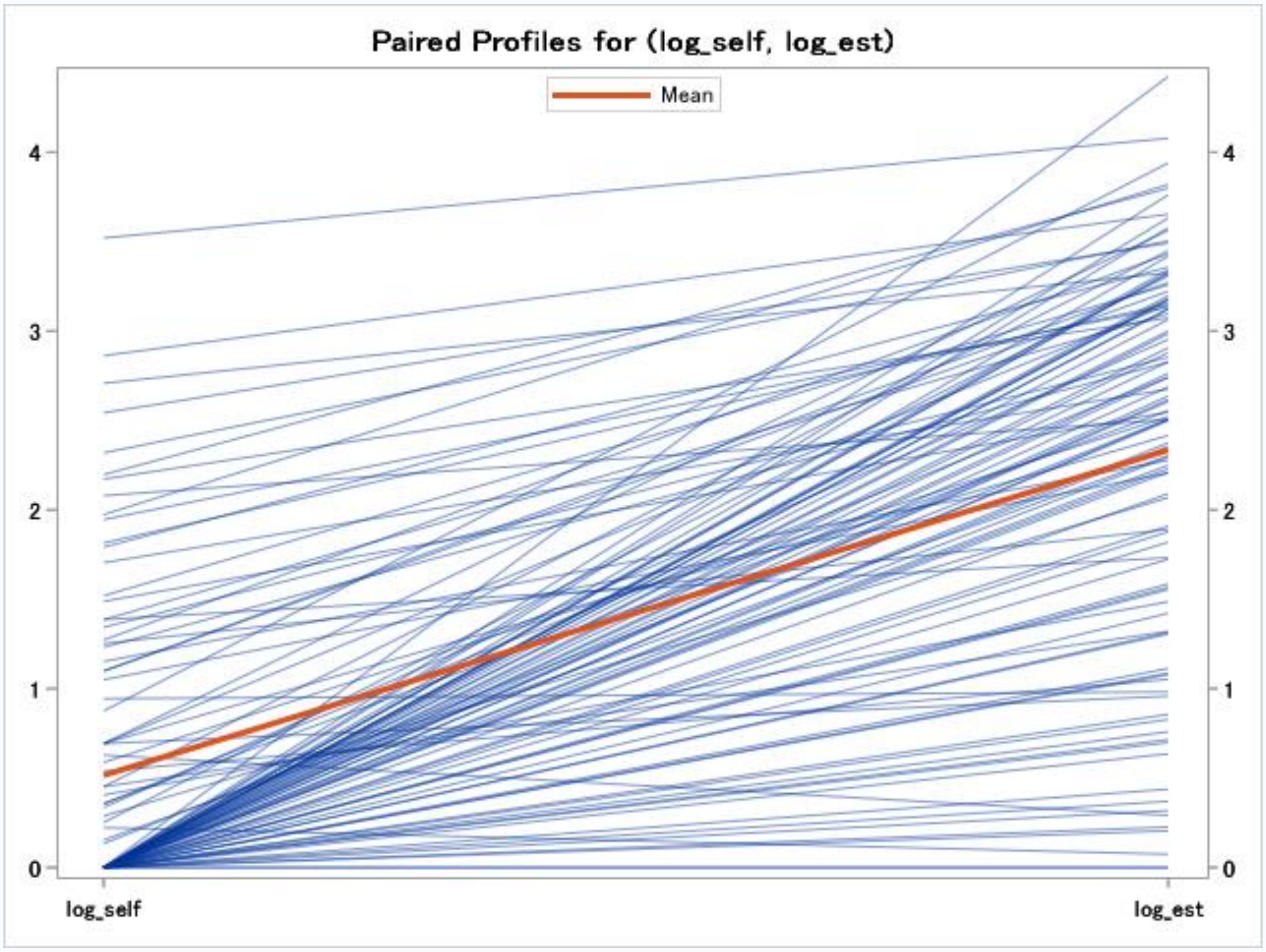

Paired Profiles for (log_self, log_est)
Mean
0
1
2
3
4
log_self
log_est

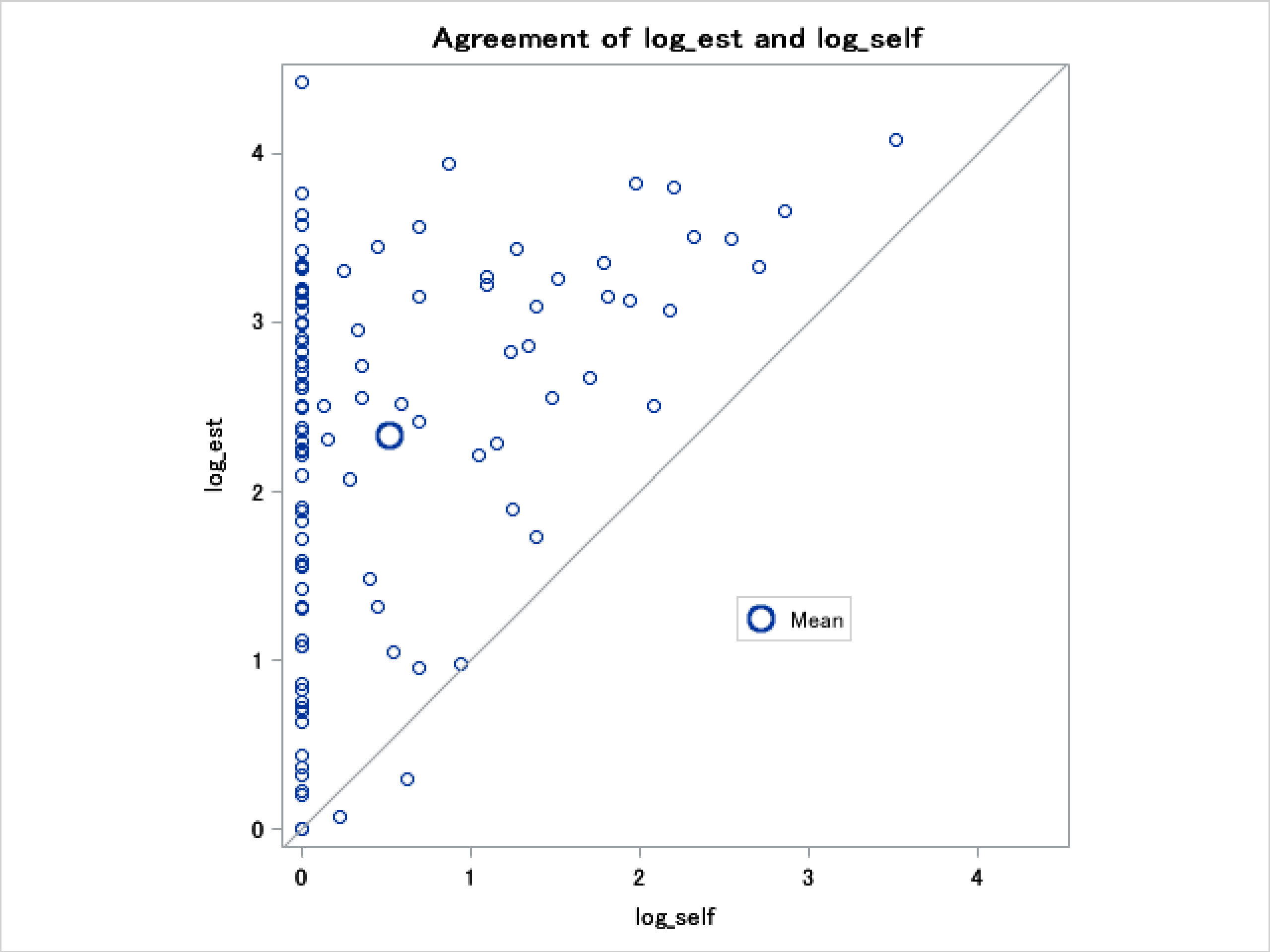

Agreement of log_est and log_self
log_est
4
3
2
1
0
0
1
2
3
4
log_self
Mean

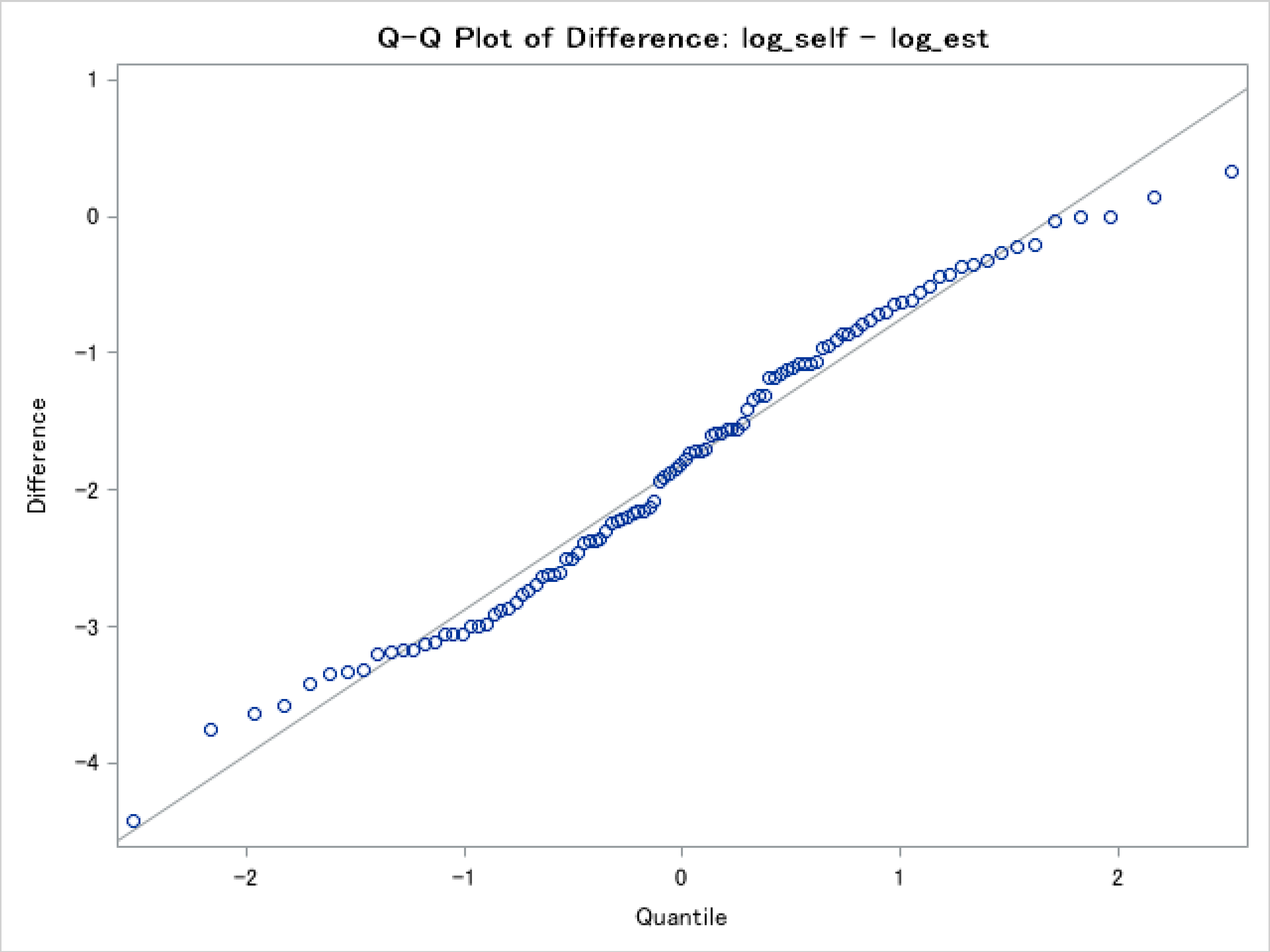
Q-Q Plot of Difference: log_self - log_est
Difference
Quantile
1
0
−1
−2
−3
−4
−2
−1
0
1
2

log transformed CPD variables then conduct paired t-test

The TTEST Procedure

Difference: log_self - log_est
AVISIT=week 6 TRTA=F ARM=5.2 mg/g

N	Mean	Std Dev	Std Err	Minimum	Maximum
106	-1.6479	1.0344	0.1005	-3.5088	0.6931

Mean	95% CL Mean		Std Dev	95% CL Std Dev	
-1.6479	-1.8472	-1.4487	1.0344	0.9115	1.1961

DF	t Value	Pr > |t|
105	-16.40	<.0001

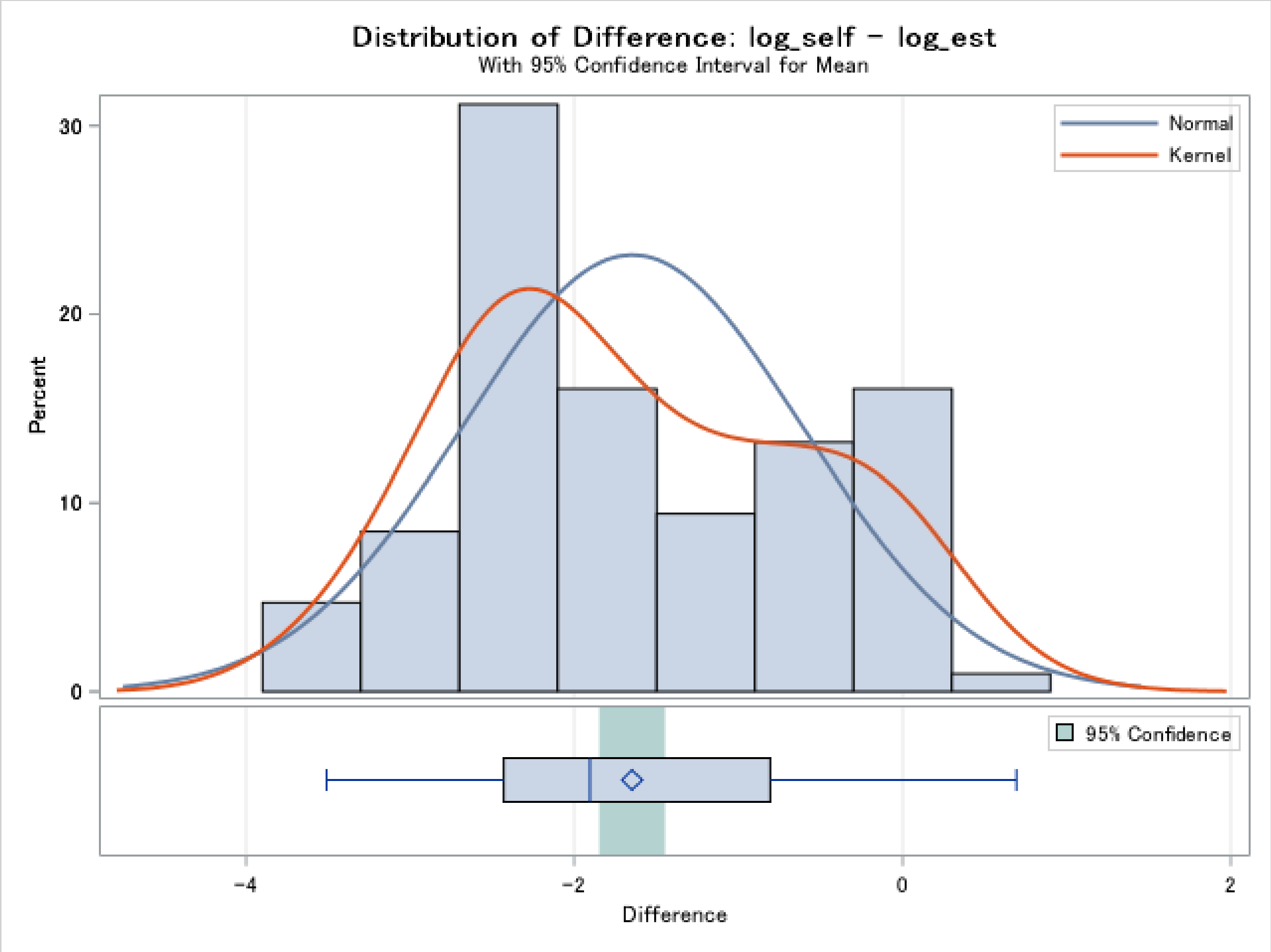

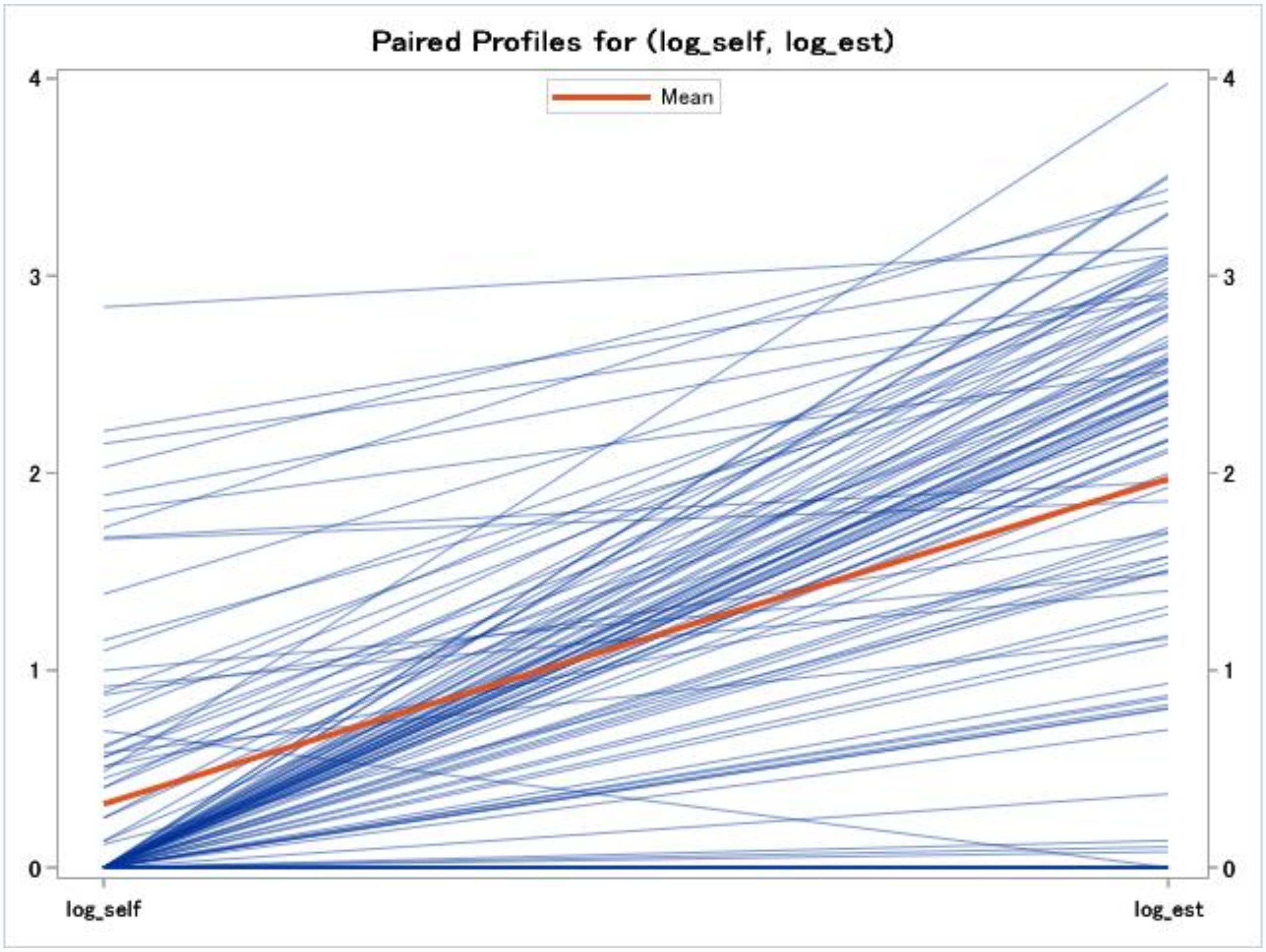
Paired Profiles for (log_self, log_est)
Mean
4
3
2
1
0
log_self
log_est

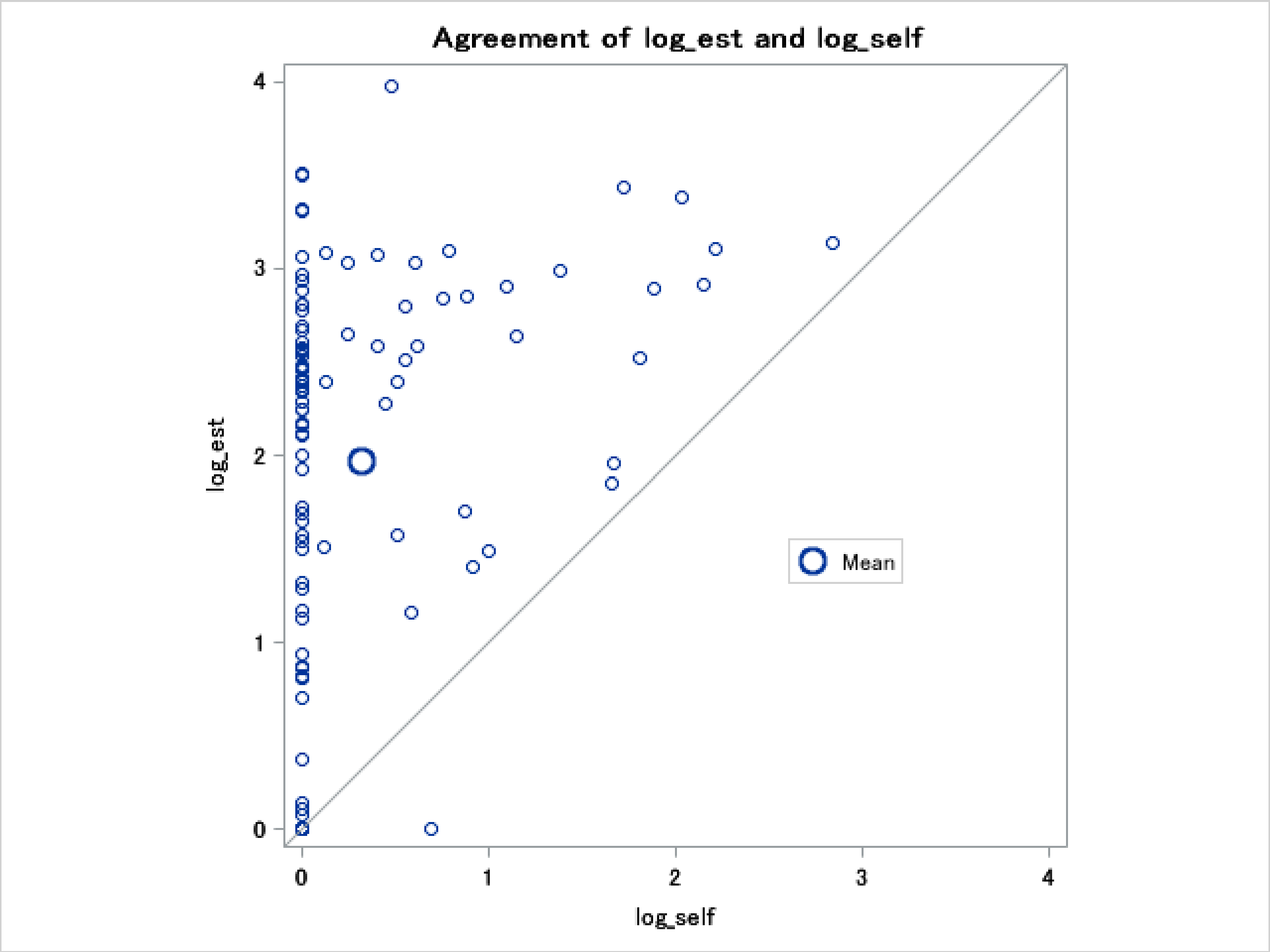

Agreement of log_est and log_self
log_est
log_self
0
1
2
3
4
0
1
2
3
4
Mean

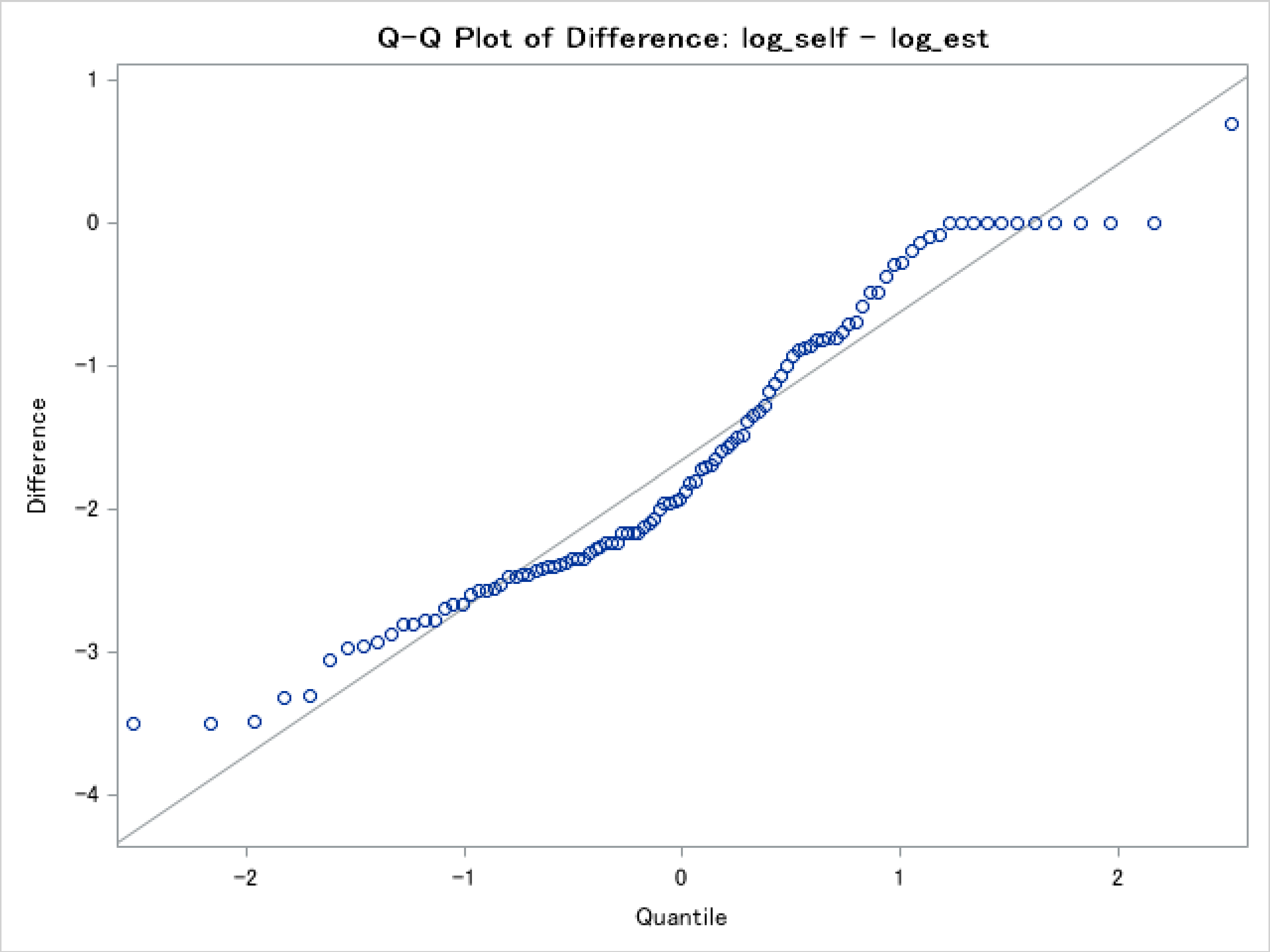

Q-Q Plot of Difference: log_self - log_est
1
0
−1
−2
−3
−4
Difference
−2
−1
0
1
2
Quantile

log transformed CPD variables then conduct paired t-test

The TTEST Procedure

Difference: log_self - log_est
AVISIT=week 6 TRTA=G ARM=0.4 mg/g

N	Mean	Std Dev	Std Err	Minimum	Maximum
104	-1.8274	1.2049	0.1181	-4.5129	0.7783

Mean	95% CL Mean		Std Dev	95% CL Std Dev	
-1.8274	-2.0617	-1.5931	1.2049	1.0604	1.3953

DF	t Value	Pr > |t|
103	-15.47	<.0001

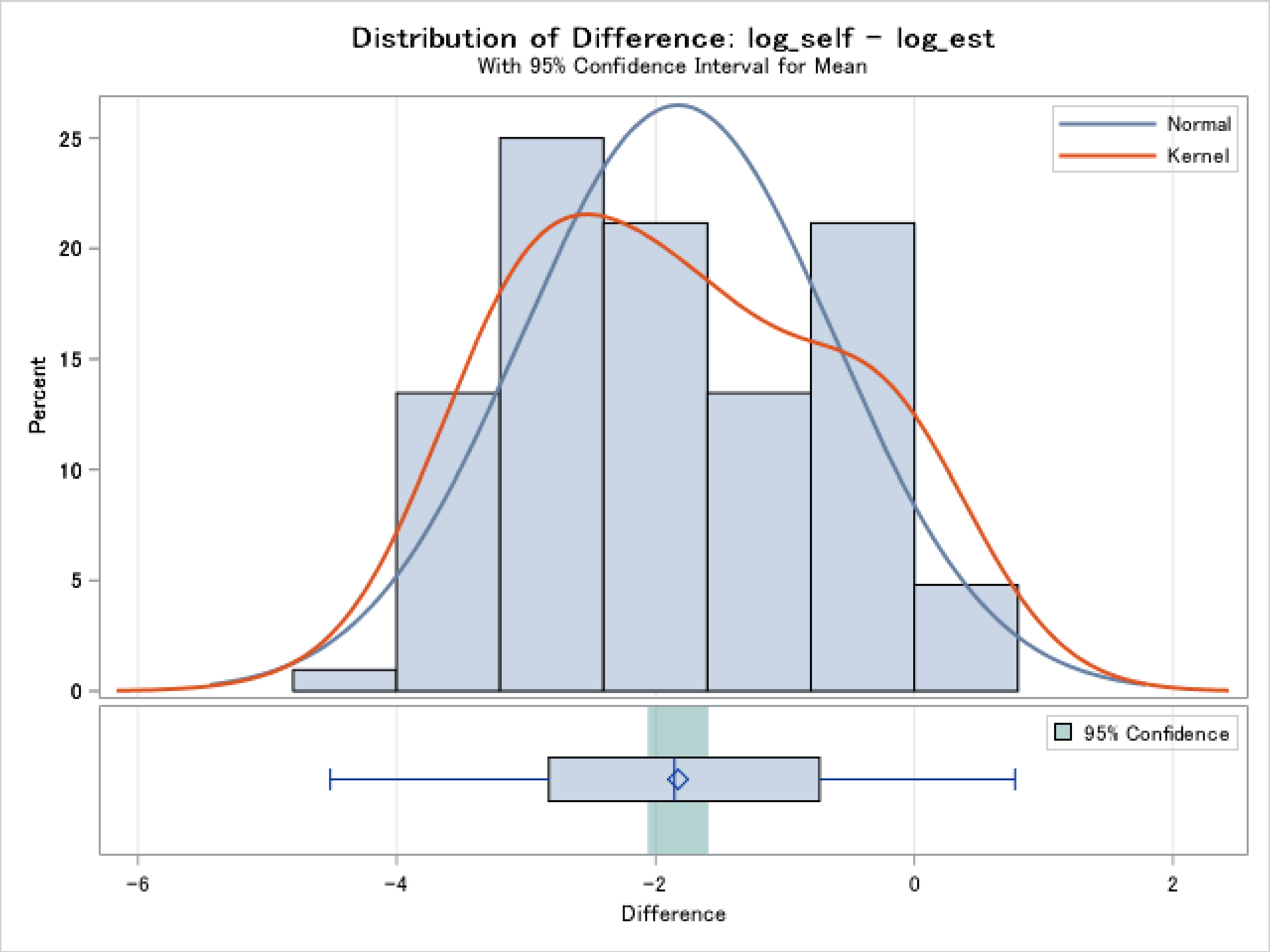

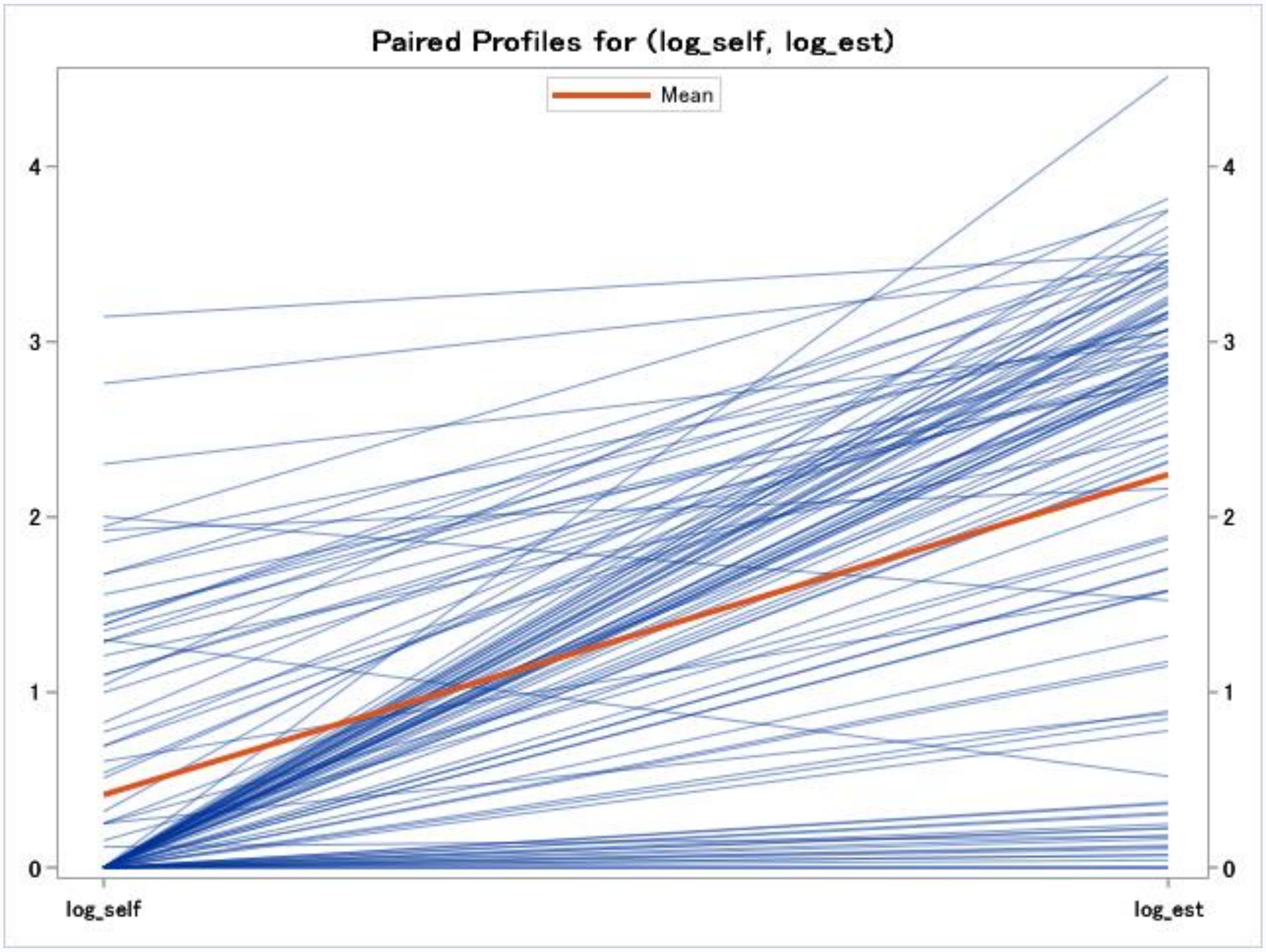

Paired Profiles for (log_self, log_est)
Mean
4
3
2
1
0
log_self
log_est

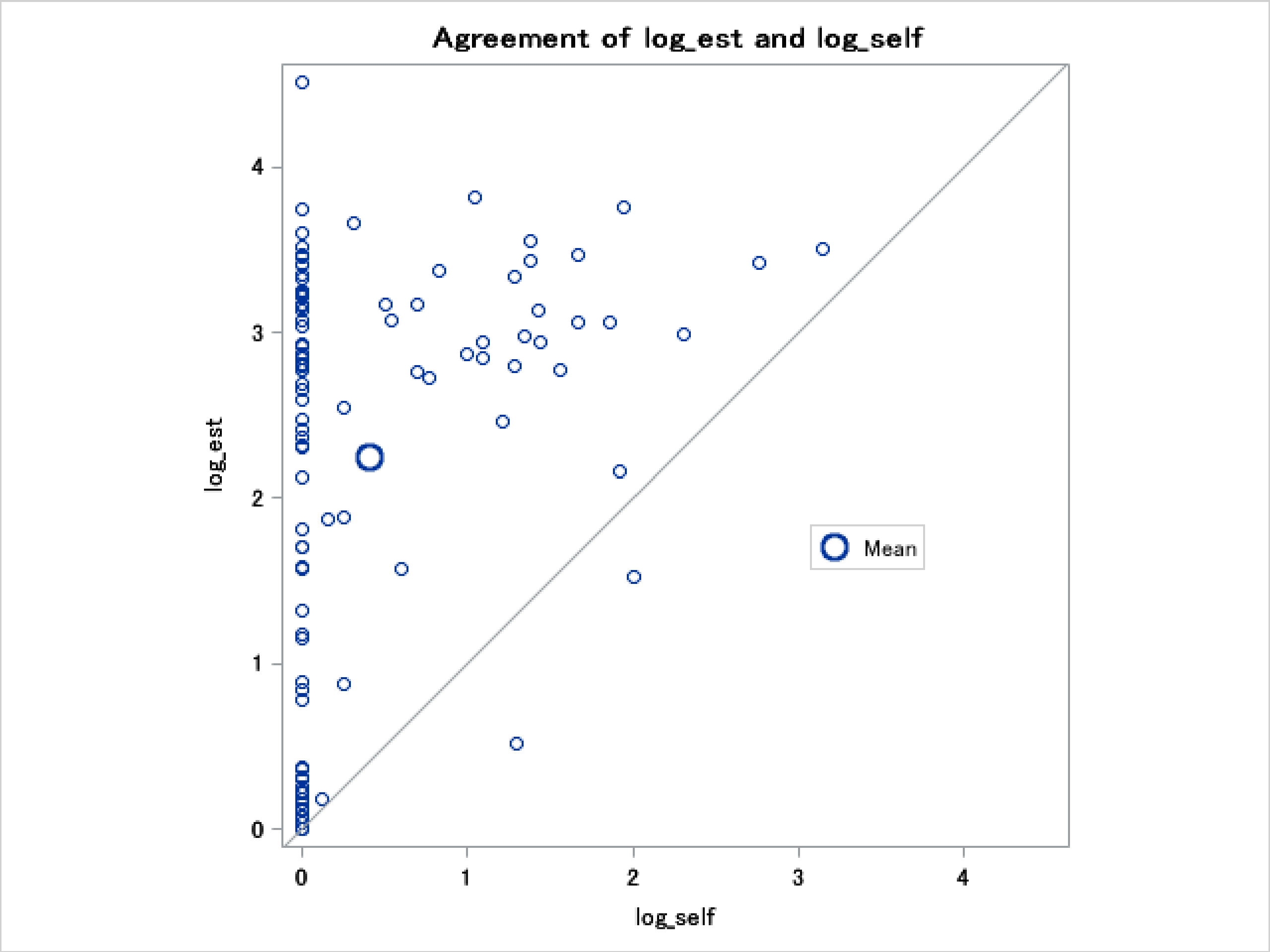

Agreement of log_est and log_self
log_est
log_self
0
1
2
3
4
Mean

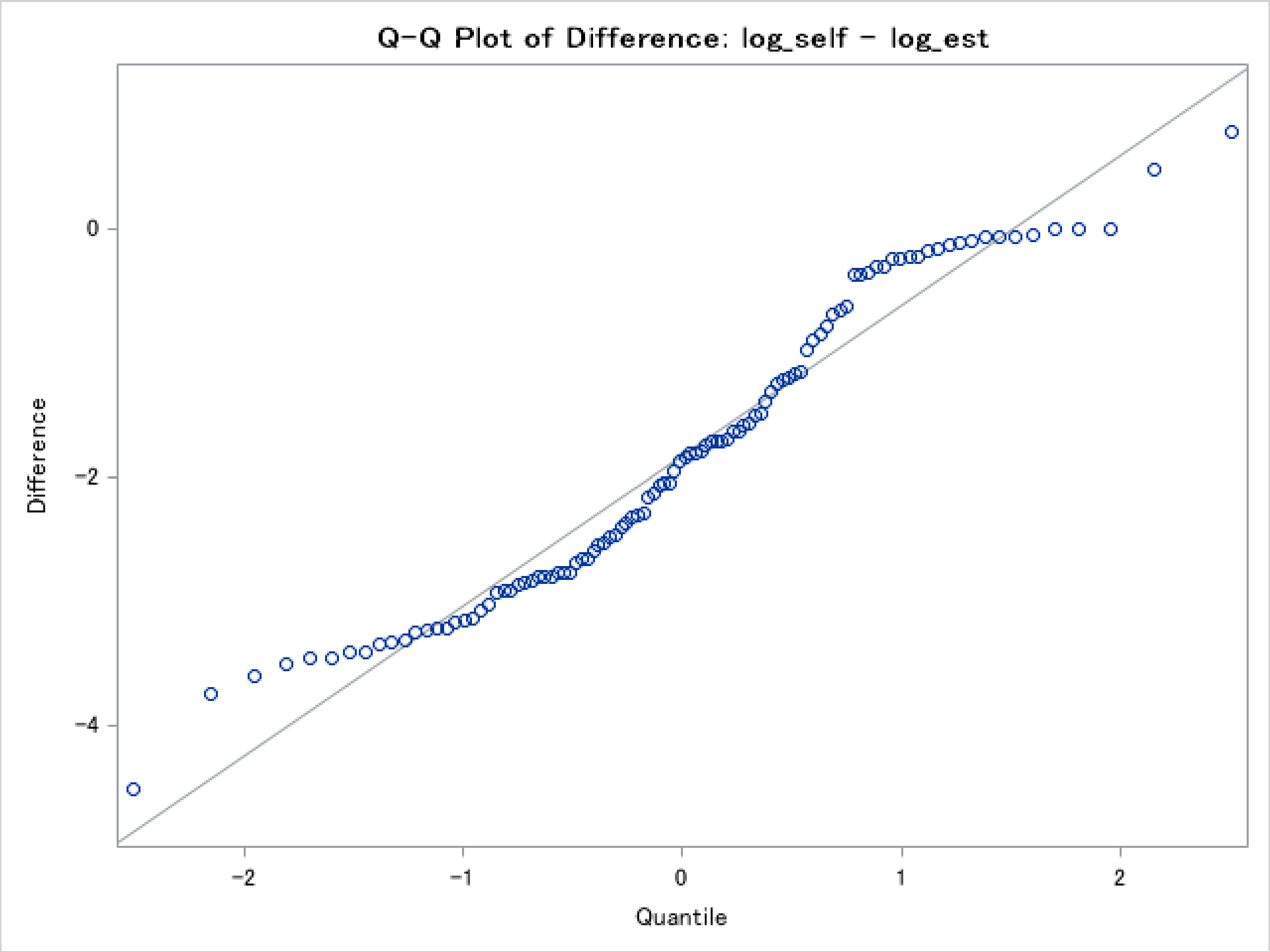

Q-Q Plot of Difference: log_self - log_est
0
-2
-4
Difference
-2
-1
0
1
2
Quantile

Wilcoxon signed-rank test on variable DIFF

The UNIVARIATE Procedure
Variable: diff
AVISIT=week 2 TRTA=A ARM=2.4 mg/g

Moments

**N**	110	**Sum Weights**	110
**Mean**	8.84619063	**Sum Observations**	973.080969
**Std Deviation**	9.44091103	**Variance**	89.130801
**Skewness**	1.77187675	**Kurtosis**	3.71437257
**Uncorrected SS**	18323.3171	**Corrected SS**	9715.25731
**Coeff Variation**	106.722898	**Std Error Mean**	0.90015555

Basic Statistical Measures

Location		Variability	
**Mean**	8.846191	**Std Deviation**	9.44091
**Median**	5.401497	**Variance**	89.13080
**Mode**	0.000000	**Range**	52.83189
		**Interquartile Range**	10.62056

Tests for Location: Mu0=0

Test		Statistic	p Value	
**Student's t**	**t**	9.827402	**Pr > |t|**	<.0001
**Sign**	**M**	51	**Pr >= |M|**	<.0001
**Signed Rank**	**S**	2893	**Pr >= |S|**	<.0001

Quantiles (Definition 5)

Level	Quantile
**100% Max**	49.9985543
**99%**	41.4735207
**95%**	27.8323782
**90%**	22.9259769

Quantiles (Definition 5)

Level	Quantile
75% Q3	13.0621255
50% Median	5.4014975
25% Q1	2.4415655
10%	0.6085307
5%	0.0524603
1%	−1.4652116
0% Min	−2.8333333

Extreme Observations

Lowest		Highest	
Value	Obs	Value	Obs
−2.833333	69	30.2937	53
−1.465212	46	30.6443	101
−0.142857	67	34.6212	18
0.000000	51	41.4735	76
0.000000	11	49.9986	12

Missing Values

Missing Value	Count	Percent Of	
		All Obs	Missing Obs
.	9	7.56	100.00

Wilcoxon signed-rank test on variable DIFF

The UNIVARIATE Procedure
Variable: diff
AVISIT=week 2 TRTA=B ARM=0.4 mg/g (HT)

Moments

**N**	115	**Sum Weights**	115
**Mean**	9.97687143	**Sum Observations**	1147.34021
**Std Deviation**	12.7413946	**Variance**	162.343135
**Skewness**	2.73968782	**Kurtosis**	10.3972301
**Uncorrected SS**	29953.9832	**Corrected SS**	18507.1174
**Coeff Variation**	127.709319	**Std Error Mean**	1.18814117

Basic Statistical Measures

Location		Variability	
**Mean**	9.976871	**Std Deviation**	12.74139
**Median**	6.006274	**Variance**	162.34314
**Mode**	.	**Range**	87.93005
		**Interquartile Range**	11.19679

Tests for Location: Mu0=0

Test	Statistic		p Value	
**Student's t**	**t**	8.397042	**Pr > |t|**	<.0001
**Sign**	**M**	53.5	**Pr >= |M|**	<.0001
**Signed Rank**	**S**	3239	**Pr >= |S|**	<.0001

Quantiles (Definition 5)

Level	Quantile
**100% Max**	82.376452
**99%**	55.932908
**95%**	35.283371
**90%**	23.438946

Quantiles (Definition 5)

Level	Quantile
75% Q3	12.807303
50% Median	6.006274
25% Q1	1.610513
10%	0.382487
5%	0.105128
1%	−1.951812
0% Min	−5.553603

Extreme Observations

Lowest		Highest	
Value	Obs	Value	Obs
−5.5536026	193	38.9080	237
−1.9518124	238	42.6219	160
−0.2740848	235	51.9079	224
−0.1900604	143	55.9329	203
0.0384768	240	82.3765	175

Missing Values

Missing Value	Count	Percent Of	
		All Obs	Missing Obs
.	8	6.50	100.00

Wilcoxon signed-rank test on variable DIFF

The UNIVARIATE Procedure
Variable: diff
AVISIT=week 2 TRTA=D ARM=1.3 mg/g

Moments

N	110	Sum Weights	110
Mean	11.5813328	Sum Observations	1273.9466
Std Deviation	13.9831564	Variance	195.528662
Skewness	2.03815455	Kurtosis	5.86233293
Uncorrected SS	36066.6237	Corrected SS	21312.6241
Coeff Variation	120.73875	Std Error Mean	1.33324165

Basic Statistical Measures

Location		Variability	
Mean	11.58133	Std Deviation	13.98316
Median	8.11764	Variance	195.52866
Mode	0.00000	Range	82.68271
		Interquartile Range	15.19399

Tests for Location: Mu0=0

Test	Statistic		p Value	
Student's t	t	8.686597	Pr > |t|	<.0001
Sign	M	47	Pr >= |M|	<.0001
Signed Rank	S	2645.5	Pr >= |S|	<.0001

Quantiles (Definition 5)

Level	Quantile
100% Max	74.3285208
99%	70.1575886
95%	32.7387640
90%	27.7129505

Quantiles (Definition 5)

Level	Quantile
75% Q3	16.6410449
50% Median	8.1176401
25% Q1	1.4470552
10%	0.0270828
5%	−1.0584213
1%	−7.1009832
0% Min	−8.3541890

Extreme Observations

Lowest		Highest	
Value	Obs	Value	Obs
−8.35419	340	38.1035	354
−7.10098	297	44.5436	300
−2.50294	327	57.1356	311
−2.35723	317	70.1576	335
−1.22222	274	74.3285	260

Missing Values

Missing Value	Count	Percent Of	
		All Obs	Missing Obs
.	9	7.56	100.00

Wilcoxon signed-rank test on variable DIFF

The UNIVARIATE Procedure
Variable: diff
AVISIT=week 2 TRTA=F ARM=5.2 mg/g

Moments

**N**	110	**Sum Weights**	110
**Mean**	9.56482393	**Sum Observations**	1052.13063
**Std Deviation**	24.9733029	**Variance**	623.665856
**Skewness**	8.63738174	**Kurtosis**	83.6900476
**Uncorrected SS**	78043.0226	**Corrected SS**	67979.5783
**Coeff Variation**	261.09527	**Std Error Mean**	2.381111

Basic Statistical Measures

Location		Variability	
**Mean**	9.564824	**Std Deviation**	24.97330
**Median**	4.990132	**Variance**	623.66586
**Mode**	0.000000	**Range**	256.09575
		**Interquartile Range**	7.91177

Tests for Location: Mu0=0

Test	Statistic		p Value	
**Student's t**	**t**	4.016958	**Pr > |t|**	0.0001
**Sign**	**M**	41.5	**Pr >= |M|**	<.0001
**Signed Rank**	**S**	2282.5	**Pr >= |S|**	<.0001

Quantiles (Definition 5)

Level	Quantile
**100% Max**	252.429087
**99%**	38.352420
**95%**	29.675964
**90%**	20.928572

Quantiles (Definition 5)

Level	Quantile
75% Q3	9.277964
50% Median	4.990132
25% Q1	1.366198
10%	0.000000
5%	−0.285714
1%	−2.857143
0% Min	−3.666667

Extreme Observations

Lowest		Highest	
Value	Obs	Value	Obs
−3.666667	436	32.3241	466
−2.857143	409	35.2060	370
−0.625000	373	38.0596	425
−0.491127	421	38.3524	467
−0.400000	386	252.4291	389

Missing Values

Missing Value	Count	Percent Of	
		All Obs	Missing Obs
.	12	9.84	100.00

Wilcoxon signed-rank test on variable DIFF

The UNIVARIATE Procedure
Variable: diff
AVISIT=week 2 TRTA=G ARM=0.4 mg/g

Moments

**N**	111	**Sum Weights**	111
**Mean**	14.6297096	**Sum Observations**	1623.89776
**Std Deviation**	21.4019633	**Variance**	458.044033
**Skewness**	3.94344345	**Kurtosis**	20.2114456
**Uncorrected SS**	74141.9963	**Corrected SS**	50384.8436
**Coeff Variation**	146.291102	**Std Error Mean**	2.03138446

Basic Statistical Measures

Location		Variability	
**Mean**	14.62971	**Std Deviation**	21.40196
**Median**	9.63652	**Variance**	458.04403
**Mode**	0.00000	**Range**	145.28413
		**Interquartile Range**	16.65964

Tests for Location: Mu0=0

Test	Statistic		p Value	
**Student's t**	**t**	7.201842	**Pr > |t|**	<.0001
**Sign**	**M**	49.5	**Pr >= |M|**	<.0001
**Signed Rank**	**S**	2821	**Pr >= |S|**	<.0001

Quantiles (Definition 5)

Level	Quantile
**100% Max**	142.123423
**99%**	137.759554
**95%**	41.729370
**90%**	30.173946

Quantiles (Definition 5)

Level	Quantile
75% Q3	18.634384
50% Median	9.636520
25% Q1	1.974747
10%	0.170598
5%	0.000000
1%	−1.929024
0% Min	−3.160711

Extreme Observations

Lowest		Highest	
Value	Obs	Value	Obs
−3.1607110	563	49.9376	557
−1.9290244	507	58.9649	526
−0.8514107	486	64.2323	562
−0.0222857	492	137.7596	530
0.0000000	578	142.1234	489

Missing Values

Missing Value	Count	Percent Of	
		All Obs	Missing Obs
.	8	6.72	100.00

Wilcoxon signed-rank test on variable DIFF

The UNIVARIATE Procedure
Variable: diff
AVISIT=week 6 TRTA=A ARM=2.4 mg/g

Moments

**N**	106	**Sum Weights**	106
**Mean**	10.8919473	**Sum Observations**	1154.54641
**Std Deviation**	10.8016797	**Variance**	116.676284
**Skewness**	1.27918905	**Kurtosis**	1.35026505
**Uncorrected SS**	24826.2685	**Corrected SS**	12251.0098
**Coeff Variation**	99.1712448	**Std Error Mean**	1.04915188

Basic Statistical Measures

Location		Variability	
**Mean**	10.89195	**Std Deviation**	10.80168
**Median**	7.47751	**Variance**	116.67628
**Mode**	0.00000	**Range**	54.83896
		**Interquartile Range**	13.86393

Tests for Location: Mu0=0

Test	Statistic		p Value	
**Student's t**	**t**	10.38167	**Pr > |t|**	<.0001
**Sign**	**M**	48	**Pr >= |M|**	<.0001
**Signed Rank**	**S**	2474	**Pr >= |S|**	<.0001

Quantiles (Definition 5)

Level	Quantile
**100% Max**	48.308866
**99%**	42.366253
**95%**	32.927981
**90%**	27.220647

Quantiles (Definition 5)

Level	Quantile
75% Q3	16.328899
50% Median	7.477513
25% Q1	2.464970
10%	0.402493
5%	0.000000
1%	-0.942804
0% Min	-6.530097

Extreme Observations

Lowest		Highest	
Value	Obs	Value	Obs
-6.530097	607	35.4179	615
-0.942804	666	36.6993	614
0.000000	719	40.4764	718
0.000000	671	42.3663	679
0.000000	669	48.3089	661

Missing Values

Missing Value	Count	Percent Of	
		All Obs	Missing Obs
.	13	10.92	100.00

Wilcoxon signed-rank test on variable DIFF

The UNIVARIATE Procedure
Variable: diff
AVISIT=week 6 TRTA=B ARM=0.4 mg/g (HT)

Moments

**N**	113	**Sum Weights**	113
**Mean**	11.1654466	**Sum Observations**	1261.69546
**Std Deviation**	11.4980756	**Variance**	132.205743
**Skewness**	1.10211054	**Kurtosis**	0.43348683
**Uncorrected SS**	28894.4365	**Corrected SS**	14807.0432
**Coeff Variation**	102.979093	**Std Error Mean**	1.08164797

Basic Statistical Measures

Location		Variability	
**Mean**	11.16545	**Std Deviation**	11.49808
**Median**	7.60922	**Variance**	132.20574
**Mode**	0.00000	**Range**	47.67471
		**Interquartile Range**	15.93378

Tests for Location: Mu0=0

Test	Statistic		p Value	
**Student's t**	**t**	10.32263	**Pr > |t|**	<.0001
**Sign**	**M**	53.5	**Pr >= |M|**	<.0001
**Signed Rank**	**S**	3049	**Pr >= |S|**	<.0001

Quantiles (Definition 5)

Level	Quantile
**100% Max**	43.939710
**99%**	42.851894
**95%**	38.208946
**90%**	27.761648

Quantiles (Definition 5)

Level	Quantile
75% Q3	17.359829
50% Median	7.609216
25% Q1	1.426054
10%	0.358431
5%	0.159561
1%	−0.916295
0% Min	−3.735003

Extreme Observations

Lowest		Highest	
Value	Obs	Value	Obs
−3.7350032	795	39.0218	740
−0.9162945	804	39.0681	734
0.0000000	807	41.2061	833
0.0000000	745	42.8519	777
0.0369019	794	43.9397	805

Missing Values

Missing Value	Count	Percent Of	
		All Obs	Missing Obs
.	10	8.13	100.00

Wilcoxon signed-rank test on variable DIFF

The UNIVARIATE Procedure
Variable: diff
AVISIT=week 6 TRTA=D ARM=1.3 mg/g

Moments

**N**	106	**Sum Weights**	106
**Mean**	13.6707062	**Sum Observations**	1449.09486
**Std Deviation**	12.9024924	**Variance**	166.474309
**Skewness**	1.86775078	**Kurtosis**	6.68545051
**Uncorrected SS**	37289.9525	**Corrected SS**	17479.8025
**Coeff Variation**	94.3805842	**Std Error Mean**	1.25320084

Basic Statistical Measures

Location		Variability	
**Mean**	13.67071	**Std Deviation**	12.90249
**Median**	11.21705	**Variance**	166.47431
**Mode**	0.00000	**Range**	82.88811
		**Interquartile Range**	18.29597

Tests for Location: Mu0=0

Test		Statistic	p Value	
**Student's t**	**t**	10.90863	**Pr > |t|**	<.0001
**Sign**	**M**	50	**Pr >= |M|**	<.0001
**Signed Rank**	**S**	2721	**Pr >= |S|**	<.0001

Quantiles (Definition 5)

Level	Quantile
**100% Max**	82.351563
**99%**	49.005886
**95%**	35.651937
**90%**	27.346568

Quantiles (Definition 5)

Level	Quantile
75% Q3	21.418528
50% Median	11.217054
25% Q1	3.122557
10%	0.607527
5%	0.227945
1%	−0.172297
0% Min	−0.536543

Extreme Observations

Lowest		Highest	
Value	Obs	Value	Obs
−0.5365433	876	36.8345	881
−0.1722969	908	38.4019	897
0.0000000	917	42.0201	943
0.0000000	857	49.0059	921
0.0980898	879	82.3516	951

Missing Values

Missing Value	Count	Percent Of	
		All Obs	Missing Obs
.	13	10.92	100.00

Wilcoxon signed-rank test on variable DIFF

The UNIVARIATE Procedure
Variable: diff
AVISIT=week 6 TRTA=F ARM=5.2 mg/g

Moments

N	106	Sum Weights	106
Mean	9.21175826	Sum Observations	976.446375
Std Deviation	8.63536191	Variance	74.5694753
Skewness	1.62568026	Kurtosis	4.8447674
Uncorrected SS	16824.5829	Corrected SS	7829.79491
Coeff Variation	93.7428194	Std Error Mean	0.83874049

Basic Statistical Measures

Location		Variability	
Mean	9.211758	Std Deviation	8.63536
Median	8.620126	Variance	74.56948
Mode	0.000000	Range	52.67497
		Interquartile Range	11.42067

Tests for Location: Mu0=0

Test	Statistic		p Value	
Student's t	t	10.98285	Pr > |t|	<.0001
Sign	M	46.5	Pr >= |M|	<.0001
Signed Rank	S	2275	Pr >= |S|	<.0001

Quantiles (Definition 5)

Level	Quantile
100% Max	51.67497
99%	32.40937
95%	25.48865
90%	19.86183

Quantiles (Definition 5)

Level	Quantile
75% Q3	13.14454
50% Median	8.62013
25% Q1	1.72387
10%	0.00000
5%	0.00000
1%	0.00000
0% Min	−1.00000

Extreme Observations

Lowest		Highest	
Value	Obs	Value	Obs
−1	979	26.3779	982
0	1065	26.6106	983
0	1062	32.0472	1068
0	1057	32.4094	1061
0	1056	51.6750	1069

Missing Values

Missing Value	Count	Percent Of	
		All Obs	Missing Obs
.	16	13.11	100.00

Wilcoxon signed-rank test on variable DIFF

The UNIVARIATE Procedure
Variable: diff
AVISIT=week 6 TRTA=G ARM=0.4 mg/g

Moments

**N**	104	**Sum Weights**	104
**Mean**	13.9148455	**Sum Observations**	1447.14394
**Std Deviation**	13.4954663	**Variance**	182.127611
**Skewness**	1.97271806	**Kurtosis**	8.57883047
**Uncorrected SS**	38895.9283	**Corrected SS**	18759.1439
**Coeff Variation**	96.9861022	**Std Error Mean**	1.32333935

Basic Statistical Measures

**Location**		**Variability**	
**Mean**	13.91485	**Std Deviation**	13.49547
**Median**	13.46990	**Variance**	182.12761
**Mode**	0.00000	**Range**	92.99648
		**Interquartile Range**	19.96945

Tests for Location: Mu0=0

**Test**	**Statistic**		**p Value**	
**Student's t**	**t**	10.51495	**Pr > |t|**	<.0001
**Sign**	**M**	48.5	**Pr >= |M|**	<.0001
**Signed Rank**	**S**	2527.5	**Pr >= |S|**	<.0001

Quantiles (Definition 5)

**Level**	**Quantile**
**100% Max**	90.1867017
**99%**	42.6323427
**95%**	35.5745025
**90%**	29.2593634

Quantiles (Definition 5)

Level	Quantile
75% Q3	21.9624661
50% Median	13.4698961
25% Q1	1.9930179
10%	0.1253591
5%	0.0428667
1%	−1.9829504
0% Min	−2.8097815

Extreme Observations

Lowest		Highest	
Value	Obs	Value	Obs
−2.80978	1165	35.6554	1199
−1.98295	1190	37.3142	1110
0.00000	1178	41.3313	1177
0.00000	1160	42.6323	1166
0.00000	1139	90.1867	1091

Missing Values

Missing Value	Count	Percent Of	
		All Obs	Missing Obs
.	15	12.61	100.00
