# Supplementary material for: Underreporting of non-study cigarette use by study participants confounds the interpretation of results from ambulatory clinical trial of reduced nicotine cigarettes
Source: Harm Reduct J. 2024 Feb 8;21:35. doi: 10.1186/s12954-024-00953-8 (PMC10854148; doi:10.1186/s12954-024-00953-8)
Supplement: Supplementary file 2 — Additional file 2: Statistical analysis SAS codes. [file 12954_2024_953_MOESM2_ESM.pdf]

Supplemental File II – Statistical SAS Codes

[Zhang et al., Underreporting of Non-Study Cigarette Use by Study Participants …]

Statistical Analysis SAS code

1. SAS code for the Wilcoxon signed-rank test:

```
*==== Read in data ====*;
data in; set in.ANALYSIS_DATA;
    where avisit in ('week 2', 'week 6');
        if trta='C' then delete; *C is the reference group, 15.8 mg/g group;
        diff=adj_cal_nonstud_cpd_tf - non_study_CPD; *Estimated Non-study CPD (tobacco filler, negative values were set to 0) minus Self-reported Non-study CPD;
        log_self = log(non_study_CPD + 1);  * +1 because of a lot of 0s;
        log_est =log(adj_cal_nonstud_cpd_tf + 1);
        keep subjid arm trta non_study_CPD avisit adj_cal_nonstud_cpd_tf diff log_self log_est;
run;

proc sort data=in; by avisit trta arm; run;


*==== conduct paired t-test on raw self-reported and estimated non-study CPD, Q-Q plots shows that the data is not normal distributed ====*;
title 'Paired t-test on raw self-reported and estimated non-study CPD'; run;

ods graphics on;

proc ttest data=in;
    by avisit trta arm;
    paired ADJ_CAL_nonstud_CPD_TF*non_study_CPD;
run;

ods graphics off;
```

```
*===== test on DIFF (it shows the same result as above) ====*;
ods graphics on;
*--- t-test on DIFF ----*;
title 't-test on variable DIFF'; run;

proc ttest data=in;
     by avisit trta arm;
     var diff;
run;

ods graphics off;


*==== From above steps, found data (DIFF) is not normally distributed, conduct paired t-test on log transformed CPD
variables ====*;
title 'log transformed CPD variables then conduct paired t-test'; run;

ods graphics on;

proc ttest data=in;
     by avisit trta arm;
     paired log_self*log_est;
run;

ods graphics off;


*=== Above step shows data is still not normal distributed even after log transformation, perform non-parametric Wilcoxon
signed-rank test on variable DIFF ===*;
title 'Wilcoxon signed-rank test on variable DIFF';
proc univariate data=in;
     by avisit trta arm;
            var diff;
```

```
run;

quit;
```

2. Sas code for the linear mixed model analysis:

```
ods output LSMeans=_LSMeansm1 ;
ods output Diffs  =_Diffsm1 ;

Proc mixed data=cpdk1;
        where baseline_cpd ne .;
        Class subjid trt avisit  ;
        Model adj_cal_tot_cpd_tf = trt avisit trt*avisit  baseline_cpd /ddfm=kr;
        Repeated avisit / type=UN subject=subjid;
        lsmeans trt*avisit/cl alpha=0.05 pdiff=control('F' 'week 2' ) adjust=dunnett;
        lsmeans trt*avisit/cl alpha=0.05 pdiff=control('F' 'week 6' ) adjust=dunnett;
run;
```
